# Pathophysiological, Genetic and Gene Expression Features of a Novel Rodent Model of the Cardio-Metabolic Syndrome

**DOI:** 10.1371/journal.pone.0002962

**Published:** 2008-08-13

**Authors:** Robert H. Wallis, Stephan C. Collins, Pamela J. Kaisaki, Karène Argoud, Steven P. Wilder, Karin J. Wallace, Massimiliano Ria, Alain Ktorza, Patrik Rorsman, Marie-Thérèse Bihoreau, Dominique Gauguier

**Affiliations:** 1 The Wellcome Trust Centre for Human Genetics, University of Oxford, Oxford, United Kingdom; 2 Oxford Centre for Diabetes, Endocrinology and Metabolism, University of Oxford, Churchill Hospital, Oxford, United Kingdom; 3 Laboratory of Pathophysiology of Nutrition, CNRS UMR 7059, University of Paris 7, Paris, France; 4 Servier International Research Institute, Courbevoie, France; Mayo Clinic College of Medicine, United States of America

## Abstract

**Background:**

Complex etiology and pathogenesis of pathophysiological components of the cardio-metabolic syndrome have been demonstrated in humans and animal models.

**Methodology/Principal Findings:**

We have generated extensive physiological, genetic and genome-wide gene expression profiles in a congenic strain of the spontaneously diabetic Goto-Kakizaki (GK) rat containing a large region (110 cM, 170 Mb) of rat chromosome 1 (RNO1), which covers diabetes and obesity quantitative trait loci (QTL), introgressed onto the genetic background of the normoglycaemic Brown Norway (BN) strain. This novel disease model, which by the length of the congenic region closely mirrors the situation of a chromosome substitution strain, exhibits a wide range of abnormalities directly relevant to components of the cardio-metabolic syndrome and diabetes complications, including hyperglycaemia, hyperinsulinaemia, enhanced insulin secretion both *in vivo* and *in vitro*, insulin resistance, hypertriglyceridemia and altered pancreatic and renal histological structures. Gene transcription data in kidney, liver, skeletal muscle and white adipose tissue indicate that a disproportionately high number (43–83%) of genes differentially expressed between congenic and BN rats map to the GK genomic interval targeted in the congenic strain, which represents less than 5% of the total length of the rat genome. Genotype analysis of single nucleotide polymorphisms (SNPs) in strains genetically related to the GK highlights clusters of conserved and strain-specific variants in RNO1 that can assist the identification of naturally occurring variants isolated in diabetic and hypertensive strains when different phenotype selection procedures were applied.

**Conclusions:**

Our results emphasize the importance of rat congenic models for defining the impact of genetic variants in well-characterised QTL regions on *in vivo* pathophysiological features and cis-/trans- regulation of gene expression. The congenic strain reported here provides a novel and sustainable model for investigating the pathogenesis and genetic basis of risks factors for the cardio-metabolic syndrome.

## Introduction

The combination of a decreased sensitivity to insulin of target tissues (insulin resistance) and compensatory hyperinsulinaemia predispose to the development of a collection of abnormalities including glucose intolerance, obesity, dyslipidemia (increased plasma triglycerides and decreased HDL cholesterol) and hypertension [1], [2]. These changes represent pathophysiological characteristics of the cardio-metabolic syndrome and important risk factors for coronary heart disease [3], [4]. Studies in animal models that spontaneously exhibit features of the human syndrome contribute to the identification of causative genes for components of the cardio-metabolic syndrome.

The wealth of phenotypic information that can be collected in inbred strains of the laboratory rat makes it a powerful tool for genetic studies of quantitative traits underlying complex phenotypes [5]. Related quantitative trait loci (QTL) identified in crosses derived from different disease rat strains often cluster [6], [7], suggesting that a proportion of genetic variants isolated in strains selectively bred for different pathologies (type 2 diabetes, hypertension, obesity) are common and underlie shared pathophysiological mechanisms. Rat chromosome 1 (RNO1) which harbors QTLs for phenotypes relevant to the cardio-metabolic syndrome and renal failure [6], [8], [9], [10], [11], [12], [13], [14] is a prime example of this situation. The fact that both the spontaneously hypertensive rat (SHR) and the diabetic Goto Kakizaki (GK) rat carry a naturally occurring functional polymorphism in a gene encoding an inositol 5-phosphatase (SHIP2), illustrates the existence of common genetic and pathophysiological backgrounds in these strains which originate from a similar Wistar outbred stock [5]. Similarly, both GK and Otsuka Long Evans Tokushima Fatty (OLETF) strains carry a mutation in a G-protein receptor (GPR10) primarily associated with obesity [15].

Inbred congenic strains are powerful systems for profiling *in vivo* effects of genes underlying a QTL on a broad range of phenotypes [16]. Chromosome substitution strains (CSS) designed to carry the entire length of a chromosome transferred from a disease strain onto the genetic background of a control strain have been proposed as novel tools facilitating phenotypic profiling of large genomic regions [16], [17]. We have developed a QTL substitution congenic strain containing GK alleles across a 110 cM region of RNO1 covering known diabetes and obesity QTLs *Nidd/gk1*, transferred onto a Brown-Norway (BN) genetic background [8], [12]. By the length of the targeted chromosomal region this congenic strain closely resembles the situation of a CSS. Phenotype and genetic studies in an experimental cross derived from this congenic strain have demonstrated that GK alleles in this region account for the differences in glucose tolerance between the congenics and the BN control [18].

Here, we carried out extensive phenotypic, genetic and genome-wide gene expression analyses in this congenic strain to demonstrate the combined impact of GK gene variants present at the locus *Nidd/gk1* on a wide range of phenotypes involved in the cardio-metabolic syndrome and diabetes complications and on cis- and trans- mechanisms of gene expression regulation in key organs (kidney, liver, skeletal muscle and white adipose tissue) for these diseases. This novel disease model exhibits fasting hyperglycaemia and hyperinsulinaemia, enhanced insulin secretion, tissue specific insulin resistance hypertriglyceridemia and structural changes in the endocrine pancreas and the kidney. Analysis of single nucleotide polymorphisms (SNPs) in rat strains revealed large chromosomal regions conserved between GK, SHR and Wistar-Kyoto (WKY) normoglycaemic controls, which can assist in the identification of naturally occurring variants selected in both diabetic and hypertensive strains and causing changes in phenotypes involved in the cardio-metabolic syndrome.

## Materials and Methods

### Animals

We used a colony of GK/Ox rats bred locally and derived in 1995 from the GK/Par colony. BN rats were obtained from a commercial supplier (Charles River Laboratories, Margate, UK). The QTL substitution congenic strain BN.GK-D1Wox18/D1Got254 (also referred as BN.GK-Nidd/gk1 throughout the text) derived from these strains using a genetic marker assisted breeding strategy (“speed congenics”) as previously described [19] was maintained by brother-sister mating. This congenic was specifically designed to contain GK alleles over a 170 Mb (110 cM) genomic interval of chromosome 1 between markers D1Wox18 (94.63 Mb) and D1Got254 (264.37 Mb), covering the QTL *Nidd/gk1*, as well as other QTLs, introgressed onto the genetic background of the BN strain. Early in the breeding programme, GK alleles on the X chromosome were lost by two consecutive breedings of male backcross animals to BN females. All animals used in this study were systematically genotyped as previously described [20] with microsatellite markers chosen to accurately monitor retention of GK alleles in the congenic interval and absence of GK allele contaminants from the genetic background.

All experiments were carried out with male congenic rats and controls. Animal procedures were approved by the ethical review panel of the University of Oxford and UK Home Office licences. Rats were allowed free access to tap water and standard laboratory chow pellets (B&K Universal Ltd, Grimston, Aldbrough, Hull) and were maintained on a 12-h light-dark cycle. All rats were identified using a microchip (identity chip, Animal Care Ltd, York, UK). Several aspects of the project (husbandry, phenotype scheduling and data storage) was administrated using a database specifically designed for congenic-based projects [21].

### Body weight, tissue weight and basal metabolism

Body weight of the animals was determined at 2, 4, 12 and 24 weeks. At 12 and 24 weeks blood samples were collected on EDTA via the tail vein of conscious rats fasted for 4 hours (post-absorptive state) to measure plasma glucose and insulin, and rats fasted overnight for 16–18 hours (fasting state) to determine plasma concentrations of glucose, insulin and lipids. Plasma was obtained immediately and stored at −80°C. Retroperitoneal fat pads (RFP) were dissected in rats killed by anesthetic overdose and weighed. Adiposity index was calculated as the ratio between RFP weight and body weight.

Plasma concentrations of glucose, insulin, total cholesterol, HDL-cholesterol, LDL-cholesterol, triglycerides, and phospholipids were determined using commercial diagnostic kits (ABX, Shefford, UK) on a Cobas Mira Plus automatic analyser (ABX, Shefford, UK). Plasma NEFA were quantified on the Cobas Mira Plus using a kit from Wako (Alpha Laboratories, Eastleigh, UK). Immunoreactive insulin (IRI) was measured with an ELISA kit (Mercodia, Uppsala, Sweden).

### In vivo arginine induced insulin secretion test

Intraperitoneal arginine tests were performed in fasted animals at 24 weeks of age as previously described [12]. Rats were anesthetised using ketamine hydrochloride (95 mg/kg body weight). A solution of L-arginine hydrochloride (0.8 g/kg body weight) was injected. Blood samples were collected on heparin before the injection and 5, 10, 15, 20 and 30 minutes afterwards. Samples were spun and plasma separated for determination of plasma IRI.

### Static incubation of pancreatic islets

Insulin secretion was measured in static incubation following overnight culture in RPMI1640 containing 5 mM glucose. Groups of 10 size-matched islets were washed twice in 300 µl of RPMI 1640 without glucose and pre-incubated at 37°C for 1 hr in a solution of 120 mM NaCl, 4.73 mM KCl, 2.5 mM CaCl_2_, 1.19 mM KH_2_PO_4_, 1.18 mM MgSO_4_, 10 mM Hepes (pH 7.4 with NaOH), 25 mM NaHCO_3_ supplemented with BSA 0.5 mg/ml and 1 mM glucose. Sample tubes were centrifuged for 2 min at 90 g and the supernatant replaced with 300 µl of EC1 supplemented with glucose and tolbutamide as indicated. Islets were then incubated for 1 hr at 37°C. The test tubes containing the islets were then centrifuged for 1 min at 90 g and the supernatant withdrawn for analysis. Pelleted islets were kept in 100 µl acid-alcohol (ratio of ethanol∶H_2_O∶HCl was 52∶17∶1) at −20°C until hormone measurement.

### Blood pressure and tissue-specific insulin sensitivity (euglycaemic-hyperinsulinaemic clamp)

Rats in a post-absorptive state were anesthetised with an intraperitoneal injection of pentobarbitone (30 mg/kg body weight). One carotid artery was freed from the vagus nerve and a catheter placed and connected to a 4 channel Biopac system with two blood pressure transducers (SS13L, Santa Barbara, CA). Arterial blood pressure was recorded for about 15 minutes once the blood pressure had stabilized.

A tracheotomy was then performed prior to the euglycaemic-hyperinsulinaemic clamp. Clamps were performed with an infusion rate of 20 µl of insulin (0.6 U/kg/h) (Humulin S, Lilly, UK) per minute. Exogenous glucose was simultaneously infused through the saphenous vein in order to maintain euglycaemia. Glycaemia was monitored for about 40 minutes until a plateau was reached for both glycaemia and exogenous glucose infusion rate. A solution of 2-Deoxy[1-^3^H]glucose (2DOG, Amersham, UK) (30 µCi) prepared in 400 µl of 0.9% NaCl was then injected via the saphenous vein. Blood samples were collected via the arterial catheter 3, 5, 10, 20, 40, 60 minutes after the injection of 2DOG. Glycaemia was immediately determined using a pocket analyzer (Bayer, UK). Blood samples were collected in parallel, deproteinised in Ba(OH)_2_/ZnSO_4_ and centrifuged. Pellets were left to evaporate and solubilised in water for 2DOG quantification as described below.

At the end of the experiment (60 minutes post 2DOG injection), rats were killed by pentobarbitone overdose. Skeletal muscles (extensor digitorum [ED]-slow twitch muscle and soleus -fast twitch muscle), liver, pancreas, RFP, brown adipose tissue (BAT), atrium and ventricle were harvested, weighed and immediately immersed in NaOH (1M) at 60°C for 1 hour until tissues were dissolved. A solution of 0.5 ml HCl (1M) was then added to each tissue digest and a 200 µl aliquot was mixed with a solution of 6% HClO_4_ (perchloric acid, PCA) and Ba(OH)_2_/ZnSO_4_. After centrifugation the supernatant was counted with the deproteinised blood samples. The concentration of 2DOG was determined with the equation:
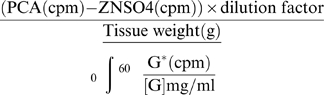



### Pancreas and renal histology

At 6 months rats were fasted overnight and killed. The tail of the pancreas and the right kidney were taken. The tail of the pancreas was cut in half and the half closest to the spleen was fixed in a solution of Bouin and then transferred to 70% EtOH. Samples were embedded in paraffin and sliced (7 µm) on a Leica RM 2155 microtome. To improve accuracy of pancreas histology, sections were taken at 10 different areas along the pancreas (150 to 200 µm between areas). A technique of indirect labelling was used for immunohistochemistry. Slides were rehydrated and incubated with an anti-porcine insulin antibody (ICN). Slides were then incubated with a peroxidase-conjugated rabbit anti-guinea pig IgG (Dako, Ely, UK) and stained using a peroxidase substrate kit (DAB, Vector Laboratories, Peterborough, UK). Islets were imaged using an Olympus BX-51 microscope. The Metamorph software was used to capture the images and the ImageJ software available from NIH (http://rsb.info.nih.gov/ij/) was used to determine the area of the pancreas and the islets.

Kidneys were fixed in a solution of picric acid containing fixative (Dubosq-Brazil). After dehydration, kidneys were embedded in paraffin and sectioned at 5 µm. Following staining with hematoxylin and eosin (H&E) or periodic acid Schiff (PAS) reagents, sections were examined qualitatively to assess glomerular and tubular damages, thickening of glomerular basement membranes, changes in capillary network, and presence of intratubular casts.

### SNP-based genotype analysis of the congenic interval in BN and Wistar related strains

Genotype data of over 20,000 rat SNPs determined in BN and BN.GK-Nidd/gk1 congenic rats, and in various colonies of WKY and SHR rats were obtained from the STAR project (http://www.snp-star.eu/). Full details of rat SNPs mapped to the congenic interval are publicly available through http://www.well.ox.ac.uk/rat_mapping_resources/SNPbased_maps.html and summarized in Table S1.

### RNA preparation

RNA samples were individually prepared from total kidney, liver, skeletal muscle (soleus) and RFP from six BN and six congenic rats. Total RNA was extracted twice using Trizol reagents (Invitrogen Life Technologies, Paisley, UK) and purified with RNeasy columns (Qiagen Ltd., Crawley, UK). RNA quality was determined with an Agilent 2100 Bioanalyzer (Agilent Technologies, Waldbronn, Germany).

### Hybridization to Affymetrix gene expression arrays

cDNA synthesis was performed with individual RNA samples (no pooling) using Superscript Double-Stranded cDNA Synthesis kits (Invitrogen Ltd., Paisley, UK). Biotin-labeled cRNA was synthesised using GeneChip® IVT Labelling Kit and purified using the GeneChip® Sample Cleanup Module (Affymetrix ltd., High Wycombe, UK). Each biotinylated cRNA (15 µg) was fragmented and individually hybridized to GeneChip Rat expression Arrays 230 2.0 containing over 31,000 probe sets representing approximately 28,700 different genes (Affymetrix ltd., High Wycombe, UK). Washing and staining procedures were performed with streptavidin-phycoerythrin (SAPE) on a fluidics station 450 according to the manufacturer's protocols (Affymetrix ltd, High Wycombe, UK). Arrays were scanned at 570 nm using an array scanner (Agilent Technologies, Waldbronn, Germany). Microarray experiments were compliant with MIAME (Minimum Information About a Microarray Experiment) and both protocol details and raw data have been deposited in ArrayExpress (http://www.ebi.ac.uk/arrayexpress/) under the accession number E-MEXP-1695.

### Statistical analyses

Statistical analysis of phenotypic data was performed using SPSS v.11.0. To ensure the homogeneity of collected data across several litters and generations of congenic and control rats, a GLM testing for various factors (such as seasonal variation, litter size, date of birth, date of experiment) was performed for each phenotype. When the effect of such factors was negligible, an independent Student's t-test was used to compare means of phenotypic data between the congenic and control strains.

Statistical analysis of gene transcription profiling data was carried out with at least five array experiments per group. Analyses were performed with the Affymetrix CEL files using the Bioconductor packages in the R language and environment as previously described [22]. Affymetrix data were normalized using the RMA method and the linear model for microarray data (LIMMA) package was used to assess statistical significance between groups.

## Results

### Body weight and adiposity index

**Figure 1:**
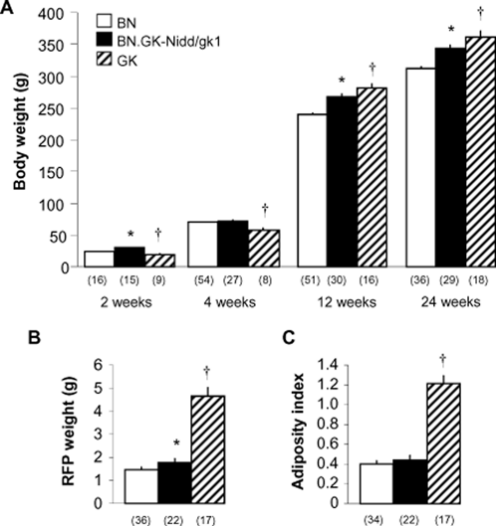
Follow-up measure of body weight in BN, GK and BN.GK-Nidd/gk1 congenic rats (A), and retroperitoneal fat pad (RFP) weight (B) and adiposity index (C) in 24 weeks old rats. Data are expressed as means±SEM. Number of rats tested is in parentheses. *P<0.01 significantly different between congenic and BN rats. ^†^P<0.01 significantly different between congenic and GK rats.

Body weight at 2 weeks was higher in congenics than in BN (Fig. 1A) (P = 4.9×10^−7^). At weaning (4 weeks) body weight was not statistically different between the two groups, but at 12 and 24 weeks, congenic rats were significantly heavier than BN controls (P = 3.2×10^−7^ and 3.8×10^−6^, respectively). Congenic rats were heavier than GK until weaning. At 12 and 24 weeks, GK rats were significantly heavier than congenics. RFP weight was significantly higher in congenics than in BN rats (Fig. 1B). As a consequence adiposity index was increased in congenics when compared to BN (Fig. 1C), but this effect was statistically significant (P = 3.3×10^−4^) in females only (data not shown). Both RFP weight and adiposity index were significantly lower in congenics than in GK rats. Overall these data indicate that GK genetic variants in the congenics account for a proportion of the phenotypic differences between GK and BN rats.

### Plasma lipid profile and glucose homeostasis

**Figure 2:**
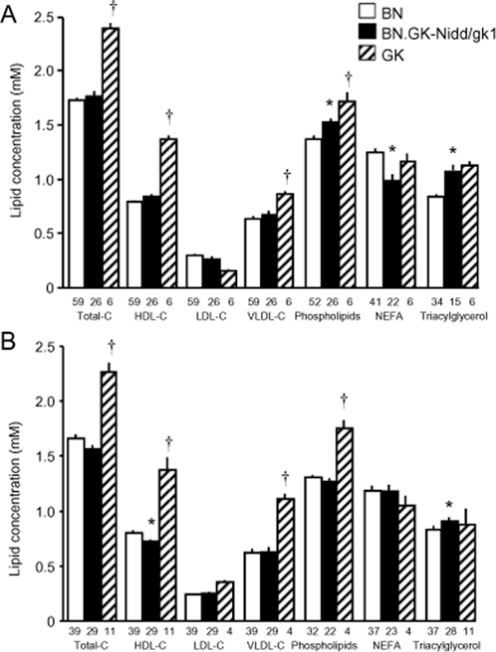
Plasma lipid profile in 12- (A) and 24-weeks old (B) BN, GK and BN.GK-Nidd/gk1 congenic rats. Data are expressed as means±SEM. Number of rats used is reported at the bottom of the charts. *P<0.01 significantly different between congenic and BN rats. ^†^P<0.01 significantly different between congenic and GK rats.

When compared to BN controls, circulating levels of several lipid variables were transiently altered in congenic animals at 12 weeks (Phospholipids, P = 3.0×10^−3^; NEFA, P = 4.0×10^−4^) or 24 weeks (HDL cholesterol, P = 3.0×10^−3^) (Fig. 2). Plasma concentrations of total, LDL and VLDL cholesterol were similar in congenic and BN control rats, but significantly lower than in GK rats. At both 12 and 24 weeks, concentration of circulating triacylglycerols was significantly higher in congenics than in BN controls, to reach levels exhibited by GK rats. These results suggest that GK variants captured in BN.GK-Nidd/gk1 congenic rats have a strong impact on triacylglyceride metabolism in the GK strain whereas loci elsewhere in the genome control the concentration of plasma cholesterol.

At 12 and 24 weeks of age, congenic rats in the post-absorptive state (4 hours- fasting) exhibited significant elevation of glycaemia (P = 4.0×10**^−4^**) and insulinaemia (P<0.05) when compared to age-matched BN controls (Table 1). Fasted congenic rats showed mild but statistically significant hyperglycaemia when compared to BN at 12 weeks only (P = 1.1×10**^−4^**), and a 27–40% reduction of insulinaemia at both 12 and 24 weeks, but these differences were not statistically significant (P = 0.06). These results suggest insulin resistance in post-absorptive congenic rats and differential adaptation to fasting in congenic and BN rats, which may affect triacylglycerol metabolism. Marked hyperglycaemia and hyperinsulinaemia in the GK strain when compared to BN.GK-Nidd/gk1 congenic rats indicates that genes outside the GK interval targeted in this congenic strain influence glucose regulation.

**Table 1:** Glycaemia, insulinaemia and blood pressure variables in male BN, GK and BN.GK-Nidd/gk1 congenic rats.

		Age	Strains		
		(weeks)	BN	BN.GK-Nidd/gk1	GK
Glycaemia (mg/dl)	Post-absorptive	12	128.3±4.0 (30)	136.3±2.4 (31) *	193.67±7.33 (6) †
		24	124.5±2.8 (26)	138.2±3.6 (19) *	227.71±6.52 (8) †
	Fasting	12	100.26±0.01 (26)	116.14±0.03 (14) *	175.73±1.30 (12) †
		24	96.10±0.01 (17)	91.17±0.05 (16)	148.41±2.42 (4) †
Insulinaemia (µg/ml)	Post-absorptive	12	0.75±0.13 (29)	1.54±0.16 (31) *	2.17±0.11 (6) †
		24	1.12±0.12 (24)	2.21±0.22 (20) *	3.26±0.06 (8) †
	Fasting	12	0.26±0.01 (17)	0.18±0.05 (16)	0.51±0.09 (8) †
		24	0.26±0.01 (16)	0.12±0.07 (5)	0.64±0.17 (4) †
Mean BP (mmHg)		12	139±13 (6)	139±11 (5)	178±5 (6) †
Diastolic BP (mmHg)		12	125±13 (6)	123±10 (5)	218±6 (6) †
Systolic BP (mmHg)		12	163±16 (6)	156±13 (5)	201±6 (6) †
Heart rate (BPM)		12	311±75 (6)	405±60 (5)	318±26 (6)

Post-absorptive values were obtained after a 4 hour-fast and fasting values after a 12 hour-fast. Data are expressed as means±SEM. Number of animals tested is in parentheses.

* P<0.05 significantly different between congenic and BN rats.

† P<0.05 significantly different between congenic and GK rats.

Owing to the magnitude of phenotype differences between GK and congenics, which are likely caused by the combined effects of numerous GK variants outside the congenic region in BN.GK-Nidd/gk1 rats, further analyses of insulin sensitivity and *in vivo* and *in vitro* insulin secretion, already well-characterized in GK , were limited to BN and congenic rats.

### In vivo arginine induced insulin secretion

**Figure 3:**
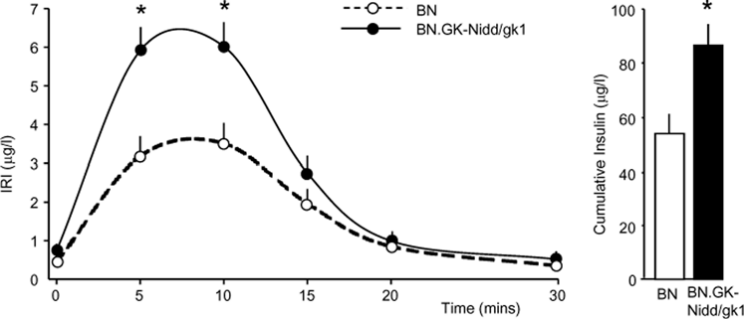
Insulin response to arginine stimulation in 24 weeks old male rats of the BN and BN.GK-Nidd/gk1 congenic strains. Immunoreactive insulin (IRI) data from BN (n = 15) and congenic (n = 6) rats are expressed as means±SEM. *P<0.01 significantly different between congenic and BN rats.

To investigate possible causes of hyperinsulinaemia in BN.GK-Nidd/gk1 congenics, insulin response to an injection of arginine *in vivo* was tested. Congenic rats showed significantly greater insulin secretion 5 and 10 minutes after the arginine injection than BN rats (P<0.005) (Fig. 3). As a consequence, the cumulative insulin response during the test was also significantly enhanced by over 25% in congenics when compared to BN (P = 0.008), thus reflecting an overall greater capacity of congenic rats to secrete insulin than BN.

### In vitro glucose-induced insulin secretion

**Figure 4:**
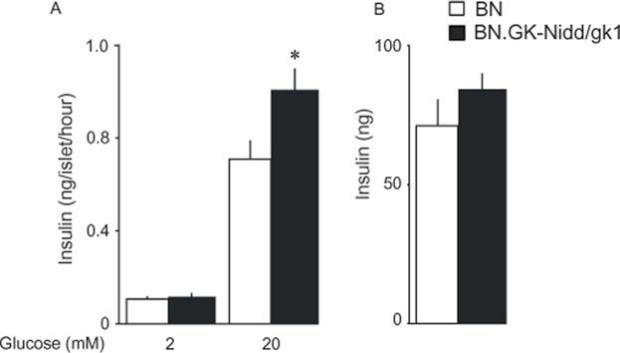
Insulin response to glucose in isolated islets from BN.GK-Nidd/gk1 congenic and BN controls. Insulin secretion in response to 2 or 20 mM glucose from batch incubation of islets from BN (n = 7) and congenics (n = 9) (A) and insulin content derived from the same experiment (B) are shown. *P<0.01 significantly different between congenics and BN rats.

To test whether functional defects in pancreatic islets account for these and previously reported [12], [18] *in vivo* stimulatory effect of GK alleles in chromosome 1 on insulin secretion in the congenics, we studied glucose induced insulin secretion in isolated islets. Islets from BN controls responded by a 12-fold increase in insulin secretion when stimulated by high (20 mM) glucose (Fig. 4A), whereas islets from congenic rats showed insulin secretion increased by 15-fold upon stimulation, an effect that was significantly higher than in BN islets. Insulin content was not statistically different in BN and congenic strains (P = 0.2) (Fig. 4B).

### Insulin sensitivity

**Figure 5:**
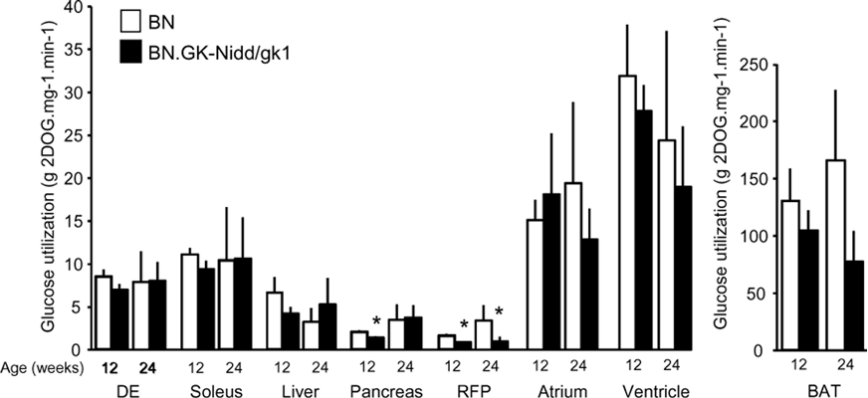
Insulin stimulated glucose uptake and utilization in 12 and 24 weeks old male rats of the BN and BN.GK-Nidd/gk1 congenic strains. Content of 2-Deoxy [1-3H]glucose per mg of tissue and per minute was measured for several organs. Data are expressed as means±SEM. Data are from 18 rats at 12 weeks (5 BN and 13 congenics) and 16 rats at 24 weeks (10 BN and 6 congenics). DE, Digitalum extensorum; RFP, Retroperitoneal fat pad; BAT, Brown adipose tissue. *P<0.01, **P<0.001 significantly different between congenic and BN rats.

Euglycaemic-hyperinsulinaemic clamps coupled with 2DOG quantification in organs were used to assess *in vivo* tissue specific glucose transport and utilization stimulated by insulin in congenics and controls. At 12 and 24 weeks, 2DOG content in non-oxidative and oxidative skeletal muscles (DE and soleus, respectively), liver, atrium and ventricle was similar in BN and congenic rats (Fig. 5). The concentration of 2DOG in BAT was lower in congenics than in BN rats but differences were not statistically significant (P = 0.08). We noted a significant reduction of pancreatic levels of 2DOG in 12 week old congenic rats when compared to BN. At both 12 and 24 weeks, the concentration of 2DOG in the RFP was consistently significantly lower in congenic than in BN, indicating reduced glucose transport and/or utilization in congenics. These results show that congenic rats develop evidence of persistent reduced biological effects of insulin (insulin resistance), which are limited to white adipose tissue.

### Blood pressure

To further document cardiovascular phenotypes linked to rat chromosome 1 QTLs and refine insulin resistant variables in BN.GK-Nidd/gk1 congenics, arterial blood pressure was analysed in 24 weeks-old congenics and controls. Measures of systolic, diastolic and mean blood pressure were similar in the rat groups (Table 1). Heart rate was increased by 30% in congenic when compared to BN but differences were not statistically significant (P = 0.19). These results demonstrate that GK alleles at the locus *Nidd/gk1* do not significantly alter blood pressure regulation. In contrast, increased blood pressure in GK when compared to BN and congenics indicates that hypertensive genetic variants may exist elsewhere in the GK genome.

### Pancreas and renal histology

**Figure 6:**
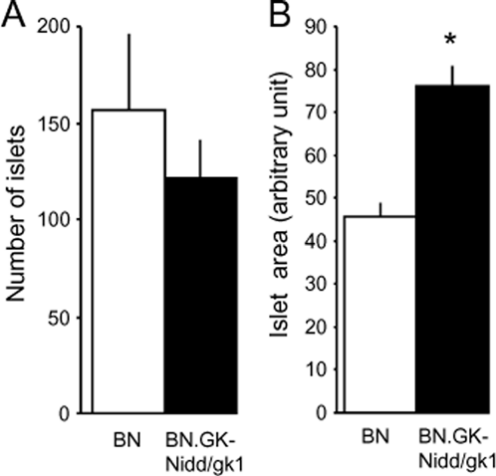
Endocrine pancreas morphological features in BN and BN.GK-Nidd/gk1 congenic strains. Four sections of pancreas spaced by at least 150 µm were used to determine islets' number (A) and average size (B). The numbers of islets were normalized to the size of the pancreas section. Data are expressed as means±SEM. *P<0.001, significantly different between congenic and BN rats.

**Figure 7:**
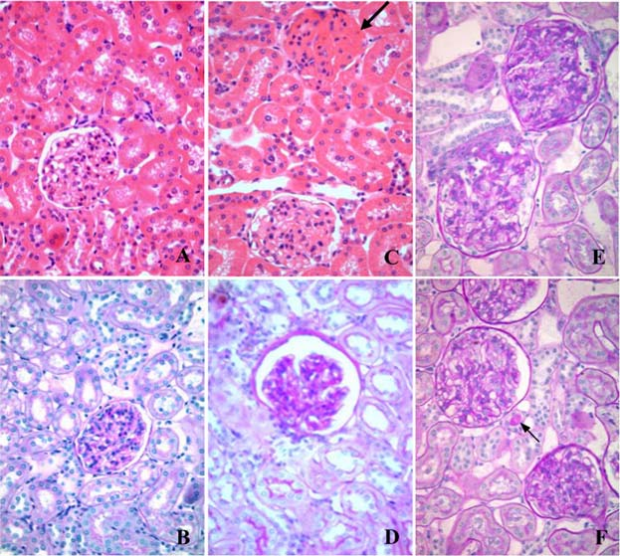
Representative photomicrographs of H&E (A, C) and PAS (B, D, E, F) stained kidney sections of BN (A, B), BN.GK-Nidd/gk1 congenic (C, D) and GK rats (E, F). Focal glomerular lesions, including thickening of the glomerular basement membrane, mesangial extracellular matrix expansion and deposition of fibrous material within the Bowman's capsule were observed in kidneys of congenic rats (C, arrow). Original magnification: ×400.

Pancreas and renal morphological features were analyzed to refine phenotype characterization of BN.GK-Nidd/gk1 congenic rats in relation to diabetes and cardiovascular traits mapped to rat chromosome 1 QTLs. The number of islets was similar in congenics and BN (Fig. 6). However, the average size of the islets was increased by over 65% in congenic when compared to BN rats (P = 6.4×10^−7^), which may contribute to hyperinsulinaemia and enhanced insulin secretion in congenics. Rats of the congenic strain exhibited glomeruli with varying degrees of deposition of eosinophilic hyaline material on the basement membranes and within the mesangium (Fig. 7C, arrow), together with glomeruli of normal morphology (Figs. 7C, 7D) when compared to kidney sections of BN rats, which showed normal glomerular and tubular morphology (Figs. 7A, 7B). In addition, tubulointerstitial histopathologic changes were noted in renal cortex of congenics. They mainly consisted of focal areas of protein/carbohydrate complex cast formations in the distal tubules and early tubular nephrosis (data not shown). Sections of GK kidneys showed evidence of deposition of eosinophilic hyaline material on glomerular and tubular basement membranes and within the mesangium (Fig. 7E) and presence of PAS positive cast within a distal tubule (Fig. 7F, arrow).

### SNP-based genotype analysis of BN.GK-Nidd/gk1 congenic, BN and Wistar related strains

Recently produced genotype data of rat SNP markers in rat strains were used to assess the extent of genetic divergence between the BN.GK-Nidd/gk1 congenic strain and other inbred strains genetically or physiologically relevant to the study. Genome-wide determination of rat SNPs in BN and congenic rats accurately defined the region of chromosome 1 of GK origin introgressed onto the genetic background of the BN strain in congenic rats and verified the absence of GK contaminants throughout the genetic background of the congenic strain. Over 12,000 SNPs were typed, including 1427 SNPs mapped to the congenic interval, which spans 178 Mb (from ENSRNOSNP2783757 located at position 89.99 Mb to ENSRNOSNP2785224 at 267.83 Mb, corresponding to the telomeric end of RNO1).

**Figure 8:**
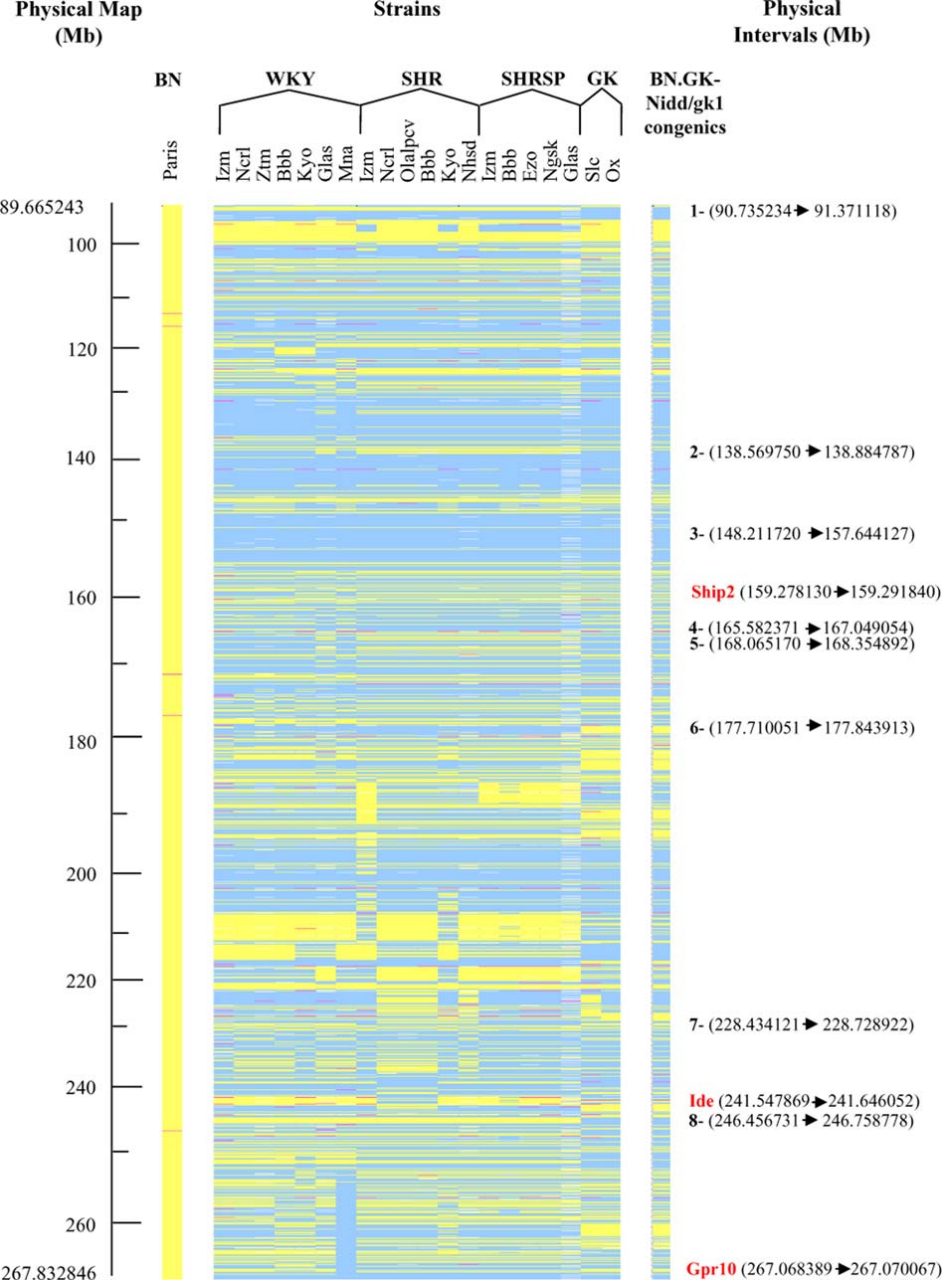
SNP-based genetic similarities and differences between BN, GK, WKY, SHR and SHRSP in the region of chromosome 1 of GK origin targeted in BN.GK-Nidd/gk1 congenic rats. Genotype data from several colonies of WKY, SHR and SHRSP are reported. For each marker, alleles shared between the BN and one or several of these strains are shown in yellow and alleles different to the BN are shown in blue. Heterozygous loci are shown in pink. Examples of intervals showing SNP genotype conservation or difference between stains are reported on the right hand side. The position of genes containing functional variants in the GK rat is reported in red. Full details of SNP markers and genomic positions are given in Table S1. SNP marker details are available through http://www.well.ox.ac.uk/rat_mapping_resources/SNPbased_maps.html.

Genotype data of rat SNPs localized in the congenic interval of the BN.GK-Nidd/gk1 strain provided an accurate determination of genetic similarities and divergences between the BN and BN.GK-Nidd/gk1 strains and between the BN.GK-Nidd/gk1 strain and colonies of Wistar-related strains, i.e. WKY, SHR and SHRSP (Table S1). There were many blocks of genotype conservation between BN.GK-Nidd/gk1 congenics, GK and colonies of WKY, SHR and SHRSP rats, including a large region between 148.2 Mb and 157.6 Mb (interval 3) (Fig. 8). Conversely, BN.GK-Nidd/gk1 congenic and GK strains exhibited specific alleles in several genomic regions (e.g. intervals 1 and 8). In a number of chromosomal segments (e.g. interval 6), all Wistar-related strains but the congenic, GK and BN strains shared the same alleles, whereas in other short genomic regions (intervals 2 and 5) congenic, GK and WKY strains carried the same alleles, which were different to BN and the hypertensive strains. In other instances (intervals 4 and 7), the hypertensive strains have different alleles compared to the other strains including BN (Fig. 8). Of note a block of genetic heterogeneity was detected in GK/Ox and GK/Slc colonies in the genomic interval 223.8 Mb–227.4 Mb.

### Gene transcription regulation in the congenic strain

**Figure 9:**
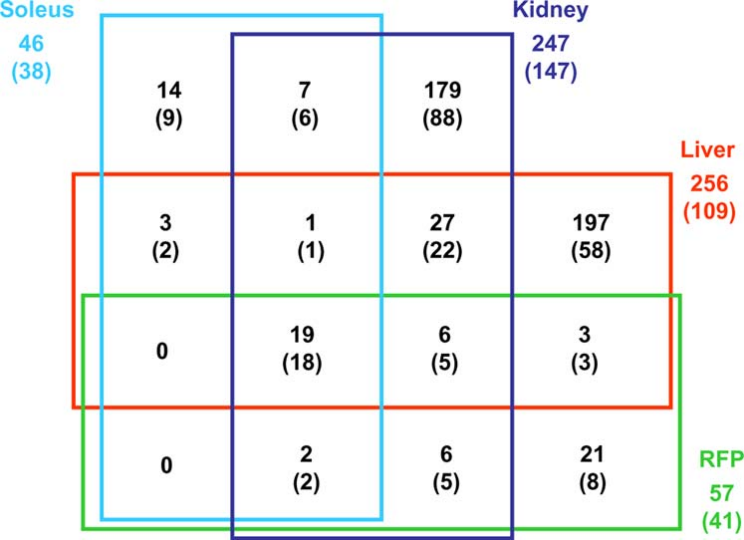
Tissue-specific patterns of genes differentially expressed between BN.GK-Nidd/gk1 congenics and BN controls. Total number of genes showing significant differential expression (P<0.05) between congenics and BN controls in kidney, skeletal muscle (soleus), liver and adipose tissue (retroperitoneal fat pads -RFP) are reported. The number of differentially expressed genes mapped to the GK genomic region in the congenic strain is reported in parentheses. Details of differentially expressed genes are given in Table S2.

**Figure 10:**
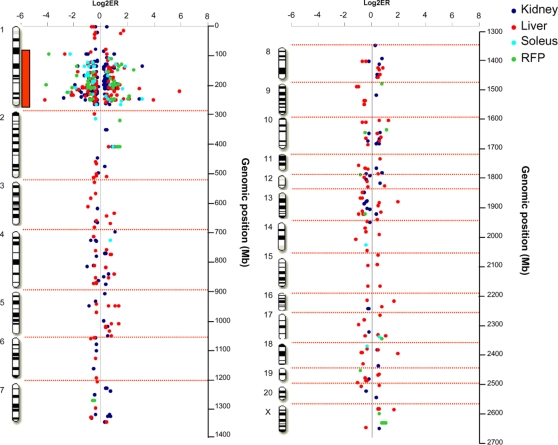
Genomic position of genes differentially expressed between BN.GK-Nidd/gk1 congenics and BN controls. Expression ratio (ER) of genes significantly differentially expressed (P<0.05) between congenics and BN in kidney, liver, skeletal muscle (soleus) and adipose tissue (retroperitoneal fat pads -RFP) is shown. The red bar along chromosome 1 indicates the length of the GK genomic region (89.99–267.83 Mb) in BN.GK-Nidd/gk1 congenics. Details of differentially expressed genes are given in Table S2.

To test a possible correlation between genotype conservation in congenic and control strains and gene expression regulation, microarray based analysis of gene transcription was carried out with biopsies of tissues (kidney, liver, skeletal muscle and white adipose tissue) directly relevant to insulin resistance and renal structural changes observed in BN.GK-Nidd/gk1 congenic rats. Evidence of significant differential transcription (P<0.05) between congenics and BN controls was observed for 516 probesets corresponding to 478 distinct genes (Table S2). Gene expression patterns were fully replicated by different probesets for 38 genes and partly discrepant results were observed for 7 genes. Gene differential expression between congenics and BN was predominantly tissue-specific, with 411 genes (79.7%) showing evidence of differential regulation in one tissue only (Fig. 9). Remarkably, a very high proportion of genes differentially expressed in kidney (60%), liver (43%), skeletal muscle (83%) and white adipose tissue (72%) actually map to the GK chromosomal introgressed in the BN.GK-Nidd/gk1 congenics, even though this strain and the BN control are genetically divergent over only 5% of their genome, as illustrated in Fig. 10. This observation strongly suggests the existence of predominantly cis-acting influences on gene expression regulation in congenic strains.

## Discussion

We have characterized the wide range of pathophysiological features of the cardio-metabolic syndrome and genome-wide gene expression patterns that are altered in rats of the BN.GK-Nidd/gk1 congenic strain designed to carry alleles of the spontaneously diabetic (type 2) GK rat across diabetes and obesity QTLs mapped to RNO1. Hyperglycaemia, hyperinsulinaemia, hypertriglyceridemia, enhanced stimulated insulin secretion, insulin resistance and increased adiposity, develop as a consequence of gene variants in the targeted chromosomal interval that can be mapped using SNP genotype data analysis identified in rat strains genetically related to the GK. Phenotypic features in congenic rats are significant when compared to BN controls, but of modest effect size when compared to GK rats, supporting the complex and polygenic nature of diabetes phenotypes in the GK strain.

Previously documented glucose intolerance and enhanced glucose-stimulated insulin secretion *in vivo* in BN.GK-Nidd/gk1 congenics [18] have validated the most significant phenotypic features of the QTLs originally detected in the (GKxBN) F2 cross [12] and further confirmed in an F2 cross derived from BN.GK-Nidd/gk1 congenic rats [18]. We have now demonstrated that rats of this strain show a much broader spectrum of hormonal and metabolic abnormalities. Enhanced insulin secretion in the congenics both *in vitro* and *in vivo* in response to glucose or arginine suggests that GK alleles in this region of RNO1 collectively affect β-cell function and consequently insulin release [23], [24]. This finding corroborates that at least in part there is an alteration in β-cell depolarization or exocytosis, thus supporting changes in the secretory machinery.

Increased islet size rather than number in BN.GK-Nidd/gk1 congenics when compared to BN rats is an additional important feature that may contribute to enhanced insulin release. These results are however inconsistent with impaired insulin secretion described in a GK.F344 congenic strain targeting a short RNO1 region of the GK QTL *Niddm1* [25], [26], suggesting either the predominant enhancing role of GK alleles of the BN.GK-Nidd/gk1 congenics outside the GK.F344 congenic region on insulin secretion, or differential effects of BN and F344 alleles on the expression of RNO1 GK variants on insulin secretion. SNP genotype data in RNO1 suggest that genetic differences between GK colonies do not account for these phenotypic discrepancies, as heterogeneity between GK/Ox and GK/Slc are located in a genomic interval (223.8 Mb–227.4 Mb) proximal to the GK region introgressed in GK.F344 congenic strains [26] (252 Mb–268 Mb).

The overall net effect of GK variants in the BN.GK-Nidd/gk1 congenics on insulin secretion contributes to hyperinsulinaemia and possibly insulin resistance. Results from euglycaemic-hyperinsulinaemic clamps indicate reduced sensitivity to the biological effects of insulin predominantly in white adipose tissue of BN.GK-Nidd/gk1 congenics and a transient decrease in glucose uptake from pancreatic tissue. Impaired insulin stimulated glucose uptake and utilization in adipocytes of congenic rats contributes to hypertriglyceridemia and hyperglycaemia, which in turn stimulates insulin secretion. The effect of reduced pancreatic glucose uptake on islet function of congenics remains to be further documented. In old congenic rats, hypertriglyceridemia was persistent and associated with significantly reduced plasma HDL cholesterol level, which indicates that the phenotype deteriorates with age towards the emergence of risk factors for coronary heart disease [2]. Further analyses of features of the cardio-metabolic syndrome showed that adiposity, body weight and heart rate were increased in BN.GK-Nidd/gk1 congenics. Focal glomerulosclerosis and basal membrane thickening, which are important features of diabetes complications in humans, provide structural evidence of renal dysfunction in congenics.

BN.GK-Nidd/gk1 congenics, as CSS, are powerful tools for profiling the phenotypic effects of a large number of gene variants, which collectively are involved either directly or indirectly in multiple pathophysiological endpoints. Among these, known GK functional variants in the genes encoding the insulin degrading enzyme (IDE) [27], a G-protein receptor GPR10 [15] and an inositol -phosphatase (SHIP2) [28] are present in these congenics rats, and should have a significant impact on adiposity, hyperinsulinaemia and insulin resistance in the congenics. However, the discrimination between primary causes and secondary effects of one or several genetic variants is difficult. For example glomerulosclerosis in congenic rats may either develop as a consequence of permanent hyperglycaemia and hyperinsulinaemia or originate from the effect of specific GK variants.

The emergence of genotype data from large collections of rat SNPs in multiple inbred strains (http://www.well.ox.ac.uk/rat_mapping_resources/SNPbased_maps.html) provides important tools for defining haplotype blocks and predicting the localization of genes underlying altered phenotypes in BN.GK-Nidd/gk1 congenics. With respect to the etiology of diabetes in the GK rat, comparing SNP genotypes between Wistar related strains (WKY, SHR, SHRSP) and BN.GK-Nidd/gk1 rats in the congenic interval could identify naturally occurring variants originally selected in the GK strain for their causative role in glucose intolerance. Providing that diabetes in the GK stems from the selective isolation of diabetes susceptibility alleles present in an outbred colony of Wistar rats, genes involved in altered phenotypes in the BN.GK-Nidd/gk1 congenics may map to RNO1 regions that are genetically different between GK rats and either hypertensive SHR/SHRSP or WKY controls. It is also possible that these genetically related disease strains share common etiological variants isolated following generations of breedings of Wistar rats exhibiting glucose intolerance (GK) or high blood pressure (SHR). The presence of a functional mutation in SHIP2 in both SHR and GK rats [28], and its localisation in a short congenic interval associated with hypertension in a SHR.WKY strain combination [28], supports this hypothesis. In addition a functional mutation in Cd36 in the SHR relates to insulin sensitivity in adipocytes rather than directly to blood pressure [29]. Increased density of SNPs in the congenic region of the BN.GK-Nidd/gk1 strain is needed to test these hypotheses.

Gene expression changes in kidney and insulin sensitive organs of BN.GK-Nidd/gk1 rats are molecular adaptations that underlie causes and consequences of phenotypic features exhibited by this strain. Remarkably about 50% of genes showing statistically significant differential expression between congenics and BN map to the GK genomic interval of the congenics, even though this region of genetic divergence between strains only represents about 5% of the total rat genome length. These effects may indicate gene expression regulation in *cis*, whereas differentially expressed genes that map outside the congenic interval undoubtedly reflect effects in *trans* and are secondary adaptations to defects caused directly of indirectly by genes in the congenic region of GK origin. Providing that current rat SNP data reliably reflect haplotype structure, integration of data from SNP genotypes and transcriptomes in BN.GK-Nidd/gk1 and BN supports *cis*-regulation of transcription when differentially expressed genes map to congenic regions of haplotype divergence between BN and GK (eg. *Mesdc2*, *Nox4* in RNO1 region 129–144 Mb). Conversely, a number of genes differentially expressed fall in regions of apparent haplotype conservation between BN and GK (eg. *LOC292861*, *Dbp*, *Sult2b1* in RNO1 region 94–100 Mb), suggesting *trans*-regulation of transcription. Increasing SNP density should resolve fragmentation of the haplotype structure in congenic regions of BN.GK-Nidd/gk1 and ascertain segments of genetic divergence and conservation between strains, thus providing information for selecting functional and positional candidate genes.

In conclusion, we have established the pathophysiological and genome-wide gene expression signatures of QTLs linked to diabetes and obesity in a congenic strain, and developed SNP and haplotype maps that can assist disease gene discovery. This congenic strain provides a novel and sustainable model for investigating the genetic basis of risks factors for the cardio-metabolic syndrome and characterizing further associated phenotypes.

## Supporting Information

Table S1 (1.61 MB PDF)

Table S2 (0.11 MB PDF)
