# Supplementary material for: Pathophysiological, Genetic and Gene Expression Features of a Novel Rodent Model of the Cardio-Metabolic Syndrome
Source: PLoS One. 2008 Aug 13;3(8):e2962. doi: 10.1371/journal.pone.0002962 (PMC2500170; doi:10.1371/journal.pone.0002962)
Supplement: Table S1 — (1.61 MB PDF) [file pone.0002962.s001.pdf]

Table S1. SNP-based genotypes in colonies of rats of the Brown-Norway (BN), Goto-Kakizaki (GK), Wistar Kyoto (WKY), Spontaneously hypertensive (SHR), SHR stroke-prone (SHRSP) strains in the region of rat chromosome 1 of GK origin in BN.GK-Nidd/gk1 congenic rats. As reported in Fig.8 alleles shared between the BN and other strains are highlighted in yellow and alleles different to the BN are shown in blue. Heterozygous loci are shown in pink. SNP positions (b) in chromosome 1 are reported. SNP marker details are available through http://www.well.ox.ac.uk/rat_mapping_resources/SNPbased_maps.html.

			STRAINS																					
SNP_Ensembl_ID	SNP_External_ID	Position (b)	BN/Par	WKY/Izm	WKY/NCrl	WKY/Ztm	WKY/Bbb	WKYO/Kyo	WKY/Gla, WKY/Gcrc	WKY/NMna	SHR/Izm	SHR/NCrl	SHR/OlaIpcv	SHR/molBbb	SHR/Kyo	SHR/NHsd	SHRSP/Izm	SHRSP/Bbb	SHRSP/Ezo	SHRSP/Ngsk	SHRSP/Gla, SHRSP/Gcrc	GK/Slc	GK/Ox	BN.GK-Nidd/gk1/Ox
ENSRNOSNP2783757	J336026	89665243	C	C	T	T	T	C	T	T	T	T	T	T	T	T	T	T	T	T	-	T	T	T
ENSRNOSNP2783758	WKY-G-j-45h11_f1_383	89798155	T	C	C	C	C	C	C	C	C	C	C	C	C	C	C	C	C	C	C	T	T	T
ENSRNOSNP2783759	gnl|ti|842490884_50710952_687	90431156	T	C	T	T	T	T	T	T	C	C	C	C	C	C	C	C	C	C	C	T	T	T
ENSRNOSNP2783760	J596244	90735234	C	C	C	C	C	C	C	C	C	C	C	C	C	C	C	C	C	C	-	G	G	G
ENSRNOSNP2783761	Cpn_1090749408	90749408	C	C	C	C	C	C	C	C	C	C	C	C	C	C	C	C	C	C	C	T	T	T
ENSRNOSNP2783762	gko-23f24_fp2_b1_431	91081446	C	C	C	C	C	C	C	C	C	C	C	C	C	C	C	C	C	C	C	G	G	G
ENSRNOSNP2783763	J546795	91171603	G	G	G	G	G	G	G	G	G	G	G	G	G	G	G	G	G	G	-	T	T	T
ENSRNOSNP2783764	J533325	91371118	G	G	G	G	G	G	G	G	G	G	G	G	G	G	G	G	G	G	-	A	A	A
ENSRNOSNP2783765	J341779	91702897	G	A	A	A	A	A	A	A	A	A	A	A	A	A	A	A	A	A	A	A	A	A
ENSRNOSNP2783766	WKYc18e09_s1_438	91834851	C	T	T	T	T	T	T	T	T	T	T	T	T	T	T	T	T	T	T	T	T	T
ENSRNOSNP2783767	J495136	91857887	C	T	T	T	T	T	T	T	T	T	T	T	T	T	T	T	T	T	-	T	T	T
ENSRNOSNP2783768	J873803	92142332	G	A	A	A	A	A	A	A	A	A	A	A	A	A	A	A	A	A	A	A	A	A
ENSRNOSNP2783769	rat101_005_o02.p1ca_204	92276533	A	G	G	G	G	G	G	G	G	G	G	G	G	G	G	G	G	G	G	G	G	G
ENSRNOSNP2783770	J642278	92287946	G	A	A	A	A	A	A	A	A	A	A	A	A	A	A	A	A	A	-	A	A	A
ENSRNOSNP2783771	WKYc95a06_s1_703	92339833	G	-	A	A	A	A	A	A	A	A	A	-	A	A	A	A	A	A	A	A	A	A
ENSRNOSNP2783772	WKYc02d01_r1_652	92476744	C	T	T	T	T	T	T	T	T	T	T	T	T	T	T	T	T	T	T	T	T	T
ENSRNOSNP2783773	J1261396	92714418	A	T	T	-	T	T	-	T	T	T	T	T	T	-	T	T	T	T	-	T	T	T
ENSRNOSNP2783774	gko-66f21_fp2_b1_272	92870190	G	-	A	A	A	A	A	A	A	A	-	A	A	-	A	A	A	A	A	A	A	A
ENSRNOSNP2783775	gko-104e7_rp2_b1_177	93597048	C	T	T	T	T	T	T	T	T	T	T	T	T	T	T	T	T	T	T	T	T	T
ENSRNOSNP2783776	WKYOa73f10_s1_456	93760176	A	G	G	G	G	G	G	G	G	G	G	G	G	G	G	G	G	G	G	G	G	G
ENSRNOSNP2783777	J1288901	93917072	A	C	C	C	C	C	A	C	A	A	A	A	A	A	A	A	A	A	A	A	A	A
ENSRNOSNP2783779	WKYc110g02_r1_646	94624428	G	A	A	-	A	A	G	A	G	G	G	G	G	G	G	G	G	G	G	G	G	G
ENSRNOSNP2783780	rdahl-25i13_rp2_b1_125	95200769	C	C	C	C	C	C	C	C	C	C	C	C	C	C	C	C	C	C	C	C	C	C
ENSRNOSNP2783782	J640192	95446362	G	G	G	G	G	G	G	G	G	G	G	G	G	G	G	G	G	G	-	G	G	G
ENSRNOSNP2783783	J876484	95622713	G	G	G	G	G	G	G	G	G	G	G	G	G	G	G	G	G	G	G	G	G	G
ENSRNOSNP2783786	J687602	96557183	G	G<br>T	G	-	G	G<br>T	-	G<br>T	-	G	G	G	-	-	-	G	G<br>T	G<br>T	-	G<br>T	G	G
ENSRNOSNP2783788	rat108_009_h10.q1cc_225	97225611	T	T	T	T	T	T	T	T	C	T	T	T	C	T	C	C	C	C	C	T	T	T

ENSRNOSNP2783789	J642006	97296856	A	A	A	A	A	A	A	A	C	A	A	A	C	A	C	C	C	C	-	A	A	A
ENSRNOSNP2783790	gnl|ti|896470776_19866867247114_269	97461389	C	C	C	C	C	C	C	C	T	C	C	C	T	C	T	T	T	T	T	C	C	C
ENSRNOSNP2783791	rat013_050_e15.p1ca_424	97608515	A	A	A	A	A	A	A	A	C	A	A	A	C	A	C	C	C	C	C	A	A	A
ENSRNOSNP2783792	rat108_018_c12.q1ca_362	97662654	C	C	C	C	C	C	C	C	T	C	C	C	T	C	T	T	T	T	T	C	C	C
ENSRNOSNP2783793	J679019	97675195	G	G	G	G	G	G	G	G	A	G	G	G	A	G	A	A	A	A	A	G	G	G
ENSRNOSNP2783794	rdahl-68l13_fp2_b1_28	97991504	A	A	A	A	A	A	A	A	G	A	A	A	G	A	G	G	G	G	G	A	A	A
ENSRNOSNP2783795	J342286	98149363	C	C	C	C	C	C	C	C	T	C	C	C	T	C	T	T	T	T	-	C	C	C
ENSRNOSNP2783796	gnl|ti|896531661_19866867344453_284	98241710	G	G	G	G	G	G	G	G	T	G	G	G	T	G	T	T	T	T	T	G	G	G
ENSRNOSNP2783797	gnl|ti|896508801_19866866706148_273	98576405	C	C	C	C	C	C	C	C	T	C	C	C	T	C	T	T	T	T	T	C	C	C
ENSRNOSNP2783798	J874515	98875538	G	G	G	G	G	G	G	G	G	G	G	G	G	G	G	G	G	G	G	G	G	G
ENSRNOSNP2783799	DS-g-a-42b06_f1_990	98935831	G	G	G	G	G	G	G	G	G	G	G	G	G	G	G	G	G	G	G	G	G	G
ENSRNOSNP2783800	J654366	99077826	T	T	T	T	T	T	T	T	T	T	T	T	T	T	T	T	T	T	-	T	T	T
ENSRNOSNP2783801	rdahl-45n15_rp2_b1_560	99079170	G	G	G	G	G	G	G	G	G	G	G	G	G	G	G	G	G	G	G	G	G	G
ENSRNOSNP2783802	gnl|ti|896499814_19866868290849_349	99221185	A	A	A	A	A	A	A	A	A	A	A	A	A	A	A	A	A	A	A	A	A	A
ENSRNOSNP2783803	J658249	99267550	C	C	C	C	C	C	C	C	C	C	C	C	C	C	C	C	C	C	C	C	C	C
ENSRNOSNP2783804	rdahl-64k18_fp2_b1_474	99365916	C	C	C	C	C	C	C	C	C	C	C	C	C	C	C	C	C	C	C	C	C	C
ENSRNOSNP2783805	rat013_031_o01.p1ca_633	99577305	T	T	T	T	T	T	T	T	T	T	T	T	T	T	T	T	T	T	T	T	T	T
ENSRNOSNP2783806	J681651	99612906	G	G	G	G	G	G	G	G	G	G	G	G	G	G	G	G	G	G	-	G	G	G
ENSRNOSNP2783807	DahlSa02d01_s1_31	99624064	G	G	G	G	G	G	G	G	G	G	G	G	G	G	G	G	G	G	G	G	G	G
ENSRNOSNP2783808	J650153	99736245	C	C	C	C	C	C	C	C	C	C	C	C	C	C	C	C	C	C	C	C	C	C
ENSRNOSNP2783809	J678979	99951882	G	G	G	G	G	G	G	G	G	G	G	G	G	G	G	G	G	G	-	G	G	G
ENSRNOSNP2783810	rdahl-67p16_fp2_b1_463	100013618	T	T	T	T	T	T	T	T	T	T	T	T	T	T	T	T	T	T	T	T	T	T
ENSRNOSNP2783811	rdahl-63m10_rp2_b1_51	100156400	T	C	C	C	C	C	C	C	C	C	C	C	C	C	C	C	C	C	C	C	C	C
ENSRNOSNP2783812	J496447	100823084	C	C	C	C	C	C	C	C	C	C	C	C	C	C	C	C	C	C	-	A	A	A
ENSRNOSNP2783813	SHRSPc51d07_r1_117	100829033	C	C	C	C	C	C	C	C	T	C	C	C	T	C	T	T	T	T	T	C	C	C
ENSRNOSNP2783814	J343473	100941911	C	C	C	C	C	C	C	C	T	C	C	C	T	C	T	T	T	T	-	T	T	T
ENSRNOSNP2783815	J880077	101108398	A	G	G	G	G	G	G	G	A	G	G	G	A	G	A	A	A	A	A	A	A	A
ENSRNOSNP2783816	gko-99b15_rp2_b1_235	101121890	T	C	C	C	C	C	C	C	C	C	C	C	C	C	C	C	C	C	C	C	C	C
ENSRNOSNP2783817	gnl|ti|896710451_19866866704029_303	101271083	A	G	G	G	G	G	G	G	G	G	G	G	G	G	G	G	G	G	G	G	G	G
ENSRNOSNP2783818	J506070	101444028	T	C	C	C	C	C	C	C	C	C	C	C	C	C	C	C	C	C	C	C	C	C
ENSRNOSNP2783819	WKYc11a03_r1_380	101536771	A	G	G	G	G	G	G	G	G	G	G	G	G	G	G	G	G	G	G	A	A	A
ENSRNOSNP2783820	J663971	101751943	T	TC	C	-	C	TC	-	TC	T	C	C	C	T	-	T	T	T	T	-	T	T	T
ENSRNOSNP2783821	rdahl-59h15_rp2_b1_356	101758157	C	A	A	A	A	A	A	A	C	A	A	A	C	A	C	C	C	C	C	C	C	C
ENSRNOSNP2783822	rat107_001_h03.p1ma_391	101802313	G	A	A	A	A	A	A	A	G	A	A	A	G	A	G	G	G	G	G	G	G	G
ENSRNOSNP2783823	J686646	101975517	A	G	G	G	G	G	G	G	G	G	G	G	G	G	G	G	G	G	-	A	A	A
ENSRNOSNP2783824	SHRSPa02d01_r1_497	101994486	T	C	C	C	C	C	C	C	C	C	C	C	C	C	C	C	C	C	C	T	T	T
ENSRNOSNP2783825	J481026	102251894	G	A	A	A	A	A	A	A	A	A	A	A	A	A	A	A	A	A	A	A	A	A
ENSRNOSNP2783826	J506657	102366482	T	G	G	G	G	G	G	G	G	G	G	G	G	G	G	G	G	G	-	G	G	G
ENSRNOSNP2783827	WKY-G-f-09f04_r1_462	102403222	A	C	C	C	C	C	C	C	C	C	C	C	C	C	C	C	C	C	C	C	C	C

ENSRNOSNP2783828	rdahl-98p3_rp2_b1_101	102554553	G	C	C	C	C	C	C	C	C	C	C	C	C	C	C	C	C	C	C	G	G	G
ENSRNOSNP2783829	WKYc46c06_r1_321	102593648	A	G	G	G	G	G	G	G	-	G	G	G	G	G	G	G	G	G	G	G	G	G
ENSRNOSNP2783830	J566352	102621505	C	G	G	G	G	G	G	G	G	G	G	G	G	G	G	G	G	G	G	G	G	G
ENSRNOSNP2783831	SHRSPa52g08_r1_790	102733546	G	-	A	A	A	A	A	A	A	-	-	A	A	G<br>A	A	A	A	A	A	G	G	G
ENSRNOSNP2783832	WKYOa21g08_r1_310	102827347	T	A	A	A	A	A	A	A	A	A	A	A	A	A	A	A	A	A	A	T	T	T
ENSRNOSNP2783833	J342756	102981652	C	A	A	-	A	A	-	A	A	A	A	A	A	-	A	A	A	A	-	C<br>A	C	C<br>A
ENSRNOSNP2783834	J533930	103254896	G	G	G	G	G	G	G	G	G	G	G	G	G	G	G	G	G	G	-	A	A	A
ENSRNOSNP2783835	gko-39j14_fp2_b1_736	103492999	C	C	C	C	C	C	C	C	C	C	C	C	C	C	C	C	C	C	C	T	-	T
ENSRNOSNP2783836	J881888	103587186	A	G	G	G	G	G	G	G	G	G	G	G	G	G	G	G	G	G	-	G	G	G
ENSRNOSNP2783837	J341638	103785594	C	C	C	C	C	C	C	C	C	C	C	C	C	C	C	C	C	C	C	C	C	C
ENSRNOSNP2783838	gko-7f3_fp2_b1_92	103819342	G	G	G	G	G	G	G	G	G	G	G	G	G	G	G	G	G	G	G	T	T	T
ENSRNOSNP2783839	J881006	103886653	T	T	T	T	T	T	T	T	T	T	T	T	T	T	T	T	T	T	-	T	T	T
ENSRNOSNP2783840	J521319	103992901	G	A	A	A	A	A	A	A	A	A	A	A	A	A	A	A	A	A	-	A	A	A
ENSRNOSNP2783841	SHRSPc65h10_s2_27	104042978	A	G	G	G	G	G	G	G	G	G	G	G	G	G	G	G	G	G	G	G	G	G
ENSRNOSNP2783842	J493818	104094080	T	C	C	-	C	C	-	C	C	C	C	C	C	-	C	C	C	C	-	C	C	C
ENSRNOSNP2783843	gnl|ti|897031259_19866867996652_370	104257021	T	T	T	T	T	T	T	T	T	T	T	T	T	T	T	T	T	T	T	T	T	T
ENSRNOSNP2783844	J875937	104391455	G	G	G	G	G	G	G	G	G	G	G	G	G	G	G	G	G	G	-	G	G	G
ENSRNOSNP2783845	gko-92k2_rp2_b1_79	104676073	T	C	C	C	-	C	C	C	C	C	C	C	C	C	C	C	C	C	C	C	C	C
ENSRNOSNP2783846	J484430	104789176	G	C	C	C	C	C	C	C	C	C	C	C	C	C	C	C	C	C	-	C	C	C
ENSRNOSNP2783847	gko-88g2_fp2_b1_461	104847294	T	T	T	T	T	T	T	T	T	T	T	T	T	T	T	T	T	T	T	C	C	C
ENSRNOSNP2783848	gko-56e2_fp2_b1_282	105190514	T	T	T	T	T	T	T	T	T	T	T	T	T	T	T	T	T	T	T	C	C	C
ENSRNOSNP2783849	gko-56i13_rp2_b1_197	105434384	C	-	T	T	T	T	T	T	T	T	T	T	T	T	T	-	T	T	T	T	T	T
ENSRNOSNP2783850	J480970	105823532	A	-	C	-	C	-	-	-	-	C	C	C	C	-	C	C	-	-	-	-	C	C
ENSRNOSNP2783851	rdahl-28e21_fp2_b1_171	105892733	C	T	T	T	T	T	T	T	T	T	T	T	T	T	T	T	T	T	T	C	C	C
ENSRNOSNP2783852	J640184	106299428	A	G	G	G	G	G	G	G	G	G	G	G	G	G	G	G	G	G	-	A	A	A
ENSRNOSNP2783853	gko-108m5_rp2_b1_91	106509849	T	C	C	C	C	C	C	C	C	C	C	C	C	C	C	C	C	C	C	C	C	C
ENSRNOSNP2783854	J521284	106857283	A	G	G	G	G	G	G	G	G	G	G	G	G	G	G	G	G	G	G	G	G	G
ENSRNOSNP2783855	J693429	107043695	T	A	A	A	A	A	A	A	A	A	A	A	A	A	A	A	A	A	-	T	T	T
ENSRNOSNP2783856	rdahl-80o1_fp2_b1_117	107043987	G	G	G	G	G	G	G	G	G	G	G	G	G	G	G	G	G	G	G	G	G	G
ENSRNOSNP2783857	gko-81g9_fp2_b1_287	107214766	T	C	C	C	C	C	C	C	C	C	C	C	C	C	C	C	C	C	C	C	C	C
ENSRNOSNP2783858	J566904	107332219	G	G	G	G	G	G	G	G	G	G	G	G	G	G	G	G	G	G	-	A	A	A
ENSRNOSNP2783859	gko-86e18_fp2_b1_170	107444560	G	T	T	T	T	T	T	T	T	T	T	T	T	T	T	T	T	T	T	T	T	T
ENSRNOSNP2783860	J536828	107522326	A	G	G	G	G	G	G	G	G	G	G	G	G	G	G	G	G	G	G	G	G	G
ENSRNOSNP2783861	gko-52j13_fp2_b1_243	107618861	T	C	C	C	C	C	C	C	C	C	C	C	C	C	C	C	C	C	C	C	C	C
ENSRNOSNP2783862	rdahl-60o14_rp2_b1_386	107870422	C	C	C	C	C	C	C	C	C	C	C	C	C	C	C	C	C	C	C	A	A	A
ENSRNOSNP2783863	J649909	107907998	T	TC	C	-	C	TC	-	TC	TC	C	C	C	TC	-	TC	C	TC	TC	-	TC	C	C
ENSRNOSNP2783864	gko-79g17_fp2_b1_79	108078865	C	C	C	C	C	C	C	C	C	C	C	C	C	C	C	C	C	C	C	T	T	T
ENSRNOSNP2783865	rat105_027_f23.q1ca_552	108175970	T	G	G	G	G	G	G	G	G	G	G	G	G	G	G	G	G	G	G	G	G	G
ENSRNOSNP2783866	J636671	108224075	C	G	G	G	G	G	G	G	G	G	G	G	G	G	G	G	G	G	G	G	G	G

ENSRNOSNP2783867	J528758	108380113	T	C	C	C	C	C	C	C	C	C	C	C	C	C	C	C	C	C	-	C	C	C
ENSRNOSNP2783868	SHRSPc37d09_r1_466	108494105	T	A	A	A	A	A	A	A	A	A	A	A	A	A	A	A	A	A	A	A	A	A
ENSRNOSNP2783869	J585688	108602110	T	C	C	C	C	C	C	C	C	C	C	C	C	C	C	C	C	C	C	C	C	C
ENSRNOSNP2783870	SHRSPa25h12_s1_314	108676156	C	A	A	A	A	A	A	A	A	A	A	A	A	A	A	A	A	A	A	C	C	C
ENSRNOSNP2783871	Cpn_1108843270	108843270	G	A	A	A	A	A	A	A	A	A	A	A	A	A	A	A	A	A	A	A	A	A
ENSRNOSNP2783872	J679516	108918674	T	T	T	T	T	T	T	T	T	T	T	T	T	T	T	T	T	T	T	C	C	C
ENSRNOSNP2783873	rat101_023_p05.q1ca_269	109063711	C	T	T	T	T	T	T	T	T	T	T	T	T	T	T	T	T	T	T	T	T	T
ENSRNOSNP2783874	Cpn_1109198245	109198245	C	C	C	C	C	C	C	C	C	C	C	C	C	C	C	C	C	C	C	C	C	C
ENSRNOSNP2783875	J677386	109245150	C	CT	C	C	C	-	C	C	-	C	C	C	C	C	C	C	-	C	C	CT	C	C
ENSRNOSNP2783876	rat108_008_n24.q1cb_603	109278332	C	T	T	T	T	T	T	T	T	T	T	T	T	T	T	T	T	T	T	T	T	T
ENSRNOSNP2783877	SHRSPa11g12_r1_990	109359529	C	T	T	T	T	T	T	T	T	T	T	T	T	T	T	T	T	T	T	T	T	T
ENSRNOSNP2783878	J488679	109447433	A	G	G	G	G	G	G	G	G	G	G	G	G	G	G	G	G	G	-	G	G	G
ENSRNOSNP2783879	WKYOa54c08_r1_684	109668244	G	A	A	A	A	A	A	A	A	A	A	A	A	A	A	A	A	A	A	A	A	A
ENSRNOSNP2783880	J1269812	109731284	A	G	G	G	G	G	G	G	G	G	G	G	G	G	G	G	G	G	-	G	G	G
ENSRNOSNP2783881	gnl|ti|896442060_19866867372388_361	109807875	C	C	C	C	C	C	C	C	C	C	C	C	C	C	C	C	C	C	C	C	C	C
ENSRNOSNP2783882	J501027	109915859	A	G	G	G	G	G	G	G	G	G	G	G	G	G	G	G	G	G	G	G	G	G
ENSRNOSNP2783883	WKYOa49g06_s1_555	110009216	C	T	T	T	T	T	T	T	T	T	T	T	T	T	T	T	T	T	T	T	T	T
ENSRNOSNP2783884	J694247	110013101	C	C	C	C	C	C	C	C	C	C	C	C	C	C	C	C	C	C	-	C	C	C
ENSRNOSNP2783885	J699038	110144828	A	A	A	A	A	A	A	A	A	A	A	A	A	A	A	A	A	A	-	A	A	A
ENSRNOSNP2783886	gnl|ti|896708706_19866867053543_317	110172143	C	C	C	C	C	C	C	C	C	C	C	C	C	C	C	C	C	C	C	C	C	C
ENSRNOSNP2783887	J1273406	110408930	C	A	A	A	A	A	A	A	A	A	A	A	A	A	A	A	A	A	-	A	A	A
ENSRNOSNP2783888	gnl|ti|896621983_19866867213485_406	110667615	T	T	T	T	T	T	T	T	T	T	T	T	T	T	T	T	T	T	T	T	T	T
ENSRNOSNP2783889	Cpn_1110839021	110839021	T	C	C	C	C	C	C	C	C	C	C	C	C	-	C	C	C	-	C	C	C	C
ENSRNOSNP2783890	J650619	110848285	C	T	T	T	T	T	T	T	T	T	T	T	T	T	T	T	T	T	-	T	T	T
ENSRNOSNP2783891	J574387	111314373	T	C	C	C	C	C	C	C	C	C	C	C	C	C	C	C	C	C	-	C	C	C
ENSRNOSNP2783893	gko-41g7_rp2_b1_354	111576360	G	C	C	C	C	C	C	C	C	C	C	C	C	C	C	C	C	C	C	C	C	C
ENSRNOSNP2783894	gko-51k9_fp2_b1_130	111781206	G	T	T	T	T	T	T	T	T	T	T	T	T	T	T	T	T	T	T	T	T	T
ENSRNOSNP2783895	rdahl-84b9_rp2_b1_347	111943004	T	T	T	T	T	T	T	T	T	T	T	T	T	T	T	T	T	T	T	T	T	T
ENSRNOSNP2783896	SHRSPa26a07_r1_494	112933199	C	A	A	A	A	A	A	A	A	A	A	A	A	A	A	A	A	A	A	A	A	A
ENSRNOSNP2783897	J547385	113002849	C	A	A	A	A	A	A	A	A	A	A	A	A	A	A	A	A	A	-	A	A	A
ENSRNOSNP2783898	gnl|ti|896912254_19866867420098_303	113140297	A	C	C	C	C	C	C	C	C	C	C	C	C	C	C	C	C	C	C	C	C	C
ENSRNOSNP2783899	J553138	113195993	C	T	T	T	T	T	T	T	T	T	T	T	T	T	T	T	T	T	-	T	T	T
ENSRNOSNP2783900	rdahl-79o2_fp2_b1_439	113368206	A	A	A	A	A	A	A	A	A	A	A	A	A	A	A	A	A	A	A	A	A	A
ENSRNOSNP2783901	J690554	114173922	C	T	T	T	T	T	T	T	T	T	T	CT	T	T	T	T	T	T	T	T	T	T
ENSRNOSNP2783902	DS-g-a-35f05_r1_120	114445860	A	A	A	A	-	A	A	A	A	A	A	A	A	A	A	A	A	A	A	A	A	A
ENSRNOSNP2783903	J521303	114575487	T	C	C	C	C	C	C	C	C	C	C	C	C	C	C	C	C	C	C	C	C	C
ENSRNOSNP2783904	SHRSPa9b06_s1_658	114584829	T	C	C	C	C	C	C	C	C	C	C	C	C	C	C	C	C	C	C	C	C	C
ENSRNOSNP2783905	J1280769	114796688	T	G	G	G	G	G	G	G	G	G	G	G	G	G	G	G	G	G	-	G	G	G
ENSRNOSNP2783906	rdahl-33g22_fp2_b1_437	114808364	T	G	G	G	G	G	G	G	G	G	G	G	G	G	G	G	G	G	G	G	G	G

ENSRNOSNP2783907	J527933	114886455	TC	C	C	-	C	C	-	C	C	C	C	C	C	-	C	C	C	C	-	C	C	C
ENSRNOSNP2783908	J585062	115008622	T	A	A	A	A	A	A	A	A	A	A	A	A	A	A	A	A	A	-	A	A	A
ENSRNOSNP2783909	SHRSPc44g10_s1_445	115118000	A	T	T	T	T	T	T	T	T	T	T	T	T	T	T	T	T	T	T	T	T	T
ENSRNOSNP2783910	J585159	115250265	T	C	C	C	C	C	C	C	C	C	C	C	C	C	C	C	C	C	C	C	C	C
ENSRNOSNP2783911	gko-43e21_fp2_b1_374	115310946	G	T	T	T	T	T	T	T	T	T	T	T	T	T	T	T	T	T	T	T	T	T
ENSRNOSNP2783912	J686927	115356045	G	G	G	G	G	G	G	G	G	G	G	G	G	G	G	G	G	G	-	G	G	G
ENSRNOSNP2783913	J548749	115457596	A	G	G	G	G	G	G	G	G	G	G	G	G	G	G	G	G	G	-	G	G	G
ENSRNOSNP2783914	gnl|ti|897040500_19866868466485_296	115599352	A	A	A	A	A	A	A	A	A	A	A	A	A	A	A	A	A	A	A	A	A	A
ENSRNOSNP2783915	J586421	115670190	C	T	T	-	T	T	-	T	T	T	T	T	T	-	T	T	T	T	-	T	T	T
ENSRNOSNP2783916	J338375	115880875	T	C	C	-	C	C	-	C	C	C	C	C	C	-	C	C	C	C	-	C	C	C
ENSRNOSNP2783917	SHRSPa18e07_s1_607	116043861	A	G	G	G	G	G	G	G	G	G	G	G	G	G	G	G	G	G	G	G	G	G
ENSRNOSNP2783918	SHRSPa36c11_r1_1122	116209253	A	G	G	G	G	G	G	G	G	G	G	G	G	G	G	G	G	G	G	G	G	G
ENSRNOSNP2783919	gko-50i18_rp2_b1_366	116402327	T	C	C	C	C	C	C	C	C	C	C	C	C	C	C	C	C	C	C	C	C	C
ENSRNOSNP2783920	gko-24o6_fp2_b1_546	116584743	G	A	A	A	A	A	-	A	A	A	A	-	A	A	A	A	A	A	A	A	A	A
ENSRNOSNP2783921	J664703	116637220	A	A<br>G	G	-	G	A<br>G	-	A<br>G	A<br>G	G	G	G	-	-	-	G	A<br>G	-	-	A<br>G	G	G
ENSRNOSNP2783922	J880979	116986999	A	C	C	C	C	C	C	C	C	C	C	C	C	C	C	C	C	C	C	C	C	C
ENSRNOSNP2783923	J492287	117169835	T	C	C	C	C	C	C	C	C	C	C	C	C	C	C	C	C	C	C	C	C	C
ENSRNOSNP2783924	J341953	117309911	C<br>G	G	G	-	G	G	-	G	G	G	G	G	G	-	G	G	G	G	-	G	G	G
ENSRNOSNP2783925	rdahl-83j18_fp2_b1_380	117415434	G	A	A	A	A	A	A	A	A	A	A	A	A	A	A	A	A	A	A	A	A	A
ENSRNOSNP2783926	gko-8g5_fp2_b1_290	117586011	T	C	C	C	C	C	C	C	C	C	C	C	C	C	C	C	C	C	C	C	C	C
ENSRNOSNP2783927	J675221	117627096	T	C	C	C	C	C	C	C	C	C	C	C	C	C	C	C	C	C	C	C	C	C
ENSRNOSNP2783928	SHRSPc30e11_r1_168	117736961	C	T	T	T	T	T	T	T	T	T	T	T	T	T	T	T	T	T	T	T	T	T
ENSRNOSNP2783929	J575682	117953954	T	C	C	C	C	C	C	C	C	C	C	C	C	C	C	C	C	C	C	C	C	C
ENSRNOSNP2783930	DS-g-c-13g02_f1_263	117988593	T	C	C	C	C	C	C	C	C	C	C	C	C	C	C	C	C	C	C	C	C	C
ENSRNOSNP2783931	WKY-G-j-16a06_f1_394	118167567	T	C	C	C	C	C	C	C	C	C	C	C	C	C	C	C	C	C	C	C	C	C
ENSRNOSNP2783932	J649238	118261965	T	T	T	T	T	T	T	T	T	T	T	T	T	T	T	T	T	T	T	T	T	T
ENSRNOSNP2783933	rdahl-51g21_fp2_b1_251	118330903	C	T	T	T	T	T	T	T	T	T	T	T	T	T	T	T	T	T	T	T	T	T
ENSRNOSNP2783934	gnl|ti|896478825_19866866843283_266	118493283	C	C	C	C	C	C	C	C	C	C	C	C	C	C	C	C	C	C	-	C	C	C
ENSRNOSNP2783935	J569388	118535742	C	A	C	C	C	C	A	C	A	A	A	A	A	A	A	A	A	A	-	A	A	A
ENSRNOSNP2783936	J560146	118746855	G	C	C	C	C	C	C	C	C	C	C	C	C	C	C	C	C	C	C	C	C	C
ENSRNOSNP2783937	rat104_006_b20.q1ca_249	118784190	A	A	G	G	G	G	A	G	A	A	A	A	A	A	A	A	A	A	A	A	A	A
ENSRNOSNP2783938	Cpn_1118784590	118784590	A	G	G	G	G	G	G	G	G	G	G	G	G	G	G	G	G	G	G	G	G	G
ENSRNOSNP2783939	rat013_045_o14.p1ca_158	118842908	C	C	G	G	G	G	C	G	C	C	C	C	C	C	C	C	C	C	C	C	C	C
ENSRNOSNP2783940	rat109_061_b02.q1ca_253	118843159	T	C	C	C	C	C	C	C	C	C	C	C	C	C	C	C	C	C	C	C	C	C
ENSRNOSNP2783941	J476188	118886978	G	A	G	G	G	G	A	G	A	A	A	A	A	A	A	A	A	A	-	A	A	A
ENSRNOSNP2783942	SHRSPa57c08_r1_491	118971412	A	G	G	G	G	G	G	G	G	G	G	G	G	G	G	G	G	G	G	G	G	G
ENSRNOSNP2783943	SHRSPa98e12_r1_926	119170766	C	T	T	T	C	C	T	T	T	T	T	T	T	T	T	T	T	T	T	T	T	T
ENSRNOSNP2783944	J1273405	119229935	A	A	A	A	G	G	A	A	A	A	A	A	A	A	A	A	A	A	-	A	A	A
ENSRNOSNP2783945	rat105_015_h21.p1ca_325	119276565	C	A	A	A	A	A	A	A	A	A	A	A	A	A	A	A	A	A	A	A	A	A

ENSRNOSNP2783946	SHRSPc42d12_s1_668	119280318	T	C	C	C	C	C	C	C	C	C	C	C	C	C	C	C	C	C	C	C	C	C
ENSRNOSNP2783947	WKYc98g04_s1_173	119377105	G	G	-	G	A	A	G	G	G	G	G	G	G	G	G	G	G	G	G	G	G	G
ENSRNOSNP2783948	J694582	119527694	A	A	A	A	T	T	A	A	A	A	A	A	A	A	A	A	A	A	A	A	A	A
ENSRNOSNP2783949	rdahl-82h21_rp2_b1_390	119527961	T	T	T	T	C	C	T	T	T	T	T	T	T	T	T	T	T	T	T	T	T	T
ENSRNOSNP2783950	J1299985	119919868	G	G	G	G	A	A	G	G	G	G	G	G	G	G	G	G	G	G	G	G	G	G
ENSRNOSNP2783951	WKYc60d07_r1_541	119928727	A	A	A	A	G	G	A	A	A	A	A	A	A	A	A	A	A	A	A	A	A	A
ENSRNOSNP2783952	J539410	120033400	T	C	C	C	T	T	C	C	C	C	C	C	C	C	C	C	C	C	-	C	C	C
ENSRNOSNP2783953	SHRSPa16f04_r1_935	120045401	T	C	C	C	T	T	C	C	C	C	C	C	C	C	C	C	C	C	C	C	C	C
ENSRNOSNP2783954	gko-62g17_rp2_b1_200	120204774	G	-	T	T	G	G	T	T	T	T	T	-	T	-	T	T	-	T	T	T	T	T
ENSRNOSNP2783955	SHRSPc15b07_r1_604	120467095	T	C	C	C	T	T	C	C	C	C	C	C	C	C	C	C	C	C	C	C	C	C
ENSRNOSNP2783956	J524177	120584397	A	T	T	T	A	A	T	T	T	T	T	T	T	T	T	T	T	T	-	T	T	T
ENSRNOSNP2783957	SHRSPc46d02_s1_42	120910968	G	C	C	C	G	G	C	C	C	C	C	C	C	C	C	C	C	C	C	C	C	C
ENSRNOSNP2783958	J561421	120921450	A	T	T	-	A	-	-	T	T	T	T	T	T	-	T	T	T	T	-	T	T	T
ENSRNOSNP2783959	gko-91c4_rp2_b1_727	121055070	T	C	C	C	T	T	C	C	C	C	C	C	C	C	C	C	C	C	C	C	C	C
ENSRNOSNP2783960	J589036	121242501	T	C	C	C	T	T	C	C	C	C	C	C	C	TC	C	C	C	C	C	C	C	C
ENSRNOSNP2783961	gko-77f10_fp2_b1_35	121494780	G	A	A	A	G	G	A	A	A	A	A	A	A	A	A	A	A	A	A	A	A	A
ENSRNOSNP2783963	WKYc106a08_r1_376	122215589	C	G	G	G	G	G	G	G	G	G	G	G	G	G	G	G	G	G	G	G	G	G
ENSRNOSNP2783964	SHRSPc30b10_s1_695	122262859	G	A	A	A	G	G	A	A	A	A	A	A	A	A	A	A	A	A	A	A	A	A
ENSRNOSNP2783965	WKYOa50d08_r1_327	122309689	T	C	C	C	C	C	C	C	C	C	C	C	C	C	C	C	C	C	C	C	C	C
ENSRNOSNP2783966	J495552	122393130	C	A	A	A	A	A	A	A	A	A	A	A	A	A	A	A	A	A	A	A	A	A
ENSRNOSNP2783967	rat101_002_l19.p1ca_223	122399011	G	A	A	A	A	A	A	A	A	A	A	A	A	A	A	A	A	A	A	A	A	A
ENSRNOSNP2783968	gnl|ti|842490727_50711248_276	122476269	A	-	A	A	G	G	G	A	G	G	G	G	G	G	G	G	G	G	G	A	A	A
ENSRNOSNP2783969	J536095	122627890	T	C	C	C	T	T	T	C	T	T	T	T	T	T	T	T	T	T	-	C	C	C
ENSRNOSNP2783970	SHRSPa25a01_s1_297	122657587	C	C	C	C	T	T	T	C	T	T	T	T	T	T	T	T	T	T	T	C	C	C
ENSRNOSNP2783971	J682284	122728576	A	A	A	-	G	A/G	-	A	A/G	G	G	G	A/G	-	A/G	G	A/G	A/G	-	A	A	A
ENSRNOSNP2783972	WKY-G-j-27a09_f1_564	122811661	T	T	T	T	G	G	G	T	G	G	G	G	G	G	G	G	G	G	G	T	T	T
ENSRNOSNP2783973	J506689	122843086	G	A	A	A	G	G	G	A	G	G	G	G	G	G	G	G	G	G	-	A	A	A
ENSRNOSNP2783974	WKYc66h11_r1_195	122900275	G	A	A	A	A	A	A	A	A	A	A	A	A	A	A	A	A	A	A	A	A	A
ENSRNOSNP2783975	J343874	122963154	C	C	C	C	T	T	T	C	T	T	T	T	T	T	T	T	T	T	-	C	C	C
ENSRNOSNP2783976	rat110_034_h15.q1ca_60	122992113	A	A	A	A	G	G	G	A	G	G	G	G	G	G	G	G	G	G	G	A	A	A
ENSRNOSNP2783977	SHRSPc56b12_s1_172	123053829	T	C	C	C	C	C	C	C	C	C	C	C	C	C	C	C	C	C	C	C	C	C
ENSRNOSNP2783978	rat105_014_o07.p1ca_225	123115506	A	G	G	G	G	G	G	G	G	G	G	G	G	G	G	G	G	G	G	G	G	G
ENSRNOSNP2783979	WKYc107d01_s1_182	123139842	T	C	C	C	C	C	C	C	C	C	C	C	C	C	C	C	C	C	C	C	C	C
ENSRNOSNP2783980	J697929	123235931	T	T	T	T	C	C	C	T	C	C	C	C	C	C	C	C	C	C	-	T	T	T
ENSRNOSNP2783981	J580501	123377800	C	T	T	T	C	C	C	T	C	C	C	C	C	C	C	C	C	C	C	T	T	T
ENSRNOSNP2783982	J561832	123808345	A	A/G	G	-	A	A	-	A/G	A	A	A	A	A	-	A	A	A	A	-	A/G	G	A/G
ENSRNOSNP2783983	rdahl-75d12_rp2_b1_105	123844713	G	G	G	G	G	G	G	G	G	G	G	G	G	G	G	G	G	G	G	G	G	G
ENSRNOSNP2783984	rdahl-2g9_fp2_b1_514	124666333	G	G	G	G	G	G	G	G	G	G	G	G	G	G	G	G	G	G	G	G	G	G
ENSRNOSNP2783985	rdahl-4j7_fp2_b1_206	124825851	A	A	A	A	A	A	A	A	A	A	A	A	A	A	A	A	A	A	A	A	A	A

ENSRNOSNP2783986	rdahl-85m10_rp2_b1_286	125067180	T	C	C	C	-	T	T	C	T	T	T	T	T	T	T	T	T	T	T	T	T	T
ENSRNOSNP2783987	WKY-G-i-43b09_r1_156	125206090	T	C	C	C	C	T	T	C	T	T	T	T	T	T	T	T	T	T	T	T	T	T
ENSRNOSNP2783988	rdahl-64f7_rp2_b1_314	125399506	G	G	G	G	G	G	G	G	G	G	G	G	G	G	G	G	G	G	G	G	-	-
ENSRNOSNP2783989	J560662	125732017	A	G	G	G	G	G	G	G	G	G	G	G	G	G	G	G	G	G	-	G	G	G
ENSRNOSNP2783990	WKYc31f03_r1_274	125744107	A	G	G	G	G	A	A	G	A	A	A	A	A	A	A	A	A	A	A	A	A	A
ENSRNOSNP2783991	gnl|ti|896459517_19866866811072_324	125888242	T	T	T	T	T	C	C	T	C	C	C	C	C	C	C	C	C	C	C	C	C	C
ENSRNOSNP2783992	J340033	125943437	C	C	C	C	C	T	T	C	T	T	T	T	T	T	T	T	T	T	T	T	T	T
ENSRNOSNP2783993	rdahl-65l17_fp2_b1_264	126031585	A	C	C	C	C	C	C	C	C	C	C	C	C	C	C	C	C	C	C	C	C	C
ENSRNOSNP2783994	SHRSPc16a12_r1_685	126182325	G	A	A	A	A	A	A	A	A	A	A	A	A	A	A	A	A	A	A	A	A	A
ENSRNOSNP2783995	J493980	126205284	C	T	T	T	T	T	T	T	T	T	T	T	T	T	T	T	T	T	-	T	T	T
ENSRNOSNP2783996	gnl|ti|896794926_19866866988801_238	126399335	T	T	T	T	T	A	A	T	A	A	A	A	A	A	A	A	A	A	A	A	A	A
ENSRNOSNP2783997	J663723	126694797	G	G	G	G	G	A	A	G	A	A	A	A	A	A	A	A	A	A	A	A	A	A
ENSRNOSNP2783998	J1264291	126785666	T	G	G	G	G	T	T	G	T	T	T	T	T	T	T	T	T	T	-	T	T	T
ENSRNOSNP2783999	rdahl-62i15_fp2_b1_120	126859750	G	A	A	A	A	A	A	A	A	A	A	A	A	A	A	A	A	A	A	A	A	A
ENSRNOSNP2784000	gnl|ti|896506642_19866866707088_346	127200967	T	C	C	C	C	T	T	C	T	T	T	T	T	T	T	T	-	T	T	C	C	C
ENSRNOSNP2784001	J513002	127221005	A	G	G	G	G	A	A	G	A	A	A	A	A	A	A	A	A	A	A	G	G	G
ENSRNOSNP2784002	gko-22g6_fp2_b1_260	127345560	G	-	A	A	A	A	G	A	G	G	G	G	G	G	G	G	G	G	G	A	A	A
ENSRNOSNP2784003	gko-63h7_fp2_b1_550	127488604	C	A	A	A	A	A	A	A	A	A	A	A	A	A	A	A	A	A	A	A	A	A
ENSRNOSNP2784004	J484245	127549453	C	A	A	A	A	A	A	A	A	A	A	A	A	A	A	A	A	A	A	A	A	A
ENSRNOSNP2784005	rdahl-51e12_fp2_b1_593	127629857	C	T	T	T	T	T	C	T	C	C	C	C	C	C	C	C	C	C	C	T	T	T
ENSRNOSNP2784006	DahlSa02a06_s1_117	127770797	C	T	T	T	T	T	C	T	C	C	C	C	C	C	C	C	C	C	C	T	T	T
ENSRNOSNP2784007	J337061	127889173	C	T	T	T	T	T	T	T	T	T	T	CT	T	T	T	T	T	T	T	T	T	T
ENSRNOSNP2784008	gko-82b16_fp2_b1_323	127910938	C	T	T	T	T	T	-	T	C	C	C	C	C	C	C	C	C	C	C	T	T	T
ENSRNOSNP2784009	SHRSPa7h07_s1_743	129396852	G	G	G	G	G	G	A	G	A	A	A	A	A	A	A	A	A	A	A	A	A	A
ENSRNOSNP2784010	J477469	129959510	T	T	T	T	T	T	C	T	C	C	C	C	C	C	C	C	C	C	-	C	C	C
ENSRNOSNP2784011	J505223	130063483	G	A	A	A	A	A	G	A	G	G	G	G	G	G	G	G	G	G	G	A	A	A
ENSRNOSNP2784012	gko-39i8_rp2_b1_95	130181654	A	A	A	A	A	A	G	A	G	G	G	G	G	G	G	G	G	G	G	G	G	G
ENSRNOSNP2784013	gko-70i2_rp2_b1_21	130473538	A	A	A	A	A	A	A	A	A	A	A	A	A	A	A	A	A	A	A	G	G	G
ENSRNOSNP2784014	J1269615	130588481	G	A	A	A	A	A	A	A	A	A	A	A	A	A	A	A	A	A	-	G	G	G
ENSRNOSNP2784015	J1278186	131035380	A	T	T	T	T	T	T	T	T	T	T	T	T	T	T	T	T	T	T	T	T	T
ENSRNOSNP2784016	rat109_012_n15.q1ca_334	131082411	C	C	C	C	C	C	A	C	A	A	A	A	A	A	A	A	A	A	A	A	A	A
ENSRNOSNP2784017	WKYe17a08_r1_604	131104776	A	G	G	G	G	G	G	G	G	G	G	G	G	G	G	G	G	G	G	G	G	G
ENSRNOSNP2784018	J488690	131227640	A	T	T	T	T	T	T	T	T	T	T	T	T	T	T	T	T	T	-	T	T	T
ENSRNOSNP2784019	WKYOa64h04_s1_476	131311649	C	T	T	T	T	T	C	T	C	C	C	C	C	C	C	C	C	C	C	C	C	C
ENSRNOSNP2784020	rat109_015_i21.q1cb_425	131354318	A	G	G	G	G	G	G	G	G	G	G	G	G	G	G	G	G	G	G	G	G	G
ENSRNOSNP2784021	J523743	131492282	G	A	A	A	A	A	A	A	A	A	A	A	A	A	A	A	A	A	A	A	A	A
ENSRNOSNP2784022	J470814	131658534	T	C	C	-	C	C	-	C	C	C	C	C	C	-	C	C	C	C	-	C	C	C
ENSRNOSNP2784023	SHRSPa43c12_s1_448	131711026	T	C	C	C	C	C	C	C	C	C	C	C	C	C	C	C	C	C	C	C	C	C
ENSRNOSNP2784024	J515676	131793615	A	A<br>C	C	-	C	-	-	-	-	C	C	C	-	-	-	C	-	-	-	A<br>C	C	A<br>C

ENSRNOSNP2784025	J525325	131893707	T	C	C	C	C	C	C	C	C	C	C	C	C	C	C	C	C	C	-	C	C	C
ENSRNOSNP2784026	gko-14e5_rp2_b1_435	131894137	T	C	C	C	C	C	C	C	C	C	C	C	C	C	C	C	C	C	C	C	C	C
ENSRNOSNP2784027	gnl|ti|896472683_19866867803712_343	132044244	A	G	G	G	G	G	G	G	G	G	G	G	G	G	G	G	G	G	G	G	G	G
ENSRNOSNP2784028	J592111	132166465	G	A	A	A	A	A	A	A	A	A	A	A	A	A	A	A	A	A	A	A	A	A
ENSRNOSNP2784029	SHRSPa40b03_s1_64	132287714	A	C	C	-	C	C	C	C	C	C	C	C	C	C	C	C	C	-	C	C	C	C
ENSRNOSNP2784030	J533431	132501440	T	C	C	C	C	C	C	C	C	C	C	C	C	C	C	C	C	C	C	C	C	C
ENSRNOSNP2784032	J634731	132914466	G	A	A	A	A	A	A	A	A	A	A	A	A	A	A	A	A	A	-	A	A	A
ENSRNOSNP2784033	WKYc33a12_s1_203	132962408	C	T	T	T	T	T	C	T	C	C	C	C	C	C	C	C	C	C	C	T	T	T
ENSRNOSNP2784034	J547318	133010282	T	C	C	C	C	C	C	C	C	C	C	C	C	C	C	C	C	C	-	C	C	C
ENSRNOSNP2784035	rdahl-90a19_fp2_b1_161	133116950	T	C	C	C	C	C	C	C	C	C	C	C	C	C	C	C	C	C	C	C	C	C
ENSRNOSNP2784036	J661392	133433124	G	T	T	T	T	T	T	T	T	T	T	T	T	T	T	T	T	T	T	T	T	T
ENSRNOSNP2784037	WKY-G-i-46a10_f1_487	133731895	A	G	G	G	G	-	G	G	G	G	G	G	G	G	G	G	G	G	G	G	G	G
ENSRNOSNP2784038	J582516	133740512	C	T	T	T	T	T	C	T	C	C	C	C	C	C	C	C	C	C	C	T	T	T
ENSRNOSNP2784039	gnl|ti|896873186_19866867442511_367	133914480	A	G	G	G	G	G	G	G	G	G	G	G	G	G	G	G	G	G	G	G	G	G
ENSRNOSNP2784040	J501490	133931084	A	G	G	G	G	G	A	G	A	A	A	A	A	A	A	A	A	A	-	G	G	G
ENSRNOSNP2784041	J639810	134056479	A	G	G	G	G	G	G	G	G	G	G	G	G	G	G	G	G	G	G	G	G	G
ENSRNOSNP2784042	J471724	134148694	T	A	A	A	A	A	T	A	T	T	T	T	T	T	T	T	T	T	-	A	A	A
ENSRNOSNP2784043	J339764	134253983	T	A	A	A	A	A	A	A	A	A	A	A	A	A	A	A	A	A	-	A	A	A
ENSRNOSNP2784044	WKYc97b12_r1_536	134341812	C	T	T	T	T	T	T	T	T	T	T	T	T	T	T	T	T	T	T	T	T	T
ENSRNOSNP2784046	gko-75p21_fp2_b1_298	134496249	T	C	C	C	C	C	C	C	C	C	C	C	C	C	C	C	C	C	C	C	C	C
ENSRNOSNP2784047	J574087	134826112	C	G	G	G	G	G	G	G	G	G	G	G	G	G	G	G	G	G	G	G	G	G
ENSRNOSNP2784048	rat109_048_n18.q1ca_161	134870070	G	A	A	A	A	A	A	A	A	A	A	A	A	A	A	A	A	A	A	A	A	A
ENSRNOSNP2784049	J682178	134990493	G	A	A	A	A	A	A	A	A	A	A	A	A	A	A	A	A	A	-	A	A	A
ENSRNOSNP2784050	Cpn_1135112460	135112460	T	G	G	G	G	G	G	G	G	G	G	G	G	G	G	G	G	G	G	G	G	G
ENSRNOSNP2784051	rat101_002_g06.p1ca_415	135115832	C	G	G	G	G	G	G	G	G	G	G	G	G	G	G	G	G	G	G	G	G	G
ENSRNOSNP2784052	J532216	135161183	A	G	G	G	G	G	G	G	G	G	G	G	G	G	G	G	G	G	G	G	G	G
ENSRNOSNP2784053	rat103_041_l11.q1ca_513	135196866	G	A	A	A	A	A	A	A	A	A	A	A	A	A	A	A	A	A	A	A	A	A
ENSRNOSNP2784054	rat110_025_i04.p1ca_231	135369940	T	C	C	C	C	C	C	C	C	C	C	C	C	C	C	C	C	C	C	C	C	C
ENSRNOSNP2784055	J880488	135467013	C	T	T	T	T	T	T	T	T	T	T	T	T	T	T	T	T	T	T	T	T	T
ENSRNOSNP2784056	rat104_001_m23.p1ca_406	135605278	C	G	G	G	G	G	G	G	G	G	G	G	G	G	G	G	G	G	G	G	G	G
ENSRNOSNP2784057	rat101_010_g17.q1ca_218	135651360	T	-	C	C	C	C	C	C	C	C	C	C	C	C	C	C	C	C	C	C	C	C
ENSRNOSNP2784058	J1295146	135775433	A	G	G	G	G	G	G	G	G	G	G	G	G	G	G	G	G	G	G	G	G	G
ENSRNOSNP2784059	rdahl-25d20_fp2_b1_427	135846868	C	T	T	T	T	T	T	T	T	T	T	T	T	T	T	T	T	T	T	T	T	T
ENSRNOSNP2784060	rat108_030_l06.p1ca_159	135877572	T	C	C	C	C	C	C	C	C	C	C	C	C	C	C	C	C	C	C	C	C	C
ENSRNOSNP2784061	rat104_001_g13.p1ca_114	136033375	G	A	A	A	A	A	G	A	G	G	G	G	G	G	G	G	G	G	G	G	G	G
ENSRNOSNP2784062	J523782	136078876	A	G	G	G	G	G	G	G	G	G	G	G	G	G	G	G	G	G	G	G	G	G
ENSRNOSNP2784063	rat109_003_f19.q1ca_263	136166822	A	G	G	G	G	G	G	G	G	G	G	G	G	G	G	G	G	G	G	G	G	G
ENSRNOSNP2784064	J567473	136206442	A	G	G	G	G	G	G	G	G	G	G	G	G	G	G	G	G	G	-	G	G	G
ENSRNOSNP2784065	WKYc92c06_s1_584	136218350	C	T	T	T	T	T	T	T	T	T	T	T	T	T	T	T	T	T	T	T	T	T

ENSRNOSNP2784066	J682137	136326140	T	C	C	C	C	C	C	C	C	C	C	C	C	C	C	C	C	C	-	C	C	C
ENSRNOSNP2784067	rat104_039_i13.q1ca_382	136552144	G	A	A	A	A	A	A	A	A	A	A	A	A	A	A	A	A	A	A	A	A	A
ENSRNOSNP2784068	rat109_029_j07.q1ca_518	136586075	A	G	G	G	G	G	G	G	G	G	G	G	G	G	G	G	G	G	G	G	G	G
ENSRNOSNP2784069	gko-64k4_rp2_b1_525	136739327	C	G	G	G	G	G	G	G	G	G	G	G	G	G	G	G	G	G	G	G	G	G
ENSRNOSNP2784070	J878219	136846086	A	T	T	T	T	T	T	T	T	T	T	T	T	T	T	T	T	T	-	T	T	T
ENSRNOSNP2784071	Cpn_1137088466	137088466	G	A	A	A	A	A	A	A	A	A	A	A	A	A	A	A	A	A	A	A	A	A
ENSRNOSNP2784073	rat104_058_d12.q1ca_172	137109541	T	C	C	C	C	C	T	C	T	T	T	T	T	T	T	T	T	T	T	T	T	T
ENSRNOSNP2784074	J583541	137157557	G	G	G	G	G	G	A	G	A	A	A	A	A	A	A	A	A	A	A	A	A	A
ENSRNOSNP2784075	WKY-G-i-38c09_f1_591	137235103	T	C	C	C	C	C	C	C	C	C	C	C	C	C	C	C	C	C	C	C	C	C
ENSRNOSNP2784076	Cpn_1137304344	137304344	T	TC	T	-	-	-	C	-	C	C	C	C	C	C	C	C	C	C	C	C	C	C
ENSRNOSNP2784077	J686814	137536814	A	G	G	G	G	G	G	G	G	G	G	G	G	G	G	G	G	G	G	G	G	G
ENSRNOSNP2784078	SHRSPc26h12_r1_226	137545537	A	A	A	A	A	A	G	A	G	G	G	G	G	G	G	G	G	G	G	G	G	G
ENSRNOSNP2784079	J515538	137707622	A	G	G	G	G	G	G	G	G	G	G	G	G	G	G	G	G	G	-	G	G	G
ENSRNOSNP2784080	rat109_045_h20.q1ca_509	137749064	G	G	G	G	G	G	A	G	A	A	A	A	A	A	A	A	A	A	A	A	A	A
ENSRNOSNP2784081	rat102_008_d20.p1ca_383	137749216	C	T	T	T	T	T	C	T	C	C	C	C	C	C	C	C	C	C	C	C	C	C
ENSRNOSNP2784082	rat109_043_p16.q1ca_308	137756320	T	C	C	C	C	C	C	C	C	C	C	C	C	C	C	C	C	C	C	C	C	C
ENSRNOSNP2784083	WKYOa63h10_s1_153	137784600	G	-	C	C	C	C	G	C	G	G	G	G	G	G	G	G	G	G	G	G	G	G
ENSRNOSNP2784084	J502851	137857367	C	C	C	C	C	C	T	C	T	T	T	T	T	T	T	T	T	T	T	T	T	T
ENSRNOSNP2784085	Cpn_1138008782	138008782	G	A	A	A	A	A	A	A	A	A	A	A	A	A	A	A	A	A	A	A	A	A
ENSRNOSNP2784086	rat102_023_b16.p1ca_217	138037820	A	G	G	G	G	G	G	G	G	G	G	G	G	G	G	G	G	G	G	G	G	G
ENSRNOSNP2784087	J548801	138077827	G	A	A	A	A	A	A	A	A	A	A	A	A	A	A	A	A	A	-	A	A	A
ENSRNOSNP2784088	Cpn_1138096182	138096182	G	G	G	G	G	G	G	G	G	G	G	G	G	G	G	G	G	G	G	G	G	G
ENSRNOSNP2784089	J695800	138230275	C	C	C	C	C	C	C	C	C	C	C	C	C	C	C	C	C	C	C	C	C	C
ENSRNOSNP2784090	gko-58j3_fp2_b1_535	138260663	C	T	T	T	T	T	T	T	T	T	T	T	T	T	T	T	T	T	T	T	T	T
ENSRNOSNP2784091	WKYe17c07_r1_591	138406217	C	T	T	T	T	T	C	T	C	C	C	C	C	C	C	C	C	C	C	T	T	T
ENSRNOSNP2784092	rat105_035_g13.q1cb_239	138529299	G	G	G	G	G	G	G	G	G	G	G	G	G	G	G	G	G	G	G	G	G	G
ENSRNOSNP2784093	gko-70n24_rp2_b1_257	138569750	C	A	A	A	A	A	C	A	C	C	C	C	C	C	C	C	C	C	C	A	A	A
ENSRNOSNP2784094	J640813	138628038	T	C	C	C	C	C	C	C	C	C	C	C	C	C	C	C	C	C	C	C	C	C
ENSRNOSNP2784095	J478219	138741608	T	C	C	C	C	C	T	C	T	T	T	T	T	T	T	T	T	T	-	C	C	C
ENSRNOSNP2784096	WKYc06c11_r1_700	138793049	A	G	G	G	G	G	A	G	A	A	A	-	A	A	A	A	-	A	A	G	G	G
ENSRNOSNP2784097	J540796	138877614	G	A	A	A	A	A	G	A	G	G	G	G	G	G	G	G	G	G	-	A	A	A
ENSRNOSNP2784098	DS-g-a-36b12_f1_125	138884787	T	C	C	C	C	C	C	C	C	C	C	C	C	C	C	C	C	C	C	C	C	C
ENSRNOSNP2784099	J556061	139058051	C	T	T	T	T	T	T	T	T	T	T	T	T	T	T	T	T	T	T	T	T	T
ENSRNOSNP2784100	WKYc109g12_s1_469	139225678	C	T	T	T	T	T	T	T	T	T	-	T	T	T	T	T	T	T	T	T	T	T
ENSRNOSNP2784101	J535160	139376966	T	C	C	C	C	C	C	C	C	C	C	C	C	C	C	C	C	C	C	C	C	C
ENSRNOSNP2784102	gnl|ti|897024582_19866867480677_384	139402562	G	G	G	G	G	G	A	G	A	A	A	A	A	A	A	A	A	A	A	A	A	A
ENSRNOSNP2784103	J690538	139723295	T	C	C	C	C	C	C	C	C	C	C	C	C	C	C	C	C	C	C	C	C	C
ENSRNOSNP2784104	DS-g-a-19g05_f1_128	139731533	A	C	C	C	C	C	C	C	C	C	C	C	C	C	C	C	C	C	C	C	C	C
ENSRNOSNP2784105	Cpn_1139908762	139908762	A	G	G	G	G	G	G	G	G	G	G	G	G	G	G	G	G	G	G	G	G	G

ENSRNOSNP2784106	J643969	139928077	T	G	G	G	G	G	G	G	G	G	G	G	G	G	G	G	G	G	-	G	G	G
ENSRNOSNP2784107	J587239	140156428	C	T	T	T	T	T	T	T	T	T	T	T	T	T	T	T	T	T	T	T	T	T
ENSRNOSNP2784108	Cpn_1140157723	140157723	G	A	A	A	A	A	A	A	A	A	A	A	A	A	A	A	A	A	A	A	A	A
ENSRNOSNP2784110	SHRSPd02b11_s1_385	140348073	A	C	C	C	C	C	C	C	C	C	C	C	C	C	C	C	C	C	C	C	C	C
ENSRNOSNP2784111	J1269962	140445019	C	G	G	G	G	G	G	G	G	G	G	G	G	G	G	G	G	G	-	G	G	G
ENSRNOSNP2784112	WKYc98h01_s1_194	140489442	T	C	C	C	C	C	C	C	C	C	C	C	C	C	C	C	C	C	C	C	C	C
ENSRNOSNP2784113	rat101_031_g24.p1ca_120	140536584	G	C	C	C	C	C	C	C	C	C	C	C	C	C	C	C	C	C	C	C	C	C
ENSRNOSNP2784114	SHRSPa28h09_s1_387	140557472	C	T	T	T	T	T	T	T	T	T	T	-	T	T	T	T	T	T	-	T	T	T
ENSRNOSNP2784115	gnl|ti|896466819_19866866741309_253	140631751	T	C	C	C	C	C	C	C	C	C	C	C	C	C	C	C	C	C	C	C	C	C
ENSRNOSNP2784116	J538856	140635848	C	T	T	T	T	T	T	T	T	T	T	T	T	T	T	T	T	T	T	T	T	T
ENSRNOSNP2784117	rdahl-93m20_fp2_b1_30	140855729	T	G	G	G	G	G	G	G	G	G	G	G	G	G	G	G	G	G	G	G	G	G
ENSRNOSNP2784118	rat110_004_b24.p1cb_433	140954681	C	T	T	T	T	T	T	T	T	T	T	T	T	T	T	T	T	T	T	T	T	T
ENSRNOSNP2784119	J632732	141047221	C	CT	C	-	C	CT	-	CT	CT	C	C	C	CT	-	C	C	-	CT	-	CT	C	CT
ENSRNOSNP2784120	rdahl-54a23_fp2_b1_378	141991143	C	T	T	T	T	T	T	T	T	T	T	T	T	T	T	T	T	T	T	T	T	T
ENSRNOSNP2784121	J502747	142202112	C	G	G	G	G	G	G	G	G	G	G	G	G	G	G	G	G	G	-	G	G	G
ENSRNOSNP2784122	J681141	142306215	A	G	G	G	G	G	G	G	G	G	G	G	G	G	G	G	G	G	-	G	G	G
ENSRNOSNP2784123	WKY-G-i-33e06_r1_299	142460692	G	T	T	T	T	T	T	T	T	T	T	T	T	T	T	T	T	T	T	T	T	T
ENSRNOSNP2784124	J481007	142523790	G	C	C	C	C	C	C	C	C	C	C	C	C	C	C	C	C	C	-	C	C	C
ENSRNOSNP2784125	gnl|ti|896833025_19866867232697_211	142629254	A	T	T	T	T	T	T	T	T	T	T	T	T	T	T	T	T	T	T	T	T	T
ENSRNOSNP2784126	J701827	142647913	C	T	T	T	T	T	T	T	T	T	T	T	T	T	T	T	T	T	-	T	T	T
ENSRNOSNP2784127	J656809	142747867	A	C	C	C	C	C	C	C	C	C	C	C	C	C	C	C	C	C	C	C	C	C
ENSRNOSNP2784128	J342513	142859802	G	A	A	A	A	A	A	A	A	A	A	A	A	A	A	A	A	A	-	A	A	A
ENSRNOSNP2784129	WKY-G-j-65d03_r1_222	142971713	C	A	A	A	A	A	A	A	A	A	A	A	A	A	A	A	A	A	A	A	A	A
ENSRNOSNP2784130	rdahl-51i14_fp2_b1_655	143203436	C	A	A	A	A	A	A	A	A	A	A	A	A	A	A	A	A	A	A	A	A	A
ENSRNOSNP2784131	J873721	143310300	C	T	T	T	T	T	T	T	T	T	T	T	T	T	T	T	T	T	T	T	T	T
ENSRNOSNP2784132	J586599	143440893	G	A	A	A	A	A	A	A	A	A	A	A	A	A	A	A	A	A	-	A	A	A
ENSRNOSNP2784133	gko-2j12_rp2_b1_467	143556969	G	A	A	A	A	A	A	A	A	A	A	A	-	A	A	A	A	A	A	A	A	A
ENSRNOSNP2784134	J513874	143650643	T	C	C	C	C	C	C	C	C	C	C	C	C	C	C	C	C	C	C	C	C	C
ENSRNOSNP2784135	J342038	143743917	A	G	G	G	G	G	G	G	G	G	G	G	G	G	G	G	G	G	-	G	G	G
ENSRNOSNP2784136	gnl|ti|896523251_19866867257922_232	143745033	T	C	C	C	C	C	C	C	C	C	C	C	C	C	C	C	C	C	C	C	C	C
ENSRNOSNP2784137	J343800	143898987	A	G	G	G	G	G	G	G	G	G	G	G	G	G	G	G	G	G	-	G	G	G
ENSRNOSNP2784138	gko-37p17_rp2_b1_373	143913178	C	A	A	A	A	A	A	A	A	A	A	A	A	A	A	A	A	A	A	A	A	A
ENSRNOSNP2784139	J533867	144006680	G	A	A	A	A	A	A	A	A	A	A	A	A	A	A	A	A	A	A	A	A	A
ENSRNOSNP2784140	J1260786	144307816	T	T	A	A	A	T	T	A	T	T	T	-	A	T	T	A	T	A	A	T	T	T
ENSRNOSNP2784141	Cpn_1144367481	144367481	C	C	T	T	T	C	C	T	C	C	C	C	T	C	C	T	C	T	T	C	C	C
ENSRNOSNP2784142	rat101_014_g21.p1ca_496	144477632	A	G	G	G	G	G	G	G	G	G	G	G	G	G	G	G	G	G	G	G	G	G
ENSRNOSNP2784143	J634537	144527766	A	C	C	C	C	C	C	C	C	C	C	C	C	C	C	C	C	C	-	C	C	C
ENSRNOSNP2784144	WKYc57d08_r1_429	144623181	A	G	G	G	G	G	G	G	G	G	G	G	G	G	G	G	G	G	G	G	G	G
ENSRNOSNP2784145	J585274	144644946	A	T	T	T	T	T	T	T	T	T	T	T	T	T	T	T	T	T	-	T	T	T

ENSRNOSNP2784146	DS-g-a-10c10_r1_777	144770806	A	T	T	T	T	T	T	T	T	T	T	T	T	T	T	T	T	T	T	T	T	T
ENSRNOSNP2784147	J647697	145067267	T	A	A	A	A	A	A	A	A	A	A	A	A	A	A	A	A	A	-	T	T	T
ENSRNOSNP2784148	J521620	145311482	G	G	G	G	G	G	G	G	G	G	G	G	G	G	G	G	G	G	-	C	C	C
ENSRNOSNP2784149	gko-25k10_fp2_b1_277	145433480	C	T	T	T	T	T	T	T	T	T	T	T	T	T	T	T	T	T	T	T	T	T
ENSRNOSNP2784151	J701324	145651709	T	A	A	A	A	A	A	A	A	A	A	A	A	A	A	A	A	A	-	T	T	T
ENSRNOSNP2784152	WKYc99b01_s1_572	146014610	C	T	T	T	T	T	T	T	T	T	T	T	T	T	T	T	-	T	T	T	T	T
ENSRNOSNP2784153	J669647	146119192	T	A	A	A	A	A	A	A	A	A	A	A	A	A	A	A	A	A	-	A	A	A
ENSRNOSNP2784154	gnl|ti|896461977_19866866743034_349	146217574	C	C	C	C	C	C	C	C	C	C	C	C	C	C	C	C	C	C	C	C	C	C
ENSRNOSNP2784155	rat104_066_f11.q1ca_135	146221008	C	T	T	T	T	T	T	T	T	T	T	T	T	T	T	T	T	T	T	C	C	C
ENSRNOSNP2784156	J882017	146309986	T	T	T	T	T	T	T	T	T	T	T	T	T	T	T	T	T	T	T	T	T	T
ENSRNOSNP2784157	J545173	146400566	C	T	T	-	T	T	-	T	T	T	T	T	T	-	T	T	T	T	-	T	T	T
ENSRNOSNP2784158	rat101_016_d14.p1ca_689	146419309	A	C	C	C	C	C	C	C	C	C	C	C	C	C	C	C	C	C	C	C	C	C
ENSRNOSNP2784159	gnl|ti|896894141_19866868331801_225	146423470	G	G	G	G	G	G	G	G	G	G	G	G	G	G	G	G	-	G	G	G	G	G
ENSRNOSNP2784160	rdahl-92k4_fp2_b1_117	146565292	C	C	C	C	C	C	C	C	C	C	C	C	C	C	C	C	C	C	C	C	C	C
ENSRNOSNP2784161	J654059	146622802	A	A	A	A	A	A	A	A	A	A	A	A	A	A	A	A	A	A	A	A	A	A
ENSRNOSNP2784162	gnl|ti|896637507_19866867262295_206	146705742	A	A	A	A	A	A	A	A	A	A	A	A	A	A	A	A	A	A	A	C	C	C
ENSRNOSNP2784163	J634525	146724951	C	C	C	C	C	C	C	C	C	C	C	C	C	C	C	C	C	C	-	C	C	C
ENSRNOSNP2784164	rat101_003_e20.p1ca_104	146834169	C	T	T	T	T	T	T	T	T	T	T	T	T	T	T	T	T	T	T	C	C	C
ENSRNOSNP2784166	Cpn_1147203547	147125141	G	-	G	G	G	A	-	G	-	A	G/A	A	G	A	A	-	-	G	G	G	G	G
ENSRNOSNP2784167	rat103_033_p01.q1ca_602	147154816	T	C	C	C	C	C	C	C	C	C	C	C	C	C	C	C	C	C	C	C	C	C
ENSRNOSNP2784168	gko-50b3_rp2_b1_419	147290621	T	C	C	C	C	C	C	C	C	C	C	C	C	C	C	C	C	C	C	C	C	C
ENSRNOSNP2784169	rdahl-98a11_rp2_b1_41	147574590	C	G	C	C	C	G	G	C	G	G	G	G	C	G	G	C	G	C	C	C	C	C
ENSRNOSNP2784170	J1275899	147623155	C	T	T	T	T	T	T	T	T	T	T	T	T	T	T	T	T	T	T	T	T	T
ENSRNOSNP2784171	J488760	147732780	C	T	T	T	T	T	T	T	T	T	T	T	T	T	T	T	T	T	-	T	T	T
ENSRNOSNP2784172	DS-g-c-07g03_f1_723	147774796	C	-	C	C	C	T	T	C	T	T	T	T	C	T	T	C	C	C	C	C	C	C
ENSRNOSNP2784173	gko-34m21_fp2_b1_279	147916215	C	A	A	A	A	A	A	A	A	A	A	A	A	A	A	A	A	A	A	A	A	A
ENSRNOSNP2784174	J532788	147926781	A	A	A	A	A	A	A	A	A	A	A	A	A	A	A	A	A	A	A	-	T	T
ENSRNOSNP2784175	gko-6n14_fp2_b2_813	148075777	C	T	C	C	C	T	T	C	T	T	T	T	C	T	T	-	C	C	C	T	T	T
ENSRNOSNP2784176	rat105_002_c16.p1ma_479	148211720	G	A	A	A	A	A	A	A	A	A	A	A	A	A	A	A	A	A	A	A	A	A
ENSRNOSNP2784177	J539462	148275814	G	A	A	A	A	A	A	A	A	A	A	A	A	A	A	A	A	A	A	A	A	A
ENSRNOSNP2784178	gnl|ti|896883940_19866868449661_290	148376298	C	C	C	C	C	C	C	-	C	C	C	C	C	C	C	C	C	C	C	C	C	C
ENSRNOSNP2784179	J680809	148590629	C	C	C	C	C	C	C	C	C	C	C	C	C	C	C	C	C	C	C	C	C	C
ENSRNOSNP2784180	SHRSPa16b06_r1_329	148596494	G	C	C	C	C	C	C	C	C	C	C	C	C	C	C	C	C	C	C	C	C	C
ENSRNOSNP2784181	J549705	148913951	A	G	G	G	G	G	G	G	G	G	G	G	G	G	G	G	G	G	G	G	G	G
ENSRNOSNP2784182	WKYc62a02_s1_430	149142304	T	G	G	G	G	G	G	G	G	G	G	G	G	G	G	G	G	G	G	G	G	G
ENSRNOSNP2784183	J872480	149258049	C	A	A	A	A	A	A	A	A	A	A	A	A	A	A	A	A	A	A	A	A	A
ENSRNOSNP2784184	rat101_030_d13.p1ca_216	149278383	G	A	A	-	A	A	-	A	A	A	A	A	A	-	A	A	A	A	-	A	A	A
ENSRNOSNP2784185	SHRSPc13h06_r1_588	149485945	T	C	C	C	C	C	C	C	C	C	C	C	C	C	C	C	C	C	C	C	C	C
ENSRNOSNP2784186	J576094	149585877	C	T	T	T	T	T	T	T	T	T	T	T	T	T	T	T	T	T	T	T	T	T

ENSRNOSNP2784187	rdahl-26k16_rp2_b1_31	149647414	T	C	C	C	C	C	C	C	C	C	C	C	C	C	C	C	C	C	C	C	-	-
ENSRNOSNP2784188	rat102_008_m19.q1ca_590	149677673	G	A	A	A	A	A	A	A	A	A	A	A	A	A	A	A	A	A	A	A	A	A
ENSRNOSNP2784189	J521480	149719493	G	A	A	A	A	A	A	A	A	A	A	A	A	A	A	A	A	A	-	A	A	A
ENSRNOSNP2784190	J341551	149850859	T	C	C	C	C	C	C	C	C	C	C	C	C	C	C	C	C	C	-	C	C	C
ENSRNOSNP2784191	rdahl-71m20_fp2_b1_25	149853461	G	A	A	A	A	A	A	A	A	A	A	A	A	A	A	A	A	A	A	A	A	A
ENSRNOSNP2784193	gko-96e1_fp2_b1_312	150096169	T	A	A	A	A	A	A	A	A	A	A	A	A	A	A	A	A	A	A	A	A	A
ENSRNOSNP2784194	gko-69e22_rp2_b1_389	150531490	A	G	G	G	G	G	G	G	G	G	G	G	G	G	G	G	G	G	G	G	G	G
ENSRNOSNP2784195	J590265	150665121	G	A	A	A	A	A	A	A	A	A	A	A	A	A	A	A	A	A	-	A	A	A
ENSRNOSNP2784196	rdahl-20h11_fp2_b1_101	150715523	C	T	T	T	T	T	T	T	T	T	T	T	T	T	T	T	T	T	T	T	-	-
ENSRNOSNP2784197	rdahl-15h19_rp2_b1_111	150871328	G	A	A	A	A	A	A	A	A	A	A	A	A	A	A	A	A	A	A	A	A	A
ENSRNOSNP2784198	gko-61d19_rp2_b1_355	151036704	G	A	A	A	A	A	A	A	A	A	A	A	A	A	A	A	A	A	A	A	A	A
ENSRNOSNP2784199	J545191	151073477	C	C	C	-	C	C	-	C	C	C	C	C	C	-	C	C	C	C	-	C	C	C
ENSRNOSNP2784200	rat108_034_p17.p1ca_344	151110344	C	T	T	T	T	T	T	T	T	T	T	T	T	T	T	T	T	T	T	T	T	T
ENSRNOSNP2784201	J342906	151176087	C	T	T	T	T	T	T	T	T	T	T	T	T	T	T	T	T	T	T	T	T	T
ENSRNOSNP2784202	gko-14a24_fp2_b1_770	151184006	G	C	C	C	C	C	C	C	C	C	C	C	C	C	C	C	C	C	C	C	C	C
ENSRNOSNP2784203	gko-19n8_fp2_b1_332	151328091	A	G	G	G	G	G	G	G	G	G	G	G	G	G	G	G	G	G	G	G	G	G
ENSRNOSNP2784204	gko-84o14_fp2_b1_603	151502658	T	C	C	C	C	C	C	C	C	C	C	C	C	-	C	C	C	C	C	C	C	C
ENSRNOSNP2784205	J1268261	151532258	T	C	C	C	C	C	C	C	C	C	C	C	C	C	C	C	C	C	C	C	C	C
ENSRNOSNP2784206	gko-52n12_rp2_b1_88	151751640	C	T	T	T	T	T	T	T	T	T	T	T	T	T	T	T	T	T	T	T	T	T
ENSRNOSNP2784207	J686665	151867905	C	A	A	A	A	A	A	A	A	A	A	A	A	A	A	A	A	A	A	A	A	A
ENSRNOSNP2784208	gko-16b9_rp2_b1_493	151950962	A	G	G	G	G	G	G	G	G	G	G	G	G	G	G	G	G	G	G	G	G	G
ENSRNOSNP2784209	gko-72i3_rp2_b1_55	152252637	C	T	T	T	T	T	T	T	T	T	T	T	T	T	T	T	T	T	T	T	T	T
ENSRNOSNP2784210	rdahl-24k12_fp2_b1_606	152447482	C	T	T	T	T	T	T	T	T	T	T	T	T	T	T	T	T	T	T	T	T	T
ENSRNOSNP2784212	SHRSPc66a05_r1_451	152809150	G	A	A	A	A	A	A	A	A	A	A	A	A	A	A	A	A	A	A	A	A	A
ENSRNOSNP2784213	gko-107n14_rp2_b1_139	152969343	C	T	T	T	T	T	T	T	T	T	T	T	T	T	T	T	T	T	T	T	T	T
ENSRNOSNP2784214	J579501	153032526	G	T	T	T	T	T	T	T	T	T	T	T	T	T	T	T	T	T	-	T	T	T
ENSRNOSNP2784215	J505115	153284646	G	C	C	-	C	C	-	C	C	C	C	C	C	-	C	C	C	C	-	C	C	C
ENSRNOSNP2784216	WKYe09g11_r1_768	153326053	T	C	C	C	C	C	C	C	C	C	C	C	C	C	C	C	C	C	C	C	C	C
ENSRNOSNP2784217	rat105_040_f05.q1ca_469	153472057	T	G	G	G	G	G	G	G	G	G	G	G	G	G	G	G	G	G	G	G	G	G
ENSRNOSNP2784218	J520598	153477913	T	C	C	C	C	C	C	C	C	C	C	C	C	C	C	C	C	C	-	C	C	C
ENSRNOSNP2784219	SHRSPc14d03_r1_326	153483576	T	C	C	C	C	C	C	C	C	C	C	C	C	C	C	C	C	C	C	C	C	C
ENSRNOSNP2784220	J475269	153623495	G	A	A	A	A	A	A	A	A	A	A	A	A	A	A	A	A	A	A	A	A	A
ENSRNOSNP2784221	SHRSPa06f12_r1_1033	153817413	G	A	-	A	A	A	A	A	A	A	A	A	A	A	A	A	A	A	A	A	A	A
ENSRNOSNP2784222	J571366	153945949	A	G	G	G	G	G	G	G	G	G	G	G	G	G	G	G	G	G	G	G	G	G
ENSRNOSNP2784223	J524373	154061178	C	T	T	T	T	T	T	T	T	T	T	T	T	T	T	T	T	T	-	T	T	T
ENSRNOSNP2784224	SHRSPc38f04_r1_198	154092675	A	G	G	G	G	G	G	G	G	G	G	G	G	G	G	G	G	G	G	G	G	G
ENSRNOSNP2784225	rat101_021_a17.q1ca_666	154143673	G	A	A	A	A	A	A	A	A	A	A	A	A	A	A	A	A	A	A	A	A	A
ENSRNOSNP2784227	DahlSa01f01_r1_140	154390719	C	T	T	T	T	T	T	T	T	T	T	T	T	T	T	T	T	T	T	T	T	T
ENSRNOSNP2784228	J578596	154495830	T	C	C	-	C	C	-	C	C	C	C	C	C	-	C	C	C	C	-	C	C	C

ENSRNOSNP2784229	rat105_024_f04.p1ca_302	154589372	T	G	G	G	G	G	G	G	G	G	G	G	G	G	G	G	G	G	G	G	G	G
ENSRNOSNP2784230	rat107_004_j03.p1ma_337	154627781	G	G	G	G	G	G	G	G	G	G	G	G	G	G	G	G	G	G	G	G	G	G
ENSRNOSNP2784231	rat103_035_a15.q1ca_614	154648007	T	C	C	C	C	C	C	C	C	C	C	C	C	C	C	C	C	C	C	C	C	C
ENSRNOSNP2784232	Cpn_1154778217	154778217	C	T	T	T	T	T	T	T	T	T	T	T	T	T	T	T	T	T	T	T	T	T
ENSRNOSNP2784233	Cpn_1154911435	154911435	T	C	C	C	C	C	C	C	C	C	C	C	C	C	C	C	C	C	C	C	C	C
ENSRNOSNP2784234	J342405	154963585	A	G	G	G	G	G	G	G	G	G	G	G	G	G	G	G	G	G	G	G	G	G
ENSRNOSNP2784235	Cpn_1154966809	154966809	G	T	T	T	T	T	T	T	T	T	T	T	T	T	T	T	T	T	T	T	T	T
ENSRNOSNP2784236	J585860	155150103	T	C	C	C	C	C	C	C	C	C	C	C	C	C	C	C	C	C	-	C	C	C
ENSRNOSNP2784237	J1295779	155282273	A	C	C	C	C	C	C	C	C	C	C	C	C	C	C	C	C	C	C	C	C	C
ENSRNOSNP2784238	rat104_002_l06.p1ma_243	155292506	G	A	A	A	A	A	A	A	A	A	A	A	A	A	A	A	A	A	A	A	A	A
ENSRNOSNP2784239	J542104	155437180	A	G	G	G	G	G	G	G	G	G	G	G	G	G	G	G	G	G	-	G	G	G
ENSRNOSNP2784240	gko-30d14_rp2_b1_25	155450447	A	G	G	G	G	G	G	G	G	G	G	G	G	G	G	G	G	G	G	G	G	G
ENSRNOSNP2784241	J876769	155549548	C	T	T	T	T	T	T	T	T	T	T	T	T	T	T	T	T	T	-	T	T	T
ENSRNOSNP2784242	rat013_041_b20.p1ca_271	155612106	C	T	T	T	T	T	T	T	T	T	T	T	T	T	T	T	T	T	T	T	T	T
ENSRNOSNP2784243	J470491	155731694	A	G	G	G	G	G	G	G	G	G	G	G	G	G	G	G	G	G	G	G	G	G
ENSRNOSNP2784244	J571514	155948907	C	T	T	T	T	T	T	T	T	T	T	T	T	T	T	T	T	T	-	T	T	T
ENSRNOSNP2784245	gko-119d13_fp2_b1_409	155950033	A	C	C	C	C	C	C	C	C	C	C	C	C	C	C	C	C	C	C	C	C	C
ENSRNOSNP2784246	SHRSPa06e11_r1_357	156122682	A	G	G	G	G	G	G	G	G	G	G	G	G	G	G	G	G	G	G	G	G	G
ENSRNOSNP2784247	gko-69l19_fp2_b1_372	156265163	T	C	C	C	C	C	C	C	C	C	C	C	C	C	C	C	C	C	C	C	C	C
ENSRNOSNP2784248	rdahl-26a6_rp2_b1_405	156415886	G	G	G	G	G	G	G	G	G	G	G	G	G	G	G	G	G	G	G	G	G	G
ENSRNOSNP2784249	J641794	156617059	C	C	C	C	C	C	C	C	C	C	C	C	C	C	C	C	C	C	-	C	C	C
ENSRNOSNP2784250	rat108_008_n19.p1cb_412	156641763	G	T	T	T	T	T	T	T	T	T	T	T	T	T	T	T	T	T	T	T	T	T
ENSRNOSNP2784251	Cpn_1156642138	156642138	C	-	T	T	T	T	T	T	T	T	T	T	T	T	T	T	T	T	T	T	T	T
ENSRNOSNP2784252	rat105_027_i03.q1ca_351	156667149	G	G	G	G	G	G	G	G	G	G	G	G	G	G	G	G	G	G	G	G	G	G
ENSRNOSNP2784253	SHRSPc26b10_s1_136	156773249	G	T	T	T	T	T	T	T	T	T	T	T	T	T	T	T	T	T	T	T	T	T
ENSRNOSNP2784254	J1277508	156785079	G	A	A	A	A	A	A	A	A	A	A	A	A	A	A	A	A	A	-	A	A	A
ENSRNOSNP2784255	rat108_017_f18.p1cb_302	156942068	C	G	G	G	G	G	G	G	G	G	G	G	G	G	G	G	G	G	G	G	G	G
ENSRNOSNP2784256	Cpn_1156992602	156992602	A	-	G	G	G	G	G	G	G	G	G	G	G	G	G	G	G	G	G	G	G	G
ENSRNOSNP2784257	J469007	157005756	T	C	C	C	C	C	C	C	C	C	C	C	C	C	C	C	C	C	C	C	C	C
ENSRNOSNP2784258	J337185	157285398	T	C	C	C	C	C	C	C	C	C	C	C	C	C	C	C	C	C	-	C	C	C
ENSRNOSNP2784259	rat103_040_i10.p1ca_352	157452645	C	C	C	C	C	C	C	C	C	C	C	C	C	C	C	C	C	C	C	C	C	C
ENSRNOSNP2784260	DS-g-a-04f12_f1_621	157603989	T	C	C	C	C	T	T	C	T	T	T	T	T	T	T	T	T	T	T	T	T	T
ENSRNOSNP2784261	rat102_027_m14.p1ca_76	157644127	G	A	A	A	A	G	G	A	G	G	G	G	G	G	G	G	G	G	G	G	G	G
ENSRNOSNP2784262	rat108_043_p19.q1ca_400	157654929	G	G	G	G	G	A	A	G	A	A	A	A	A	A	A	A	A	A	A	A	A	A
ENSRNOSNP2784263	J695556	157786204	A	A	A	A	A	A	A	A	A	A	A	A	A	A	A	A	A	A	-	A	A	A
ENSRNOSNP2784264	gko-118p18_rp2_b1_257	157840347	G	A	A	A	A	A	A	A	A	A	A	A	A	A	A	A	A	A	A	A	A	A
ENSRNOSNP2784265	Cpn_1157893099	157893099	C	CT	T	T	T	T	T	T	T	T	T	T	T	T	T	T	T	T	T	T	T	T
ENSRNOSNP2784266	Cpn_1157980292	157980292	C	T	T	T	T	C	C	T	C	C	C	C	C	C	C	C	C	C	C	C	C	C
ENSRNOSNP2784267	J657141	158009944	T	A	A	A	A	A	A	A	A	A	A	A	A	A	A	A	A	A	A	A	A	A

ENSRNOSNP2784268	Cpn_1158079778	158079778	A	A	A	A	A	A	A	A	A	A	A	A	A	A	A	A	A	A	A	A	A	A
ENSRNOSNP2784269	rat108_018_g17.p1ca_325	158082648	T	T	T	T	T	C	C	T	C	C	C	C	C	C	C	C	C	C	C	C	C	C
ENSRNOSNP2784270	rat108_003_p06.p1ca_275	158285641	T	A	A	A	A	A	A	A	A	A	A	A	A	A	A	A	A	A	A	A	A	A
ENSRNOSNP2784271	rat108_003_p06.q1ca_691	158286851	C	T	T	T	T	T	T	T	T	T	T	T	T	T	T	T	T	T	T	T	T	T
ENSRNOSNP2784272	Cpn_1158319587	158319587	A	A	A	A	A	C	C	A	C	C	C	C	C	C	C	C	C	C	C	C	C	C
ENSRNOSNP2784273	J1272680	158343064	G	A	A	A	A	G	G	A	G	G	G	G	G	G	G	G	G	G	G	G	G	G
ENSRNOSNP2784274	rat103_024_i08.p1ca_333	158440449	A	G	G	G	G	G	G	G	G	G	G	G	G	G	G	G	G	G	G	G	G	G
ENSRNOSNP2784275	J634682	158454843	T	A	A	A	A	A	A	A	A	A	A	A	A	A	A	A	A	A	-	A	A	A
ENSRNOSNP2784276	WKYe08c01_r1_536	158512936	A	C	C	C	C	C	C	C	C	C	C	C	C	C	C	C	C	C	C	C	C	C
ENSRNOSNP2784277	WKYc69b08_s1_784	158667110	T	C	C	C	C	T	T	C	T	T	T	T	T	T	T	T	T	T	T	T	T	T
ENSRNOSNP2784278	J594649	158773079	G	G	G	G	G	T	T	G	T	T	T	T	T	T	T	T	T	T	T	T	T	T
ENSRNOSNP2784279	rat013_024_b07.p1ca_290	158820192	C	T	T	T	T	C	C	T	C	C	C	C	C	C	C	C	C	C	C	C	C	C
ENSRNOSNP2784280	Cpn_1158844904	158844904	G	G	G	G	G	A	A	G	A	A	A	A	A	A	A	A	A	A	A	A	A	A
ENSRNOSNP2784281	rat013_057_m18.q1ca_333	158904692	A	C	C	C	C	A	A	C	A	A	A	A	A	A	A	A	A	A	A	A	A	A
ENSRNOSNP2784282	J1265379	158993924	G	A	A	-	A	A	-	A	A	A	A	A	A	-	A	A	A	A	-	A	A	A
ENSRNOSNP2784283	rat110_002_h23.p1ca_654	159012679	C	C	C	C	C	T	T	C	T	T	T	T	T	T	T	T	T	T	T	T	T	T
ENSRNOSNP2784284	Cpn_1159013781	159013781	C	C	C	C	C	C	C	C	C	C	C	C	C	C	C	C	C	C	C	C	C	C
ENSRNOSNP2784285	DS-g-a-04a01_f1_779	159056642	T	T	T	T	T	C	C	T	C	C	C	C	C	C	C	C	C	C	C	C	C	C
ENSRNOSNP2784286	rat101_030_d08.q1ca_628	159252425	A	G	G	G	G	G	G	G	G	G	G	G	G	G	G	G	G	G	G	G	G	G
ENSRNOSNP2784287	Cpn_1159252677	159252677	T	C	C	C	C	C	C	C	C	C	C	C	C	C	C	C	C	C	C	C	C	C
ENSRNOSNP2784288	rat104_001_n08.q1ca_227	159315306	T	G	G	G	G	T	T	G	T	T	T	T	T	T	T	T	T	T	T	T	T	T
ENSRNOSNP2784289	WKYc72c05_r1_330	159379966	G	T	T	T	T	G	G	T	G	G	G	G	G	G	G	G	G	G	G	G	G	G
ENSRNOSNP2784290	WKYc15d09_s1_288	159502725	T	C	C	C	C	C	C	C	C	C	C	C	C	C	C	C	C	C	C	C	C	C
ENSRNOSNP2784291	J549858	159592122	T	G	G	G	G	G	G	G	G	G	G	G	G	G	G	G	G	G	G	G	G	G
ENSRNOSNP2784292	rat101_010_c19.p1ca_200	159600736	A	G	G	G	G	A	A	G	A	A	A	A	A	A	A	A	A	A	A	A	A	A
ENSRNOSNP2784293	J552483	159712285	C	C	C	C	C	T	T	C	T	T	T	T	T	T	T	T	T	T	-	T	T	T
ENSRNOSNP2784294	J538041	159923068	T	C	C	C	C	C	C	C	C	C	C	C	C	C	C	C	C	C	C	C	C	C
ENSRNOSNP2784295	gnl|ti|896461875_19866866743025_311	160129372	A	A	A	A	A	A	A	A	A	A	A	A	A	A	A	A	A	A	A	A	A	A
ENSRNOSNP2784296	J682192	160165958	T	C	C	C	C	T	T	C	T	T	T	T	T	T	T	T	T	T	-	T	T	T
ENSRNOSNP2784297	J668724	160499973	A	A<br>G	G	-	G	-	-	-	-	G	G	G	-	-	-	G	-	-	-	A<br>G	G	G
ENSRNOSNP2784298	gko-21j19_fp2_b1_361	160640313	C	C	C	C	C	T	T	C	T	T	T	T	T	T	T	T	T	T	T	T	T	T
ENSRNOSNP2784299	gnl|ti|896503444_19866868493666_284	160788294	T	T	T	T	T	T	T	T	T	T	T	T	T	T	T	T	T	T	T	T	T	T
ENSRNOSNP2784300	J679265	160914918	A	C	C	C	C	C	C	C	C	C	C	C	C	C	C	C	C	C	C	C	C	C
ENSRNOSNP2784301	rdahl-31g14_rp2_b1_216	160977730	A	A	A	A	A	A	A	A	A	A	A	A	A	A	A	A	A	A	A	A	A	A
ENSRNOSNP2784302	J596350	161021274	G	G	G	G	G	A	A	G	A	A	A	A	A	A	A	A	A	A	-	A	A	A
ENSRNOSNP2784303	J1264446	161244643	C	T	T	T	T	T	T	T	T	T	T	T	T	T	T	T	T	T	T	T	T	T
ENSRNOSNP2784304	WKY-G-i-16e01_f1_255	161255791	C	G	G	G	G	G	G	G	G	G	G	G	G	G	G	G	G	G	G	G	G	G
ENSRNOSNP2784305	J551789	161348714	G	A	A	A	A	A	A	A	A	A	A	A	A	A	A	A	A	A	-	A	A	A
ENSRNOSNP2784306	gnl|ti|896892585_19866868391687_284	161441063	A	G	G	G	G	G	G	G	G	G	G	G	G	G	G	G	G	G	G	G	G	G

ENSRNOSNP2784307	rat105_033_m22.p1ca_267	161597761	T	T	T	T	T	T	T	T	T	T	T	T	T	T	T	T	T	T	T	T	T	T
ENSRNOSNP2784308	J595387	161661740	T	C	C	C	C	C	C	C	C	C	C	C	C	C	C	C	C	C	C	C	C	C
ENSRNOSNP2784309	J499581	161857704	G	A	A	A	A	A	A	A	A	A	A	A	A	A	A	A	A	A	-	A	A	A
ENSRNOSNP2784310	gnl|ti|896581974_19866867296351_255	162245247	T	T	T	T	T	T	C	T	C	C	C	C	C	C	C	C	C	C	-	T	T	T
ENSRNOSNP2784311	rdahl-96c10_rp2_b1_335	162391700	C	C	C	C	C	C	T	C	T	T	T	T	T	T	T	-	T	T	T	C	C	C
ENSRNOSNP2784312	gko-34i22_fp2_b1_479	162743882	C	A	A	A	A	A	C	A	C	C	C	C	C	C	C	C	C	C	C	A	A	A
ENSRNOSNP2784313	rdahl-76b14_fp2_b1_257	162885765	T	C	C	C	C	C	C	C	C	C	C	C	C	C	C	C	C	C	C	C	C	C
ENSRNOSNP2784314	J540758	162999602	C	T	T	T	T	T	T	T	T	T	T	T	T	T	T	T	T	T	-	T	T	T
ENSRNOSNP2784315	rat105_016_d18.q1ca_152	163167086	C	T	T	T	T	T	T	T	T	T	T	T	T	T	T	T	T	T	T	T	T	T
ENSRNOSNP2784316	J698297	163211642	T	C	C	C	C	C	C	C	C	C	C	C	C	C	C	C	C	C	C	C	C	C
ENSRNOSNP2784317	Cpn_1163213039	163213039	G	-	T	T	T	T	G	T	G	G	G	G	G	G	G	G	G	G	G/T	T	T	T
ENSRNOSNP2784318	rat108_028_o16.q1ca_301	163283110	G	G	G	G	G	G	-	G	A	A	A	A	A	A	A	A	A	A	A	G	G	G
ENSRNOSNP2784319	rat013_009_g11.p1ca_404	163320085	G	A	G	A	G	A	A	A	A	G	G	G	A	A	A	G	A	A	A	A	G	G
ENSRNOSNP2784320	J649757	163320735	C	T	T	T	T	T	T	T	T	T	T	T	T	T	T	T	T	T	-	T	T	T
ENSRNOSNP2784321	gnl|ti|896505209_19866867106466_245	163477674	T	C	C	C	C	C	C	C	C	C	C	C	C	C	C	C	C	C	-	C	C	C
ENSRNOSNP2784322	rdahl-98f15_rp2_b1_293	163886079	G	G	G	G	G	-	T	G	T	T	T	T	T	T	T	T	T	T	T	G	G	G
ENSRNOSNP2784323	gko-93e11_rp2_b1_463	164290219	T	A	A	A	A	A	A	A	A	A	A	A	A	A	A	A	A	A	A	A	A	A
ENSRNOSNP2784324	J695568	164656091	T	T	T	T	T	T	C	T	C	C	C	C	C	C	C	C	C	C	C	T	T	T
ENSRNOSNP2784325	J539170	164769352	T	C	C	C	C	C	T	C	T	T	T	T	T	T	T	T	T	T	-	C	C	C
ENSRNOSNP2784326	J1273532	164880269	A	G	G	G	G	G	G	G	G	G	G	G	G	G	G	G	G	G	-	G	G	G
ENSRNOSNP2784327	SHRSPc09g06_r1_639	164909020	T	C	C	C	C	C	C	C	C	C	C	C	C	C	C	C	C	C	C	C	C	C
ENSRNOSNP2784328	J882378	164977429	T	T	T	T	T	T	C	T	C	C	C	C	C	C	C	C	C	C	C	T	T	T
ENSRNOSNP2784329	SHRSPa31a11_r1_810	164995363	G	G	G	G	G	G	T	G	T	T	T	T	T	T	T	T	T	T	T	G	G	G
ENSRNOSNP2784330	J597551	165099494	G	C	C	C	C	C	G	C	G	G	G	G	G	G	G	G	G	G	-	C	C	C
ENSRNOSNP2784331	rat109_054_l19.q1ca_375	165131370	A	G	G	G	G	G	G	G	G	G	G	G	G	G	G	G	G	G	G	G	G	G
ENSRNOSNP2784332	rdahl-50a2_rp2_b1_332	165139197	T	C	C	C	C	C	C	C	C	C	C	C	C	C	C	C	C	C	C	C	C	C
ENSRNOSNP2784333	J529689	165288315	G	A	A	A	A	A	A	A	A	A	A	A	A	A	A	A	A	A	A	A	A	A
ENSRNOSNP2784334	gko-63e5_rp2_b1_447	165289921	A	C	C	C	C	C	C	C	C	C	C	C	C	C	C	C	C	C	C	C	C	C
ENSRNOSNP2784335	gko-95l3_rp2_b1_91	165483208	A	C	C	C	C	C	C	C	C	C	C	C	C	C	C	C	C	C	C	C	C	C
ENSRNOSNP2784336	J668682	165582371	A	A	A	A	A	A	C	A	C	C	C	C	C	C	C	C	C	C	-	A	A	A
ENSRNOSNP2784337	gnl|ti|896958457_19866867470784_367	165706254	C	C	C	C	C	C	G	C	G	G	G	G	G	G	G	G	G	G	G	C	C	C
ENSRNOSNP2784338	J574813	165830647	A	G	G	G	G	G	G	G	G	G	G	G	G	G	G	G	G	G	-	G	G	G
ENSRNOSNP2784339	J1269255	166394801	A	A/G	G	-	G	A/G	-	A/G	A/G	G	G	G	A/G	-	A/G	G	A/G	A/G	-	A/G	G	A/G
ENSRNOSNP2784340	WKY-G-f-11d04_r1_413	166395179	G	A	A	A	A	A	G	A	G	G	G	G	G	G	G	G	G	G	G	A	A	A
ENSRNOSNP2784341	rat106_024_l08.p1cb_347	166399733	T	T	T	T	T	T	C	T	C	C	C	C	C	C	C	C	C	C	C	T	T	T
ENSRNOSNP2784342	WKYc109d11_r1_430	166658875	T	C	C	C	C	C	T	C	T	T	T	T	T	T	T	T	T	T	T	C	C	C
ENSRNOSNP2784343	WKYOa47f11_s1_383	166767579	T	C	C	C	C	C	T	C	T	T	T	T	T	T	T	T	T	T	T	C	C	C
ENSRNOSNP2784344	SHRSPc47c01_r1_397	166850332	T	C	C	C	-	C	C	C	C	C	C	C	C	C	C	C	C	C	C	C	C	C
ENSRNOSNP2784345	DS-g-c-10e09_r1_416	166927477	T	T	-	T	T	T	C	T	C	C	C	C	C	C	C	C	C	C	C	T	T	T

ENSRNOSNP2784346	J1284013	166957383	G	A	A	A	A	A	G	A	G	G	G	G	G	G	G	G	G	G	-	A	A	A
ENSRNOSNP2784347	WKYOa57f10_s1_409	166988352	G	T	T	T	T	T	G	T	G	G	G	G	G	G	G	G	G	G	G	T	T	T
ENSRNOSNP2784348	rat105_013_l18.p1ca_185	167049054	C	G	G	G	G	G	G	G	G	G	G	G	G	G	G	G	G	G	G	G	G	G
ENSRNOSNP2784349	WKYd02c05_s1_301	167055022	T	G	G	G	G	G	T	G	T	T	T	T	T	T	T	T	T	T	T	G	G	G
ENSRNOSNP2784350	J519146	167073437	A	G	G	G	G	G	A	G	A	A	A	A	A	A	A	A	A	A	A	G	G	G
ENSRNOSNP2784351	rat109_047_d05.q1cb_497	167120104	C	T	T	T	T	T	T	T	T	T	T	T	T	T	T	T	T	T	T	T	T	T
ENSRNOSNP2784352	J697425	167169987	C	T	T	T	T	T	T	T	T	T	T	T	T	T	T	T	T	T	-	T	T	T
ENSRNOSNP2784353	J686236	167293648	A	A	A	A	A	A	G	A	G	G	G	G	G	G	G	G	G	G	-	A	A	A
ENSRNOSNP2784354	WKYe18e07_r1_731	167305861	A	G	G	G	G	G	A	G	A	A	A	A	A	A	A	A	A	A	A	G	G	G
ENSRNOSNP2784355	rat102_009_e12.p1ca_394	167519996	G	A	A	A	A	A	A	A	A	A	A	A	A	A	A	A	A	A	A	A	A	A
ENSRNOSNP2784356	rat101_009_e23.p1ca_224	167609450	G	A	A	A	A	A	A	A	A	A	A	A	A	A	A	A	A	A	A	A	A	A
ENSRNOSNP2784357	J696429	167613220	C	A	A	A	A	A	A	A	A	A	A	A	A	A	A	A	A	A	-	A	A	A
ENSRNOSNP2784358	rat108_039_g23.p1ca_194	167622373	A	G	G	G	G	G	G	G	G	G	G	G	G	G	G	G	G	G	G	G	G	G
ENSRNOSNP2784359	J512540	167882970	A	G	G	-	G	G	-	G	G	G	G	G	G	-	G	G	G	G	-	G	G	G
ENSRNOSNP2784360	J549135	168065170	A	G	G	G	G	G	A	G	A	A	A	A	A	A	A	A	A	A	A	G	G	G
ENSRNOSNP2784361	WKYc15h05_s1_114	168118608	A	G	G	G	G	G	-	G	A	A	A	A	A	A	A	A	A	A	A	G	G	G
ENSRNOSNP2784362	WKYc13h03_s1_138	168354892	G	A	A	A	A	A	G	A	G	G	G	G	G	G	G	G	G	G	G	A	A	A
ENSRNOSNP2784363	J673755	168448018	G	G	G	G	G	G	A	G	A	A	A	A	A	A	A	A	A	A	A	G	G	G
ENSRNOSNP2784364	J479605	168545661	A	G	G	G	G	G	A	G	A	A	A	A	A	A	A	A	A	A	-	G	G	G
ENSRNOSNP2784365	Cpn_1168569897	168569897	G	T	T	T	T	T	T	T	T	T	T	T	T	T	T	T	T	T	T	T	T	T
ENSRNOSNP2784366	rat105_039_o01.p1ca_283	168571574	C	T	T	T	T	T	T	T	T	T	T	T	T	T	T	T	T	T	T	T	T	T
ENSRNOSNP2784367	rat102_019_k23.p1ca_172	168627918	T	C	C	C	C	C	T	C	T	T	-	T	T	T	T	T	T	T	T	C	C	C
ENSRNOSNP2784368	J560584	168718003	T	C	C	C	C	C	C	C	C	C	C	C	C	C	C	C	C	C	-	C	C	C
ENSRNOSNP2784369	J583372	168863206	A	T	T	T	T	T	A	T	A	A	A	A	A	A	A	A	A	A	A	T	T	T
ENSRNOSNP2784370	SHRSPc04a10_r1_790	168954312	G	G	G	G	G	G	A	G	A	A	A	A	A	G/A	A	A	A	A	A	G	G	G
ENSRNOSNP2784371	J693431	169009512	T	T	T	T	T	T	T	T	T	T	T	T	T	T	T	T	T	T	-	T	T	T
ENSRNOSNP2784372	J879501	169106151	T	T	T	T	T	T	C	T	C	C	C	C	C	-	C	C	C	C	-	T	T	T
ENSRNOSNP2784373	gnl|ti|896438209_19866861444740_216	169207094	T	C	C	C	C	C	C	C	C	C	C	C	C	C	C	C	C	C	C	C	C	C
ENSRNOSNP2784374	J641280	169269375	C	A	A	A	A	A	A	A	A	A	A	A	A	A	A	A	A	A	A	A	A	A
ENSRNOSNP2784375	SHRSPc01g05_r1_574	169405055	C	C	C	C	C	C	T	C	T	T	T	T	T	T	T	T	T	T	T	C	C	C
ENSRNOSNP2784376	J687715	169498796	C	T	T	T	T	T	T	T	T	T	T	T	T	T	T	T	T	T	-	T	T	T
ENSRNOSNP2784377	rat109_045_l10.q1ca_390	169514160	C	G	G	G	G	G	G	G	G	G	G	G	G	G	G	G	G	G	G	G	G	G
ENSRNOSNP2784378	J539061	169617517	C	A	A	A	A	A	C	A	C	C	C	C	C	C	C	C	C	C	C	A	A	A
ENSRNOSNP2784379	rdahl-89h7_rp2_b1_225	169621713	T	C	C	C	C	C	T	C	T	T	T	T	T	T	T	T	T	T	T	C	C	C
ENSRNOSNP2784380	Cpn_1169919000	169919000	A	G	G	G	G	G	G	G	G	G	G	G	G	G	G	G	G	G	G	G	G	G
ENSRNOSNP2784381	J486372	169922059	G	T	T	T	T	T	T	T	T	T	T	T	T	T	T	T	T	T	T	T	T	T
ENSRNOSNP2784382	rat102_027_g05.p1ca_159	169926329	A	G	G	G	G	G	G	G	G	G	G	G	G	G	G	G	G	G	G	G	G	G
ENSRNOSNP2784383	WKYc17d06_r1_769	169995049	A	T	T	T	T	T	T	T	T	T	T	T	T	T	T	T	T	T	T	T	T	T
ENSRNOSNP2784384	rat013_042_h18.q1ca_209	170037305	C	C	C	C	C	C	C	C	C	C	C	C	C	C	C	C	C	C	C	C	C	C

ENSRNOSNP2784385	J339457	170059164	A	G	G	G	G	G	G	G	G	G	G	G	G	G	G	G	G	G	-	G	G	G
ENSRNOSNP2784386	rat104_017_n17.q1ca_148	170155100	A	T	T	T	T	T	A	T	A	A	A	A	A	A	A	A	A	A	A	T	T	T
ENSRNOSNP2784387	rat105_019_e07.q1ca_158	170215759	T	C	C	C	C	C	C	C	C	C	C	C	C	C	C	C	C	C	C	C	C	C
ENSRNOSNP2784388	J491809	170320387	A	T	T	T	T	T	T	T	T	T	T	T	T	T	T	T	T	T	T	T	T	T
ENSRNOSNP2784389	rat104_078_k11.q1ca_318	170387966	A	G	G	G	G	G	G	G	G	G	G	G	G	G	G	G	G	G	G	G	G	G
ENSRNOSNP2784390	rat104_041_g07.q1ca_568	170390397	T	A	A	A	A	A	A	A	A	A	A	A	A	A	A	A	A	A	A	A	A	A
ENSRNOSNP2784391	Cpn_1170390711	170390711	A	T	T	T	T	T	T	T	T	T	T	T	T	T	T	T	T	T	T	T	T	T
ENSRNOSNP2784392	J524207	170414581	G	T	T	T	T	T	T	T	T	T	T	T	T	T	T	T	T	T	-	T	T	T
ENSRNOSNP2784393	WKYc108e11_r1_248	170487421	C	T	T	T	T	T	T	T	T	T	T	T	T	T	T	T	T	T	T	T	T	T
ENSRNOSNP2784394	WKYOa15a01_s1_253	170540189	G	A	A	A	A	A	A	A	A	A	A	A	A	A	A	A	A	A	A	A	A	A
ENSRNOSNP2784395	J681433	170683132	T	T	T	T	T	T	C	T	C	C	C	C	C	C	C	C	C	C	C	T	T	T
ENSRNOSNP2784397	J493936	171031968	T<br>G	G	G	-	G	G	-	G	G	G	G	G	G	-	G	G	G	G	-	G	G	G
ENSRNOSNP2784398	gko-15d8_fp2_b1_203	171121005	C	C	C	C	C	C	C	C	C	C	C	C	C	C	C	C	C	C	C	T	T	T
ENSRNOSNP2784399	J544697	171122739	G	G	G	G	G	G	G	G	G	G	G	G	G	G	G	G	G	G	-	A	A	A
ENSRNOSNP2784400	J698287	171233490	C	C	C	C	C	C	T	C	T	T	T	T	T	T	T	T	T	T	-	C	C	C
ENSRNOSNP2784401	J693497	171339211	A	A	A	A	A	A	G	A	G	G	G	G	G	G	G	G	G	G	G	G	G	G
ENSRNOSNP2784402	WKYd03a03_s1_24	171350839	T	C	C	C	C	C	T	C	T	T	T	T	T	T	T	T	T	T	T	T	T	T
ENSRNOSNP2784403	SHRSPa19e09_r1_1104	171442718	C	C	C	C	C	C	T	C	T	T	T	T	T	T	T	T	T	T	T	C	C	C
ENSRNOSNP2784404	J525106	171714538	G	A	A	A	A	A	G	A	G	G	G	G	G	G	G	G	G	G	G	A	A	A
ENSRNOSNP2784405	J546199	171836884	G	G	G	G	G	G	G	G	G	G	G	G	G	G	G	G	G	G	-	A	A	A
ENSRNOSNP2784406	rdahl-50l22_fp2_b1_78	171896496	A	G	G	G	G	G	G	G	G	G	G	G	G	G	G	G	G	G	G	A	A	A
ENSRNOSNP2784407	J565259	171934152	G	T	T	T	T	T	T	T	T	T	T	T	T	T	T	T	T	T	-	T	T	T
ENSRNOSNP2784408	rat101_007_d20.q1ca_330	172020573	T	C	C	C	C	C	T	C	T	T	T	T	T	T	T	T	T	T	T	T	T	T
ENSRNOSNP2784409	Cpn_1172021329	172021329	T	C	C	C	C	C	C	C	C	C	C	C	C	-	C	C	C	C	C	C	C	C
ENSRNOSNP2784410	J496805	172099041	C	C	C	-	C	C	-	C	CT	C<br>T	CT	CT	CT	-	CT	C<br>T	CT	CT	-	CT	C<br>T	CT
ENSRNOSNP2784411	Cpn_1172199898	172199898	G	A	A	A	A	A	A	A	A	A	A	A	A	A	A	A	A	A	A	A	A	A
ENSRNOSNP2784412	rat013_038_a14.p1ca_244	172237945	A	G	G	G	G	G	G	G	G	G	G	G	G	G	G	G	G	G	G	G	G	G
ENSRNOSNP2784413	SHRSPa8d01_s1_545	172504303	T	A	A	A	A	A	A	A	A	A	A	A	A	A	A	A	A	A	A	A	A	A
ENSRNOSNP2784414	Cpn_1172577432	172577432	C	T	T	T	T	T	T	T	T	T	T	T	T	T	T	T	T	T	T	T	T	T
ENSRNOSNP2784415	J481917	172608002	A	G	G	G	G	G	G	G	G	G	G	G	G	G	G	G	G	G	-	G	G	G
ENSRNOSNP2784416	J1311061	172943805	C	T	T	T	T	T	C	T	C	C	C	C	C	C	C	C	C	C	C	C	C	C
ENSRNOSNP2784417	WKYOa16b12_s1_248	172998518	T	C	C	T	C	T	C	C	C	C	C	C	C	C	C	C	C	C	C	C	C	C
ENSRNOSNP2784418	gko-8j6_fp2_b1_537	173147475	A	G	G	G	G	G	G	G	G	G	G	G	G	G	G	G	G	-	G	G	G	G
ENSRNOSNP2784419	J667769	173261603	A	G	G	G	G	G	G	G	G	G	G	G	G	G	G	G	G	G	G	G	G	G
ENSRNOSNP2784420	J697407	173376457	G	A	A	G	A	G	A	A	A	A	A	A	A	A	A	A	A	A	-	A	A	A
ENSRNOSNP2784421	WKYe15d11_r1_436	173398184	A	G	G	G	G	G	G	G	G	G	G	G	G	G	G	G	G	G	G	G	G	G
ENSRNOSNP2784422	J873194	173478225	T	G	G	G	G	G	G	G	G	G	G	G	G	G	G	G	G	G	-	G	G	G
ENSRNOSNP2784423	gnl|ti|896858076_19866867464063_287	173583296	T	G	G	G	G	G	G	G	G	G	G	G	G	G	G	G	G	G	G	G	G	G
ENSRNOSNP2784424	J593426	173632653	G	G<br>A	G	-	G	-	-	G	-	G	G	G	-	-	G	G	G	G	-	A	A	A

ENSRNOSNP2784425	gko-63n3_fp2_b1_627	173785040	T	A	A	A	A	A	A	A	A	A	A	A	A	A	A	A	A	A	A	A	A	A
ENSRNOSNP2784426	J567536	174068994	G	G/A	G	-	G	A	-	-	A	A	A	A	A	-	A	A	A	A	-	A	A	A
ENSRNOSNP2784427	WKYd02e03_s1_144	174129878	T	A	A	A	A	A	A	A	A	A	A	A	A	A	A	A	A	A	A	T	T	T
ENSRNOSNP2784428	Cpn_1174184945	174184945	C	-	T	C	T	C	C	T	C	C	C	C	C	C	C	C	C	C	C	C	C	C
ENSRNOSNP2784429	J337196	174206410	C	T	T	T	T	T	T	T	T	T	T	T	T	T	T	T	T	T	-	C	C	C
ENSRNOSNP2784430	J684089	174391647	C	C	C	T	C	T	T	C	T	T	T	T	T	T	T	T	T	T	T	T	T	T
ENSRNOSNP2784431	SHRSPc44g03_r1_72	174412169	G	G	G	A	G	A	A	G	A	A	A	A	A	A	A	A	A	A	A	G	G	G
ENSRNOSNP2784432	rat102_026_b19.p1ca_405	174432513	C	T	T	C	T	C	C	T	C	C	C	C	C	C	C	C	C	C	C	C	C	C
ENSRNOSNP2784433	rat101_012_k12.p1ca_212	174485928	G	A	A	G	A	G	G	A	G	G	G	G	G	G	G	G	G	G	G	G	G	G
ENSRNOSNP2784434	WKYe16c06_r1_573	174592784	C	-	T	-	T	T	T	T	T	T	T	T	T	T	T	T	T	T	T	C	C	C
ENSRNOSNP2784435	rat108_002_a10.q1ca_399	174638055	A	G	G	G	G	G	G	G	G	G	G	G	G	G	G	G	G	G	G	G	G	G
ENSRNOSNP2784436	J595023	174705409	T	C	C	C	C	C	C	C	C	C	C	C	C	C	C	C	C	C	C	C	C	C
ENSRNOSNP2784437	SHRSPa33g10_r1_712	174804498	A	G	G	G	G	G	G	G	G	G	G	G	G	G	G	G	G	G	G	A	A	A
ENSRNOSNP2784438	J500060	174881987	A	A	A	A	A	A	A	A	A	A	A	A	A	A	A	A	A	A	-	G	G	G
ENSRNOSNP2784439	J702083	175088031	T	C	C	C	C	C	C	C	C	C	C	C	C	C	C	C	C	C	C	C	C	C
ENSRNOSNP2784440	J540152	175186734	A	A	A	A	A	A	A	A	A	A	A	A	A	A	A	A	A	A	-	C	C	C
ENSRNOSNP2784441	WKYc110e11_s1_429	175233123	T	C	C	C	C	C	C	C	C	C	C	C	C	C	C	C	C	C	C	T	T	T
ENSRNOSNP2784442	J881815	175283820	A	T	T	A	T	A	A	T	A	A	A	A	A	A	A	A	A	A	-	T	T	T
ENSRNOSNP2784443	J636104	175416545	C	T	T	C	T	C	C	T	C	C	C	C	C	C	C	C	C	C	C	T	T	T
ENSRNOSNP2784444	J565017	175521125	G	G	G	A	G	A	A	G	A	A	A	A	A	A	A	A	A	A	-	A	A	A
ENSRNOSNP2784445	SHRSPa57e07_s1_198	175536967	C	C	C	G	C	G	G	C	G	G	G	G	G	G	G	G	G	G	G	G	G	G
ENSRNOSNP2784446	J647068	175612854	A	A	A	A	A	A	A	A	A	A	A	A	A	A	A	A	A	A	-	A	A	A
ENSRNOSNP2784447	WKYc04b07_s1_668	175619519	A	C	C	C	C	C	C	C	C	C	C	C	C	C	C	C	C	C	C	C	C	C
ENSRNOSNP2784448	WKY-G-i-08c04_r1_308	175677908	A	G	G	G	G	G	G	G	G	G	G	G	G	G	G	G	G	G	G	A	A	A
ENSRNOSNP2784449	J880086	175783549	A	G	G	G	G	G	G	G	G	G	G	G	G	G	G	G	G	G	G	A	A	A
ENSRNOSNP2784450	rat105_010_i07.p1ca_311	175807317	C	T	T	C	T	C	C	T	C	C	C	C	C	C	C	C	C	C	C	C	C	C
ENSRNOSNP2784451	SHRSPc17c12_r1_414	175944948	C	C	C	A	C	A	A	C	A	A	A	A	A	A	A	A	A	A	A	A	A	A
ENSRNOSNP2784452	J551036	176182891	A/G	G	G	-	G	G	-	G	G	G	G	G	G	-	G	G	G	G	-	G	G	G
ENSRNOSNP2784453	WKYc22a06_s1_235	176403842	T	C	C	C	-	C	C	C	C	C	C	C	C	C	C	C	C	C	C	C	C	C
ENSRNOSNP2784454	J596828	176474058	T	C	C	C	C	C	C	C	C	C	C	C	C	C	C	C	C	C	-	C	C	C
ENSRNOSNP2784455	WKY-G-i-52a11_f1_1190	176588782	C	T	T	T	T	T	T	T	T	T	T	T	T	T	T	T	T	T	T	T	T	T
ENSRNOSNP2784456	J552234	176699623	A	A	A	A	A	A	A	A	A	A	A	A	A	A	A	A	A	A	-	G	G	G
ENSRNOSNP2784457	gnl|ti|896748103_19866867109270_371	176761645	G	G	G	G	G	G	G	G	G	G	G	G	G	-	G	G	G	G	G	G	G	G
ENSRNOSNP2784458	J477923	176841732	G	G	G	G	G	G	G	G	G	G	G	G	G	G	G	G	G	G	-	A	A	A
ENSRNOSNP2784459	rat105_037_g22.p1ca_351	176960454	G	G	G	T	G	T	T	G	T	T	T	T	T	T	T	T	T	T	T	T	T	T
ENSRNOSNP2784460	J880546	177071931	A	A	A	G	A	G	G	A	G	G	G	G	G	G	G	G	G	G	-	G	G	G
ENSRNOSNP2784461	Cpn_1177207742	177207742	G	G	G	G	G	G	G	G	G	G	G	G	G	G	G	G	G	G	G	G	G	G
ENSRNOSNP2784462	J527102	177221553	T	T	T	T	T	T	T	T	T	T	T	T	T	T	T	T	T	T	-	C	C	C
ENSRNOSNP2784464	gko-66d20_fp2_b1_68	177388037	T	C	C	C	C	C	C	C	C	C	C	C	C	C	C	C	C	C	C	C	C	C

ENSRNOSNP2784465	J658570	177498939	G	A	A	G	A	G	G	A	G	G	G	G	G	G	G	G	G	G	-	A	A	A
ENSRNOSNP2784466	J501732	177641595	A	A<br>G	G	-	G	-	-	G	-	G	G	G	-	-	-	G	G	-	-	A<br>G	G	G
ENSRNOSNP2784467	rat104_063_f19.q1ca_239	177710051	T	C	C	C	C	C	C	C	C	C	C	C	C	C	C	C	C	C	C	T	T	T
ENSRNOSNP2784468	rat104_016_i10.q1ca_233	177729282	A	A	T	T	T	T	T	T	T	T	T	T	T	T	T	T	T	T	T	A	A	A
ENSRNOSNP2784469	rat110_012_k07.p1ca_206	177742486	A	A	G	G	G	G	G	G	G	G	G	G	G	G	G	G	G	G	G	A	A	A
ENSRNOSNP2784470	rat103_009_n14.p1ca_367	177787510	G	A	A	A	A	A	A	A	A	A	A	A	A	A	A	A	A	A	A	G	G	G
ENSRNOSNP2784471	rat104_035_i20.q1ca_278	177808278	T	C	C	C	C	C	C	C	C	C	C	C	C	C	C	C	C	C	C	T	T	T
ENSRNOSNP2784472	rat110_009_b22.p1cb_209	177832111	A	T	T	T	T	T	T	T	T	T	T	T	T	T	T	T	T	T	T	A	A	A
ENSRNOSNP2784473	rat110_004_a12.p1ca_181	177843913	A	G	G	G	G	G	G	G	G	G	G	G	G	G	G	G	G	G	G	A	A	A
ENSRNOSNP2784474	rat110_003_l14.p1ca_603	177868706	A	T	A	A	A	A	T	A	T	T	T	T	A	T	T	T	T	T	T	A	A	A
ENSRNOSNP2784475	J658758	177950683	G	A	A	-	A	A	-	A	A	A	A	A	A	-	A	A	A	A	-	-	G	G
ENSRNOSNP2784476	J651074	178186474	A	G	G	-	G	G	-	G	G	G	G	G	G	-	G	G	G	G	-	A	A	A
ENSRNOSNP2784477	J1268611	179037626	C	T	T	T	T	T	T	T	T	T	T	T	T	T	T	T	T	T	-	T	T	T
ENSRNOSNP2784478	J342027	179193258	A	G	G	G	G	G	G	G	G	G	G	G	G	G	G	G	G	G	-	A	A	A
ENSRNOSNP2784479	gko-6d2_rp2_b1_54	179327802	C	C	C	C	C	C	C	C	C	C	C	-	C	C	C	C	C	C	C	T	T	T
ENSRNOSNP2784480	J641586	179745834	A	A<br>G	G	-	G	A<br>G	-	A<br>G	A<br>G	G	G	G	A<br>G	-	A<br>G	G	A<br>G	A<br>G	-	A<br>G	G	G
ENSRNOSNP2784481	WKYa70e11_s1_159	179862388	C	T	T	T	T	T	T	T	T	T	T	T	T	T	T	T	-	T	T	T	T	T
ENSRNOSNP2784482	J337681	179969065	G	A	A	A	A	A	A	A	A	A	A	A	A	A	A	A	A	A	-	A	A	A
ENSRNOSNP2784483	WKYc17d09_r1_311	180111057	A	A	T	T	T	T	A	T	A	A	A	A	T	A	A	A	A	A	A	A	A	A
ENSRNOSNP2784484	J520374	180115236	C	C	C	C	C	C	C	C	C	C	C	C	C	C	C	C	C	C	C	T	T	T
ENSRNOSNP2784485	SHRSPc39f12_s1_515	180177547	G	A	G	G	G	G	A	G	A	A	A	A	G	A	A	A	A	A	A	G	G	G
ENSRNOSNP2784486	J677340	180265854	A	G	G	G	G	G	G	G	G	G	G	G	G	G	G	G	G	G	-	G	G	G
ENSRNOSNP2784487	gnl|ti|896653520_19866868031586_293	180447327	C	T	C	C	C	C	T	C	T	T	T	T	C	T	T	T	T	T	T	C	C	C
ENSRNOSNP2784488	rat110_026_a05.p1ca_576	180563088	A	G	G	G	G	G	G	G	G	G	G	G	G	G	G	G	G	G	G	G	G	G
ENSRNOSNP2784489	J1275243	180564598	G	G	A	A	A	A	G	A	G	G	G	G	A	G	G	G	G	G	G	G	G	G
ENSRNOSNP2784490	J524358	180794675	A	T	T	T	T	T	T	T	T	T	T	T	T	T	T	T	T	T	-	T	T	T
ENSRNOSNP2784491	WKYc05b11_r1_249	180822160	C	C	T	T	T	T	C	T	C	C	C	C	T	C	C	C	C	C	C	C	C	C
ENSRNOSNP2784492	J475703	180945746	T	C	C	C	C	C	C	C	C	C	C	C	C	C	C	C	C	C	C	C	C	TC
ENSRNOSNP2784493	Cpn_1181012647	181012647	C	T	T	T	T	T	T	T	T	T	T	T	T	T	T	T	T	T	T	T	T	T
ENSRNOSNP2784494	rat104_071_p19.q1cb_328	181134479	G	G	A	G	A	G	G	-	G	G	G	G	G	G	G	G	G	G	G	G	G	G
ENSRNOSNP2784495	SHRSPc44h01_s1_152	181287248	C	G	C	C	C	C	G	C	G	G	G	G	C	G	G	G	G	G	G	C	C	C
ENSRNOSNP2784496	J681483	181346420	G	G	G	G	G	G	G	G	G	G	G	G	G	G	G	G	G	G	G	A	A	A
ENSRNOSNP2784497	rat101_002_n17.p1ca_220	181456824	A	A	A	A	A	A	A	A	A	A	A	A	A	A	A	A	A	A	A	G	G	G
ENSRNOSNP2784498	SHRSPc48b07_r1_494	181494720	T	T	T	T	T	T	T	T	T	T	T	T	T	T	T	T	T	T	T	C	C	C
ENSRNOSNP2784499	J342907	181648511	A	C	C	C	C	C	C	C	C	C	C	C	C	C	C	C	C	C	C	A	A	A
ENSRNOSNP2784500	SHRSPa88h04_r1_739	181758850	A	G	-	-	-	-	G	-	G	G	G	G	-	G	G	G	G	G	G	A	A	A
ENSRNOSNP2784501	Cpn_1181818332	181818332	G	G	G	G	G	G	G<br>C	G	G	G	G	G	G	G	G	G	G	G	G	G	G	G
ENSRNOSNP2784502	SHRSPa77h07_r1_728	181894869	A	A	A	A	A	A	A	A	A	A	A	A	A	A	-	A	A	A	-	A	-	-
ENSRNOSNP2784504	gnl|ti|896632804_19866867268301_330	182078913	T	C	C	C	C	C	C	C	C	C	C	C	C	C	C	C	C	C	C	T	T	T

ENSRNOSNP2784505	rat105_024_a03.q1ca_182	182133563	C	T	T	T	T	T	T	T	T	T	T	T	T	T	T	T	T	T	T	C	C	C
ENSRNOSNP2784507	J536664	182256273	G	G	G	G	G	G	G	G	G	G	G	G	G	G	G	G	G	G	-	G	G	G
ENSRNOSNP2784508	Cpn_1182298109	182298109	C	G	C	C	C	C	G	C	G	G	G	G	C	G	G	G	G	G	G	C	C	C
ENSRNOSNP2784509	J696930	182379407	T	A	T	T	T	T	A	T	A	A	A	A	T	A	A	A	A	A	A	T	T	T
ENSRNOSNP2784510	J648013	182535432	C	C	C	C	C	C	C	C	C	C	C	C	C	C	C	C	C	C	-	C	C	C
ENSRNOSNP2784511	gko-41g16_rp2_b1_39	182647806	C	T	C	C	C	C	T	C	T	T	T	T	C	T	T	T	T	T	T	C	C	C
ENSRNOSNP2784512	J549179	182725298	C	C	C	C	C	C	C	C	C	C	C	C	C	C	C	C	C	C	C	C	C	C
ENSRNOSNP2784513	WKY-G-i-27h03_f1_175	182868662	C	C	C	C	C	C	C	C	C	C	C	C	C	C	C	C	C	C	C	T	T	T
ENSRNOSNP2784514	gnl|ti|897048428_19866867478919_368	183065176	C	T	T	T	T	T	C	T	C	C	C	C	T	C	C	C	C	C	C	C	C	C
ENSRNOSNP2784515	SHRSPa17e11_s1_471	183683647	A	T	T	T	T	T	T	T	T	T	T	T	T	T	T	T	T	T	T	A	A	A
ENSRNOSNP2784516	J634672	183900353	T	G	G	G	G	G	G	G	G	G	G	G	G	G	G	G	G	G	G	T	T	T
ENSRNOSNP2784517	SHRSPc41g07_r1_426	184360555	C	-	T	T	T	T	T	T	T	T	T	T	T	T	T	T	T	T	T	C	C	C
ENSRNOSNP2784518	J685120	184391827	G	T	T	T	T	T	T	T	T	T	T	T	T	T	T	T	T	T	T	G	G	G
ENSRNOSNP2784519	J660716	184517563	T	C	C	C	C	C	C	C	C	C	C	C	C	C	C	C	C	C	-	T	T	T
ENSRNOSNP2784520	rat104_006_b07.q1ca_390	184629593	T	A	A	A	A	A	A	A	A	A	A	A	A	A	A	A	A	A	A	T	T	T
ENSRNOSNP2784521	DS-g-c-15b01_f1_315	184681859	A	G	G	G	G	G	G	G	G	G	G	G	G	G	G	G	G	G	G	A	A	A
ENSRNOSNP2784522	rat104_043_h23.q1ca_244	184702022	G	A	A	A	A	A	G	A	G	G	G	G	A	G	G	G	G	G	G	G	G	G
ENSRNOSNP2784523	J649513	184874718	A	G	G	G	G	G	G	G	G	G	G	G	G	G	G	G	G	G	-	A	A	A
ENSRNOSNP2784524	Cpn_1184935644	184935644	G	C	C	C	C	C	C	C	C	C	C	C	C	C	C	C	C	-	C	G	G	G
ENSRNOSNP2784525	WKYc33g07_s1_244	184995554	G	T	T	T	T	T	G	T	G	G	G	G	T	-	G	G	G	G	G	G	G	G
ENSRNOSNP2784526	J681607	185195584	C	C	C	C	C	C	T	C	T	T	T	T	C	T	T	T	T	T	-	C	C	C
ENSRNOSNP2784527	J339456	185299237	A	G	G	G	G	G	G	G	G	G	G	G	G	G	G	G	G	G	G	A	A	A
ENSRNOSNP2784528	gko-55l3_rp2_b1_77	185341704	C	A	A	A	A	A	C	A	C	C	C	C	A	C	C	C	C	C	C	A	A	A
ENSRNOSNP2784529	J494466	185413648	G	G	G	G	G	G	G	G	G	G	G	G	G	G	G	G	G	G	-	A	A	A
ENSRNOSNP2784530	J560889	185541630	G	G	G	G	G	G	A	G	A	A	A	A	G	A	A	A	A	A	-	A	A	A
ENSRNOSNP2784531	rat104_061_l16.q1ca_474	185546033	T	C	C	C	C	C	C	C	C	C	C	C	C	C	C	C	C	C	C	C	C	C
ENSRNOSNP2784532	Cpn_1185633136	185633136	C	C	C	C	C	C	T	C	T	T	T	T	C	T	T	T	T	T	T	T	T	T
ENSRNOSNP2784533	rat101_017_b19.p1cb_362	185692567	T	T	T	T	T	T	T	T	T	T	T	T	T	T	T	T	T	T	T	T	T	T
ENSRNOSNP2784534	J507596	185762321	A	A	A	A	A	A	A	A	A	A	A	A	A	A	A	A	A	A	A	G	G	G
ENSRNOSNP2784535	rat101_017_a06.q1ca_391	185853325	T	C	C	C	C	C	C	C	C	C	C	C	C	C	C	C	C	C	C	T	T	T
ENSRNOSNP2784536	J877779	185950950	G	G	G	G	G	G	G	G	A	G	G	G	G	G	A	A	A	A	-	G	G	G
ENSRNOSNP2784537	WKYOa28f12_s1_242	186055581	T	C	C	C	C	C	C	C	T	C	C	C	C	C	T	T	T	T	T	T	T	T
ENSRNOSNP2784538	rat104_054_g07.q1cb_456	186165480	C	G	G	G	G	G	G	G	C	G	G	G	G	G	C	C	C	C	C	C	C	C
ENSRNOSNP2784539	J549925	186168275	A	A	A	A	A	A	A	A	G	A	A	A	A	A	G	G	G	G	G	G	G	G
ENSRNOSNP2784541	J877866	186368264	C	C	C	C	C	C	C	C	G	C	C	C	C	C	G	G	G	G	-	C	C	C
ENSRNOSNP2784542	rdahl-101f23_fp2_b1_279	186370915	A	-	A	A	A	A	A	A	G	A	A	A	A	A	G	G	G	G	G	A	A	A
ENSRNOSNP2784543	J551745	186529653	T	T	T	T	T	T	T	T	T	T	T	T	T	T	T	T	T	T	T	C	C	C
ENSRNOSNP2784544	Cpn_1186535990	186535990	A	A	A	A	A	A	A	A	T	A	A	A	A	A	T	T	T	T	T	A	A	A
ENSRNOSNP2784545	rat104_064_e09.q1ca_133	186684588	G	T	T	T	T	T	T	T	G	T	T	T	T	T	G	G	G	G	G	T	T	T

ENSRNOSNP2784546	J536800	186857909	C	CT	C	-	C	C	-	C	C	C	C	C	C	-	C	C	C	C	-	T	T	T
ENSRNOSNP2784547	Cpn_1186870514	186870514	A	G	G	G	G	G	G	G	A	G	G	G	G	G	A	A	A	A	A	G	G	G
ENSRNOSNP2784548	J572319	186956187	A	A	A	-	A	A	-	A	A	A	A	A	A	-	A	A	A	A	-	C	C	C
ENSRNOSNP2784549	rat104_003_f11.p1ca_191	187059047	C	T	T	T	T	T	T	T	C	T	T	T	T	T	C	C	C	C	C	C	C	C
ENSRNOSNP2784550	rat103_032_l17.q1ca_312	187060174	G	T	T	T	T	T	T	T	G	T	T	T	T	T	G	G	G	G	G	T	T	T
ENSRNOSNP2784551	Cpn_1187203527	187203527	T	G	G	G	G	G	G	G	T	G	G	G	G	G	T	T	T	T	T	G	G	G
ENSRNOSNP2784552	J574424	187204804	A	A G	G	-	G	A G	-	A G	A	G	G	G	-	-	A	A	A	A	-	A G	G	G
ENSRNOSNP2784553	rat101_025_i11.p1ca_350	187207578	G	A	A	A	A	A	A	A	G	A	A	A	A	A	G	G	G	G	G	A	A	A
ENSRNOSNP2784554	rat106_034_h09.q1ca_609	187295452	A	A	A	A	A	A	A	A	A	A	A	A	A	A	A	A	A	A	A	G	G	G
ENSRNOSNP2784555	J526562	187309571	A	A	A	A	A	A	A	A	A	A	A	A	A	A	A	A	A	A	-	G	G	G
ENSRNOSNP2784556	J526181	187414420	T	T	T	-	T	T	-	T	T	T	T	T	T	-	T	T	T	T	-	A	A	A
ENSRNOSNP2784557	Cpn_1187528619	187528619	T	C	C	C	C	C	C	C	T	C	C	C	C	C	T	T	T	T	T	T	T	T
ENSRNOSNP2784558	J639601	187662982	C	-	C	-	C	-	-	-	-	C	C	C	-	-	-	C	-	-	-	C A	C	C
ENSRNOSNP2784559	gnl|ti|897019368_19866868274491_285	187787779	A	G	G	G	G	G	G	G	A	G	G	G	G	G	A	A	A	A	A	G	G	G
ENSRNOSNP2784560	rat102_007_h11.q1ca_371	187804556	T	C	C	C	C	C	C	C	T	C	C	C	C	C	T	T	T	T	T	C	C	C
ENSRNOSNP2784561	J504572	187807636	A	A	A	A	A	A	A	A	A	A	A	A	A	A	A	A	A	A	-	G	G	G
ENSRNOSNP2784562	rat106_027_h07.p1ca_460	187915683	A	G	G	G	G	G	G	G	A	G	G	G	G	G	A	A	A	A	A	G	G	G
ENSRNOSNP2784563	rat101_019_p15.p1ca_656	187947190	T	C	C	C	C	C	C	C	T	C	C	C	C	C	T	T	T	T	T	C	C	C
ENSRNOSNP2784564	Cpn_1187978763	187978763	C	A	A	A	A	A	A	A	C	A	A	A	A	A	C	C	C	C	C	A	A	A
ENSRNOSNP2784565	WKYc56g04_s1_313	187980634	T	C	C	C	C	C	C	C	T	C	C	C	C	C	T	T	T	T	T	C	C	C
ENSRNOSNP2784566	J591687	188030859	T	T	T	T	T	T	T	T	T	T	T	T	T	T	T	T	T	T	-	C	C	C
ENSRNOSNP2784567	gnl|ti|896850846_19866867431310_275	188137496	A	T	T	T	T	T	T	T	A	T	T	T	T	T	A	A	A	A	A	T	T	T
ENSRNOSNP2784568	J871949	188285859	T	C	C	C	C	C	C	C	T	C	C	C	C	C	T	T	T	T	T	C	C	C
ENSRNOSNP2784569	rdahl-29h7_fp2_b1_239	188312379	G	G	G	G	G	G	G	G	G	G	G	G	G	G	G	G	G	G	G	G	G	G
ENSRNOSNP2784570	J558962	188389740	G	C	C	C	C	C	C	C	G	C	C	C	C	C	G	G	G	G	-	C	C	C
ENSRNOSNP2784571	rdahl-63l3_rp2_b1_413	188464430	T	C	C	C	C	C	C	C	T	C	C	C	C	C	T	T	T	T	T	C	C	C
ENSRNOSNP2784572	J587168	188521616	T	T	T	-	T	T	-	T	T	T	T	T	T	-	T	T	T	T	-	C	C	C
ENSRNOSNP2784573	rat108_010_n21.q1cb_261	188643674	T	C	C	C	C	C	C	C	T	C	C	C	C	C	C	C	C	C	C	T	T	T
ENSRNOSNP2784574	J502912	188720091	A	A	A	A	A	A	A	A	A	A	A	A	A	A	A	A	A	A	-	G	G	G
ENSRNOSNP2784575	rdahl-81l4_fp2_b1_202	188781797	G	G	G	G	G	G	G	G	G	G	G	G	G	G	G	G	G	G	G	G	G	G
ENSRNOSNP2784576	J545456	188833745	G	G	G	G	G	G	G	G	G	G	G	G	G	G	G	G	G	G	-	A	A	A
ENSRNOSNP2784577	rat107_023_l16.p1ca_402	188895283	C	C	C	C	C	C	C	C	C	C	C	C	C	C	C	C	C	C	C	C	C	C
ENSRNOSNP2784578	J679026	188958949	C	C	C	C	C	C	C	C	C	C	C	C	C	C	C	C	C	C	C	C	C	C
ENSRNOSNP2784579	WKYc42e08_s1_486	189086998	C	T	T	T	T	T	T	T	C	T	T	T	T	T	T	T	T	T	T	T	T	T
ENSRNOSNP2784580	J1271983	189147923	C	A	A	A	A	A	A	A	C	A	A	A	A	A	A	A	A	A	-	A	A	A
ENSRNOSNP2784581	gnl|ti|896576983_19866868286697_276	189396078	T	C	C	C	-	C	C	C	T	C	C	C	C	C	C	-	C	C	C	T	T	T
ENSRNOSNP2784582	WKYc94h08_s1_413	189625567	G	A	A	A	A	A	A	A	G	A	A	A	A	A	A	A	A	A	A	G	G	G
ENSRNOSNP2784583	J672981	189713200	C	CT	C	-	C	-	-	-	-	C	C	C	-	-	-	C	-	-	-	CT	C	C
ENSRNOSNP2784584	rdahl-1o18_rp2_b1_43	189900345	G	G	G	G	G	G	G	G	G	G	G	G	G	G	G	G	G	G	G	G	G	G

ENSRNOSNP2784585	rat109_045_g06.q1ca_639	189924824	G	T	T	T	T	T	T	T	G	T	T	T	T	T	T	T	T	T	T	G	G	G
ENSRNOSNP2784586	Cpn_1190079641	190079641	C	T	T	T	T	T	T	T	C	T	T	T	T	T	T	T	T	T	T	C	C	C
ENSRNOSNP2784588	WKYc36d10_s1_327	190281302	T	C	C	C	C	C	C	C	T	C	C	C	C	C	C	C	C	C	C	T	T	T
ENSRNOSNP2784589	J1265920	190335449	A	G	G	G	G	G	G	G	A	G	G	G	G	G	G	G	G	G	-	A	A	A
ENSRNOSNP2784590	J339481	191124400	C	T	T	T	T	T	T	T	C	T	T	T	T	T	T	T	T	T	T	C	C	C
ENSRNOSNP2784591	rat108_007_a02.q1cb_498	191139811	C	T	T	T	T	T	T	T	C	T	T	T	T	T	T	T	T	T	T	C	C	C
ENSRNOSNP2784592	J700198	191233659	T	T	T	T	T	T	T	T	T	T	T	T	T	T	T	T	T	T	-	T	T	T
ENSRNOSNP2784593	WKYa71f05_s1_175	191240400	A	G	G	G	G	G	G	G	A	G	G	G	G	G	G	G	G	G	G	A	A	A
ENSRNOSNP2784594	J337899	191477586	C	T	T	T	T	T	T	T	C	T	T	T	T	T	T	T	T	T	T	C	C	C
ENSRNOSNP2784595	Cpn_1191511911	191511911	A	G	G	G	G	G	G	G	A	G	G	G	G	G	G	G	G	G	G	G	G	G
ENSRNOSNP2784596	J336735	191643847	G	T	T	T	T	T	T	T	G	T	T	T	T	T	T	T	T	T	-	G	G	G
ENSRNOSNP2784597	J636872	191818813	G	G	G	G	G	G	G	G	G	G	G	G	G	G	G	G	G	G	G	G	G	G
ENSRNOSNP2784598	rdahl-57e18_fp2_b1_350	191854465	T	T	T	T	T	T	T	T	T	T	T	T	T	T	T	T	T	T	T	C	C	C
ENSRNOSNP2784599	Cpn_1192177195	192027314	A	G	G	G	G	G	G	G	A	G	G	G	G	G	G	G	G	G	G	A	A	A
ENSRNOSNP2784600	gko-99p5_rp2_b1_273	192548657	A	A	A	A	A	A	A	A	A	A	A	A	A	A	A	A	A	A	A	G	G	G
ENSRNOSNP2784601	gko-105l8_fp2_b1_438	192700265	G	G	G	G	G	G	G	G	G	G	G	G	G	G	G	G	G	G	G	A	A	A
ENSRNOSNP2784602	SHRSPa25b10_s1_297	193106681	T	C	C	C	C	C	C	C	C	C	C	C	C	C	C	C	C	C	C	T	T	T
ENSRNOSNP2784603	DS-g-a-40h12_f1_36	193174911	G	A	A	A	A	A	A	A	A	A	A	A	A	A	A	A	A	A	A	A	A	A
ENSRNOSNP2784604	Cpn_1193218073	193218073	A	A	A	A	A	A	A	A	A	A	A	A	A	A	A	A	A	A	A	A	A	A
ENSRNOSNP2784605	rat108_018_c13.p1ca_659	193221647	T	C	C	C	C	C	C	C	C	C	C	C	C	C	C	C	C	C	C	T	T	T
ENSRNOSNP2784606	WKYc48c06_s1_510	193248816	G	A	A	A	A	A	-	A	A	A	-	A	A	A	A	A	A	A	A	G	G	G
ENSRNOSNP2784607	J530958	193271919	G	G	G	G	G	G	G	G	G	G	G	G	G	G	G	G	G	G	-	A	A	A
ENSRNOSNP2784608	J342520	193363363	A	G	G	G	G	G	G	G	G	G	G	G	G	G	G	G	G	G	-	G	G	G
ENSRNOSNP2784609	SHRSPa92d07_r1_562	193421868	T	C	C	C	C	C	C	C	C	C	C	C	C	C	C	C	C	C	C	T	T	T
ENSRNOSNP2784610	WKY-G-i-06b06_r1_435	193659821	G	A	A	A	A	A	A	A	A	A	A	A	A	A	A	A	A	A	A	G	G	G
ENSRNOSNP2784611	J638588	193790344	C	G	G	G	G	G	G	G	G	G	G	G	G	G	G	G	G	G	-	C	C	C
ENSRNOSNP2784612	rat102_037_n14.q1ca_449	194003410	C	T	T	T	T	T	T	T	C	T	T	T	T	T	T	T	T	T	T	C	C	C
ENSRNOSNP2784613	rat102_037_n14.q1ca_296	194003563	A	G	G	G	G	G	G	G	A	G	G	G	G	G	G	G	G	G	G	A	A	A
ENSRNOSNP2784614	J660444	194026392	C	T	T	T	T	T	T	T	-	T	T	T	T	T	T	T	T	T	T	-	C	C
ENSRNOSNP2784615	DS-g-a-18a01_f1_367	194127234	G	G	G	G	G	G	G	G	G	G	G	G	G	G	G	G	G	G	G	G	G	G
ENSRNOSNP2784616	gnl|ti|896732648_19866867002799_277	194505012	C	C	C	C	C	C	C	C	C	C	C	C	C	C	C	C	C	C	C	C	C	C
ENSRNOSNP2784617	J645768	194668536	C	C	C	C	C	C	C	C	C	C	C	C	C	C	C	C	C	C	C	C	C	C
ENSRNOSNP2784618	gnl|ti|896949457_19866867454870_329	194673191	A	A	A	A	A	A	A	A	A	A	A	A	A	A	A	A	A	A	A	A	A	A
ENSRNOSNP2784619	J567166	194778386	A	A	A	A	A	A	A	A	A	A	A	A	A	A	A	A	A	A	-	C	C	C
ENSRNOSNP2784620	J1271754	195088727	G	A	A	-	A	A	-	A	-	A	A	A	A	-	A	A	A	A	-	G<br>A	G	G
ENSRNOSNP2784621	Cpn_1195132894	195132894	T	C	C	C	C	C	C	C	C	C	C	C	C	C	C	C	C	C	C	T	T	T
ENSRNOSNP2784622	J1296994	195200656	A	G	G	G	G	G	G	G	-	G	G	G	G	G	G	G	G	G	-	G	G	G
ENSRNOSNP2784623	gnl|ti|896916708_19866868309411_282	195273115	C	-	C	-	C	C	C	C	C	C	-	C	C	C	C	C	C	C	C	C	C	C
ENSRNOSNP2784624	J527132	195298379	G	A	A	A	A	A	A	A	G	A	A	A	A	A	A	A	A	A	-	A	A	A

ENSRNOSNP2784625	J343142	195533574	A	C	C	C	C	C	C	C	A	C	C	C	C	C	C	C	C	C	C	C	C	C
ENSRNOSNP2784626	rdahl-75h4_fp2_b1_223	195619861	G	G	G	G	G	G	G	G	G	G	G	G	G	G	G	G	G	G	G	G	G	G
ENSRNOSNP2784627	J1309394	195679003	C	T	T	T	T	T	T	T	C	T	T	T	T	T	T	T	T	T	-	T	T	T
ENSRNOSNP2784628	gko-23j19_fp2_b1_75	195771373	T	C	C	C	C	C	C	C	T	C	C	C	C	C	C	C	C	C	C	C	C	C
ENSRNOSNP2784629	J681266	195848351	G	G<br>A	G	-	G	-	-	-	-	G	G	G	-	-	-	G	-	-	-	G<br>A	G	G
ENSRNOSNP2784630	J676072	195944183	C	G	G	G	G	G	G	G	C	G	G	G	G	G	G	G	G	G	-	G	G	G
ENSRNOSNP2784631	J568184	196158202	G	A	A	A	A	A	A	A	A	A	A	A	A	A	A	A	A	A	A	A	A	A
ENSRNOSNP2784632	SHRSPc76b12_r2_867	196176285	C	T	T	T	T	T	T	T	T	T	T	T	T	T	T	T	T	T	T	T	T	T
ENSRNOSNP2784633	SHRSPa02d04_r1_987	196204823	A	G	G	G	G	G	G	G	G	G	G	G	G	G	G	G	G	G	G	G	G	G
ENSRNOSNP2784634	J685461	196252307	C	C	C	C	C	C	C	C	C	C	C	C	C	C	C	C	C	C	-	C	C	C
ENSRNOSNP2784635	gko-23d18_fp2_b1_55	196391719	G	A	A	-	A	A	A	A	G	A	A	A	A	A	A	A	A	A	A	A	A	A
ENSRNOSNP2784636	J549849	196479223	T	C	C	C	C	C	C	C	C	C	C	C	C	C	C	C	C	C	C	C	C	C
ENSRNOSNP2784637	rdahl-62k20_fp2_b1_46	196572137	A	G	G	G	G	G	G	G	G	G	G	G	G	G	G	G	G	G	G	G	G	G
ENSRNOSNP2784638	gko-43g21_rp2_b1_49	196789613	C	T	T	T	T	T	T	T	C	T	T	T	T	T	T	T	T	T	T	T	T	T
ENSRNOSNP2784639	J478902	197208770	T	C	C	C	C	C	C	C	C	C	C	C	C	C	C	C	C	C	-	C	C	C
ENSRNOSNP2784640	DahlSc01a05_r1_244	197212851	T	C	C	C	C	C	C	C	T	C	C	C	C	C	C	C	C	C	C	C	C	C
ENSRNOSNP2784641	J682065	197343185	G	A	A	A	A	A	A	A	A	A	A	A	A	A	A	A	A	A	A	A	A	A
ENSRNOSNP2784642	SHRSPc36c10_r1_149	197492099	G	C	C	C	C	C	C	C	G	C	C	C	C	C	C	C	C	C	C	C	C	C
ENSRNOSNP2784643	SHRSPa12c06_r1_419	197533634	G	-	A	A	A	A	A	A	G	A	A	A	A	A	A	A	A	A	A	A	A	A
ENSRNOSNP2784644	J1266568	197550461	C	T	T	T	T	T	T	T	C	T	T	T	T	T	T	T	T	T	-	T	T	T
ENSRNOSNP2784645	J484663	197700672	C	T	T	T	T	T	T	T	T	T	T	T	T	T	T	T	T	T	T	T	T	T
ENSRNOSNP2784646	WKYOa44f11_s1_209	197762084	C	T	T	T	T	T	T	T	C	T	T	T	T	T	T	T	T	T	T	T	T	T
ENSRNOSNP2784647	J535903	197823092	A	C	C	C	C	C	C	C	C	C	C	C	C	C	C	C	C	C	-	C	C	C
ENSRNOSNP2784648	WKYc100g01_s1_575	197862317	A	C	C	C	C	C	C	C	C	C	C	C	C	C	C	C	C	C	-	C	C	C
ENSRNOSNP2784649	SHRSPc48f12_r1_504	197922583	A	G	G	G	G	G	G	G	G	G	G	G	G	G	G	G	G	G	G	G	G	G
ENSRNOSNP2784651	WKYc70e12_s1_793	198161874	C	T	T	T	T	T	T	T	C	T	T	T	T	T	T	T	T	T	T	T	T	T
ENSRNOSNP2784652	J548270	198174661	C	T	T	T	T	T	T	T	C	T	T	T	T	T	T	T	T	T	-	T	T	T
ENSRNOSNP2784653	J480983	198290840	A	G	G	G	G	G	G	G	A	G	G	G	G	G	G	G	G	G	-	G	G	G
ENSRNOSNP2784654	gnl|ti|897021218_19866868014633_288	198455269	G	G	G	G	G	G	G	G	T	G	G	G	G	G	G	G	G	G	G	G	G	G
ENSRNOSNP2784655	J343838	198482961	C	T	T	T	T	T	T	T	T	T	T	T	T	T	T	T	T	T	T	T	T	T
ENSRNOSNP2784656	J650031	198593171	G	G	G	G	G	G	G	G	G	G	G	G	G	G	G	G	G	G	-	G	G	G
ENSRNOSNP2784657	J522409	198792664	G	A	A	-	A	A	-	A	-	A	A	A	A	-	A	A	A	A	-	A	A	A
ENSRNOSNP2784658	WKYc32c05_s1_624	198837639	A	G	G	G	G	G	G	G	A	G	G	G	G	G	G	G	G	G	G	G	G	G
ENSRNOSNP2784659	rat105_010_g05.p1ca_617	198966339	T	G	G	G	G	G	G	G	T	G	G	G	G	G	G	G	G	G	G	G	G	G
ENSRNOSNP2784660	rdahl-59g15_fp2_b1_484	199008174	C	C	C	C	C	C	C	C	G	C	C	C	C	C	C	C	C	C	C	C	C	C
ENSRNOSNP2784661	J547724	199165045	G	A	A	-	A	A	-	A	-	A	A	A	A	-	A	A	A	A	-	A	A	A
ENSRNOSNP2784662	gko-8i10_rp2_b1_817	199166234	T	G	G	G	G	G	G	G	G	G	G	G	G	G	G	G	G	G	G	G	G	G
ENSRNOSNP2784663	WKYc55c12_r1_272	199384293	C	T	T	T	T	T	T	T	T	T	T	T	T	T	T	T	T	T	T	T	T	T
ENSRNOSNP2784664	J673025	199573723	A	G	G	G	G	G	G	G	G	G	G	G	G	G	G	G	G	G	G	G	G	G

ENSRNOSNP2784665	SHRSPc34d11_r1_409	199652599	C	T	T	T	T	T	T	T	C	T	T	T	T	T	T	T	T	T	T	T	T	T
ENSRNOSNP2784666	Cpn_1199896241	199896241	T	C	-	C	C	-	C	-	T	C	-	C	C	C	-	C	C	C	C	C	C	C
ENSRNOSNP2784667	rat108_029_f10.p1ca_469	199951622	A	C	C	C	C	C	C	C	A	C	C	C	C	C	C	C	C	C	C	C	C	C
ENSRNOSNP2784668	J474903	199973909	T	A	A	A	A	A	A	A	T	A	A	A	A	A	A	A	A	A	-	A	A	A
ENSRNOSNP2784669	SHRSPc53c12_s1_673	199983879	A	T	T	T	T	T	T	T	T	T	T	T	T	T	T	T	T	T	T	T	T	T
ENSRNOSNP2784671	gnl|ti|896463214_19866866707570_243	200383832	A	G	G	G	G	G	G	G	G	G	G	G	G	G	G	G	G	G	G	G	G	G
ENSRNOSNP2784672	J579132	200703738	C	T	T	T	T	T	T	T	T	T	T	T	T	T	T	T	T	T	T	T	T	T
ENSRNOSNP2784673	gko-89d19_rp2_b1_110	200884723	C	T	T	T	T	T	T	T	T	T	T	T	T	T	T	T	T	T	T	T	T	T
ENSRNOSNP2784674	rat104_025_d13.q1ca_456	201009836	G	A	A	A	A	A	A	A	A	A	A	A	A	A	A	A	A	A	A	A	A	A
ENSRNOSNP2784675	J568813	201033796	A	T	T	T	T	T	T	T	T	T	T	T	T	T	T	T	T	T	T	T	T	T
ENSRNOSNP2784676	Cpn_1201127302	201127302	A	G	G	G	G	G	G	G	G	G	G	G	G	G	G	G	G	G	G	G	G	G
ENSRNOSNP2784677	J643122	201163107	T	A	A	A	A	A	A	A	A	A	A	A	A	A	A	A	A	A	-	A	A	A
ENSRNOSNP2784678	SHRSPc54g05_r1_205	201205817	T	C	C	C	C	C	C	C	C	C	C	C	C	C	C	C	C	C	C	C	C	C
ENSRNOSNP2784679	DS-g-c-08a10_f1_719	201239290	T	-	T	T	T	T	T	T	C	T	T	T	T	T	T	T	T	T	T	T	T	T
ENSRNOSNP2784680	rat104_060_j14.q1ca_492	201288519	A	G	G	G	G	G	G	G	G	G	G	G	G	G	G	G	G	G	G	G	G	G
ENSRNOSNP2784681	J478729	201345565	G	T	T	T	T	T	T	T	G	T	T	T	T	T	T	T	T	T	T	T	T	T
ENSRNOSNP2784682	rat105_034_k23.q1ca_649	201358699	G	G	G	G	G	G	G	G	C	G	G	G	G	G	G	G	G	G	G	G	G	G
ENSRNOSNP2784683	J340946	201437648	A	T	T	T	T	T	T	T	T	T	T	T	T	T	T	T	T	T	-	T	T	T
ENSRNOSNP2784684	rat103_016_p19.p1ca_624	201491419	T	C	C	C	C	C	C	C	C	C	C	C	C	C	C	C	C	C	C	C	C	C
ENSRNOSNP2784685	gko-21k22_rp2_b1_153	201562871	T	C	C	C	C	C	C	C	T	C	C	C	C	C	C	C	C	C	C	C	C	C
ENSRNOSNP2784686	rat104_065_n09.q1ca_269	201645380	G	C	C	C	C	C	C	C	C	C	C	C	C	C	C	C	C	C	C	C	C	C
ENSRNOSNP2784687	rat110_024_n22.q1cb_156	201657836	A	C	C	C	C	C	C	C	C	C	C	C	C	C	C	C	C	C	C	C	C	C
ENSRNOSNP2784688	J657543	201667969	A	A<br>G	G	-	G	A<br>G	-	A<br>G	A<br>G	G	G	-	A<br>G	-	A<br>G	G	A<br>G	A<br>G	-	A<br>G	G	A<br>G
ENSRNOSNP2784689	rat102_037_d08.q1ca_392	201717322	G	A	A	A	A	A	A	A	G	A	A	A	G	A	A	A	A	A	A	A	A	A
ENSRNOSNP2784690	J532064	201785269	A	G	G	-	G	G	-	G	G	G	G	G	G	-	G	G	G	G	-	G	G	G
ENSRNOSNP2784691	Cpn_1202010692	202010692	A	A	A	A	A	A	A	A	C	A	A	A	C	A	A	A	A	A	A	A	A	A
ENSRNOSNP2784692	J582115	202107918	C	T	T	T	T	T	T	T	T	T	T	T	T	T	T	T	T	T	T	T	T	T
ENSRNOSNP2784693	rdahl-90f6_fp2_b1_504	202355992	T	C	C	C	C	C	C	C	C	C	C	C	C	C	C	C	C	C	C	C	C	C
ENSRNOSNP2784694	J664042	202411515	C	T	T	T	T	T	T	T	T	T	T	T	T	T	T	T	T	T	T	T	T	T
ENSRNOSNP2784695	SHRSPc11d09_s2_487	202690116	C	A	A	A	A	A	A	A	C	A	A	A	C	A	A	A	A	A	A	A	A	A
ENSRNOSNP2784696	J1300005	202813531	G	T	T	T	T	T	T	T	G	T	T	T	G	T	T	T	T	T	T	T	T	T
ENSRNOSNP2784697	gko-7k12_rp2_b1_68	202813776	C	G	G	G	G	G	G	G	C	G	G	G	C	G	G	G	G	G	G	G	G	G
ENSRNOSNP2784698	J673128	202942942	G	A	A	A	A	A	A	A	A	A	A	A	A	A	A	A	A	A	-	A	A	A
ENSRNOSNP2784699	gnl|ti|896962923_19866868044073_303	203121880	G	T	T	T	T	T	T	T	G	T	T	T	G	T	T	T	T	T	T	T	T	T
ENSRNOSNP2784700	J1266358	203185124	C	G	G	-	G	G	-	G	-	G	G	G	-	-	G	G	G	G	-	G	G	G
ENSRNOSNP2784701	Cpn_1203295449	203295449	A	G	G	G	G	G	G	G	G	G	G	G	G	G	G	G	G	G	G	G	G	G
ENSRNOSNP2784702	J549517	203403072	T	C	C	C	C	C	C	C	C	C	C	C	C	C	C	C	C	C	-	C	C	C
ENSRNOSNP2784703	gko-99o9_rp2_b1_255	203479457	T	C	C	C	C	C	C	C	C	C	C	C	C	C	C	C	C	C	C	C	C	C
ENSRNOSNP2784705	rat103_031_c08.p1ca_151	203857537	G	G	G	G	G	G	G	G	T	G	G	G	T	G	G	G	G	G	G	G	G	G

ENSRNOSNP2784706	rat104_020_e15.q1ca_314	203858375	G	A	A	A	A	A	A	A	A	A	A	A	A	A	A	A	A	A	A	A	A	A
ENSRNOSNP2784707	J1275564	203871259	A	G	G	G	G	G	G	G	A	G	G	G	A	G	G	G	G	G	-	G	G	G
ENSRNOSNP2784708	Cpn_1203891431	203891431	G	T	T	T	T	T	T	T	G	T	T	T	G	T	T	T	T	T	T	T	T	T
ENSRNOSNP2784709	rat104_077_a02.q1ca_162	203934819	G	A	A	A	A	A	A	A	G	A	A	A	G	A	A	A	A	A	A	A	A	A
ENSRNOSNP2784710	rat103_024_l04.p1ca_500	204066186	G	G	G	G	G	G	G	G	A	G	G	G	A	G	G	G	G	G	G	G	G	G
ENSRNOSNP2784711	rat103_012_o11.p1ca_90	204187969	C	A	A	A	A	A	A	A	A	A	A	A	A	A	A	A	A	A	A	A	A	A
ENSRNOSNP2784712	rat110_007_i20.q1cb_742	204258191	G	A	A	A	A	A	A	A	G	A	A	A	G	A	A	A	A	A	A	A	A	A
ENSRNOSNP2784713	rat110_017_o14.q1ca_395	204258541	G	A	A	A	A	A	A	A	G	A	A	A	G	A	A	A	A	A	A	A	A	A
ENSRNOSNP2784714	J562698	204270162	A	G	G	G	G	G	G	G	G	G	G	G	G	G	G	G	G	G	-	G	G	G
ENSRNOSNP2784715	WKYOa01b03_r1_818	204324576	G	A	A	A	A	A	A	A	G	A	A	A	G	A	A	A	A	A	A	A	A	A
ENSRNOSNP2784716	J581488	204391572	A	G	G	G	G	G	G	G	A	G	G	G	A	G	G	G	G	G	G	G	G	G
ENSRNOSNP2784717	gko-34e12_rp2_b1_147	204573537	C	T	T	T	T	T	T	T	C	T	T	T	C	T	T	T	T	T	T	T	T	T
ENSRNOSNP2784718	SHRSPc52d06_s1_444	204793594	T	C	C	C	C	C	C	C	C	C	C	C	C	C	C	C	C	C	C	C	C	C
ENSRNOSNP2784719	rat108_042_h04.p1cb_350	204878579	A	C	C	C	C	C	C	C	A	C	C	C	A	C	C	C	C	C	C	C	C	C
ENSRNOSNP2784720	J468482	205041917	A	G	G	G	G	G	G	G	G	G	G	G	G	G	G	G	G	G	-	G	G	G
ENSRNOSNP2784721	gko-18d13_rp2_b1_157	205216272	A	-	A	A	A	A	A	A	G	A	A	A	G	A	A	A	A	A	A	G	G	G
ENSRNOSNP2784722	J517612	205376613	T	T	T	-	T	T	-	T	TC	T	T	T	TC	-	T	T	T	T	-	TC	C	TC
ENSRNOSNP2784723	WKY-G-j-12a09_f1_1130	205671704	T	C	C	C	C	C	C	C	C	C	C	C	C	C	C	C	C	C	C	T	T	T
ENSRNOSNP2784724	J544198	205733521	T	T	T	T	T	T	T	T	C	T	T	T	C	T	T	T	T	T	T	C	C	C
ENSRNOSNP2784725	rat106_017_o23.q1ca_142	205759970	C	C	C	C	C	C	C	C	A	C	C	C	A	C	C	C	C	C	C	A	A	A
ENSRNOSNP2784726	gko-79m3_rp2_b1_52	206245790	G	G	G	G	G	G	G	G	G	G	G	G	G	G	G	G	G	G	G	A	A	A
ENSRNOSNP2784727	gko-21a12_fp2_b1_165	206413402	T	T	T	T	T	T	T	T	G	T	T	T	G	T	T	T	T	T	T	G	G	G
ENSRNOSNP2784728	J594050	206552616	C	C	C	C	C	C	C	C	C	C	C	C	C	C	C	C	C	C	-	G	G	G
ENSRNOSNP2784729	Cpn_1206682156	206682156	G	G	G	G	G	G	G	G	A	G	G	G	A	G	G	G	G	G	G	G	G	G
ENSRNOSNP2784730	rat013_028_h13.q1ca_705	206800577	T	T	T	T	T	T	T	T	A	T	T	T	A	T	T	T	T	T	T	T	T	T
ENSRNOSNP2784731	rat107_026_k13.p1ca_310	206816789	G	G	G	G	G	G	G	G	A	G	G	G	A	G	G	G	G	G	G	A	A	A
ENSRNOSNP2784732	J579069	206878409	C	C	C	C	C	C	C	C	C	C	C	C	C	C	C	C	C	C	C	T	T	T
ENSRNOSNP2784733	rat103_041_f02.q1ca_544	207000773	G	G	G	G	G	G	G	G	A	G	G	G	A	G	G	G	G	G	G	A	A	A
ENSRNOSNP2784734	J519024	207066536	C	C	C	C	C	C	C	C	C	C	C	C	C	C	C	C	C	C	-	T	T	T
ENSRNOSNP2784735	J541152	207171392	A	A	A	A	A	A	A	A	G	A	A	A	G	A	A	A	A	A	-	G	G	G
ENSRNOSNP2784736	Cpn_1207450582	207450582	G	G	G	G	G	G	G	G	A	G	G	G	A	G	G	-	G	G	G	G	G	G
ENSRNOSNP2784737	rat106_025_f16.p1ca_195	207503859	C	C	C	C	C	C	C	C	T	C	C	C	T	C	C	C	C	C	C	C	C	C
ENSRNOSNP2784738	rat105_019_j14.p1ca_275	207546592	C	C	C	C	C	C	C	C	A	C	C	C	A	C	C	C	C	C	C	C	C	C
ENSRNOSNP2784739	rat103_002_i01.p1ma_346	207659997	A	A	A	A	A	A	A	A	G	A	A	A	G	A	A	A	A	A	A	G	G	G
ENSRNOSNP2784741	J469256	207715030	A	A	A	-	A	A	-	A	A	A	A	A	A	-	A	A	A	A	-	G	G	G
ENSRNOSNP2784742	rat105_008_d14.q1cb_380	207991933	T	T	T	T	T	T	T	T	G	T	T	T	G	T	T	T	T	T	T	G	G	G
ENSRNOSNP2784743	rat103_036_e09.p1ca_64	208010733	T	T	T	T	T	T	T	T	C	T	T	T	C	T	T	T	T	T	T	C	C	C
ENSRNOSNP2784744	J523349	208029058	C	-	C	-	C	C/A	-	-	-	C	C	C	-	-	-	C	-	-	-	-	C	C
ENSRNOSNP2784745	gko-89f18_fp2_b1_229	208045208	C	C	C	C	C	C	C	C	C	C	C	C	C	C	C	C	C	C	C	T	T	T

ENSRNOSNP2784747	J512251	208365046	C	C	C	C	C	C	C	C	C	C	C	C	C	C	C	C	C	C	C	T	T	T
ENSRNOSNP2784749	J597981	208943871	G	G	G	G	G	G	G	G	G	G	G	G	G	G	G	G	G	G	-	A	A	A
ENSRNOSNP2784751	rat107_039_n19.q1ca_544	209120111	C	C	C	C	C	C	C	C	T	C	C	C	T	C	C	C	C	C	C	C	C	C
ENSRNOSNP2784752	J591772	209238631	T	T	T	T	T	T	T	T	C	T	T	T	C	T	T	T	T	T	-	C	C	C
ENSRNOSNP2784753	gko-55m21_rp2_b1_391	209356367	G	G	G	G	G	G	G	G	G	G	G	G	G	G	G	G	G	G	G	A	A	A
ENSRNOSNP2784754	gnl|ti|842489386_50201915_185	210111319	T	T	T	T	T	T	T	T	C	T	T	T	C	T	T	T	T	T	T	T	T	T
ENSRNOSNP2784755	J490759	210305935	T	T	T	T	T	T	T	T	C	T	T	T	C	T	T	T	T	T	T	C	C	C
ENSRNOSNP2784756	gko-34o3_fp2_b1_278	210307659	T	T	T	T	T	T	T	T	C	T	T	T	C	T	T	T	T	T	T	C	C	C
ENSRNOSNP2784757	J484809	210725491	G	-	G	-	G	-	-	-	A	G	G	G	A	-	-	G	-	-	-	A	A	A
ENSRNOSNP2784760	gko-111a21_rp2_b1_328	211452603	C	C	C	C	C	C	C	C	T	C	C	C	T	C	C	C	C	C	C	T	T	T
ENSRNOSNP2784761	J520769	211712619	G	G	G	G	G	G	G	G	G	G	G	G	G	G	G	G	G	G	-	A	A	A
ENSRNOSNP2784763	J504423	211984589	A	A	A	A	A	A	A	A	T	A	A	A	T	A	A	A	A	A	A	T	T	T
ENSRNOSNP2784764	J504362	212207881	A	A	A	A	A	A	A	A	G	A	A	A	G	A	A	A	A	A	-	G	G	G
ENSRNOSNP2784765	J546914	212326492	T	T	T	T	T	T	T	T	C	T	T	T	C	T	T	T	T	T	T	C	C	C
ENSRNOSNP2784767	rat108_004_h18.p1ca_421	212684637	C	T	T	T	T	T	T	T	C	T	T	T	C	T	T	T	T	T	T	C	C	C
ENSRNOSNP2784768	WKYc74e05_s1_234	212698251	C	T	T	T	T	T	T	T	C	T	T	T	C	T	T	T	T	T	T	C	C	C
ENSRNOSNP2784769	J583174	212700831	C	C	C	C	C	C	C	C	C	C	C	C	C	C	C	C	C	C	C	C	C	C
ENSRNOSNP2784770	J552110	213054299	T	-	G	-	G	-	-	-	-	G	G	G	-	-	-	G	-	-	-	-	G	G
ENSRNOSNP2784771	SHRSPc69a08_r1_506	213097073	A	C	C	C	C	C	C	C	C	C	C	C	C	C	C	C	C	C	C	C	C	C
ENSRNOSNP2784772	J522133	213360576	T	T	T	T	T	G	G	T	T	G	G	G	T	G	G	G	G	G	G	G	G	G
ENSRNOSNP2784773	SHRSPa93a03_r1_927	213369475	T	T	T	T	T	C	C	T	T	C	C	C	T	C	C	C	C	C	C	C	C	C
ENSRNOSNP2784774	J687515	213490233	T	T	T	T	T	C	C	T	T	C	C	C	T	C	C	C	C	C	-	C	C	C
ENSRNOSNP2784775	J687665	213695414	G	G	G	G	G	G	G	G	G	G	G	G	G	G	G	G	G	G	G	A	A	A
ENSRNOSNP2784776	J552187	213847769	T	T	T	T	T	C	C	T	T	C	C	C	T	C	C	C	C	C	-	C	C	C
ENSRNOSNP2784777	J564805	214191508	G	G	G	-	G	-	-	G	G	-	-	-	G	-	-	-	-	-	-	A	A	A
ENSRNOSNP2784778	rat110_007_h05.q1ca_632	214553496	A	A	A	A	A	G	G	A	A	G	G	G	A	G	G	G	G	G	G	G	G	G
ENSRNOSNP2784779	gko-67h11_rp2_b1_746	214567332	G	G	G	G	G	G	G	G	G	G	G	G	G	G	G	G	G	G	G	A	A	A
ENSRNOSNP2784780	J483181	214980672	A	A	A	A	A	G	G	A	A	G	G	G	A	G	G	G	G	G	G	G	G	G
ENSRNOSNP2784781	gko-50o16_fp2_b1_555	215270348	T	T	T	T	T	C	C	T	T	C	C	C	T	C	C	C	C	C	C	C	C	C
ENSRNOSNP2784782	J538266	215357322	G	G	G	G	G	G	G	G	G	G	G	G	G	G	G	G	G	G	G	C	C	C
ENSRNOSNP2784783	gko-44k23_fp2_b1_186	215458051	C	C	C	C	C	C	C	C	C	C	C	C	C	C	C	C	C	C	C	T	T	T
ENSRNOSNP2784785	rat013_012_f08.q1ca_137	215633597	A	A	A	A	A	A	A	A	A	A	A	A	A	A	A	A	A	A	A	G	G	G
ENSRNOSNP2784786	rat105_003_e24.q1ca_567	215843126	G	G	G	G	G	A	A	G	G	A	A	A	G	A	A	A	A	A	A	A	A	A
ENSRNOSNP2784787	SHRSPa17h02_r1_1010	215903496	A	A	A	A	A	G	G	A	A	G	G	G	A	G	G	G	G	G	G	G	G	G
ENSRNOSNP2784788	J519647	216100661	T	T	T	T	T	T	T	T	T	T	T	T	T	T	T	T	T	T	T	A	A	A
ENSRNOSNP2784789	J586397	216895614	G	G	G	G	G	G	G	G	G	G	G	G	G	G	G	G	G	G	-	A	A	A
ENSRNOSNP2784790	J565153	217078676	A	A	A	A	A	G	G	A	A	G	G	G	A	G	G	G	G	G	G	G	G	G
ENSRNOSNP2784791	SHRSPa63h07_r1_487	217173732	C	C	C	C	C	G	G	C	C	G	G	G	C	G	G	G	G	G	G	G	G	G
ENSRNOSNP2784792	gko-38i6_fp2_b1_442	217347493	A	A	A	A	A	G	G	A	A	G	G	G	A	G	G	G	G	G	G	G	G	G

ENSRNOSNP2784793	J337103	217498692	A	A	A	A	A	G	G	A	A	G	G	G	A	G	G	G	G	G	G	G	G	G
ENSRNOSNP2784794	rat106_012_m15.p1ca_509	217680074	T	C	C	C	C	C	C	C	C	C	C	C	C	C	C	C	C	C	C	C	C	C
ENSRNOSNP2784795	J1292750	217684703	T	C	C	C	C	C	C	C	C	C	C	C	C	C	C	C	C	C	-	T	T	T
ENSRNOSNP2784796	WKYc13f12_s1_715	217757979	A	G	G	G	G	G	G	G	G	G	G	G	G	G	G	G	G	G	G	A	A	A
ENSRNOSNP2784797	J673261	217787646	G	A	A	A	A	A	A	A	A	A	A	A	A	A	A	A	A	A	-	G	G	G
ENSRNOSNP2784798	SHRSPc27b07_r1_136	217793653	C	G	G	G	G	G	G	G	G	G	G	G	G	G	G	G	G	G	G	C	C	C
ENSRNOSNP2784799	J501411	217960224	T	TC	C	C	C	C	C	C	C	C	C	C	C	C	C	C	C	C	C	TC	C	C
ENSRNOSNP2784800	rdahl-82l4_rp2_b1_81	217969821	T	C	C	C	C	C	C	C	C	C	C	C	C	C	C	C	C	C	C	C	C	C
ENSRNOSNP2784801	gko-76n6_rp2_b1_68	218123598	T	C	C	C	C	C	C	C	C	C	C	C	C	C	C	C	C	C	C	C	C	C
ENSRNOSNP2784802	J528117	218271781	T	T<br>A	-	-	-	T<br>A	-	T<br>A	T<br>A	A	A	T<br>A	T<br>A	-	T<br>A	T<br>A	T<br>A	T<br>A	-	T<br>A	A	T<br>A
ENSRNOSNP2784803	rdahl-15f17_fp2_b1_177	218322153	C	T	T	T	T	C	C	T	T	C	C	C	T	C	C	C	C	C	C	C	C	C
ENSRNOSNP2784804	J528173	218448462	C	C	C	C	C	C	C	C	C	C	C	C	C	C	C	C	C	C	-	T	T	T
ENSRNOSNP2784805	gnl|ti|896658520_19866868287293_270	218463655	G	-	-	T	T	G	G	-	T	G	G	G	T	G	G	G	G	G	G	G	G	G
ENSRNOSNP2784806	J661574	218583670	G	A	A	A	A	A	G	A	A	G	G	G	A	G	G	G	G	G	G	G	G	G
ENSRNOSNP2784807	J564379	218708639	A	T	T	T	T	T	A	T	T	A	A	A	T	A	A	A	A	A	-	T	T	T
ENSRNOSNP2784808	gko-19i4_rp2_b1_571	218710299	G	A	A	A	A	A	G	A	A	G	G	G	A	G	G	G	G	G	G	A	A	A
ENSRNOSNP2784810	rdahl-19c3_rp2_b1_395	218987150	C	A	A	A	A	A	C	A	A	C	C	C	A	C	C	C	C	C	C	A	A	A
ENSRNOSNP2784811	J673110	219066139	G	A	A	A	A	A	G	A	A	G	G	G	A	G	G	G	G	G	-	A	A	A
ENSRNOSNP2784812	J1264834	219240758	C	G	G	G	G	G	C	G	G	C	C	C	G	C	C	C	C	C	C	G	G	G
ENSRNOSNP2784813	gko-118k9_rp2_b1_197	219279209	A	A	A	A	A	A	A	A	A	A	A	A	A	A	A	A	A	A	A	T	T	T
ENSRNOSNP2784814	J668530	219601279	C	T	T	T	T	T	C	T	T	C	C	C	T	C	C	C	C	C	C	C	C	C
ENSRNOSNP2784815	WKY-G-j-55e03_f1_505	219650011	A	C	C	C	C	C	A	C	C	A	A	A	C	A	A	A	A	A	A	C	C	C
ENSRNOSNP2784816	rat105_017_e23.p1ca_490	219766152	A	G	G	G	G	G	A	G	G	A	A	A	G	A	A	A	A	A	A	A	A	A
ENSRNOSNP2784817	gnl|ti|486467735_28450740_463	219794692	A	-	A	A	A	A	A	A	A	A	A	A	A	A	A	A	-	A	A	A	A	A
ENSRNOSNP2784818	rat107_012_p14.p1ca_213	219857076	C	C	C	C	C	C	C	C	C	C	C	C	C	C	C	C	C	C	C	T	T	T
ENSRNOSNP2784819	J1295259	219923963	C	T	T	T	T	T	C	T	T	C	C	C	T	C	C	C	C	C	C	C	C	C
ENSRNOSNP2784820	Cpn_1219988698	219988698	A	T	T	T	T	T	A	T	T	A	A	A	T	A	A	A	A	A	A	T	T	T
ENSRNOSNP2784821	rat105_017_a21.q1ca_493	219989584	A	G	G	G	G	G	A	G	G	A	A	A	G	A	A	A	A	A	A	G	G	G
ENSRNOSNP2784822	gko-109h15_rp2_b1_648	220207165	T	C	C	C	C	C	T	C	C	T	T	T	C	T	T	T	T	T	T	C	C	C
ENSRNOSNP2784823	J696731	220366233	A	G	G	G	G	G	G	G	G	G	G	G	G	G	G	G	G	G	-	G	G	G
ENSRNOSNP2784824	gnl|ti|896926356_19866868269857_281	220461004	T	C	C	C	-	C	C	C	C	C	C	C	C	C	C	C	C	C	C	C	C	C
ENSRNOSNP2784825	J655794	220574228	T	T	T	T	T	T	C	T	T	C	C	C	T	C	C	C	TC	TC	C	TC	C	C
ENSRNOSNP2784826	rdahl-59k17_fp2_b1_630	220638344	A	A	A	A	A	A	G	A	A	G	G	G	A	G	G	G	G	G	G	G	G	G
ENSRNOSNP2784827	J643631	220678460	C	C	C	C	C	C	C	C	C	C	C	C	C	C	C	C	C	C	-	C	C	C
ENSRNOSNP2784828	rat105_034_d09.p1ca_273	220725810	T	T	T	T	T	T	T	T	T	T	T	T	T	T	T	T	T	T	T	T	T	T
ENSRNOSNP2784829	gnl|ti|896971685_19866868306746_227	220867150	G	G	G	G	G	G	G	G	G	G	G	G	G	G	G	G	G	G	G	G	G	G
ENSRNOSNP2784830	rdahl-77n7_fp2_b1_137	221336519	A	A	A	A	A	A	A	A	A	A	A	A	A	A	A	A	A	A	A	A	A	A
ENSRNOSNP2784831	gnl|ti|896771844_19866866822766_304	221734297	T	T	T	T	T	T	T	T	T	T	T	T	T	T	T	T	-	T	T	T	T	T
ENSRNOSNP2784832	rdahl-47e20_fp2_b1_318	221967464	A	A	A	A	A	A	A	A	A	A	A	A	A	A	A	A	A	A	A	A	A	A

ENSRNOSNP2784833	DS-g-a-38g08_r1_491	222249801	C	C	C	C	C	C	T	C	C	T	T	T	C	T	C	C	C	C	-	T	T	T
ENSRNOSNP2784834	SHRSPa43h04_s1_819	222478178	T	C	C	C	C	C	T	C	C	T	T	T	C	T	C	C	C	C	C	C	C	C
ENSRNOSNP2784835	J596140	223093095	A	-	A	-	A	A/T	-	-	-	A	A	A	-	-	-	A	A/T	A/T	-	A/T	A	A
ENSRNOSNP2784836	SHRSPc33f05_r1_338	223127353	G	C	C	C	C	C	C	C	C	G	G	G	C	G	C	C	C	C	C	C	C	C
ENSRNOSNP2784837	J506310	223188441	A	C	C	-	C	C	-	C	C	C	C	C	C	-	C	C	C	C	-	C	C	C
ENSRNOSNP2784838	J1260586	223474188	C	T	T	T	T	T	T	T	T	C	C	C	T	C	T	T	T	T	T	T	T	T
ENSRNOSNP2784839	J482894	223704445	C	T	T	T	T	T	T	T	T	C	C	C	T	C	T	T	T	T	-	T	T	T
ENSRNOSNP2784840	gnl|ti|896840146_19866867170110_323	223710972	T	A	A	A	A	A	A	A	A	T	T	T	A	T	A	A	A	A	A	A	A	A
ENSRNOSNP2784841	J559161	223814404	G	A	A	A	A	A	A	A	A	A	A	A	A	A	A	A	A	A	A	G	A	A
ENSRNOSNP2784842	gnl|ti|896828697_19866868382869_454	223864981	A	G	G	G	G	G	G	G	G	G	G	G	G	G	G	G	G	G	G	A	G	G
ENSRNOSNP2784843	J574278	223948650	C	A	A	A	A	A	A	A	A	A	A	A	A	A	A	A	A	A	-	C	A	A
ENSRNOSNP2784844	J527939	224128466	G	C	C	C	C	C	C	C	C	G	G	G	C	G	C	C	C	C	C	G	C	C
ENSRNOSNP2784845	gnl|ti|896607033_19866866956703_245	224216155	G	T	T	T	T	T	T	T	T	G	G	G	T	G	T	T	T	T	T	G	T	T
ENSRNOSNP2784846	J528500	224226608	A	G	G	G	G	G	G	G	G	A	A	A	G	A	G	G	G	G	-	A	G	G
ENSRNOSNP2784847	J679686	224339653	C	G	G	G	G	G	G	G	G	C	C	C	G	C	G	G	G	G	-	C	G	G
ENSRNOSNP2784848	gko-17c4_rp2_b1_643	224377627	A	C	C	C	C	C	C	C	C	A	A	A	C	A	C	C	C	C	C	A	C	C
ENSRNOSNP2784849	rat109_042_b24.q1ca_347	224450553	C	T	T	C/T	T	T	CT	T	T	C	C	C	T	C	T	T	T	T	C/T	C	T	T
ENSRNOSNP2784850	J876926	224498444	T	C	C	C	C	C	C	C	C	T	T	T	C	T	C	C	C	C	-	T	C	C
ENSRNOSNP2784851	gko-17a18_rp2_b1_736	224530235	C	G	G	G	G	G	G	G	G	C	C	C	G	C	G	G	G	G	G	C	G	G
ENSRNOSNP2784852	J525939	224740245	T	C	C	C	C	C	C	C	C	C	C	C	C	C	C	C	C	C	-	C	C	C
ENSRNOSNP2784853	rat101_026_f17.q1ca_517	225073386	A	G	G	G	G	G	G	G	G	G	G	G	G	G	G	G	G	G	G	G	G	G
ENSRNOSNP2784854	Cpn_1225096659	225096659	G	-	T	T	T	T	T	T	T	G	G	G	T	G/T	T	T	-	T	T	G	T	T
ENSRNOSNP2784855	DS-g-a-29b11_f1_39	225292689	C	C	C	C	C	C	C	C	C	C	C	C	C	C	C	C	C	C	C	C	C	C
ENSRNOSNP2784856	gko-61c14_fp2_b1_634	225467067	C	T	T	T	T	T	T	T	T	T	T	T	T	T	T	T	T	T	T	T	T	T
ENSRNOSNP2784857	J658194	225579245	G	G	G	G	G	G	G	G	G	G	G	G	G	G	G	G	G	G	-	G	G	G
ENSRNOSNP2784858	gko-33i19_fp2_b1_113	225730889	C	T	T	T	T	T	T	T	T	C	C	C	T	C	T	T	T	T	T	C	T	T
ENSRNOSNP2784859	J576143	225788091	C	T	T	T	T	T	T	T	T	T	T	T	T	T	T	T	T	T	T	T	T	T
ENSRNOSNP2784860	Cpn_1225978942	225978942	A	-	A	A	A	A	A	A	A	A	A	A	A	A	A	A	A	A	A	A	A	A
ENSRNOSNP2784861	J1260913	226097882	T	TC	C	C	C	TC	C	-	TC	T	T	T	TC	T	-	C	TC	TC	C	T	C	C
ENSRNOSNP2784862	WKYc61d08_r1_454	226148316	C	T	T	T	T	T	T	T	T	C	C	C	C	-	T	T	T	T	T	C	T	T
ENSRNOSNP2784863	J473945	226226832	T	C	C	C	C	C	C	C	C	T	T	T	C	T	C	C	C	C	-	T	C	C
ENSRNOSNP2784864	rat101_039_h14.q1ca_398	226279794	C	G	G	G	G	G	G	G	G	G	G	G	G	G	G	G	G	G	G	C	G	G
ENSRNOSNP2784865	SHRSPa56g02_r1_538	226391086	T	G	G	G	G	G	G	G	G	T	T	T	T	T	G	G	G	G	G	T	T	T
ENSRNOSNP2784866	WKYc16a05_s1_488	226429326	T	C	C	C	C	C	C	C	C	C	C	C	C	C	C	C	C	C	C	T	T	T
ENSRNOSNP2784867	rat106_003_f22.q1ca_142	226446443	A	A	A	A	A	A	A	A	A	A	A	A	A	A	A	A	A	A	A	A	A	A
ENSRNOSNP2784868	gnl|ti|896830637_19866868228151_299	226580639	C	G	G	G	G	G	G	G	G	C	C	C	G	C	G	G	G	G	G	C	C	C
ENSRNOSNP2784869	J666471	226640285	A	A/G	G	-	G	A/G	-	A/G	A/G	G	G	G	A/G	-	A/G	G	A/G	A/G	-	A	A	A
ENSRNOSNP2784870	J341248	226850093	A	G	G	G	G	G	G	G	G	G	G	G	A	G	G	G	G	G	-	G	A	A
ENSRNOSNP2784871	gnl|ti|896617915_19866867129164_382	227045735	C	C	C	C	C	C	C	C	C	G	G	G	C	G	C	C	C	C	C	G	C	C

ENSRNOSNP2784872	SHRSPc54e04_r1_72	227265052	G	A	A	A	A	A	A	A	A	G	G	G	G	G	A	A	A	A	A	G	G	G
ENSRNOSNP2784873	rat108_030_e24.q1ca_539	227335895	T	C	C	C	C	C	C	C	C	C	C	C	C	C	C	C	C	C	C	C	T	T
ENSRNOSNP2784874	gnl|ti|896820859_19866866776828_277	227639603	A	A	A	A	A	A	A	A	A	A	A	A	A	A	A	A	A	A	A	A	A	A
ENSRNOSNP2784875	rat102_025_l09.p1ca_173	227646045	G	T	T	T	T	T	T	T	T	G	G	G	G	G	T	T	T	T	T	G	G	G
ENSRNOSNP2784876	WKYc15d06_s1_573	227813881	G	-	A	A	A	A	A	A	A	A	A	A	A	A	A	A	A	A	A	A	A	A
ENSRNOSNP2784877	J337594	227966280	C	A	A	A	A	A	A	A	A	C	C	C	C	C	A	A	A	A	A	A	A	A
ENSRNOSNP2784878	WKY-G-j-60d01_r1_352	228072240	G	A	A	A	A	A	A	A	A	G	G	G	G	G	A	A	A	A	A	A	A	A
ENSRNOSNP2784879	gnl|ti|897027681_19866867976161_232	228434121	T	T	T	T	T	T	T	T	G	G	G	G	G	G	G	G	G	G	G	T	T	T
ENSRNOSNP2784880	gnl|ti|896531497_19866867344669_287	228575734	A	A	A	A	A	A	A	A	C	C	C	C	C	C	C	C	C	C	C	A	A	A
ENSRNOSNP2784881	J685977	228728922	A	C	A	A	C	C	A	A	C	C	C	C	C	C	C	C	C	C	C	A	A	A
ENSRNOSNP2784882	gko-37n21_rp2_b1_120	228900903	G	G	-	G	G	G	G	G	G	G	G	G	G	G	G	G	G	G	G	A	A	A
ENSRNOSNP2784883	gko-118g10_rp2_b1_118	229399235	G	G	T	T	G	G	T	T	G	T	T	T	G	T	G	G	G	G	G	T	T	T
ENSRNOSNP2784884	J342589	229877171	G	A	G	G	A	A	G	G	A	G	G	G	A	G	A	A	A	A	-	G	G	G
ENSRNOSNP2784885	gnl|ti|896620335_19866867173869_210	229879574	A	G	A	A	G	G	A	A	G	A	A	A	G	A	G	G	G	G	G	A	A	A
ENSRNOSNP2784886	gko-97k15_rp2_b1_590	230020491	C	C	T	T	C	C	T	T	C	T	T	T	C	T	C	C	C	C	C	T	T	T
ENSRNOSNP2784887	gko-87i3_fp2_b1_431	230242767	T	T	C	C	T	T	C	C	T	C	C	C	T	C	T	T	T	T	T	C	C	C
ENSRNOSNP2784888	SHRSPa28g02_s1_103	230310655	C	C	C	C	C	C	C	C	C	C	C	C	C	C	C	T	C	C	C	C	C	C
ENSRNOSNP2784889	gko-68g20_fp2_b1_82	230389433	C	T	T	T	T	T	T	T	T	T	T	T	T	T	T	T	T	T	T	T	T	T
ENSRNOSNP2784890	J581210	230389782	T	C	C	C	C	C	C	C	C	C	C	C	C	C	C	C	C	C	C	C	C	C
ENSRNOSNP2784891	J598254	230619646	C	A	C	C	A	A	C	C	A	A	A	A	A	C	A	A	A	A	-	A	A	A
ENSRNOSNP2784892	gko-119a17_rp2_b1_614	230628906	G	-	G	G	-	A	G	G	A	A	-	A	A	G	A	A	A	A	A	A	A	A
ENSRNOSNP2784893	J1310798	230740802	G	T	T	T	T	T	T	T	T	T	T	T	T	T	T	T	T	T	T	G	G	G
ENSRNOSNP2784894	gnl|ti|896725419_19866867101706_269	230861125	G	G	A	A	G	G	-	A	G	G	G	G	G	A	G	G	G	G	G	A	A	A
ENSRNOSNP2784895	rat105_017_h23.p1ca_437	230917961	T	G	G	G	G	G	G	G	G	G	G	G	G	G	G	G	G	G	G	G	G	G
ENSRNOSNP2784896	J472494	230984770	T	A	A	A	A	A	A	A	A	A	A	A	A	A	A	A	A	A	-	A	A	A
ENSRNOSNP2784897	gnl|ti|896782797_19866868312915_275	231057531	A	C	C	C	C	C	C	C	C	C	C	C	C	C	C	C	C	C	C	C	C	C
ENSRNOSNP2784898	SHRSPc29h07_r1_164	231210039	C	-	C	C	T	T	C	C	T	T	-	T	T	C	T	T	T	T	T	C	C	C
ENSRNOSNP2784899	SHRSPa48f04_r1_629	231245082	A	T	A	A	T	T	A	A	T	T	T	T	T	A	T	T	T	T	T	A	A	A
ENSRNOSNP2784900	gnl|ti|896773589_19866866939034_293	231424978	C	G	G	G	G	G	G	G	G	G	G	G	G	G	G	G	G	G	G	G	G	G
ENSRNOSNP2784901	rat101_032_m24.p1ca_601	231586914	C	G	C	C	G	G	C	C	G	G	G	G	G	C	G	G	G	G	G	C	C	C
ENSRNOSNP2784902	J533436	231608383	G	A	A	A	A	A	A	A	A	A	A	A	A	A	A	A	A	A	A	A	A	A
ENSRNOSNP2784903	J586908	231882085	A	A	G	G	A	A	G	G	A	A	A	A	A	G	A	A	A	A	-	G	G	G
ENSRNOSNP2784904	WKYc86a03_s1_569	231992987	C	T	T	T	T	T	T	T	T	C	C	C	T	T	T	T	T	T	T	T	T	T
ENSRNOSNP2784905	J524891	232036319	A	G	G	G	G	G	G	G	G	G	G	G	G	G	G	G	G	G	G	G	G	G
ENSRNOSNP2784906	gko-35h15_fp2_b1_630	232196547	A	A<br>G	G	G	A	A	G	G	A	A	A	A	A	G	A	A	A	A	A	G	G	G
ENSRNOSNP2784907	SHRSPa16e07_r1_578	232495583	G	T	T	T	T	T	T	T	T	T	T	T	T	T	T	T	T	T	T	T	T	T
ENSRNOSNP2784908	rat108_028_m24.q1ca_535	232549532	A	G	G	G	G	G	G	G	G	G	G	G	G	G	G	G	G	G	G	G	G	G
ENSRNOSNP2784909	rat108_028_m24.q1ca_232	232549835	C	T	C	C	C	T	C	C	T	C	C	C	T	C	T	T	T	T	T	C	C	C
ENSRNOSNP2784910	rat101_004_e05.q1ca_165	232549902	T	C	C	C	C	C	C	C	C	C	C	C	C	C	C	C	C	C	C	C	C	C

ENSRNOSNP2784911	J337496	232551337	T	C	C	C	C	C	C	C	C	C	C	C	C	C	C	C	C	C	-	C	C	C
ENSRNOSNP2784912	rat104_061_k24.q1ca_528	232577215	C	C	G	G	G	C	G	G	C	C	C	C	C	G	C	C	C	C	C	G	G	G
ENSRNOSNP2784913	rat109_031_i15.q1ca_630	232652991	T	C	C	C	C	C	C	C	C	C	C	C	C	C	C	C	C	C	C	C	C	C
ENSRNOSNP2784914	rat108_044_j09.p1cb_531	232658295	T	G	G	G	G	G	G	G	G	G	G	G	G	G	G	G	G	G	G	G	G	G
ENSRNOSNP2784915	J481729	232663304	G	A	A	A	A	A	A	A	A	A	A	A	A	A	A	A	A	A	-	A	A	A
ENSRNOSNP2784916	gnl|ti|521885291_31478933_197	233057952	C	C	C	C	C	C	C	C	C	C	C	C	C	C	C	C	C	C	C	C	C	C
ENSRNOSNP2784917	rat108_043_h24.p1ca_329	233067602	G	-	A	A	A	A	A	A	A	A	A	A	A	A	A	A	A	A	A	A	A	A
ENSRNOSNP2784918	gko-84e14_rp2_b1_658	233279014	G	A	G	G	G	A	G	G	A	A	A	A	A	G	A	A	A	A	A	A	A	A
ENSRNOSNP2784919	J530954	233344691	A	C	A	A	A	C	A	A	C	A	A	A	C	A	C	C	C	C	C	C	C	C
ENSRNOSNP2784920	gko-50a8_rp2_b1_695	233456702	A	T	A	A	A	T	A	A	T	A	A	A	T	A	T	T	T	T	T	T	T	T
ENSRNOSNP2784921	gko-40k10_fp2_b1_260	233807677	G	-	T	T	T	T	T	T	T	T	T	T	T	T	T	T	-	T	-	T	T	T
ENSRNOSNP2784922	J499954	233807884	T	C	C	C	C	C	C	C	C	C	C	C	C	C	C	C	C	C	C	C	C	C
ENSRNOSNP2784923	WKYc72c12_r1_398	233971162	G	G	A	A	A	G	A	A	G	G	G	G	G	A	G	G	G	G	G	G	G	G
ENSRNOSNP2784924	J516680	234062665	C	T	T	T	T	T	T	T	T	T	T	T	T	T	T	T	T	T	-	T	T	T
ENSRNOSNP2784925	J571373	234276833	G	A	G	G	G	A	G	G	A	A	A	A	A	G	A	A	A	A	-	A	A	A
ENSRNOSNP2784926	J341308	234468552	T	C	T	T	T	C	T	T	C	T	T	T	C	T	C	C	C	C	C	C	C	C
ENSRNOSNP2784927	WKYc18f07_s1_241	234501701	C	C	T	T	T	C	T	T	C	C	C	C	C	T	C	C	C	C	-	C	C	C
ENSRNOSNP2784928	J684773	234601040	C	C	A	A	A	C	A	A	C	A	A	A	C	A	C	C	C	C	-	C	C	C
ENSRNOSNP2784929	gko-66e1_rp2_b1_264	234642714	G	A	G	G	G	A	G	G	A	A	A	A	A	G	A	A	A	A	A	A	A	A
ENSRNOSNP2784930	SHRSPa19g04_r1_294	234782979	A	C	A	A	A	C	A	A	C	A	A	A	C	A	C	C	C	C	C	C	C	C
ENSRNOSNP2784931	J484979	234842302	A	G	A	A	A	G	A	A	G	A	A	A	G	A	G	G	G	G	-	G	G	G
ENSRNOSNP2784932	WKYc86c12_s1_261	235038478	G	C	C	C	C	C	C	C	C	C	C	C	C	C	C	C	C	C	C	C	C	C
ENSRNOSNP2784933	gko-76k22_rp2_b1_340	235202746	G	T	G	G	G	T	G	G	T	G	G	G	T	G	T	T	-	T	T	T	T	T
ENSRNOSNP2784934	J550349	235229275	A	T	A	A	A	T	A	A	T	A	A	A	T	A	T	T	T	T	-	T	T	T
ENSRNOSNP2784935	SHRSPa63f03_s1_397	235392218	C	G	G	G	G	G	G	G	G	G	G	G	G	G	G	G	G	G	G	G	G	G
ENSRNOSNP2784936	J342524	235498195	T	C	T	T	T	C	T	T	C	T	T	T	C	T	C	C	C	C	C	C	C	C
ENSRNOSNP2784937	gko-27k6_rp2_b1_579	235599177	T	G	G	G	G	G	G	G	G	T	-	T	G	G	G	G	G	G	G	G	G	G
ENSRNOSNP2784938	WKYc04a09_s1_360	235763841	G	G	A	A	A	G	A	A	G	G	G	G	G	A	G	G	G	G	G	G	G	G
ENSRNOSNP2784939	J1275901	235990207	T	T	G	G	G	T	G	T/G	T	T	T	T	T	G	T	T	T	T	T	T	T	T
ENSRNOSNP2784940	J341208	236108183	G	A	G	G	G	A	G	G	A	G	G	G	A	G	A	A	A	A	-	A	A	A
ENSRNOSNP2784941	Cpn_1236133599	236133599	C	T	C	C	C	T	C	C	T	C	C	C	T	C	T	T	T	T	T	T	T	T
ENSRNOSNP2784942	SHRSPc21h11_s2_310	236180503	C	T	C	C	C	T	C	C	T	C	C	C	T	C	T	T	T	T	T	T	T	T
ENSRNOSNP2784943	J583392	236239752	A	G	G	G	G	G	G	G	G	G	G	G	G	G	G	G	G	G	-	G	G	G
ENSRNOSNP2784944	rat110_003_n14.q1ca_426	236396526	G	T	T	T	T	T	T	T	T	G	G	G	T	T	T	T	T	T	T	G	G	G
ENSRNOSNP2784945	WKY-G-i-30d10_f1_128	236599476	A	G	G	G	G	G	G	G	G	-	G	G	G	G	G	G	G	G	G	G	G	G
ENSRNOSNP2784946	rat108_013_l14.p1ca_196	236683656	T	C	C	C	C	C	C	C	C	C	C	C	C	C	C	C	C	C	C	C	C	C
ENSRNOSNP2784947	J596533	236793809	A	A	A	A	A	A	A	A	-	C	C	C	-	A	A	A	A	A	A	A/C	C	A/C
ENSRNOSNP2784948	WKY-G-f-15a08_f1_295	236899082	A	G	G	G	G	G	G	G	G	G	G	G	G	G	G	G	G	G	G	G	G	G
ENSRNOSNP2784949	SHRSPa56g08_r1_197	236995052	C	T	T	T	T	T	T	T	T	C	C	C	T	T	T	T	T	T	T	C	C	C

ENSRNOSNP2784950	J563567	237021546	C	C	C	C	C	C	C	C	C	T	T	T	C	C	C	C	C	C	-	T	T	T
ENSRNOSNP2784951	gko-97h11_rp2_b1_354	237148023	C	A	C	C	C	A	C	C	A	A	A	A	A	C	A	A	A	A	A	A	A	A
ENSRNOSNP2784952	J491517	237383999	C	T	T	T	T	T	T	T	T	T	T	T	T	T	T	T	T	T	-	T	T	T
ENSRNOSNP2784953	gko-104o17_fp2_b1_508	237469738	T	C	C	C	C	C	C	C	C	C	C	C	C	C	C	C	C	C	C	C	C	C
ENSRNOSNP2784954	WKYe09a09_r1_136	237503621	T	C	C	C	C	C	C	C	C	C	C	C	C	C	C	C	C	C	C	C	C	C
ENSRNOSNP2784955	WKY-G-i-44f06_f1_1430	237835559	C	A	A	A	A	A	A	A	A	A	A	A	A	A	A	A	A	A	A	A	A	A
ENSRNOSNP2784956	J488006	237910637	T	T	T	T	T	T	T	T	T	C	C	C	T	T	T	T	T	T	T	TC	C	TC
ENSRNOSNP2784957	gko-27e7_rp2_b1_105	237978144	A	A	A	A	A	A	A	A	A	C	C	C	A	A	A	A	A	A	A	C	C	C
ENSRNOSNP2784958	J572328	238479524	A	A	A	A	A	A	A	A	A	T	T	T	A	A	A	A	A	A	-	T	T	T
ENSRNOSNP2784959	rat104_013_k22.q1ca_686	238861117	C	A	A	A	A	A	A	A	A	A	A	A	A	A	A	A	A	A	A	A	A	A
ENSRNOSNP2784960	SHRSPc64a05_r1_121	238892832	G	T	T	T	T	T	T	T	T	T	T	T	T	T	T	T	T	T	T	T	T	T
ENSRNOSNP2784961	J598326	239048791	C	T	T	T	T	T	T	T	T	T	T	T	T	T	T	T	T	T	-	T	T	T
ENSRNOSNP2784962	gko-108f14_rp2_b1_311	239048926	A	G	G	G	G	G	G	G	G	G	G	G	G	G	G	G	G	G	G	G	G	G
ENSRNOSNP2784963	J342461	239161787	C	A	A	A	A	A	A	A	A	A	A	A	A	A	A	A	A	A	-	A	A	A
ENSRNOSNP2784964	SHRSPc10g01_s2_205	239812380	G	A	A	A	A	A	A	A	A	G	G	G	A	A	A	A	A	A	A	G	G	G
ENSRNOSNP2784965	rat101_015_m05.p1ca_544	240135307	G	A	A	A	A	A	A	A	A	A	A	A	A	A	A	A	A	A	A	A	A	A
ENSRNOSNP2784966	J1300459	240425135	A	G	G	G	G	G	G	G	G	A	A	A	G	G	G	G	G	G	-	A	A	A
ENSRNOSNP2784967	gko-18c21_rp2_b1_62	240506905	C	C	C	C	C	C	C	CT	C	T	T	T	C	C	C	C	C	C	C	T	T	T
ENSRNOSNP2784968	J474918	240576215	A	T	T	T	T	T	T	T	T	T	T	T	T	T	T	T	T	T	T	T	T	T
ENSRNOSNP2784969	WKYOa42g04_r1_262	240673620	G	T	T	T	T	T	T	T	T	T	T	T	T	T	T	T	T	T	T	T	T	T
ENSRNOSNP2784970	SHRSPc10b09_s2_124	240766152	G	A	A	A	A	A	A	A	A	G	G	G	A	A	A	A	A	A	A	G	G	G
ENSRNOSNP2784971	rat104_028_b17.q1ca_305	240839725	C	T	T	T	T	T	T	T	T	C	C	C	T	T	T	T	T	T	T	C	C	C
ENSRNOSNP2784972	SHRSPc52e03_r1_432	240928043	G	A	A	A	A	A	A	A	A	G	G	G	A	A	A	A	A	A	A	G	G	G
ENSRNOSNP2784973	Cpn_1240992368	240992368	C	T	T	T	T	T	T	T	T	T	T	T	T	T	T	T	T	T	T	T	T	T
ENSRNOSNP2784975	Cpn_1241639040	241639040	T	T	T	T	T	T	T	T	T	C	C	C	T	T	T	T	T	T	T	C	-	-
ENSRNOSNP2784976	WKYc72b11_s1_461	242263558	T	A	A	A	A	A	A	A	A	A	A	A	A	A	A	A	A	A	A	A	A	A
ENSRNOSNP2784977	J697156	242329444	C	C	C	C	C	C	C	C	C	T	T	T	C	C	C	C	C	C	-	T	T	T
ENSRNOSNP2784978	J503759	242418966	G	G<br>A	G	-	G	G<br>A	-	-	G<br>A	A	A	A	G	-	-	G	G<br>A	G<br>A	-	A	A	A
ENSRNOSNP2784979	Cpn_1242637435	242444039	G	G	G	G	G	G	G	G	G	G	G	G	G	G	G	G	G	G	G	G	G	G
ENSRNOSNP2784980	J595364	242551679	T	T	T	T	T	T	T	T	T	C	C	C	T	T	T	T	T	T	-	C	C	C
ENSRNOSNP2784981	gko-67a10_rp2_b1_301	242599651	T	T	T	T	T	T	T	T	T	A	A	A	T	T	T	T	T	T	T	A	A	A
ENSRNOSNP2784982	J551759	243881365	C	C	C	-	C	C	-	C	C	C	C	C	C	-	C	C	C	C	-	CT	C<br>T	CT
ENSRNOSNP2784983	J655226	244519462	A	A	A	A	A	A	A	A	A	T	T	T	A	A	A	A	A	A	-	A	A	A
ENSRNOSNP2784984	J632995	244621903	T	T	T	-	T	T	-	T	T	C	C	C	T	-	T	T	T	T	-	T	T	T
ENSRNOSNP2784985	J468480	244723523	G	G	G	G	G	G	G	G	G	A	A	A	G	G	G	G	G	G	-	A	A	A
ENSRNOSNP2784986	Cpn_1245301605	245301605	T	T	T	T	T	T	TC	T	T	C	C	C	T	T	T	T	T	T	T	C	C	C
ENSRNOSNP2784987	J671394	245348611	G	G<br>C	G	G	G	G<br>C	G	-	G<br>C	G	G	G	-	G	-	G	-	G<br>C	G	G<br>C	G	G
ENSRNOSNP2784988	Cpn_1245350235	245350235	A	A	A	A	A	A	A	A	A	A	A	A	A	A	A	A	A	A	A	A	A	A
ENSRNOSNP2784989	rat109_024_e21.q1ca_332	245413465	A	C	C	C	C	C	C	C	C	A	A	A	C	C	C	C	C	C	C	A	A	A

ENSRNOSNP2784990	rat102_001_b02.p1ca_544	245418191	T	C	C	C	C	C	C	C	C	C	C	C	C	C	C	C	C	C	C	T	T	T
ENSRNOSNP2784991	Cpn_1245418564	245418564	C	-	T	T	C<br>T	T	T	T	T	C	C	C	T	-	T	T	CT	T	C<br>T	C	C	C
ENSRNOSNP2784992	rat102_018_o15.p1ca_117	245418921	G	T	T	T	T	T	T	T	T	G	G	G	T	T	T	T	T	T	T	G	G	G
ENSRNOSNP2784993	rat108_001_j01.q1ca_703	245686574	T	C	C	C	C	C	C	C	C	C	C	C	C	C	C	C	C	C	C	T	T	T
ENSRNOSNP2784994	SHRSPc08h12_r1_470	245691446	A	G	G	G	G	G	G	G	G	A	A	A	G	G	G	G	G	G	G	A	-	-
ENSRNOSNP2784995	rat108_031_b04.q1ca_198	245725606	A	G	G	G	G	G	G	G	G	G	G	G	G	G	G	G	G	G	G	A	A	A
ENSRNOSNP2784996	J1305629	245763299	A	G	G	G	G	G	G	G	G	G	G	G	G	G	G	G	G	G	G	A	A	A
ENSRNOSNP2784997	J682298	245886655	G	G	G	G	G	G	G	G	G	G	G	G	G	G	G	G	G	G	-	A	A	A
ENSRNOSNP2784998	SHRSPc21h03_r1_724	245924574	T	C	C	C	C	C	C	C	C	C	C	C	C	C	C	C	C	C	C	T	T	T
ENSRNOSNP2784999	WKYc21c09_s1_747	246010144	G	A	A	A	A	A	A	A	A	A	-	A	A	A	A	A	A	A	A	A	A	A
ENSRNOSNP2785000	J691977	246033390	G	G	G	G	G	G	G	G	G	G	G	G	G	G	G	G	G	G	-	A	A	A
ENSRNOSNP2785001	J562377	246145270	G	G<br>C	G	-	G	-	-	-	G<br>C	G	G	G	-	-	-	G	-	G<br>C	-	G<br>C	G	G
ENSRNOSNP2785002	SHRSPc11g10_s2_236	246189919	T	C	C	C	C	C	C	C	C	C	C	C	C	C	C	C	C	C	C	T	T	T
ENSRNOSNP2785003	WKYe11f03_r1_428	246261698	A	G	G	G	G	G	G	G	G	G	G	G	G	G	G	G	G	G	G	A	A	A
ENSRNOSNP2785004	rat110_014_i18.p1ca_405	246412724	G	A	A	A	A	A	A	A	A	A	A	A	A	A	A	A	A	A	A	G	G	G
ENSRNOSNP2785005	J654209	246456731	A	A	A	A	A	A	A	A	A	A	A	A	A	A	A	A	A	A	A	T	T	T
ENSRNOSNP2785006	DS-g-a-37c04_f1_201	246504030	G	G	G	G	G	G	G	G	G	G	G	G	G	G	G	G	G	G	G	A	A	A
ENSRNOSNP2785007	rat106_020_a13.q1ca_430	246615233	C	C	C	C	C	C	C	C	C	C	C	C	C	C	C	C	C	C	C	A	A	A
ENSRNOSNP2785008	rat106_020_a13.p1ca_77	246615541	G	G	G	G	G	G	G	G	G	G	G	G	G	G	G	G	G	G	G	A	A	A
ENSRNOSNP2785009	J558948	246623550	C	C	C	C	C	C	C	C	C	C	C	C	C	C	C	C	C	C	-	T	T	T
ENSRNOSNP2785010	gko-71c18_fp2_b1_522	246757144	G	G	G	G	G	G	G	G	G	G	G	G	G	G	G	G	G	G	G	A	A	A
ENSRNOSNP2785011	J583803	246758778	G	G	G	G	G	G	G	G	G	G	G	G	G	G	G	G	G	G	G	A	A	A
ENSRNOSNP2785012	rdahl-79m5_fp2_b1_231	246906744	T	T	T	T	T	T	T	T	T	T	T	T	T	T	T	T	T	T	T	C	C	C
ENSRNOSNP2785013	J641134	247068095	T	C	C	C	C	C	C	C	C	C	C	C	C	C	C	C	C	C	C	C	C	C
ENSRNOSNP2785014	rat106_005_o04.p1ca_742	247093374	A	-	-	-	-	-	-	A<br>G	G	G	G	G	G	G	G	G	G	G	G	G	G	G
ENSRNOSNP2785015	rat101_017_o02.q1ca_293	247171067	G	A	A	A	A	A	A	A	A	A	A	A	A	A	A	A	A	A	A	G	G	G
ENSRNOSNP2785016	J483648	247172158	C	C	C	C	C	C	C	C	C	C	C	C	C	C	C	C	C	C	-	T	T	T
ENSRNOSNP2785017	J469697	247326999	T	C	C	-	C	C	-	C	C	C	C	C	C	-	C	C	C	C	-	C	C	C
ENSRNOSNP2785018	Cpn_1247347259	247347259	A	G	G	G	G	G	G	G	G	G	G	G	G	G	G	G	G	G	G	G	G	G
ENSRNOSNP2785019	rat101_010_m22.q1ca_510	247350344	A	G	G	G	G	G	G	G	G	G	G	G	G	G	G	G	G	G	G	A	A	A
ENSRNOSNP2785020	SHRSPc30g08_r1_304	247403219	C	T	T	T	T	T	T	T	T	T	T	T	T	T	T	T	T	T	T	C	C	C
ENSRNOSNP2785021	gko-69a5_rp2_b1_122	247638201	G	G	G	G	G	G	G<br>A	G	G	G	G	G	G	G	G	G	G	G	G	A	A	A
ENSRNOSNP2785022	J592374	247736591	T<br>A	A	A	-	A	A	-	A	A	A	A	A	A	-	A	A	A	A	-	A	A	A
ENSRNOSNP2785023	WKYc95d02_s1_305	247817588	T	C	C	C	C	C	C	C	C	C	C	C	C	C	C	C	C	C	C	C	C	C
ENSRNOSNP2785024	J696336	247929983	T	C	C	C	C	C	C	C	C	C	C	C	C	C	C	C	C	C	-	C	C	C
ENSRNOSNP2785025	gko-69a22_rp2_b1_673	247958585	A	G	G	G	G	G	G	G	G	G	G	G	G	G	G	G	G	G	G	G	G	G
ENSRNOSNP2785026	Cpn_1248118570	248118570	A	A	A	A	A	A	A	A	A	A	A	A	A	A	A	A	A	A	A	G	G	G
ENSRNOSNP2785027	rat105_030_k01.q1cb_180	248121772	C	C	C	C	C	C	C	C	C	C	C	C	C	C	C	C	C	C	C	T	T	T
ENSRNOSNP2785028	rat107_019_h02.q1ca_371	248140473	C	C	C	C	C	C	C	C	C	C	C	C	C	C	C	C	C	C	C	A	A	A

ENSRNOSNP2785029	rat110_018_e16.p1cb_311	248149537	C	T	T	C<br>T	T	T	CT	T	T	T	T	T	T	CT	T	T	T	T	C<br>T	C	C	C
ENSRNOSNP2785030	Cpn_1248162056	248162056	G	A	A	A	A	A	A	A	A	A	A	A	A	A	A	A	A	A	A	G	G	G
ENSRNOSNP2785031	J691737	248247899	T	T	T	-	T	T	-	T	T	T	T	T	T	-	T	T	T	T	-	TC	C	C
ENSRNOSNP2785032	WKYc35e06_s2_228	248279989	A	G	G	G	G	G	G	G	G	G	G	G	G	G	G	G	G	G	G	G	G	G
ENSRNOSNP2785033	SHRSPc30a05_s1_498	248363805	G	C	C	C	C	C	C	C	C	C	C	C	C	C	C	C	C	C	C	C	C	C
ENSRNOSNP2785034	J498212	248416578	T	C	C	C	C	C	C	C	C	C	C	C	C	C	C	C	C	C	-	C	C	C
ENSRNOSNP2785035	gnl|ti|896832643_19866867090830_285	248437877	A	A<br>G	A	A	A	A	A	A	A	A	A	A	-	A	A	-	A	A	A	G	G	G
ENSRNOSNP2785036	J1266991	248582237	A	G	G	G	G	G	G	G	G	G	G	G	G	G	G	G	G	G	G	G	G	G
ENSRNOSNP2785037	Cpn_1248660645	248660645	G	A	A	A	A	A	A	A	A	A	A	A	A	A	A	A	A	A	A	G	G	G
ENSRNOSNP2785038	J478743	248707446	C	C	C	C	C	C	C	C	C	C	C	C	C	C	C	C	C	C	-	G	G	G
ENSRNOSNP2785039	Cpn_1248736424	248736424	G	G	G	G	G	G	G	G	G	G	G	G	G	G	G	G	G	G	G	A	A	A
ENSRNOSNP2785040	J551489	249108630	T	G	G	G	G	G	G	G	G	G	G	G	G	G	G	G	G	G	G	G	G	G
ENSRNOSNP2785041	SHRSPc58a10_r1_755	249114531	A	G	G	G	G	G	G	G	G	G	G	G	G	G	G	G	G	G	-	G	G	G
ENSRNOSNP2785042	rat107_035_e20.q1ca_550	249157927	G	G	G	G	G	G	G	G	G	G	G	G	G	G	G	G	G	G	G	A	A	A
ENSRNOSNP2785043	rat110_008_m06.p1cb_275	249210876	A	G	G	G	G	G	G	G	G	G	G	G	G	G	G	G	G	G	G	A	A	A
ENSRNOSNP2785044	Cpn_1249216631	249216631	A	G	G	G	G	G	G	G	G	G	G	G	G	G	G	G	G	G	G	G	G	G
ENSRNOSNP2785046	WKYc12g07_s1_716	249231645	C	T	T	T	T	T	T	T	T	T	T	T	T	T	T	T	T	T	T	C	C	C
ENSRNOSNP2785047	J640129	249360014	G	G	G	G	G	G	G	G	A	A	A	A	A	A	A	A	A	A	-	G	G	G
ENSRNOSNP2785048	rat108_043_m03.p1ca_450	249369521	C	C	C	C	C	C	C	C	T	T	T	T	T	T	T	T	T	T	T	T	T	T
ENSRNOSNP2785049	WKYc45d03_s1_454	249482081	G	A	A	A	A	A	A	A	G	G	G	G	G	G	G	G	G	G	G	G	G	G
ENSRNOSNP2785050	J552100	249599183	C	-	C	-	C	-	-	-	T	T	T	T	T	-	T	T	T	T	-	T	T	T
ENSRNOSNP2785051	rat101_023_h20.p1ca_291	249618804	T	C	C	C	C	C	C	C	T	T	T	T	T	C	T	T	T	T	T	T	T	T
ENSRNOSNP2785052	SHRSPc37f09_r1_729	249675076	G	G	G	G	G	G	G	G	A	A	A	A	A	G	A	A	A	A	A	A	A	A
ENSRNOSNP2785053	SHRSPc22h10_s1_756	249849309	G	G	G	G	G	G	G	G	A	A	A	A	A	A	A	A	A	A	A	A	A	A
ENSRNOSNP2785054	J534118	249899504	A	A	A	A	A	A	A	A	A	A	A	A	A	A	A	A	A	A	A	G	G	G
ENSRNOSNP2785055	SHRSPc63e12_r1_708	249940523	C	T	T	T	T	T	T	T	T	T	T	T	T	T	T	T	T	T	T	T	T	T
ENSRNOSNP2785056	J677619	250015295	A	A	A	A	A	A	A	A	A	A	A	A	A	A	A	A	A	A	-	G	G	G
ENSRNOSNP2785057	rat103_029_c10.p1ca_163	250068468	C	C	C	C	C	C	C	C	C	C	C	C	C	C	C	C	C	C	C	T	T	T
ENSRNOSNP2785058	gnl|ti|842492887_50135814_337	250090066	A	A	A	A	A	G	A	A	G	G	G	G	G	G	G	G	G	G	G	G	G	G
ENSRNOSNP2785059	SHRSPc24a04_r1_643	250192242	T	T	T	T	T	C	T	T	C	C	C	C	C	C	C	C	C	C	-	C	C	C
ENSRNOSNP2785060	SHRSPc42a09_s1_245	250588452	G	A	A	A	-	A	A	A	A	A	A	A	A	A	A	A	A	A	A	A	A	A
ENSRNOSNP2785061	rat104_052_a11.q1ca_648	250718525	G	A	A	A	A	A	A	A	A	A	A	A	A	A	A	A	A	A	A	A	A	A
ENSRNOSNP2785062	rat108_014_c15.q1ca_222	250982839	G	G	G	G	G	C	G	G	C	C	C	C	C	C	C	C	C	C	C	G	G	G
ENSRNOSNP2785063	Cpn_1251008997	251008997	A	A	A	-	A	A	A	A	A	A	A	A	A	A	A	A	A	A	A	G	G	G
ENSRNOSNP2785064	J592320	251019250	C	C	C	C	C	C	C	C	C	C	C	C	C	C	C	C	C	C	-	T	T	T
ENSRNOSNP2785065	Cpn_1251356420	251356420	C	C	C	C	C	C	C	C	C	C	C	C	C	C	C	C	C	C	C	T	T	T
ENSRNOSNP2785066	Cpn_1251519520	251519520	A	A	A	A	A	A	A	A	A	A	A	A	A	A	A	A	A	A	A	C	C	C
ENSRNOSNP2785067	rat108_037_c06.p1ca_716	251546440	C	C	C	C	C	G	C	C	G	G	G	G	G	G	G	G	G	G	G	G	G	G
ENSRNOSNP2785068	J481278	251645526	T	A	A	A	A	A	A	A	A	A	A	A	A	A	A	A	A	A	A	A	A	A

ENSRNOSNP2785069	rat108_035_e01.q1ca_144	251755814	T	C	C	C	C	C	C	C	C	C	C	C	C	C	C	C	C	C	C	C	C	C
ENSRNOSNP2785070	WKYc75c11_r1_62	251758852	A	T	T	T	T	T	T	T	T	T	T	T	T	T	T	T	T	T	T	T	T	T
ENSRNOSNP2785071	J556031	251876667	T	C	C	C	C	C	C	C	C	C	C	C	C	C	C	C	C	C	-	C	C	C
ENSRNOSNP2785072	gko-107d17_fp2_b1_464	251919957	G	G	G	G	G	A	G	G	A	A	A	A	A	A	A	A	A	A	A	A	A	A
ENSRNOSNP2785073	J668614	251982463	T	C	C	C	C	T	C	C	T	T	T	T	T	T	T	T	T	T	T	T	T	T
ENSRNOSNP2785074	J572842	252075188	C	C	C	C	C	T	C	C	T	T	T	T	T	T	T	T	T	T	-	T	T	T
ENSRNOSNP2785075	J651201	252181230	T	C	C	C	C	C	C	C	C	C	C	C	C	C	C	C	C	C	-	C	C	C
ENSRNOSNP2785076	Cpn_1252197077	252197077	T	C	C	C	C	T	C	C	T	T	T	-	T	T	T	T	T	T	T	T	-	-
ENSRNOSNP2785077	rat101_037_e13.p1ca_191	252201419	A	G	G	G	G	G	G	G	G	G	G	G	G	G	G	G	G	G	G	G	G	G
ENSRNOSNP2785079	rat101_001_o12.q1ca_300	252453603	G	T	T	T	T	G	T	T	G	G	G	G	G	G	G	G	G	G	G	G	G	G
ENSRNOSNP2785080	J877804	252460516	A	C	C	-	C	A	-	C	A	A	A	A	A	A	A	A	A	A	-	A	A	A
ENSRNOSNP2785081	SHRSPc08c09_r1_712	252511949	C	G	G	G	G	G	G	G	G	G	G	G	G	G	G	G	G	G	G	G	G	G
ENSRNOSNP2785082	WKYOa55f10_s1_199	252657633	G	A	A	-	A	G	A	A	G	G	G	G	G	G	G	-	G	G	G	G	G	G
ENSRNOSNP2785083	J522384	252661969	G	G	G	G	G	A	G	G	A	A	A	G<br>A	A	A	A	A	A	A	A	A	A	A
ENSRNOSNP2785084	J1271521	252779708	T	C	C	C	C	T	C	C	T	T	T	T	T	T	T	T	T	T	-	T	T	T
ENSRNOSNP2785085	Cpn_1252871262	252871262	C	T	T	T	T	T	T	T	T	T	T	T	T	T	T	T	T	T	T	T	T	T
ENSRNOSNP2785086	rat104_016_g10.q1ca_264	252875633	G	A	A	A	A	G	A	A	G	G	G	G	G	G	G	G	G	G	G	G	G	G
ENSRNOSNP2785087	rat013_054_i07.q1ca_232	252985998	G	C	C	C	C	G	C	C	G	G	G	G	G	G	G	G	G	G	G	G	G	G
ENSRNOSNP2785088	WKYc107b12_s1_229	252995488	G	A	A	A	A	A	A	A	A	A	A	-	A	A	A	A	A	A	A	A	A	A
ENSRNOSNP2785089	J596912	253011626	C	C	C	C	C	T	C	T	T	T	T	T	T	T	T	T	T	T	T	T	T	T
ENSRNOSNP2785091	SHRSPa25g10_s1_775	253246042	G	G	G	G	G	T	G	T	T	T	T	T	T	T	T	T	T	T	T	T	T	T
ENSRNOSNP2785092	gko-6d5_rp2_b1_412	253423962	A	A	A	A	A	C	A	C	C	C	C	C	C	C	C	C	C	C	C	C	C	C
ENSRNOSNP2785093	J649473	253643590	T	T	T	-	T	T	-	T	T	T	T	T	T	T	T	T	T	T	T	T	T	T
ENSRNOSNP2785094	rdahl-33d6_fp2_b1_205	253644637	T	T	T	T	T	C	T	C	C	C	C	C	C	C	C	C	C	C	C	C	C	C
ENSRNOSNP2785095	J1269649	253806438	C	G	G	G	G	G	G	G	G	G	G	G	G	G	G	G	G	G	-	G	G	G
ENSRNOSNP2785096	SHRSPc62h12_s1_272	254209324	T	T	T	T	-	C	T	C	C	C	C	C	C	C	C	C	C	C	C	T	T	T
ENSRNOSNP2785097	gnl|ti|896700629_19866867954014_268	254514369	A	A	A	A	A	A	A	T	A	A	A	A	A	A	A	A	A	A	A	A	A	A
ENSRNOSNP2785098	J677586	254550790	C	C	C	C	C	A	C	A	A	A	A	A	A	A	A	A	A	A	-	C	C	C
ENSRNOSNP2785099	J636317	254715527	A	T	T	T	T	A	T	T	A	A	A	A	A	A	A	A	A	A	A	T	T	T
ENSRNOSNP2785100	SHRSPc60h06_s1_517	254824212	C	C	C	C	C	C	C	T	T	T	T	T	C	T	T	T	T	T	T	C	C	C
ENSRNOSNP2785101	J1264254	254908544	G	A	A	A	A	G	A	A	A	A	A	A	A	A	A	A	A	A	-	A	A	A
ENSRNOSNP2785102	gnl|ti|896616272_19866867247325_375	255259646	G	G	G	G	G	G	G	A	G	G	G	G	A	G	G	G	G	G	G	G	G	G
ENSRNOSNP2785103	J1273692	255362995	T	C	C	C	C	C	C	C	C	C	C	C	C	C	C	C	C	C	C	T	T	T
ENSRNOSNP2785104	J1273298	255666505	A	G	G	G	G	G	G	G	A	A	A	A	A	A	A	A	A	A	A	G	G	G
ENSRNOSNP2785105	gko-50g15_rp2_b1_254	255707762	A	A	A	A	A	A	A	G	A	A	A	-	A	A	A	A	A	A	A	G	G	G
ENSRNOSNP2785106	J1266955	255757909	A	G	G	G	G	G	G	G	G	G	G	G	G	G	G	G	G	G	-	G	G	G
ENSRNOSNP2785107	rat101_015_o06.p1cb_144	255765717	C	T	T	T	T	T	T	T	T	T	T	T	C	T	T	T	T	T	T	C	C	C
ENSRNOSNP2785108	J664720	255885332	G	A	A	A	A	A	A	A	A	A	A	A	A	A	A	A	A	A	-	G	G	G
ENSRNOSNP2785109	WKYc95b02_s1_448	255933254	A	G	G	G	G	G	G	G	G	G	G	G	G	G	G	G	G	G	G	A	A	A

ENSRNOSNP2785110	J568085	255998182	T	T	T	T	T	T	T	C	T	T	T	T	T	T	T	T	T	T	T	C	C	C
ENSRNOSNP2785111	WKYc96h01_s1_143	256049463	C	T	T	T	T	T	T	T	T	T	T	T	C	T	T	T	T	T	T	T	T	T
ENSRNOSNP2785112	Cpn_1256081651	256081651	A	C	C	C	C	C	C	C	C	C	C	C	C	C	C	C	C	-	C	A	A	A
ENSRNOSNP2785113	J598123	256102412	T	T	T	T	T	T	T	A	T	T	T	T	T	T	T	T	T	T	-	A	A	A
ENSRNOSNP2785114	J529527	256341785	A	A	A	-	T	A T	-	T	A T	T	T	T	A T	-	A T	T	A T	A T	-	A T	T	A T
ENSRNOSNP2785115	WKYc28h03_r1_466	256345221	A	A	A	A	C	C	A	C	C	C	C	C	C	C	C	C	C	C	C	C	C	C
ENSRNOSNP2785116	WKYOa52g07_s1_155	256566508	T	C	C	C	C	C	C	C	C	C	C	C	C	C	C	-	C	C	C	C	C	C
ENSRNOSNP2785117	J536037	256651378	T	T	T	T	T	T	T	A	T	T	T	T	T	T	T	T	T	T	T	A	A	A
ENSRNOSNP2785118	WKYe12f08_s1_142	256694017	A	A	A	A	G	G	A	G	A	A	-	A	A	A	A	A	A	A	A	G	G	G
ENSRNOSNP2785119	J477674	256775981	G	A	A	A	A	A	A	A	A	A	A	A	A	A	A	A	A	A	-	A	A	A
ENSRNOSNP2785120	WKYc74c06_s1_792	256807760	T	T	T	T	C	C	T	C	T	T	T	T	T	T	T	T	T	T	T	T	T	T
ENSRNOSNP2785121	gnl|ti|896487007_19866867643685_278	256985121	T	T	T	T	G	G	T	G	T	T	T	T	G	T	T	T	T	T	T	T	T	T
ENSRNOSNP2785122	J549695	257092128	G	A	A	A	A	A	A	A	A	A	A	A	A	A	A	A	A	A	A	A	A	A
ENSRNOSNP2785123	J563204	257302761	T	T	T	T	T	T	T	C	T	T	T	T	T	T	T	T	T	T	-	C	C	C
ENSRNOSNP2785124	WKYOa11c05_s1_415	257307659	A	A	A	A	G	G	A	G	A	A	A	A	G	A	A	A	A	A	A	A	A	A
ENSRNOSNP2785126	gnl|ti|896804887_19866867039419_252	257483034	A	A	A	A	A	A	A	C	A	A	A	A	C	A	A	A	A	A	A	C	C	C
ENSRNOSNP2785127	J548815	257517511	G	G	G	G	A	A	G	A	G	G	G	G	A	G	G	G	G	G	-	A	A	A
ENSRNOSNP2785128	gnl|ti|896696792_19866868286899_289	257629363	T	G	G	G	-	T	G	G	G	G	G	G	G	G	G	G	G	G	G	G	G	G
ENSRNOSNP2785129	J657133	257758658	C	T	T	T	T	T	T	T	T	T	T	T	T	T	T	T	T	T	T	C	C	C
ENSRNOSNP2785130	gko-103i3_fp2_b1_461	257812633	G	G A	A	A	G	G	A	A	G	G	G	G	G	G	G	G	G	G	G	A	A	A
ENSRNOSNP2785131	J658290	257937532	T	G	G	-	G	G	-	G	G	G	G	G	G	-	G	G	G	G	-	G	G	G
ENSRNOSNP2785132	gko-85n6_fp2_b1_424	257951523	A	G	G	G	G	G	G	G	G	G	G	G	G	G	G	G	G	G	G	G	G	G
ENSRNOSNP2785133	J1287994	258032919	A	A	A	-	G	G	-	G	A	A	A	A	A	-	A	A	A	A	-	A	A	A
ENSRNOSNP2785134	rdahl-97n21_fp2_b1_260	258097849	G	A	A	A	G	G	A	A	G	G	G	G	G	G	G	-	G	G	G	G	G	G
ENSRNOSNP2785135	J537014	258160379	G	T	T	T	T	T	T	T	G	G	G	G	T	-	G	G	G	G	-	T	T	T
ENSRNOSNP2785136	rdahl-74p19_fp2_b1_808	258250123	C	T	T	T	C	C	T	T	T	T	T	T	T	T	T	T	T	T	T	C	C	C
ENSRNOSNP2785137	gnl|ti|896984663_19866867936401_360	258395004	C	A	A	A	A	A	A	A	A	A	A	A	C	A	A	A	A	A	A	A	A	A
ENSRNOSNP2785138	rdahl-19j5_fp2_b1_660	258533775	G	T	T	T	G	G	T	T	T	T	T	T	T	T	T	T	T	T	T	G	G	G
ENSRNOSNP2785139	J589089	258565344	A	G	G	G	A	A	G	G	G	G	G	G	A	G	G	G	G	G	G	G	G	G
ENSRNOSNP2785140	WKY-G-i-24c09_f1_1296	258949177	A	A	A	A	C	C	A	C	A	A	A	A	A	A	A	A	A	A	A	A	A	A
ENSRNOSNP2785141	gnl|ti|842493205_50149288_295	259115635	G	G A	A	A	G	G	A	A	G	G	G	G	A	G	G	G	G	G	G	G	G	G
ENSRNOSNP2785142	J649333	259303930	G	G	G	G	A	A	G	A	G	G	G	G	G	G	G	G	G	G	G	A	A	A
ENSRNOSNP2785143	WKYc96g09_s1_647	259431003	G	G	G	G	C	C	G	C	G	G	G	G	G	G	G	G	G	G	G	G	G	G
ENSRNOSNP2785144	J684532	259466027	A	C	C	C	C	C	C	C	C	C	C	C	C	C	C	C	C	C	-	A	A	A
ENSRNOSNP2785145	gko-85d23_fp2_b1_704	259613114	G	G	G	G	A	A	G	A	G	G	G	G	G	G	G	G	G	G	G	A	A	A
ENSRNOSNP2785146	WKYc55f03_s1_57	259682909	C	T	T	-	T	T	T	T	T	T	T	T	T	T	T	T	T	T	T	T	T	T
ENSRNOSNP2785147	Cpn_1259757182	259757182	G	A	A	A	A	A	A	A	A	A	A	A	A	A	A	A	A	A	A	A	A	A
ENSRNOSNP2785148	J687411	259768068	G	G	G	G	G	G	G	A	G	G	G	G	G	G	G	G	G	G	-	G	G	G
ENSRNOSNP2785150	J543992	260120668	C	C	C	C	C	C	C	T	C	C	C	C	C	C	C	C	C	C	-	T	T	T

ENSRNOSNP2785151	gnl|ti|476330671_26627261_129	260202462	T	T	T	T	T	T	T	C	T	T	T	T	T	T	T	T	T	T	T	T	T	T
ENSRNOSNP2785152	J658707	260370013	C	T	T	T	C	C	T	T	T	T	T	T	C	T	T	T	T	T	T	C	C	C
ENSRNOSNP2785153	DS-g-a-15a02_f1_60	260371087	C	T	T	T	T	T	T	T	T	T	T	T	T	T	T	T	T	T	T	C	C	C
ENSRNOSNP2785154	SHRSPa9b08_r1_918	260544878	C	T	T	T	C	C	T	T	T	T	T	T	C	T	T	T	T	T	T	C	C	C
ENSRNOSNP2785155	J645488	260590833	C	T	T	T	C	C	T	T	T	T	T	T	C	T	T	T	T	T	-	C	C	C
ENSRNOSNP2785156	SHRSPc01h04_r1_546	260651657	G	C	C	C	C	C	C	C	C	C	C	C	C	C	C	C	C	C	C	G	G	G
ENSRNOSNP2785157	rdahl-62o10_fp2_b1_249	260951572	G	A	A	A	A	A	A	A	A	A	A	A	A	A	A	A	A	A	A	G	G	G
ENSRNOSNP2785158	WKY-G-i-21d02_f1_619	261096696	G	G	G	G	A	A	G	A	G	G	G	G	A	G	G	G	G	G	G	G	G	G
ENSRNOSNP2785159	J651012	261397288	G	A	A	A	G	G	A	A	A	A	A	A	G	A	A	A	A	A	A	G	G	G
ENSRNOSNP2785160	J676474	261489130	T	G	G	G	G	G	G	G	G	G	G	G	G	G	G	G	G	G	-	T	T	T
ENSRNOSNP2785161	WKYc110b11_s1_498	261569764	T	C	C	C	C	C	C	C	C	C	C	C	C	C	C	C	C	C	C	T	T	T
ENSRNOSNP2785162	gnl|ti|896624883_19866867208900_237	261746841	A	G	G	G	A	A	G	G	G	G	G	G	A	G	G	G	G	G	G	A	A	A
ENSRNOSNP2785163	gnl|ti|896489948_19866867495271_268	261897757	C	T	T	T	C	C	T	T	T	T	T	T	T	T	T	T	T	T	T	C	C	C
ENSRNOSNP2785164	gnl|ti|842492566_50138909_560	262057778	T	C	C	C	C	C	C	C	C	C	C	C	C	C	C	C	C	C	C	T	T	T
ENSRNOSNP2785165	J341558	262147178	C	T	T	T	C	C	T	T	T	T	T	T	T	T	T	T	T	T	T	C	C	C
ENSRNOSNP2785166	rat105_018_p20.p1ca_86	262225561	T	G	G	G	G	G	G	G	G	G	G	G	G	G	G	G	G	G	G	T	T	T
ENSRNOSNP2785167	J339670	262237876	C	T	T	T	T	T	T	T	T	T	T	T	C	T	T	T	T	T	-	C	C	C
ENSRNOSNP2785168	DS-g-a-04b09_r1_564	262373521	C	T	T	T	T	T	T	T	T	T	T	T	T	T	T	T	T	T	T	T	T	T
ENSRNOSNP2785169	rat107_011_n15.q1ca_156	262422913	G	A	A	-	G	G	-	A	A	A	A	A	G	-	A	A	A	A	-	G	G	G
ENSRNOSNP2785170	J546155	262491779	T	T	T	T	T	T	T	C	T	T	T	T	-	T	T	T	T	T	T	C	C	C
ENSRNOSNP2785171	rdahl-89m18_rp2_b1_144	262669943	A	G	G	G	G	G	G	G	G	G	G	G	G	G	G	G	G	G	G	A	A	A
ENSRNOSNP2785172	Cpn_1262822608	262822608	T	C	C	C	C	C	C	C	C	C	C	C	C	C	C	C	C	C	C	C	C	C
ENSRNOSNP2785173	Cpn_1262901030	262901030	T	C	C	C	T	T	C	C	T	T	T	T	T	T	T	T	T	T	T	T	-	-
ENSRNOSNP2785174	rat104_068_e04.q1ca_446	262932476	C	C	C	C	G	G	C	G	C	C	C	C	G	C	C	C	C	C	C	C	C	C
ENSRNOSNP2785175	J694885	263048790	G	A	A	A	G	G	A	A	A	A	A	A	G	A	A	A	A	A	-	A	A	A
ENSRNOSNP2785176	rat101_035_o18.p1ca_177	263117937	C	C	C	C	T	T	C	T	C	C	C	C	T	C	C	C	C	C	C	C	C	C
ENSRNOSNP2785177	SHRSPc22f12_s2_256	263242097	G	G	G	G	G	G	G	A	A	A	A	A	G	A	A	A	A	A	A	G	-	-
ENSRNOSNP2785178	J650021	263319210	T	T	T	T	T	T	T	C	C	C	C	C	T	C	C	C	C	C	-	T	T	T
ENSRNOSNP2785179	gnl|ti|896460202_19866866926148_373	263391751	T	C	C	C	T	T	C	C	T	T	T	T	T	T	T	T	T	T	T	T	T	T
ENSRNOSNP2785180	rat102_028_j01.p1ca_215	263448768	A	G	G	G	G	G	G	G	A	A	A	A	G	A	A	A	A	A	A	G	G	G
ENSRNOSNP2785181	J879251	263547789	A	G	G	G	G	G	G	G	G	G	G	G	G	G	G	G	G	G	G	A	A	A
ENSRNOSNP2785182	SHRSPc44h05_r1_198	263619558	G	G	G	G	A	A	G	A	A	A	A	A	A	A	A	A	A	A	A	G	G	G
ENSRNOSNP2785183	J569048	263650015	A	C	-	-	-	-	-	C	C	C	-	-	C	-	C	C	-	-	-	C	-	-
ENSRNOSNP2785184	Cpn_1263669531	263669531	G	A	A	A	G	G	A	A	A	A	A	A	G	A	A	A	A	A	A	G	G	G
ENSRNOSNP2785185	WKY-G-i-35e07_f1_419	263861863	T	C	C	C	C	C	C	C	C	C	C	C	C	C	C	C	C	C	C	C	C	C
ENSRNOSNP2785186	J339947	263983907	C	C	C	C	C	C	C	T	T	T	T	T	C	T	T	T	T	T	-	T	T	T
ENSRNOSNP2785187	gko-67b20_fp2_b1_310	264023249	C	C	C	C	C	C	C	T	C	C	C	C	C	C	C	C	C	C	C	T	T	T
ENSRNOSNP2785188	gko-45k4_fp2_b1_327	264163573	G	G	G	G	G	G	G	A	G	G	G	G	G	G	G	G	G	G	G	-	A	A
ENSRNOSNP2785189	J506032	264165063	G	G	G	-	G	-	-	A	G	G	G	G	G	-	G	G	G	G	-	A	A	A

ENSRNOSNP2785190	J639804	264276274	G	G	G	G	G	G	G	A	A	A	A	A	G	A	A	A	A	A	-	G	G	G
ENSRNOSNP2785191	gko-76k16_rp2_b1_86	264305485	G	G	G	G	G	G	G	A	G	G	G	G	G	G	G	G	G	G	G	A	A	A
ENSRNOSNP2785192	rat104_069_p02.q1ca_479	264614701	A	C	C	C	C	C	C	C	C	C	C	C	C	C	C	C	C	C	C	C	C	C
ENSRNOSNP2785193	Cpn_1264614861	264614861	C	T	T	T	T	T	T	T	T	T	T	T	T	T	T	T	T	T	T	T	T	T
ENSRNOSNP2785194	J472637	264637488	A	A	A	A	A	A	A	G	A	A	A	A	A	A	A	A	A	A	-	G	G	G
ENSRNOSNP2785195	J507202	264748071	T	T	T	T	T	T	T	A	T	T	T	T	T	T	T	T	T	T	-	A	A	A
ENSRNOSNP2785196	WKYc81d05_s1_72	264838258	A	G	G	G	G	G	G	G	G	G	G	G	G	G	G	G	G	G	G	A	A	A
ENSRNOSNP2785197	J506658	264898559	C	C	C	C	C	C	C	T	C	C	C	C	C	C	C	C	C	C	C	T	T	T
ENSRNOSNP2785198	SHRSPa17c07_r1_925	264975989	G	C	C	C	C	C	C	C	C	C	C	C	C	C	C	C	C	C	C	G	G	G
ENSRNOSNP2785199	J580196	265120413	G	G	G	G	G	G	G	T	G	G	G	G	G	G	G	G	G	G	-	T	T	T
ENSRNOSNP2785200	gko-67j4_rp2_b1_656	265120622	T	C	C	C	T	T	C	C	C	C	C	C	C	C	C	C	C	C	C	C	C	C
ENSRNOSNP2785201	rat101_031_n21.p1ca_169	265305730	G	G	G	G	A	A	G	A	G	G	G	G	G	G	G	G	G	G	G	G	G	G
ENSRNOSNP2785202	rat101_005_a10.p1ca_321	265306719	A	A	A	A	G	G	A	G	A	A	A	A	A	A	A	A	A	A	A	G	G	G
ENSRNOSNP2785203	gko-57g10_rp2_b1_307	265606203	T	G	G	G	T	T	G	G	G	G	G	G	G	G	G	G	G	G	G	G	G	G
ENSRNOSNP2785204	rdahl-13d10_fp2_b1_119	265780564	T	A	A	A	A	A	A	A	A	A	A	A	A	A	A	A	A	A	A	A	A	A
ENSRNOSNP2785205	SHRSPc67g11_r1_497	266004074	G	G	G	G	G	G	G	C	C	C	-	C	C	G/C	C	C	C	C	C	G	G	G
ENSRNOSNP2785206	J876269	266026349	C	T	T	T	C	C	T	T	T	T	T	T	T	T	T	T	T	T	-	T	T	T
ENSRNOSNP2785207	J557484	266121730	C	A	A	A	C	C	A	A	C	C	C	C	C	C	C	C	C	C	C	A	A	A
ENSRNOSNP2785208	gnl|ti|896599295_19866867070955_252	266197938	T	C	C	C	C	C	C	C	C	C	C	C	C	C	C	C	C	C	C	C	C	C
ENSRNOSNP2785209	J483342	266217621	G	C	C	C	-	-	C	C	C	C	C	C	C	C	C	C	C	C	-	C	C	C
ENSRNOSNP2785210	rat110_008_h22.p1ca_98	266227149	G	A	A	A	A	A	A	A	A	A	A	A	A	A	A	A	A	A	A	A	A	A
ENSRNOSNP2785211	SHRSPc12b09_r1_586	266460770	C	C	C	C	C	C	C	T	T	T	T	T	T	T	T	T	T	T	T	C	C	C
ENSRNOSNP2785212	J873120	266493431	C	C	C	C	C	C	C	A	A	A	A	A	A	A	A	A	A	A	A	C	C	C
ENSRNOSNP2785213	WKYc30a10_r1_599	266567568	T	T	T	T	C	C	T	C	T	T	T	T	T	T	T	T	T	T	T	T	T	T
ENSRNOSNP2785214	gnl|ti|897030222_19866868437155_261	266742664	C	C	C	C	C	C	C	T	T	T	T	T	T	T	T	T	T	T	T	C	C	C
ENSRNOSNP2785215	J490207	266979802	G	A	A	A	A	A	A	A	G	G	G	G	G	G	G	G	G	G	G	A	A	A
ENSRNOSNP2785216	rat102_039_o17.q1ca_138	267130082	G	C	C	C	C	C	C	C	C	C	C	C	C	C	C	C	C	C	C	C	C	C
ENSRNOSNP2785217	rat103_033_l14.q1ca_306	267130263	A	G	G	G	G	G	G	G	G	G	G	G	G	G	G	G	G	G	G	G	G	G
ENSRNOSNP2785218	Cpn_1267130354	267130354	G	G	G	G	G	G	G	C	C	C	C	C	C	C	C	C	C	C	C	G	G	G
ENSRNOSNP2785219	J672723	267194715	G	C	C	C	C	C	C	C	C	C	C	C	C	C	C	C	C	C	-	C	C	C
ENSRNOSNP2785220	WKYc57g01_s1_188	267269141	T	C	C	C	C	C	C	C	C	C	C	C	C	C	C	C	C	C	C	C	C	C
ENSRNOSNP2785221	J680709	267369088	C	G	G	G	G	G	G	G	G	G	G	G	G	G	G	G	G	G	-	G	G	G
ENSRNOSNP2785222	rdahl-90j15_rp2_b1_405	267426035	A	G	G	G	G	G	G	G	G	G	G	G	G	G	G	G	G	G	G	G	G	G
ENSRNOSNP2785223	SHRSPa01h01_r1_37	267825206	C	T	T	T	T	T	T	T	T	T	T	T	T	T	T	T	T	T	T	T	T	T
ENSRNOSNP2785224	J526751	267832846	G	A	A	A	A	A	A	A	A	A	A	A	A	A	A	A	A	A	-	A	A	A
