# Supplementary material for: Pathophysiological, Genetic and Gene Expression Features of a Novel Rodent Model of the Cardio-Metabolic Syndrome
Source: PLoS One. 2008 Aug 13;3(8):e2962. doi: 10.1371/journal.pone.0002962 (PMC2500170; doi:10.1371/journal.pone.0002962)
Supplement: Table S2 — (0.11 MB PDF) [file pone.0002962.s002.pdf]

Table S2. Statistically significant transcription changes (P<0.05) between BN.GK-Nidd/gk1 congenics and BN controls in kidney, liver, skeletal muscle (Soleus) and adipose tissue (Retroperitoneal fat pad -RFP). When several probesets were designed for the same gene, results are given for individual probesets. Transcription ratio (TR) between groups and statistical significance are reported.

					Kidney		Liver		Soleus		RFP			
Chr	Genomic Location (bp)	Affymetrix	Symbol	Gene description	TR	P-Value	TR	P-Value	TR	P-Value	TR	P-Value	GenBank	UniGene
1	3319791	1375658_at	---	Transcribed locus	-1.11	3.71E-01	-1.48	4.26E-02	-1.24	4.87E-01	-1.37	2.04E-01	AI412317	Rn.22768
1	3598474	1380865_at	---	Transcribed locus	1.32	7.25E-04	-1.39	1.18E-01	1.01	9.89E-01	-1.02	9.40E-01	BM385284	---
1	3609014	1372650_at	---	Transcribed locus strongly similar to KIAA1010 protein	-1.35	1.67E-04	-1.65	3.17E-03	1.03	9.88E-01	-1.36	1.51E-01	BI296334	Rn.11811
1	17388089	1368155_at	Cyp2c40	cytochrome P450, family 2, subfamily c, polypeptide 40	1.01	9.73E-01	3.14	8.86E-03	-1.03	9.79E-01	1.00	9.96E-01	NM_031572	Rn.2586
1	20839512	1367798_at	Ahcy	S-adenosylhomocysteine hydrolase	-1.22	4.88E-02	-1.05	7.23E-01	-1.03	9.76E-01	1.04	8.13E-01	NM_017201	Rn.5878
1	21087436	1367905_at	Enpp3	ectonucleotide pyrophosphatase/phosphodiesterase 3	1.06	7.75E-01	-1.33	3.52E-02	-1.17	9.66E-01	1.03	9.69E-01	NM_019370	Rn.44
1	26954423	1372626_at	Tpd52l1	Tumor protein D52-like 1	-1.05	8.11E-01	2.71	8.55E-03	-1.02	9.92E-01	1.25	6.24E-01	AI231999	Rn.24825
1	29159371	1390632_at	---	LOC361464	1.05	8.01E-01	-1.42	2.77E-02	1.03	9.94E-01	1.18	3.16E-01	BE107414	Rn.51175
1	41919698	1379249_at	Wtap	Wilms' tumour 1-associating protein	1.03	9.14E-01	-1.40	2.60E-02	1.04	9.79E-01	1.15	4.90E-01	AA900400	Rn.6971
1	78854683	1394796_at	LOC688391	MAP/microtubule affinity-regulating kinase 4	1.01	9.34E-01	1.26	3.42E-02	1.12	8.96E-01	1.00	9.99E-01	AA964964	Rn.33009
1	79074787	1368587_at	Apoc1	apolipoprotein C-I	-1.35	1.66E-03	1.01	9.60E-01	-1.07	9.54E-01	-1.25	3.70E-01	NM_012824	Rn.8887
1	84461905	1375706_at	---	Transcribed locus	1.17	1.56E-02	1.30	1.84E-01	-1.10	9.06E-01	1.26	3.16E-01	AI230273	---
1	87995365	1383012_at	---	Transcribed locus moderately similar to LOC360381	-1.08	7.87E-01	-1.69	4.35E-02	-1.06	9.59E-01	-1.03	9.30E-01	BE115155	Rn.15145
1	93820963	1385465_at	---	Transcribed locus	-2.19	2.50E-05	-6.59	3.00E-09	-4.89	1.60E-04	-15.03	8.09E-07	BG379188	---
1	94490876	1371080_at	LOC292861	kallikrein	-1.94	9.69E-05	-1.02	9.53E-01	1.01	9.91E-01	-1.04	7.97E-01	AI169562	Rn.21391
1	96251683	1387874_at	Dbp	D site albumin promoter binding protein	1.97	9.38E-03	2.41	1.28E-01	1.62	4.63E-01	1.58	3.92E-01	AI230048	Rn.11274
1	96276007	1376248_at	Sult2b1	sulfotransferase family, cytosolic, 2B, member 1	1.01	9.67E-01	1.35	4.77E-02	1.01	9.94E-01	1.51	4.68E-01	BM390571	Rn.12882
1	109357282	1378391_at	---	Transcribed locus	1.30	2.26E-02	-1.05	7.43E-01	1.05	9.53E-01	-1.02	9.35E-01	AI511069	Rn.41288
1	110892401	1383510_at	---	Transcribed locus	1.56	1.67E-04	1.38	3.54E-02	1.51	3.19E-02	1.71	1.10E-02	AI230360	Rn.249
1	118489584	1375177_at	---	Similar to Krueppel-like factor 13	1.18	7.66E-02	1.37	2.91E-02	1.16	6.81E-01	1.39	2.41E-01	AI454932	Rn.7654
1	118855516	1372117_at	Mtmr10	myotubularin related protein 10	-1.19	7.23E-03	-1.04	8.87E-01	1.02	9.88E-01	-1.21	2.19E-01	BE329026	Rn.3510
1	120403185	1387812_at	Pace4	Subtilisin - like endoprotease	-1.40	1.82E-02	-1.14	4.70E-01	-1.16	8.96E-01	1.24	1.37E-01	NM_012999	Rn.950
1	121177229	1377357_at	---	Transcribed locus	-1.25	1.28E-03	-1.09	7.87E-01	-1.24	1.52E-01	-1.34	1.90E-01	BE106428	Rn.23597
1	122546780	1376738_at	Ttc23	tetratricopeptide repeat domain 23	-1.04	8.31E-01	-1.75	2.70E-03	-1.99	1.09E-03	1.01	9.62E-01	BF284579	Rn.16924
1	122910984	1380971_at	Igf1r	Insulin-like growth factor 1 receptor	-1.65	2.60E-04	-1.03	9.22E-01	-1.52	3.10E-02	-1.23	4.64E-01	BE120747	Rn.28746
1	128797261	1379830_at	---	Transcribed locus	2.16	5.00E-06	2.28	8.89E-05	2.19	4.84E-03	2.81	2.85E-03	BF396633	Rn.63869
1	129657552	1379236_at	---	Transcribed locus	1.25	1.84E-02	1.04	9.23E-01	-1.10	9.55E-01	1.32	2.87E-01	BF405781	Rn.60839
1	131460918	1398727_at	---	Transcribed locus weakly similar to phospholipid-transporting ATPase IF	1.31	4.67E-02	-1.27	3.57E-01	1.06	9.75E-01	-1.31	3.45E-01	BF398091	Rn.32838
1	131643841	1377209_at	---	Transcribed locus	-1.17	1.57E-01	-1.98	8.89E-05	-1.01	9.89E-01	1.09	5.86E-01	BI283664	Rn.16545
1	134627350	1376950_at	Det1	de-etiolated homolog 1	-1.08	5.07E-01	-1.45	4.51E-02	-1.16	8.96E-01	-1.11	6.58E-01	BE098978	Rn.9608
1	135592140	1379361_at	Pex11a	peroxisomal biogenesis factor 11A	-1.33	3.86E-03	-1.15	6.68E-01	-1.46	3.59E-01	1.09	7.91E-01	BI273703	Rn.25177
1	135593294	1387740_at	Pex11a	peroxisomal biogenesis factor 11A	-1.36	3.35E-03	-1.10	7.70E-01	-1.24	6.01E-01	1.03	9.56E-01	NM_053487	Rn.14519
1	135945648	1389650_at	Znf366	Zinc finger protein 366	-3.30	1.22E-07	-1.21	3.39E-01	-2.43	2.01E-03	-3.51	2.59E-06	BI295153	---
1	135946914	1381453_at	---	Transcribed locus	-1.32	1.56E-02	1.07	6.86E-01	1.35	9.05E-01	-1.30	2.08E-01	BE110814	Rn.27028
1	137905242	1368872_a_at	Homer2	homer homolog 2 (Drosophila)	-1.36	3.89E-03	1.10	7.23E-01	-1.41	3.65E-01	1.06	8.85E-01	NM_053309	Rn.30014
1	137953277	1397452_at	---	Transcribed locus	-1.30	6.99E-03	-1.18	3.22E-01	-1.91	2.51E-02	-1.44	1.01E-01	AI112776	---

1	138058667	1372066_at	Fam103a1	family with sequence similarity 103A1	1.20	4.77E-03	-1.03	8.73E-01	-1.14	5.43E-01	1.07	7.67E-01	AI170703	Rn.4307
1	138064475	1376780_at	---	Transcribed locus	8.41	1.09E-09	1.96	1.52E-03	3.53	6.13E-06	2.48	9.81E-05	AI172311	Rn.40252
1	138077689	1392745_at	RGD1305713	similar to RIKEN cDNA 3110040N11	1.73	6.90E-05	1.48	3.89E-03	1.85	1.09E-03	2.22	1.54E-04	BG380502	Rn.64273
1	138174461	1374151_at	Hdgfrp3	Hepatoma-derived growth factor, related protein 3	-1.14	3.19E-01	-1.24	5.44E-02	-1.45	1.05E-02	-1.41	5.14E-02	BI289940	Rn.8544
1	138175446	1394596_at	Hdgfrp3	Hepatoma-derived growth factor, related protein 3	-1.14	3.25E-01	-1.19	3.63E-01	-1.38	2.18E-02	-1.41	2.57E-01	AW531463	Rn.47713
1	138476741	1368931_at	Sh3gl3	SH3 domain protein 2 C1	-1.01	9.80E-01	1.47	3.27E-02	-1.01	9.91E-01	-1.10	7.09E-01	NM_031238	Rn.5909
1	140251089	1371820_at	Mesdc2	mesoderm development candiate 2	1.15	3.42E-02	1.14	4.57E-01	-1.07	9.60E-01	1.08	5.42E-01	BI282064	Rn.13022
1	140614228	1373315_at	---	Transcribed locus	-1.75	2.11E-05	1.06	8.35E-01	-1.08	9.70E-01	1.05	8.50E-01	AI176425	Rn.800
1	143494221	1368543_at	Nox4	NADPH oxidase 4	1.65	6.90E-05	2.38	7.55E-02	-1.59	6.90E-01	-1.41	3.46E-01	NM_053524	Rn.14744
1	143682602	1393508_at	---	Transcribed locus	1.92	3.11E-06	3.25	2.04E-03	-1.37	9.06E-01	-1.14	7.99E-01	AI043805	Rn.18728
1	146832490	1369452_a_at	Picalm	phosphatidylinositol binding clathrin assembly protein	1.15	4.46E-02	-1.03	9.25E-01	-1.04	9.74E-01	-1.00	9.96E-01	NM_053554	Rn.10888
1	147162130	1390107_at	---	Transcribed locus	1.57	2.21E-06	1.62	1.40E-03	1.11	9.70E-01	-1.01	9.76E-01	BG670294	---
1	149835626	1393657_at	Prcp	prolylcarboxypeptidase (angiotensinase C)	1.26	1.04E-01	1.53	5.83E-03	1.27	5.68E-01	5.28	8.09E-07	BI285860	Rn.78613
1	149841592	1379248_at	---	Transcribed locus	1.25	8.65E-02	1.44	3.46E-03	1.21	5.36E-01	2.41	2.96E-03	AA926318	Rn.8457
1	154408321	1392533_at	Nars2	asparaginyl-tRNA synthetase 2, mitochondrial	1.02	9.25E-01	-1.04	9.14E-01	1.36	2.82E-02	1.15	4.93E-01	AI716888	Rn.16411
1	154864943	1383104_at	LOC361606	similar to RIKEN cDNA 1810020D17	1.09	2.62E-01	1.26	2.26E-02	1.14	7.86E-01	1.27	1.28E-01	BI295116	Rn.19052
1	155052201	1384877_at	Aqp11	aquaporin 11	1.49	2.78E-05	1.19	1.76E-01	-1.02	9.81E-01	-1.33	6.30E-01	AI072405	Rn.20144
1	155443097	1385851_at	Capn5	Calpain 5	1.12	2.24E-01	1.42	4.43E-02	-1.03	9.88E-01	1.07	8.66E-01	AA925441	Rn.15526
1	155530962	1390187_at	---	Transcribed locus	1.22	6.44E-02	1.76	1.45E-02	1.08	9.68E-01	1.48	8.34E-02	AI060043	Rn.19122
1	156251610	1374729_at	---	Transcribed locus strongly similar to UV radiation resistance associated gene	-1.04	6.86E-01	-1.14	3.19E-01	-1.49	3.76E-03	-1.06	7.15E-01	BM385616	Rn.15803
1	156525993	1371615_at	Dgat2	diacylglycerol O-acyltransferase homolog 2	1.55	7.21E-05	1.15	4.79E-01	1.07	9.89E-01	1.55	2.13E-01	BI279069	Rn.9523
1	156526795	1391045_at	Dgat2	diacylglycerol O-acyltransferase homolog 2	1.47	9.65E-06	1.16	4.06E-01	1.05	9.91E-01	1.75	1.28E-01	BI277463	Rn.23436
1	156594938	1383979_at	---	Transcribed locus	1.49	2.27E-03	-1.05	7.77E-01	1.02	9.88E-01	1.16	7.20E-01	BM392389	Rn.19175
1	156745316	1371310_s_at	Serpinh1	serine (or cysteine) proteinase inhibitor, clade H, member 1	1.09	5.92E-01	-3.39	1.34E-06	-1.14	9.87E-01	1.13	6.49E-01	BI285495	Rn.32111
1	157021524	1397957_at	---	Transcribed locus	-1.28	5.54E-03	1.03	8.98E-01	-1.07	9.55E-01	-1.25	1.18E-01	AA955460	Rn.73491
1	157024354	1391138_at	---	Transcribed locus	1.39	2.12E-03	-1.03	9.25E-01	1.11	7.25E-01	1.46	2.28E-02	BF410536	---
1	157531462	1384265_at	Pold3	polymerase (DNA-directed), delta 3, accessory subunit	1.00	9.80E-01	-1.38	1.47E-02	-1.05	9.74E-01	-1.02	9.37E-01	BI278347	Rn.45023
1	158043874	1374416_at	---	Transcribed locus	1.34	5.63E-04	-1.08	6.13E-01	1.25	4.01E-01	1.10	7.06E-01	AI169330	---
1	158085215	1379306_at	---	Transcribed locus	-1.80	2.84E-06	-1.76	4.13E-04	-1.99	5.46E-05	-1.56	2.28E-02	BE111794	Rn.19759
1	158160911	1370334_at	Plekhb1	evectin-1	-1.39	3.86E-03	-1.70	2.77E-02	-1.16	9.53E-01	-1.45	7.19E-02	AF081582	Rn.8415
1	158396007	1374907_at	---	Transcribed locus	1.33	2.11E-02	1.11	5.64E-01	1.31	9.18E-01	1.26	3.20E-01	BI274338	Rn.41004
1	159550706	1369031_at	Il18bp	interferon gamma inducing factor binding protein	1.31	3.98E-03	1.40	3.83E-02	1.62	1.71E-01	1.29	2.04E-01	NM_053374	Rn.20083
1	167456059	1371085_at	Ascl3	achaete-scute complex homolog-like 3	-2.37	1.81E-07	-2.20	4.01E-04	-2.16	5.46E-05	-3.56	8.09E-07	AB046449	Rn.19594
1	167465181	1374675_at	---	Similar to D7H11orf15 protein	1.27	2.51E-04	1.24	2.15E-01	1.40	3.21E-02	1.23	9.32E-02	BG374992	Rn.8151
1	168680142	1383139_at	rnf141	Ring finger protein 141	-1.07	3.88E-01	-1.44	2.35E-03	-1.01	9.91E-01	-1.03	9.12E-01	BM386881	Rn.11887
1	168682049	1393939_at	rnf141	Ring finger protein 141	-1.04	8.36E-01	-1.33	2.57E-02	1.03	9.79E-01	1.07	6.60E-01	AI137989	Rn.27556
1	168683040	1385787_at	rnf141	ring finger protein 141	1.03	8.89E-01	-1.47	1.79E-03	1.04	9.79E-01	1.05	8.89E-01	AI059580	Rn.19219
1	169040461	1396347_at	---	Transcribed locus	-1.05	8.69E-01	-1.04	8.51E-01	-1.88	2.12E-02	1.06	9.45E-01	BF395640	Rn.47103
1	170822207	1373808_at	---	CDNA clone IMAGE:7193711	1.21	1.83E-02	-1.23	4.61E-01	1.28	5.43E-01	-1.02	9.47E-01	BF417211	Rn.13986
1	172157027	1373487_at	---	Transcribed locus	-1.39	3.43E-05	-1.00	9.91E-01	-1.16	9.44E-01	-1.29	6.02E-01	AI030552	Rn.6377
1	174571185	1379433_at	Pik3c2a	Phosphatidylinositol 3-kinase, C2 domain containing, alpha polypeptide	1.48	1.58E-04	1.58	3.14E-03	1.36	6.57E-01	1.48	1.65E-01	AI059449	Rn.19464

1	174750004	1370000_at	Nucb2	nucleobindin 2	1.36	8.76E-04	1.86	4.25E-03	-1.01	1.00E+00	1.21	2.60E-01	NM_021663	Rn.41602
1	175946698	1374543_at	---	Transcribed locus	-1.23	2.90E-02	1.01	9.82E-01	1.08	9.67E-01	-1.51	7.34E-03	AA858791	Rn.16899
1	177210126	1382159_at	---	CDNA clone MGC:94842 IMAGE:7109216	-1.58	2.84E-06	-1.55	2.52E-03	-1.78	2.51E-03	-1.77	1.35E-03	BF386740	Rn.24108
1	177345660	1373336_at	---	G protein-coupled receptor, family C, group 5B	-1.08	5.48E-01	-1.01	9.83E-01	-1.08	9.81E-01	1.58	1.97E-02	AI411074	Rn.9999
1	177347289	1371671_at	---	G protein-coupled receptor, family C, group 5B	1.01	9.85E-01	-1.02	9.39E-01	-1.00	1.00E+00	1.61	2.14E-03	AI228513	Rn.15338
1	177849164	1386933_at	Gp2	glycoprotein 2 (zymogen granule membrane)	-1.88	7.98E-05	-1.04	8.73E-01	-1.05	9.64E-01	1.10	5.26E-01	NM_134418	Rn.11223
1	178220412	1383303_at	---	Similar to RIKEN cDNA 4933424N09	1.01	9.69E-01	-1.53	4.91E-03	1.02	9.89E-01	-1.09	5.48E-01	BI282211	Rn.28004
1	178222864	1394458_at	---	Similar to RIKEN cDNA 4933424N09	-1.03	8.99E-01	-1.57	9.82E-03	1.11	8.61E-01	-1.09	6.91E-01	BF291026	---
1	179630314	1381176_at	---	Transcribed locus	1.34	2.95E-03	1.05	7.38E-01	1.53	6.67E-01	1.34	2.40E-01	BF409078	---
1	181000243	1398544_at	---	Transcribed locus	1.18	2.56E-02	1.05	7.74E-01	1.41	1.92E-01	1.34	3.62E-01	BF398673	Rn.79288
1	185515397	1388462_at	---	Transcribed locus	-1.06	6.03E-01	1.87	1.02E-04	1.19	5.71E-01	1.30	2.88E-01	AI407506	---
1	186111163	1390333_at	Ppp4c	Protein phosphatase 4 (formerly X), catalytic subunit	1.25	9.43E-04	1.43	1.63E-03	1.18	5.43E-01	1.18	3.04E-01	AA957050	Rn.9173
1	186343049	1397628_at	RGD1305592	similar to RIKEN cDNA 2900092E17	-1.17	8.78E-02	-1.27	4.64E-02	-1.59	2.95E-02	1.01	9.86E-01	BM390965	Rn.38914
1	186561872	1393347_at	Itgal	Integrin alpha L	3.41	8.27E-06	2.64	3.50E-04	1.37	1.61E-01	3.07	1.42E-03	BI289767	Rn.16919
1	187040391	1393731_at	Rnf40	ring finger protein 40	-1.68	2.38E-04	-1.65	4.18E-03	-1.39	3.85E-02	-2.00	1.29E-03	BF403369	Rn.71174
1	187360436	1370379_at	Prss8	protease, serine, 8 (prostasin)	1.18	4.43E-02	-1.02	9.31E-01	1.01	9.95E-01	-1.04	8.78E-01	AF202076	Rn.24211
1	187411479	1393068_at	---	Transcribed locus	1.20	1.53E-01	1.02	9.70E-01	1.39	2.68E-01	1.91	4.54E-02	AA956927	Rn.9132
1	187614459	1367782_at	Cox6a2	cytochrome c oxidase, subunit VIa, polypeptide 2	1.19	5.00E-02	1.06	6.84E-01	1.01	9.89E-01	6.82	3.16E-01	NM_012812	Rn.5119
1	187656500	1370887_at	Tgfb1i1	transforming growth factor beta 1 induced transcript 1	1.20	3.36E-02	1.03	9.22E-01	1.05	9.85E-01	1.08	7.20E-01	BI279862	Rn.23203
1	187710576	1389160_at	Eraf	erythroid associated factor	-1.06	9.54E-01	1.91	4.11E-02	1.05	9.77E-01	1.42	7.76E-01	AI230287	Rn.12724
1	187953748	1390026_at	Bag3	Bcl2-associated athanogene 3	1.03	8.65E-01	-1.57	2.81E-02	-1.00	9.97E-01	-1.19	5.02E-01	AI231792	Rn.2699
1	188095194	1391312_at	Inpp5f	inositol polyphosphate-5-phosphatase F	1.31	4.34E-02	1.17	1.80E-01	1.15	7.15E-01	1.40	1.37E-01	BE108090	Rn.51411
1	188192344	1380163_at	Sec23ip	SEC23 interacting protein	-1.41	1.18E-03	-1.36	1.65E-02	-1.21	4.40E-01	-1.37	9.98E-02	BI302685	Rn.51166
1	193333964	1380137_at	---	Transcribed locus	-1.07	6.06E-01	-1.03	9.48E-01	-1.53	2.26E-02	1.08	7.92E-01	BF406176	Rn.60916
1	193367639	1374323_at	---	Transcribed locus	-1.01	9.58E-01	-1.26	2.12E-02	-1.16	6.08E-01	-1.08	6.25E-01	BM389139	Rn.7853
1	195421407	1371115_at	Ptpre	protein tyrosine phosphatase, receptor type, E	-1.41	7.99E-03	-1.08	6.87E-01	-1.39	6.01E-01	-1.91	2.85E-03	D78610	Rn.10766
1	195455253	1379313_at	---	Transcribed locus	1.96	1.06E-04	1.28	3.77E-01	1.95	6.63E-03	2.45	8.31E-04	BE108519	---
1	196779357	1368311_at	Mgmt	O-6-methylguanine-DNA methyltransferase	2.27	3.34E-07	1.91	9.10E-06	1.80	1.60E-04	1.81	3.47E-04	NM_012861	Rn.9836
1	197265481	1382727_at	---	Transcribed locus	1.48	5.08E-04	1.22	8.26E-02	1.22	9.54E-01	1.21	4.62E-01	AI071140	Rn.20024
1	197326005	1367870_at	Txnl2	thioredoxin-like 2	-1.08	3.80E-01	-1.26	2.99E-02	-1.13	8.31E-01	-1.03	8.79E-01	NM_032614	Rn.3578
1	198123105	1391919_at	---	Similar to hypothetical protein LOC256536	1.19	4.30E-02	1.04	8.63E-01	-1.04	9.74E-01	-1.11	6.14E-01	AW523472	Rn.45649
1	198865303	1392252_at	---	Transcribed locus	-1.02	9.42E-01	1.10	7.54E-01	-1.09	9.54E-01	-1.94	2.82E-02	AA819893	Rn.37078
1	199033695	1377534_at	Stk32c	Serine/threonine kinase 32C	-3.94	3.13E-05	1.02	9.43E-01	1.08	9.55E-01	-1.06	7.91E-01	BE097305	Rn.49654
1	201068525	1367871_at	Cyp2e1	cytochrome P450, family 2, subfamily e, polypeptide 1	-1.06	8.03E-01	-1.06	6.86E-01	-2.43	2.20E-01	-17.15	3.23E-03	NM_031543	Rn.1372
1	201153605	1393436_at	Scgb1c1	secretoglobin, family 1C, member 1	-1.39	1.12E-04	1.02	9.33E-01	-1.00	9.98E-01	1.06	7.16E-01	AI549033	Rn.41707
1	201171387	1372925_at	Sirt3	sirtuin 3	-1.35	8.05E-02	-2.04	1.34E-03	-1.01	1.00E+00	1.19	3.64E-01	AI233135	Rn.24698
1	201242709	1394195_at	---	Transcribed locus	1.19	3.14E-02	1.08	7.10E-01	-1.00	9.98E-01	1.04	8.59E-01	AI638983	Rn.43897
1	201276888	1374831_at	Athl1	acid trehalase-like 1	1.59	1.48E-06	-1.07	7.53E-01	1.10	9.74E-01	-1.17	3.41E-01	AI172579	Rn.23728
1	201284348	1367696_at	Ifitm2	interferon induced transmembrane protein 2 (1-8D)	1.14	4.46E-02	1.18	3.99E-01	-1.16	9.55E-01	-1.08	6.90E-01	NM_030833	Rn.2648
1	201438410	1378605_at	Sigirr	single immunoglobulin and toll-interleukin 1 receptor domain	-1.09	4.60E-01	2.00	6.90E-04	-1.10	9.44E-01	-1.13	6.62E-01	AA818702	Rn.16525
1	201447921	1393633_at	---	Transcribed locus weakly similar to tumor protein p53 inducible protein 5	1.29	4.26E-02	-1.02	9.39E-01	-1.01	9.92E-01	1.01	9.80E-01	AI071826	Rn.21998

1	201505805	1394936_at	Ptdss2	Phosphatidylserine synthase 2	1.22	6.71E-03	1.21	2.98E-01	1.16	8.22E-01	1.39	8.40E-02	AW521480	Rn.69818
1	201506258	1385901_at	---	Transcribed locus	1.63	1.06E-04	1.50	2.59E-03	1.59	3.10E-02	2.10	2.13E-03	BE118197	---
1	201707420	1392324_at	Eps8l2	EPS8-like 2	-1.39	4.51E-02	-1.22	3.51E-01	-1.12	9.67E-01	1.02	9.14E-01	AI137983	Rn.23793
1	201714598	1372654_at	Eps8l2	EPS8-like 2	-1.37	2.49E-04	-1.53	8.55E-03	-1.30	6.39E-01	-1.06	7.78E-01	BI285918	Rn.13824
1	201748707	1391202_at	---	Transcribed locus strongly similar to protein D030034H08	2.54	6.25E-06	2.57	5.81E-06	1.24	2.52E-01	1.85	3.85E-03	AW252232	---
1	201807314	1367681_at	Cd151	CD151 antigen	1.20	3.49E-03	1.15	2.81E-01	-1.09	9.74E-01	1.05	8.82E-01	NM_022523	Rn.1465
1	201836895	1383152_at	Chid1	chitinase domain containing 1	-1.38	3.70E-03	-1.51	1.71E-02	-1.34	5.43E-01	-1.20	4.14E-01	BF544427	Rn.27342
1	204340635	1379472_at	Nadsyn1	NAD synthetase 1	1.27	2.80E-02	1.34	1.16E-01	1.27	3.11E-01	1.35	1.61E-01	AI232666	Rn.22886
1	204402226	1368189_at	Dhcr7	7-dehydrocholesterol reductase	-1.16	4.69E-01	2.30	4.71E-03	-1.02	9.90E-01	1.70	3.06E-01	NM_022389	Rn.228
1	205163016	1391199_at	Fadd	Fas (TNFRSF6)-associated via death domain	-1.31	1.35E-02	-1.18	4.19E-01	-1.03	9.87E-01	-1.11	5.78E-01	BE100715	Rn.16183
1	205856421	1376313_at	Tpcn2	two pore segment channel 2	-1.29	1.09E-02	-2.68	6.89E-05	-1.36	1.69E-01	-1.98	8.31E-04	BI296463	Rn.27378
1	206725911	1389785_at	Acy3	aspartoacylase (aminoacylase) 3	-1.16	1.27E-01	-1.35	2.39E-02	1.24	5.82E-01	1.23	2.87E-01	AA892933	Rn.22552
1	206780855	1388122_at	Gstp1	glutathione-S-transferase, pi 1/pi 2	-1.18	2.93E-02	-1.21	3.46E-01	-1.02	9.83E-01	-1.28	2.17E-01	X02904	Rn.44821
1	207056887	1399154_at	Fbxl11	F-box and leucine-rich repeat protein 11	-1.11	4.17E-01	-1.27	2.86E-02	-1.07	9.55E-01	-1.16	3.97E-01	BF389273	Rn.12228
1	207153791	1382197_at	Rhod	ras homolog gene family, member D	-1.03	7.84E-01	-1.30	3.71E-02	1.07	9.48E-01	-1.14	4.15E-01	AA955648	Rn.25197
1	207547786	1389080_at	---	RNA binding motif protein 4	-1.16	3.61E-02	-1.35	3.83E-02	-1.11	8.15E-01	-1.30	1.74E-01	AI233457	Rn.8556
1	207557693	1384091_at	---	Transcribed locus	-1.47	7.21E-05	-1.18	2.01E-01	-1.15	7.25E-01	-1.57	8.40E-02	AA899909	Rn.14995
1	207558819	1395762_at	---	Transcribed locus weakly similar to LOC360381	-2.11	1.86E-06	-1.66	1.20E-02	-1.85	1.90E-03	-1.73	1.51E-01	BG670091	Rn.72606
1	207710462	1382476_x_at	---	Transcribed locus	1.23	4.43E-02	1.26	1.22E-01	-1.01	9.92E-01	-1.05	8.90E-01	AI060133	---
1	208630009	1371595_at	---	Transcribed locus weakly similar to LOC360381	-1.23	7.57E-02	-1.80	1.32E-03	1.01	9.94E-01	-1.18	2.53E-01	BM384301	Rn.8145
1	208630791	1397164_at	---	Transcribed locus weakly similar to LOC360381	-1.20	1.99E-01	-2.57	2.07E-04	1.02	9.92E-01	1.05	8.92E-01	AI175779	Rn.55633
1	208701589	1389145_at	Cdc42ep2	CDC42 effector protein (Rho GTPase binding) 2	-1.13	3.09E-01	-1.03	9.18E-01	1.82	2.82E-02	-1.15	6.51E-01	D86683	Rn.17464
1	209062910	1368166_at	Arl2	ADP-ribosylation factor-like 2	1.05	6.82E-01	1.46	3.84E-03	1.01	9.91E-01	1.18	3.63E-01	NM_031711	Rn.36965
1	209160917	1377235_a_at	Ppp2r5b	protein phosphatase 2, regulatory subunit B (B56), beta isoform	1.32	2.54E-03	-1.00	1.00E+00	1.04	9.74E-01	-1.12	4.68E-01	BI281643	Rn.40996
1	209294583	1372756_at	---	Mitogen activated protein kinase kinase kinase kinase 2	-1.20	5.83E-02	-1.09	6.65E-01	-1.11	8.89E-01	-1.73	1.98E-02	BG378166	---
1	209479235	1396039_at	---	Transcribed locus	1.29	3.29E-02	1.02	9.19E-01	-1.01	9.95E-01	1.07	5.45E-01	BG377396	---
1	209741843	1367677_at	Prdx5	peroxiredoxin 5	-1.02	8.52E-01	-1.44	2.10E-03	1.09	9.54E-01	1.04	8.45E-01	NM_053610	Rn.2944
1	209814122	1380854_at	---	Transcribed locus	1.84	4.80E-05	1.86	1.69E-02	1.58	4.56E-01	1.88	6.39E-03	BF396482	Rn.70676
1	209902080	1381774_at	Lrp16	LRP16 protein	-1.18	1.36E-01	-1.26	4.02E-02	-1.12	9.60E-01	-1.11	5.98E-01	BE095849	Rn.26587
1	209918841	1384304_at	Lrp16	LRP16 protein	1.19	4.55E-02	1.02	9.09E-01	1.00	1.00E+00	1.03	8.59E-01	AW524615	Rn.45890
1	210030010	1372958_at	---	Transcribed locus strongly similar to HSPC263	-1.92	1.47E-06	-1.43	2.69E-02	-1.39	3.97E-02	-1.49	1.42E-03	BE111986	Rn.35476
1	210030671	1399073_at	Otub1	OTU domain, ubiquitin aldehyde binding 1	-2.65	1.12E-07	-2.16	7.49E-06	-3.71	1.53E-04	-1.93	1.54E-04	BI274378	---
1	210267960	1373571_at	Rtn3	reticulon 3	-1.39	2.55E-04	-1.06	7.74E-01	-1.18	6.00E-01	-1.30	1.48E-01	AI170276	Rn.37848
1	210376903	1384797_at	---	Transcribed locus	-1.02	9.25E-01	-1.34	2.60E-02	-1.33	8.34E-01	1.10	6.58E-01	BF404325	Rn.59545
1	210378419	1395980_at	---	Similar to RIKEN cDNA 5730596K20	-1.05	7.42E-01	-1.32	2.60E-02	-1.15	9.15E-01	-1.02	9.00E-01	BF393243	Rn.48295
1	210382751	1383835_at	---	Transcribed locus	-1.93	4.85E-05	-2.55	7.68E-05	-1.43	3.83E-01	-4.44	3.04E-06	BI303704	Rn.32756
1	210777138	1369840_at	RGD:620976	integral membrane transport protein UST4r	-1.08	3.92E-01	1.53	1.62E-02	1.05	9.61E-01	1.13	5.10E-01	NM_134379	Rn.81213
1	210918867	1369450_at	RGD:620985	integral membrane transport protein UST5r	1.72	9.69E-05	1.13	2.96E-01	-1.01	9.94E-01	-1.04	7.92E-01	NM_134380	Rn.81214
1	211446536	1385005_at	---	Transcribed locus	2.09	2.21E-06	1.54	1.11E-01	1.02	9.87E-01	1.00	9.90E-01	AW530264	---
1	211676320	1371811_at	Bscl2	Bernardinelli-Seip congenital lipodystrophy 2 homolog	1.19	3.65E-02	1.05	8.02E-01	1.03	9.79E-01	-1.04	8.86E-01	BI278232	---
1	211774518	1373527_at	---	Transcribed locus	-1.38	3.04E-04	-1.26	9.65E-02	-1.33	5.43E-01	-1.69	2.13E-03	BI275677	Rn.11674

1	212346271	1377869_at	---	Similar to carbon catabolite repression 4 protein homolog	1.42	4.44E-02	7.16	4.73E-02	1.12	9.74E-01	-1.56	2.03E-01	BI284261	Rn.15040
1	212482220	1391477_at	---	Transcribed locus	-1.67	2.94E-04	-1.13	4.15E-01	-1.11	9.07E-01	-1.13	5.11E-01	BF396595	---
1	212670777	1368453_at	Fads2	fatty acid desaturase 2	-1.04	8.07E-01	1.69	3.93E-03	1.00	1.00E+00	1.08	7.61E-01	NM_031344	Rn.32872
1	212829200	1367857_at	Fads1	fatty acid desaturase 1	1.48	1.20E-02	2.31	6.44E-05	-1.07	9.83E-01	1.08	7.54E-01	NM_053445	Rn.28161
1	213449268	1384981_at	Cd6	CD6 antigen	-1.05	7.03E-01	1.07	7.15E-01	3.01	3.13E-06	-1.00	9.94E-01	BM384254	Rn.57361
1	213697961	1377626_at	---	Transcribed locus	-1.57	3.25E-02	-1.25	7.59E-01	-1.44	2.11E-01	-1.30	5.74E-01	AA924350	---
1	215546341	1369427_at	Mpeg1	macrophage expressed gene 1	1.10	7.27E-01	-3.61	2.67E-03	1.04	9.94E-01	1.15	7.86E-01	NM_022617	Rn.48702
1	215549786	1389006_at	Mpeg1	Macrophage expressed gene 1	1.21	2.41E-01	-3.23	4.27E-03	1.17	9.91E-01	1.24	7.41E-01	AI170394	Rn.4301
1	215824004	1391652_at	---	Transcribed locus	-1.29	1.06E-04	-1.37	2.39E-02	-1.11	9.38E-01	-1.03	9.25E-01	AI411140	Rn.21872
1	215826644	1380905_at	---	Transcribed locus weakly similar to RIKEN 5830411J07	-1.16	4.01E-01	-1.39	2.77E-02	1.03	9.74E-01	-1.02	9.24E-01	AA893260	Rn.40554
1	215919898	1370592_at	Keg1	kidney expressed gene 1	-1.93	4.09E-06	-1.34	1.56E-01	1.03	9.74E-01	1.06	6.65E-01	AB019693	Rn.22745
1	219430819	1381749_at	---	Transcribed locus	-1.31	5.70E-03	1.11	4.74E-01	-1.07	8.90E-01	1.10	7.78E-01	AI703743	Rn.40793
1	219432441	1372665_at	---	Transcribed locus	-1.06	5.26E-01	1.97	6.32E-04	-1.01	9.98E-01	1.15	5.08E-01	AI230228	Rn.30218
1	219483445	1369433_at	Cep78	centrosomal protein 78	-1.41	2.64E-04	1.04	8.89E-01	1.02	9.88E-01	-1.25	1.81E-01	NM_021741	Rn.42936
1	220070142	1393825_at	---	TGFB inducible early growth response 3	-1.68	1.18E-03	-1.05	7.91E-01	-1.03	9.74E-01	-1.03	8.89E-01	AI454854	Rn.35127
1	220146892	1381557_at	---	TGFB inducible early growth response 3	-3.07	5.30E-07	1.05	9.13E-01	-1.20	8.89E-01	-1.09	5.40E-01	BI289045	Rn.73873
1	222420824	1392994_at	---	Similar to Enolase 1	1.17	8.04E-02	1.91	8.53E-03	1.15	5.92E-01	1.53	5.14E-02	AW522671	Rn.19716
1	223642130	1367614_at	Anxa1	annexin A1	-1.27	1.75E-03	-1.04	9.42E-01	-1.16	9.76E-01	1.28	2.16E-01	NM_012904	Rn.1792
1	223657605	1394451_at	Anxa1	annexin A1	-1.38	1.95E-02	1.17	3.83E-01	-1.43	8.90E-01	1.04	9.50E-01	AI236455	Rn.63926
1	223895680	1368718_at	Aldh1a4	aldehyde dehydrogenase family 1, subfamily A4	1.21	8.78E-02	57.68	2.78E-09	1.32	3.83E-01	3.05	1.17E-01	M23995	Rn.74044
1	223895697	1387022_at	Aldh1a1	aldehyde dehydrogenase family 1, member A1	-1.20	2.70E-03	-1.96	6.77E-03	1.28	8.89E-01	1.02	9.94E-01	NM_022407	Rn.6132
1	224611744	1388868_at	Zfp216	zinc finger protein 216	-1.30	1.18E-03	-1.21	2.16E-01	-1.04	9.66E-01	1.04	9.40E-01	AI172425	Rn.4050
1	224614398	1383905_at	---	Transcribed locus	-1.53	5.25E-04	-1.36	2.59E-02	-1.37	6.31E-02	-1.14	7.78E-01	BF401729	Rn.17596
1	225262131	1394160_at	---	Transmembrane protein 2	-2.84	5.00E-06	-4.63	6.02E-08	-5.21	3.13E-06	-5.78	2.91E-05	AI101945	Rn.28119
1	229028588	1372069_at	Ankrd15	ankyrin repeat domain 15	-1.25	5.40E-03	-1.11	6.82E-01	-1.09	7.29E-01	-1.21	2.07E-01	BF284716	Rn.2191
1	232764894	1373083_at	---	Similar to hypothetical protein FLJ10058	1.03	8.39E-01	1.33	3.64E-02	-1.04	9.85E-01	1.07	8.34E-01	AW915681	Rn.7335
1	232816523	1374074_at	Cdc37l	cell division cycle 37 homolog (S. cerevisiae)-like	-1.14	1.72E-01	-1.49	3.21E-03	-1.23	9.08E-02	-1.09	6.28E-01	BF285292	Rn.19147
1	232822760	1368095_at	---	Transcribed locus	1.18	1.22E-02	-1.00	1.00E+00	1.12	6.01E-01	-1.03	9.53E-01	NM_013218	---
1	232842713	1375577_at	---	Transcribed locus	1.31	2.44E-02	1.26	5.36E-02	1.35	6.01E-01	1.27	2.16E-01	BF400031	---
1	233488233	1383992_at	---	Transcribed locus	-1.31	1.57E-02	-1.16	3.34E-01	1.00	1.00E+00	-1.19	3.54E-01	BM383990	---
1	233490983	1388889_at	Ermp1	endoplasmic reticulum metallopeptidase 1	-1.61	1.68E-06	-1.88	9.10E-06	-1.54	1.61E-03	-1.75	1.29E-04	AI598417	Rn.7753
1	233493258	1384975_at	Ermp1	endoplasmic reticulum metallopeptidase 1	1.31	1.04E-03	1.24	2.16E-01	1.10	8.92E-01	1.43	7.06E-02	BM387447	Rn.81999
1	233573321	1390596_at	Mlana	melan-A	-2.09	3.31E-05	-1.04	8.88E-01	1.03	9.80E-01	-1.20	6.19E-01	BG378791	Rn.12340
1	233845989	1389581_at	Il33	interleukin 33	1.06	7.69E-01	-4.82	1.99E-07	-1.13	9.60E-01	1.07	7.99E-01	BF390510	Rn.51945
1	233848805	1373970_at	Il33	interleukin 33	1.02	8.77E-01	-4.38	1.56E-07	-1.10	9.64E-01	1.01	9.90E-01	AI716248	Rn.1927
1	233911139	1378702_at	---	Transcribed locus	1.05	7.35E-01	1.65	4.91E-03	-1.01	9.95E-01	1.02	9.45E-01	BF413730	Rn.62443
1	234004644	1373117_at	---	Transcribed locus strongly similar to ubiquitin-like containing PHD and RING finger domains, 2	1.20	6.44E-02	2.22	1.42E-06	-1.03	9.74E-01	-1.22	3.20E-01	BI275765	Rn.2173
1	234010715	1379334_at	Gldc	Glycine dehydrogenase	-1.32	5.40E-03	1.01	9.85E-01	-1.02	9.79E-01	-1.07	7.69E-01	BF547086	Rn.7547
1	234011046	1376427_a_at	---	Transcribed locus	-2.03	1.18E-03	1.11	7.51E-01	1.00	9.97E-01	-1.02	9.55E-01	AI029729	Rn.17101
1	234148696	1379240_at	---	Transcribed locus	1.02	9.29E-01	1.34	8.00E-03	1.01	9.97E-01	-1.00	9.96E-01	AA893453	---
1	236029865	1368784_at	Acf	apobec-1 complementation factor	-2.41	8.27E-06	-4.38	3.65E-06	1.01	9.97E-01	1.05	7.78E-01	NM_133400	Rn.81027

1	237532482	1381816_at	---	Similar to hypothetical protein FLJ11218	1.26	2.63E-02	1.12	5.61E-01	1.18	3.30E-01	1.11	4.96E-01	BE119617	Rn.57170
1	237801122	1382008_at	---	Similar to hypothetical protein FLJ11218	1.35	7.21E-05	1.07	7.02E-01	1.03	9.91E-01	1.14	5.04E-01	AI044348	Rn.15885
1	238436908	1384842_s_at	Fas	Tumor necrosis factor receptor superfamily, member 6	1.01	9.85E-01	-2.30	9.22E-03	-1.27	5.06E-01	1.02	9.47E-01	AI231531	Rn.24581
1	238630381	1368075_at	Lipa	lipase A, lysosomal acid	-1.01	9.63E-01	-1.43	4.71E-03	-1.09	9.74E-01	1.06	7.14E-01	NM_012732	Rn.48656
1	238773058	1369836_at	Ifit1	interferon-induced protein - tetratricopeptide repeats 1	1.70	3.68E-01	2.08	3.89E-01	1.64	3.88E-01	4.29	3.06E-02	NM_020096	Rn.48677
1	239134330	1395503_at	Ears2	glutamyl-tRNA synthetase 2 mitochondrial	1.12	2.71E-01	-1.04	8.51E-01	1.29	4.96E-02	1.17	5.45E-01	BF564382	Rn.48264
1	241907434	1385619_at	Kif11	kinesin-like 1	1.44	8.02E-03	-1.02	9.59E-01	1.04	9.85E-01	-1.02	9.67E-01	BF562797	Rn.12560
1	241911796	1390891_at	Kif11	Kinesin-like 1	1.46	2.51E-04	-1.04	8.75E-01	-1.23	9.54E-01	1.11	5.08E-01	BE116384	Rn.12560
1	242399258	1375857_at	Fer1l3	Myoferlin (Fer-1 like protein 3)	1.27	2.89E-02	1.44	1.96E-02	1.17	9.74E-01	1.24	3.88E-01	AW917760	Rn.8225
1	242637190	1371762_at	Rbp4	retinol binding protein 4, plasma	-2.74	5.04E-06	1.02	9.17E-01	1.06	9.89E-01	1.26	3.76E-01	AA858962	Rn.35762
1	243377878	1387328_at	Cyp2c	Cytochrome P450, subfamily IIC (mephenytoin 4-hydroxylase)	-7.11	8.23E-04	1.05	7.87E-01	-1.05	9.63E-01	1.01	9.77E-01	NM_019184	Rn.10870
1	243955564	1370580_a_at	Cyp2c37	cytochrome P450, 2c37	1.04	8.80E-01	-1.34	1.75E-02	-1.02	9.89E-01	1.01	9.78E-01	M18336	Rn.7245
1	245606005	1380940_at	---	Transcribed locus	-1.09	7.16E-01	-1.45	4.88E-02	1.26	6.30E-01	-1.23	4.00E-01	BF402603	Rn.60083
1	246526313	1380053_at	---	Transcribed locus strongly similar to transmembrane protein TM9SF3	-1.31	8.98E-03	-1.42	2.99E-02	-1.33	1.80E-01	-1.18	2.86E-01	BF397371	Rn.63785
1	246527703	1379627_at	Tm9sf3	transmembrane 9 superfamily member 3	-1.16	2.22E-01	-1.30	3.20E-02	-1.30	5.02E-01	-1.04	8.97E-01	AW534630	Rn.38604
1	246892433	1390719_at	---	Transcribed locus	-1.44	6.44E-02	-1.59	2.77E-02	-1.20	7.08E-01	-1.53	2.11E-01	BI294889	Rn.21735
1	247199740	1382155_at	---	Transcribed locus	7.02	2.21E-09	4.23	1.99E-07	8.22	2.87E-09	3.94	8.10E-05	AA875055	Rn.32203
1	247207051	1397902_at	---	Transcribed locus	1.68	3.46E-03	1.48	1.63E-02	1.70	5.12E-02	1.32	1.21E-01	BF548780	---
1	247210645	1391441_at	---	Transcribed locus	-1.47	1.32E-03	-1.31	2.44E-01	-1.15	9.15E-01	-1.55	1.30E-02	AA899943	Rn.8955
1	247464374	1390815_at	---	Transcribed locus	1.49	6.49E-03	1.11	5.43E-01	1.04	9.74E-01	1.10	5.76E-01	BF282870	Rn.28026
1	248246887	1382699_s_at	Hps1	Hermansky-Pudlak syndrome 1 homolog	-1.28	1.56E-03	-1.79	6.35E-03	-1.09	9.67E-01	1.31	8.40E-02	AW920326	Rn.49280
1	248272489	1398031_at	---	Transcribed locus	1.27	4.07E-03	1.43	8.55E-03	1.15	6.23E-01	1.23	1.28E-01	BF406285	Rn.59908
1	248275767	1378802_at	RGD:1303232	Phytn_dehydro and Pyr_redox domain containing protein	1.44	1.31E-04	3.18	1.34E-06	1.40	6.58E-03	1.98	1.46E-03	AI105366	Rn.33808
1	248818538	1368381_at	Crtac1	cartilage acidic protein 1	1.13	3.62E-01	1.05	8.52E-01	1.09	9.35E-01	1.70	1.79E-02	NM_134401	Rn.15989
1	248964264	1372342_at	Mrvldc1	MARVEL (membrane-associating) domain containing 1	1.03	8.43E-01	1.37	3.06E-02	1.13	9.41E-01	1.13	7.42E-01	AI176583	Rn.1215
1	248986211	1370252_at	---	Transcribed locus	1.22	2.25E-03	1.19	3.47E-01	1.01	9.95E-01	1.10	6.49E-01	BI282724	Rn.13633
1	249076556	1393126_at	Ubtd1	ubiquitin domain containing 1	1.08	4.37E-01	1.21	4.82E-02	1.25	2.61E-01	-1.05	8.03E-01	BI293253	Rn.41952
1	249351593	1398473_at	---	Transcribed locus	1.24	2.95E-03	1.28	5.40E-02	1.15	5.43E-01	1.16	2.72E-01	BI290651	Rn.19119
1	249454987	1371580_at	---	Similar to band 7 protein (35.3 kD) (4N53)	1.22	1.55E-02	1.12	5.50E-01	1.06	9.70E-01	-1.02	9.56E-01	AI102725	Rn.36741
1	249616941	1386889_at	Scd2	stearoyl-Coenzyme A desaturase 2	-1.56	1.77E-03	-1.05	8.01E-01	1.08	9.76E-01	1.11	8.00E-01	BE107760	Rn.2627
1	249616941	1367668_a_at	Scd2	stearoyl-Coenzyme A desaturase 2	-2.32	5.88E-07	-1.23	3.62E-01	-1.15	9.74E-01	-1.02	9.80E-01	NM_031841	Rn.2627
1	249717541	1370355_at	Scd1	stearoyl-Coenzyme A desaturase 1	2.31	3.32E-01	14.72	3.50E-04	2.75	5.43E-01	4.56	3.63E-01	J02585	Rn.1023
1	249857514	1375628_at	Ndufb8	NADH dehydrogenase (ubiquinone) 1b subcomplex 8	-1.22	8.02E-03	-1.18	2.08E-01	-1.11	9.54E-01	1.03	9.43E-01	AI059654	Rn.3383
1	250338099	1398472_at	Peo1	progressive external ophthalmoplegia 1	1.98	1.86E-06	2.13	9.10E-06	1.88	1.60E-04	1.86	2.31E-03	AA925995	Rn.44164
1	251226644	1383025_at	---	Transcribed locus	-1.07	7.03E-01	-1.26	4.22E-02	-1.07	9.55E-01	-1.14	4.88E-01	BE097791	Rn.41557
1	251269244	1373987_at	Kcnip2	Kv channel-interacting protein 2	-1.06	7.72E-01	-1.11	5.93E-01	-1.43	6.82E-01	-1.79	4.84E-02	AI410448	---
1	251294346	1374960_at	---	Similar to 9130011E15Rik protein	1.58	1.87E-03	1.16	3.07E-01	1.83	5.46E-05	1.45	2.41E-01	BM390080	Rn.8704
1	251295100	1384117_at	---	Similar to 9130011E15Rik protein	1.43	8.35E-04	1.16	3.80E-01	1.58	1.09E-03	1.24	2.16E-01	BF555676	Rn.23632
1	251958407	1385042_at	LOC361769	similar to Su(fu) protein	-1.25	4.46E-02	1.03	9.03E-01	-1.12	9.30E-01	-1.11	4.84E-01	BM387871	Rn.82107
1	252160167	1390543_at	---	Transcribed locus	1.08	6.37E-01	1.37	1.97E-02	1.10	9.55E-01	1.08	6.32E-01	BF396416	Rn.8770
1	252228606	1387123_at	Cyp17a1	cytochrome P450, family 17, subfamily a, polypeptide 1	-1.03	9.06E-01	-	2.96E-09	1.05	9.60E-01	1.01	9.75E-01	NM_012753	Rn.10172

							18.13							
1	252467278	1391413_at	Nt5c2	5'-nucleotidase, cytosolic II	1.42	1.56E-03	1.07	8.03E-01	1.18	8.63E-01	1.08	6.39E-01	BM387140	Rn.25070
1	252622559	1389760_at	Rnf134	ring finger protein 134	-1.14	2.44E-01	-1.72	1.32E-03	-1.42	5.11E-01	-1.74	2.80E-03	AA858786	Rn.16897
1	253140049	1373295_at	Obfc1	oligonucleotide/oligosaccharide-binding fold	1.04	8.68E-01	-1.11	4.82E-01	2.83	1.09E-03	1.10	4.69E-01	BI281889	Rn.15899
1	253498365	1389832_at	Gsto1	glutathione S-transferase omega 1	1.01	9.07E-01	1.71	2.00E-05	1.17	3.04E-01	1.13	3.88E-01	BE113459	---
1	259355566	1370160_at	Xpnpep1	X-prolyl aminopeptidase (aminopeptidase P) 1, soluble	1.04	8.22E-01	1.25	3.71E-02	-1.02	9.88E-01	-1.05	7.43E-01	AI178742	Rn.25763
1	259720176	1368963_at	Mxi1	Max interacting protein 1	1.32	7.25E-04	1.05	8.01E-01	1.13	9.38E-01	1.15	4.10E-01	NM_013160	Rn.37510
1	259800723	1372093_at	Mxi1	Max interacting protein 1	1.26	5.25E-04	1.15	3.84E-01	1.13	9.30E-01	1.06	8.59E-01	AI409308	Rn.1297
1	261578072	1372452_at	Gpam	Glycerol-3-phosphate acyltransferase, mitochondrial	-1.33	2.29E-02	-1.44	1.03E-01	1.10	9.60E-01	1.05	8.01E-01	BG666882	Rn.8234
1	261580925	1382986_at	---	Transcribed locus	-1.34	1.18E-03	-1.58	5.36E-02	1.11	9.60E-01	1.14	5.36E-01	BI288209	Rn.36575
1	261581210	1369758_at	Gpam	Glycerol-3-phosphate acyltransferase, mitochondrial	-1.15	2.29E-01	-1.41	4.49E-02	1.10	9.74E-01	1.27	3.20E-01	U36771	Rn.44456
1	263033222	1383495_at	Dclre1a	DNA cross-link repair 1A, PSO2 homolog	1.05	6.41E-01	-1.37	1.23E-02	-1.05	9.74E-01	1.00	9.90E-01	AI556858	Rn.7714
1	264023960	1382151_at	Trub1	TruB pseudouridine (psi) synthase homolog 1	-1.21	5.40E-03	-1.01	9.78E-01	-1.10	9.62E-01	-1.17	4.19E-01	AI145625	Rn.24324
1	264698911	1388738_at	---	Similar to KIAA0534 protein	1.27	5.63E-04	1.67	7.53E-02	1.21	8.80E-01	-1.05	8.50E-01	AI411227	Rn.12950
1	265516187	1373480_at	---	Transcribed locus	-1.42	4.52E-03	1.02	9.42E-01	-1.07	9.49E-01	-1.78	1.16E-01	BG670210	Rn.18598
1	266184962	1385185_at	---	Transcribed locus	1.56	1.44E-03	-1.01	9.80E-01	2.73	1.90E-03	1.23	1.28E-01	BF388679	---
1	267340629	1389298_at	---	Similar to 2700078E11Rik protein	-1.23	3.29E-03	-1.40	5.49E-02	-1.29	2.00E-02	-1.37	1.07E-01	AI412864	Rn.13358
1	---	1389527_at	LOC378467	promethin	1.30	8.57E-04	1.33	2.16E-01	1.60	8.67E-05	1.39	1.46E-01	BM384073	Rn.7304
1	---	1374657_at	---	Similar to RIKEN 1810020D17	1.17	4.46E-02	-1.11	6.50E-01	-1.02	9.89E-01	1.06	8.19E-01	AA848437	Rn.6362
1	---	1384151_at	Sirt3	Sirtuin 3	-1.23	1.09E-02	1.05	7.94E-01	-1.17	5.94E-01	-1.24	1.18E-01	AI578392	Rn.18788
1	---	1368497_at	Abcc2	ATP-binding cassette, sub-family C (CFTR/MRP), member 2	1.49	1.56E-03	-1.15	5.51E-01	1.04	9.74E-01	1.15	3.88E-01	NM_012833	Rn.10265
2	---	1368512_a_at	Enpep	glutamyl aminopeptidase	-1.06	6.36E-01	1.33	2.15E-02	-1.13	8.89E-01	-1.25	1.80E-01	AF214568	Rn.53979
2	---	1383248_at	Fmo5	flavin containing monooxygenase 5	-1.23	1.17E-01	-1.29	2.57E-02	-1.17	8.20E-01	-1.11	5.65E-01	AI454611	Rn.7038
2	180397196	1378929_at	---	Transcribed locus	-1.21	4.88E-02	-1.08	7.22E-01	1.13	9.54E-01	-1.12	5.14E-01	BE116137	Rn.16728
2	192343763	1382611_at	---	Transcribed locus	-1.03	8.80E-01	-1.37	4.54E-02	1.00	1.00E+00	-1.04	8.25E-01	AA945938	Rn.24100
2	199210309	1398866_at	Magi3	membrane-associated guanylate kinase-related	-1.06	6.84E-01	-1.29	3.62E-02	-1.10	9.74E-01	-1.15	5.70E-01	AF255614	Rn.22579
2	210152887	1392915_at	Col11a1	Procollagen, type XI, alpha 1	1.21	1.71E-02	-1.03	9.18E-01	-1.25	9.54E-01	1.08	6.11E-01	BM389291	Rn.260
2	229621058	1385444_at	---	Transcribed locus	-1.17	4.74E-02	-1.04	8.54E-01	1.24	8.82E-01	1.20	5.61E-01	AI071994	---
2	234116850	1368277_at	Ppp3ca	protein phosphatase 3, catalytic subunit, alpha isoform	1.04	6.91E-01	-1.28	2.08E-02	-1.06	9.44E-01	1.03	9.20E-01	NM_017041	Rn.6866
2	240864081	1368220_at	Gtf2b	general transcription factor IIB	1.01	9.29E-01	-1.30	3.86E-02	1.02	9.91E-01	-1.01	9.68E-01	NM_031041	Rn.6109
2	244624597	1373146_at	Ssx2ip	synovial sarcoma, X breakpoint 2 interacting protein	1.03	8.15E-01	1.31	3.83E-02	1.09	9.67E-01	1.06	8.09E-01	AI716240	Rn.21417
2	247417022	1382780_at	---	Transcribed locus	1.06	7.50E-01	-1.51	8.66E-03	1.10	9.05E-01	1.02	9.45E-01	AI059229	Rn.21060
2	31239665	1398281_at	Tpmt	thiopurine methyltransferase	-1.00	9.86E-01	-1.38	4.46E-02	-1.02	9.87E-01	1.13	4.84E-01	NM_031329	Rn.31429
2	46469312	1372750_at	Fst	Follistatin	1.15	4.03E-01	1.05	7.94E-01	-1.37	2.55E-02	-1.41	4.58E-01	AI102517	Rn.2743
2	53619481	1384580_at	---	Transcribed locus	-1.01	9.58E-01	1.36	1.83E-01	-1.30	8.96E-01	2.57	4.30E-03	AI045191	Rn.16145
2	84666901	1393330_at	---	Transcribed locus	1.26	1.47E-02	-1.07	8.08E-01	-1.04	9.89E-01	1.04	8.64E-01	AI137898	---
2	85097492	1391556_at	---	Transcribed locus	1.32	9.74E-04	-1.00	9.94E-01	1.03	9.74E-01	-1.22	3.06E-01	AW524218	Rn.43151
2	85102158	1390437_at	---	Transcribed locus	1.24	2.33E-02	-1.12	5.24E-01	1.16	8.45E-01	-1.30	3.06E-01	BE116385	Rn.14619
3	108987885	1385189_at	Duoxa1	dual oxidase maturation factor 1	1.08	5.49E-01	2.00	8.77E-04	-1.02	9.90E-01	1.05	9.10E-01	BG372602	Rn.66938
3	120206688	1375911_at	RGD735140	hypothetical protein LK44	-1.02	9.35E-01	1.30	4.83E-02	1.22	8.84E-01	1.12	8.64E-01	AI171772	Rn.6449
3	134408813	1372365_at	---	Transcribed locus	1.12	3.66E-01	-1.31	3.83E-02	-1.22	9.41E-01	-1.32	3.12E-01	BM392293	Rn.34193

3	137065404	1368901_at	Thbd	thrombomodulin	-1.36	1.08E-03	-1.11	7.00E-01	-1.22	8.81E-01	-1.16	5.55E-01BG666306	Rn.15127
3	141164582	1372162_at	Acas2l	acetyl-Coenzyme A synthetase 2 (AMP forming)-like	-1.22	2.86E-03	-1.02	9.35E-01	1.41	9.41E-01	-1.02	9.71E-01AI176565	Rn.3528
3	144966613	1383117_at	Pxmp4	peroxisomal membrane protein 4	1.02	9.24E-01	1.57	2.77E-02	1.21	8.87E-01	1.17	4.09E-01AI232414	Rn.7752
3	144967165	1393421_at	Pxmp4	peroxisomal membrane protein 4	1.02	9.10E-01	1.64	9.16E-03	1.13	9.55E-01	1.14	4.87E-01BG664011	Rn.7752
3	156765428	1375417_at	---	Transcribed locus	-1.06	5.08E-01	-1.40	1.92E-02	-1.07	9.22E-01	1.00	9.98E-01BM391419	Rn.2952
3	3387045	1371390_at	Tubb2c	tubulin, beta2c	1.05	6.36E-01	-1.39	4.64E-02	-1.12	9.62E-01	1.06	7.69E-01AA892044	Rn.3823
3	42334922	1375870_a_at	Rbms1	RNA binding motif, single stranded interacting protein 1	-1.07	7.26E-01	-1.34	3.20E-02	-1.13	9.56E-01	-1.04	9.13E-01BF420140	Rn.12152
3	59588738	1379814_at	---	CDNA clone IMAGE:7302159	-1.20	2.37E-01	-1.74	1.32E-03	-1.03	9.89E-01	-1.22	4.17E-01AW532796	Rn.14694
3	87766997	1369693_a_at	Slc1a2	solute carrier family 1 (glial high affinity glutamate transporter), member 2	-1.01	9.66E-01	-1.96	2.01E-02	-1.11	9.17E-01	-1.04	8.99E-01NM_017215	Rn.10240
3	92267350	1392077_at	C11orf8	Chromosome 11 open reading frame 8	-1.21	1.49E-02	1.01	9.66E-01	1.33	9.09E-01	-1.00	9.94E-01AW536022	Rn.48596
4	116573982	1376792_at	---	Transcribed locus strongly similar to BC014699	1.01	9.72E-01	-1.40	1.32E-03	-1.01	9.92E-01	-1.07	6.64E-01AW251313	Rn.828
4	117760903	1378365_at	---	Transcribed locus	-1.49	8.35E-04	-1.01	9.85E-01	1.02	9.81E-01	-1.08	5.91E-01AW529483	Rn.41289
4	118979266	1384392_at	Cyp26b1	cytochrome P450, family 26, subfamily b, polypeptide 1	-2.03	1.48E-03	-2.10	6.07E-02	-1.52	7.39E-01	-1.35	4.12E-01BF397093	Rn.9325
4	123167718	1375247_at	---	Transcribed locus	-1.07	6.39E-01	1.26	4.34E-02	1.05	9.85E-01	-1.08	8.25E-01BG372713	---
4	144384326	1369415_at	Bhlhb2	Basic helix-loop-helix domain containing, class B2	1.47	5.00E-02	1.68	1.69E-01	1.11	9.79E-01	1.46	5.13E-01NM_053328	Rn.81055
4	144389968	1379483_at	Bhlhb2	Basic helix-loop-helix domain containing, class B2	1.26	3.74E-01	1.93	3.27E-02	1.13	9.63E-01	1.37	3.64E-01AI548256	---
4	153760608	1388583_at	---	Transcribed locus	1.17	2.23E-02	-1.08	6.74E-01	-1.06	9.74E-01	-1.51	2.16E-01BF283398	---
4	156719621	1368135_at	Ninj2	ninjurin 2	-1.01	9.81E-01	1.48	2.77E-02	-1.07	9.64E-01	1.05	8.28E-01NM_021595	Rn.35505
4	157540210	1377759_at	Bid	BH3 interacting domain death agonist	1.17	1.54E-01	-1.36	4.64E-02	-1.00	1.00E+00	1.02	9.59E-01BG666928	Rn.31142
4	167055577	1371680_at	---	Transcribed locus	-1.17	2.93E-02	-1.32	7.64E-02	-1.19	8.54E-01	-1.33	1.94E-01BI303631	Rn.36100
4	173816456	1395687_at	Atf7ip	activating transcription factor 7 interacting protein	1.09	7.27E-01	-1.26	3.20E-02	1.35	7.88E-01	1.17	5.05E-01BE113420	Rn.50750
4	178087788	1371996_at	---	Transcribed locus	-1.04	6.70E-01	-1.29	3.27E-02	-1.04	9.70E-01	-1.01	9.66E-01AI232384	Rn.1477
4	178089913	1385728_at	Aebp2	AE binding protein 2	-1.08	8.18E-01	-1.48	2.59E-02	-1.08	9.54E-01	-1.14	5.70E-01BE108006	Rn.51376
4	179798409	1387974_a_at	Slc21a4	kidney specific organic anion transporter	1.29	7.72E-03	1.01	9.66E-01	-1.04	9.65E-01	1.05	8.34E-01AF445995	Rn.10583
4	18639988	1385098_at	---	Transcribed locus	-1.03	9.02E-01	-1.51	8.81E-03	1.05	9.87E-01	1.25	5.60E-01AW526343	Rn.46170
4	1951630	1368511_at	Bhlhb3	basic helix-loop-helix domain containing, class B3	2.00	3.98E-03	1.30	1.87E-01	1.39	4.79E-01	1.22	4.64E-01NM_133303	Rn.10784
4	30231615	1389718_at	---	Transcribed locus	-1.01	9.36E-01	1.02	9.59E-01	1.58	2.55E-02	1.23	6.91E-01AA956417	Rn.23170
4	30277254	1378074_at	---	Transcribed locus	-1.73	2.89E-02	-1.58	3.69E-01	-1.12	8.26E-01	-1.26	6.49E-01AI175045	Rn.11766
4	30278907	1369150_at	Pdk4	pyruvate dehydrogenase kinase, isoenzyme 4	-1.35	4.72E-02	-1.59	3.51E-01	-1.18	9.33E-01	-1.10	9.15E-01NM_053551	Rn.30070
4	33777889	1389679_at	---	Transcribed locus strongly similar to glucocorticoid induced transcript 1	-1.30	1.44E-03	-1.20	5.08E-01	-1.10	9.55E-01	-1.37	3.49E-01AI178923	Rn.25041
4	63005373	1392510_at	RGD1308013	similar to hypothetical protein B230314O19	1.00	9.87E-01	1.31	3.84E-02	-1.08	9.60E-01	1.25	4.96E-01BF283618	Rn.23539
4	66540360	1385739_at	---	Transcribed locus	-1.04	7.89E-01	-1.32	3.27E-02	-1.15	8.80E-01	1.03	9.16E-01BE120816	Rn.53919
4	66544163	1383332_at	---	Homeodomain interacting protein kinase 2	-1.11	4.76E-01	-1.32	3.83E-02	-1.13	9.60E-01	1.36	5.45E-01BI281615	Rn.50802
4	77489449	1377824_a_at	RGD1306512	similar to igf2 mRNA-binding protein 3	-1.01	9.27E-01	1.68	2.10E-03	-1.01	9.92E-01	-1.06	7.08E-01AI230709	Rn.31564
4	77489449	1390860_at	RGD1306512	similar to igf2 mRNA-binding protein 3	-1.08	5.54E-01	1.60	2.58E-02	1.05	9.63E-01	1.02	9.54E-01AI230709	Rn.31564
4	84015299	1373975_at	---	Thioether S-methyltransferase	-1.25	3.41E-02	-1.22	6.14E-01	-1.19	9.55E-01	-1.22	6.45E-01AI232716	Rn.19133
4	75834713	1382206_a_at	Akap2	A kinase (PRKA) anchor protein 2	1.27	1.55E-02	1.04	8.84E-01	-1.07	9.70E-01	1.13	4.90E-01BF398063	Rn.35524
4	75860093	1385566_at	Akap2	A kinase (PRKA) anchor protein 2	1.20	4.67E-02	1.00	9.89E-01	-1.03	9.89E-01	-1.03	9.22E-01AI072144	Rn.21728
5	116749562	1370706_a_at	Cyp2j9	cytochrome P450, family 2, subfamily j, polypeptide 9	1.18	4.48E-02	1.03	9.46E-01	-1.06	9.54E-01	1.16	6.65E-01U39943	Rn.37480
5	125711811	1384225_at	---	Transcribed locus	-1.05	8.88E-01	2.50	2.62E-04	-1.24	9.44E-01	-1.00	9.95E-01BI289963	Rn.54031
5	127669581	1372012_at	---	Transcribed locus	-1.02	9.75E-01	1.67	3.61E-02	1.22	5.43E-01	1.10	7.61E-01BF417479	Rn.9470

5	136316202	1368717_at	Faah	fatty acid amide hydrolase	-1.07	6.39E-01	1.24	3.86E-02	-1.05	9.74E-01	1.01	9.74E-01	NM_024132	Rn.10619
5	137121609	1393228_at	RGD1310014	similar to RIKEN cDNA A830048M07	1.37	3.87E-02	1.13	4.31E-01	1.06	9.79E-01	-1.04	8.74E-01	AA957707	Rn.9366
5	138002009	1388475_at	---	Transcribed locus	1.04	7.58E-01	1.24	4.48E-02	1.08	9.04E-01	1.08	5.89E-01	AA899150	---
5	153275132	1380433_at	---	Transcribed locus moderately similar to LOC360381	-1.00	9.94E-01	1.55	9.29E-03	1.18	8.48E-01	1.12	7.28E-01	AI229240	Rn.22923
5	167250629	1368460_at	Slc2a5	solute carrier family 2, member 5	1.05	6.06E-01	1.65	2.15E-03	1.02	9.87E-01	1.11	6.77E-01	NM_031741	Rn.10900
5	168195933	1378745_at	---	Transcribed locus	1.39	2.44E-02	1.52	2.98E-01	1.23	7.31E-01	1.20	4.53E-01	BG374483	Rn.73043
5	23458632	1393345_at	---	Transcribed locus	1.21	1.22E-02	1.14	5.52E-01	-1.02	9.91E-01	1.05	8.09E-01	BF561145	Rn.64084
5	49544500	1370381_at	Pnrc1	proline rich 2	-1.36	4.38E-02	-1.26	2.38E-01	-1.09	9.54E-01	-1.16	5.63E-01	U61729	Rn.10967
5	58938315	1372237_at	---	Transcribed locus	-1.04	6.67E-01	-1.29	2.60E-02	-1.04	9.60E-01	-1.08	6.89E-01	BI298306	Rn.39045
5	66029808	1397732_at	---	Transcribed locus	1.03	8.64E-01	1.49	3.76E-02	1.01	9.94E-01	1.01	9.75E-01	BF408724	Rn.62173
5	66120335	1386656_at	---	Transcribed locus strongly similar to protein KIAA0141	1.03	8.30E-01	2.43	9.13E-03	-1.01	9.89E-01	-1.02	9.04E-01	BF558870	Rn.24326
5	66120989	1378786_at	---	Transcribed locus strongly similar to protein KIAA0141	1.02	9.34E-01	2.07	2.81E-02	1.01	9.94E-01	-1.09	7.25E-01	AI228437	Rn.24326
5	66250741	1387508_at	Baat	bile acid-Coenzyme A: amino acid N-acyltransferase	-1.91	1.36E-02	-1.10	5.37E-01	1.02	9.89E-01	1.01	9.62E-01	NM_017300	Rn.11129
5	87772852	1390456_at	---	Transcribed locus	-1.04	8.49E-01	-1.26	2.77E-02	1.05	9.66E-01	1.06	7.93E-01	AW524471	Rn.46564
6	1142802	1398622_at	---	Cysteine-rich motor neuron 1	-1.10	3.50E-01	-1.44	2.15E-02	1.17	6.48E-01	-1.02	9.60E-01	AI703807	Rn.24755
6	1874020	1377867_at	LOC313837	Glutaminyl-peptide cyclotransferase	-1.35	1.01E-02	1.05	8.41E-01	-1.17	9.70E-01	1.04	9.34E-01	BM390001	Rn.7474
6	2465140	1395983_at	---	Transcribed locus	1.04	9.16E-01	-1.75	1.63E-03	1.00	9.98E-01	-1.02	9.47E-01	BF407722	Rn.55953
6	22664926	1388447_at	---	Transcribed locus strongly similar to NP_084275	-1.25	4.84E-02	-1.01	9.76E-01	-1.07	9.67E-01	-1.17	5.08E-01	AA800701	Rn.37644
6	43956734	1398597_at	---	Transcribed locus	-1.31	3.18E-02	-1.05	8.59E-01	-1.37	5.43E-01	-1.20	4.64E-01	AI044699	Rn.30788
6	71899173	1383615_a_at	Hectd1	HECT domain containing 1	-1.01	9.82E-01	-1.37	3.50E-02	1.10	9.67E-01	1.42	4.53E-01	AI059295	Rn.16492
6	104739289	1368609_at	Slc10a1	solute carrier family 10 (sodium/bile acid cotransporter family), member 1	-1.49	9.47E-03	1.06	6.58E-01	-1.00	1.00E+00	-1.01	9.45E-01	NM_017047	Rn.9913
6	136702106	1373833_at	---	Transcribed locus	2.61	1.15E-07	1.98	9.10E-06	2.41	3.13E-06	2.58	2.02E-04	BI288945	Rn.16913
6	138293318	1388272_at	Igh-1a	immunoglobulin heavy chain 1a (serum IgG2a)	-1.56	7.60E-01	-1.31	4.42E-02	-1.03	9.91E-01	-1.08	9.70E-01	AI411947	Rn.968
7	113428605	1379783_at	---	Transcribed locus	1.53	5.00E-02	-1.03	9.24E-01	-1.04	9.74E-01	-1.08	7.10E-01	AI113136	---
7	114780280	1387052_at	Gpt1	glutamic pyruvic transaminase 1, soluble	-1.37	4.11E-02	-1.04	9.24E-01	1.17	9.13E-01	1.25	4.00E-01	NM_031039	Rn.6318
7	120199287	1369919_at	Tef	thyrotroph embryonic factor	1.63	1.18E-02	1.17	6.70E-01	1.31	5.43E-01	1.49	3.19E-01	NM_019194	Rn.81206
7	120213376	1390819_at	---	Transcribed locus	1.42	3.72E-02	1.26	3.53E-01	1.15	7.94E-01	1.05	9.01E-01	BF419374	Rn.59871
7	122816095	1378800_at	---	Transcribed locus	-1.06	7.25E-01	-1.61	2.35E-03	1.03	9.79E-01	-1.03	8.92E-01	BI291526	Rn.61644
7	122828237	1376501_at	Arhgap8	Rho GTPase activating protein 8	-1.08	5.02E-01	-1.74	2.47E-02	1.01	9.93E-01	-1.08	8.27E-01	AA945062	Rn.7830
7	127299720	1376702_at	---	Megalencephalic leukoencephalopathy with subcortical cysts 1	-1.63	9.65E-03	1.38	2.49E-01	-1.01	9.90E-01	1.01	9.80E-01	AI228307	Rn.12794
7	138541852	1371363_at	Gpd1	glycerol-3-phosphate dehydrogenase 1 (soluble)	1.06	6.06E-01	1.34	2.76E-02	1.64	8.20E-01	1.20	5.36E-01	BI277042	---
7	139762616	1369064_a_at	Scn8a	sodium channel, voltage-gated, type 8, alpha polypeptide	1.23	8.98E-03	1.04	8.67E-01	1.05	9.74E-01	1.02	9.26E-01	NM_019266	Rn.10073
7	1710433	1383695_at	Vipr1	vasoactive intestinal peptide receptor 1	-1.00	9.92E-01	-1.24	3.20E-02	1.00	1.00E+00	1.07	8.63E-01	BI301509	Rn.11756
7	24553367	1370333_a_at	Igf1	insulin-like growth factor 1	1.33	2.63E-02	1.23	5.44E-02	-1.05	9.91E-01	1.06	9.04E-01	M15481	Rn.6282
7	24630588	1388469_at	Igf1	Insulin-like growth factor I	1.27	4.80E-02	1.34	6.18E-02	-1.06	9.91E-01	1.06	9.12E-01	AA945615	Rn.4303
7	35313826	1388502_at	---	Inositol polyphosphate-5-phosphatase B	1.62	2.99E-02	1.25	4.69E-01	1.15	8.20E-01	1.34	3.05E-01	AI412624	Rn.2252
7	47261064	1373896_at	---	Transcribed locus	-1.40	3.18E-02	1.03	9.33E-01	-1.10	9.60E-01	1.05	8.59E-01	AA943569	Rn.7596
7	67495874	1373814_at	R3hdm2	R3H domain containing 2	-1.11	3.47E-01	1.11	6.95E-01	1.06	9.79E-01	-1.55	2.28E-02	BI291270	Rn.22359
7	67663517	1391527_at	Nab2	Ngfi-A binding protein 2	-1.13	5.46E-01	-1.16	6.09E-01	-1.20	8.81E-01	-1.40	1.64E-02	BE109296	Rn.53955
7	91777036	1393615_at	---	Transcribed locus	-1.07	7.22E-01	-1.33	3.20E-02	1.18	5.04E-01	1.18	8.12E-01	AI059603	Rn.19226
8	---	1368520_at	Apoa4	apolipoprotein A-IV	-1.00	9.82E-01	1.77	3.18E-04	1.01	9.90E-01	-1.04	8.45E-01	NM_012737	Rn.15739

8	100345077	1378131_at	Slc9a4	Solute carrier family 9 (sodium/hydrogen exchanger), isoform 9	1.28	6.20E-03	-1.04	8.59E-01	-1.21	9.67E-01	1.12	5.02E-01	AI045015	Rn.16082
8	100671280	1382569_at	Paqr9	Progestin and adipoQ receptor family member IX	1.15	3.98E-01	1.47	4.17E-02	1.05	9.79E-01	-1.02	8.82E-01	BF386242	Rn.64169
8	111535452	1377992_at	---	Transcribed locus	1.26	4.61E-02	-1.17	4.34E-01	1.00	1.00E+00	1.09	8.13E-01	AI111775	Rn.26636
8	112129534	1372352_at	Armet	arginine-rich, mutated in early stage tumors	-1.01	9.46E-01	1.35	2.81E-02	-1.04	9.90E-01	1.04	8.89E-01	AI170666	Rn.12213
8	2038610	1387818_at	Casp11	caspase 11	1.17	3.51E-02	1.19	2.26E-01	-1.11	9.36E-01	1.20	2.73E-01	NM_053736	Rn.16195
8	47060850	1387703_a_at	Usp2	Ubiquitin specific protease 2	1.61	2.37E-02	2.31	2.36E-01	1.14	9.70E-01	1.54	6.31E-01	AF106659	Rn.19491
8	47086381	1381811_at	Usp2	Ubiquitin specific protease 2	1.61	4.74E-02	2.31	2.95E-01	1.15	9.67E-01	1.65	5.08E-01	BM385405	Rn.12335
8	55468550	1375542_at	Rdx	Radixin	-1.03	9.02E-01	-1.44	1.09E-02	1.04	9.79E-01	1.03	9.00E-01	AI407092	Rn.7684
8	56857559	1377934_at	---	Transcribed locus	-1.24	3.66E-02	-1.69	5.23E-03	1.01	9.97E-01	-1.06	7.59E-01	BF387289	Rn.17683
8	66507241	1369063_at	Anp32a	acidic (leucine-rich) nuclear phosphoprotein 32 family, member A	1.39	2.49E-02	1.07	9.07E-01	1.10	9.73E-01	1.31	2.72E-01	NM_012903	Rn.10123
8	70222125	1372616_at	---	Transcribed locus	1.03	9.21E-01	3.63	8.55E-03	1.07	9.70E-01	1.12	5.08E-01	AI144796	---
8	79653387	1393971_at	---	Transcribed locus	1.05	7.83E-01	1.68	2.46E-02	1.04	9.74E-01	-1.01	9.77E-01	AW535616	Rn.48527
8	80231779	1368273_at	Mapk6	mitogen-activated protein kinase 6	1.08	3.58E-01	1.30	2.76E-02	-1.02	9.88E-01	1.15	4.66E-01	NM_031622	Rn.2566
8	84660813	1370927_at	Col12a1	procollagen, type XII, alpha 1	-1.03	7.77E-01	1.41	2.77E-02	-1.28	9.70E-01	1.12	8.25E-01	BE108345	Rn.11218
9	12620444	1389066_at	Dscr1l1	Down syndrome critical region gene 1-like 1	-1.02	8.85E-01	-2.11	2.54E-03	1.10	9.70E-01	1.08	8.64E-01	BI274408	Rn.24428
9	12622578	1374235_at	Dscr1l1	Down syndrome critical region gene 1-like 1	1.05	6.51E-01	-2.27	2.70E-03	1.06	9.67E-01	1.22	5.43E-01	AI138048	Rn.23797
9	3307609	1382627_at	---	Special AT-rich sequence binding protein 1	1.02	9.45E-01	1.00	9.90E-01	1.12	9.74E-01	1.65	4.69E-02	AA956525	---
9	42932597	1372646_at	RGD1305645	similar to RIKEN cDNA 1500015O10	1.23	5.40E-03	1.01	9.85E-01	-1.14	8.81E-01	1.33	1.28E-01	BF418649	Rn.16593
9	61589021	1382995_at	Nrp2	Neuropilin 2	-1.15	3.48E-01	-1.49	4.02E-02	-1.20	9.37E-01	-1.26	3.05E-01	AA859669	Rn.57331
9	61591097	1378171_at	Nrp2	Neuropilin 2	-1.11	5.72E-01	-1.57	8.12E-04	-1.16	9.44E-01	-1.09	6.92E-01	BF409060	Rn.49567
9	74642893	1371542_at	Tuba4a	Alpha-tubulin 4	1.02	9.26E-01	-1.56	1.49E-02	1.02	9.92E-01	1.94	4.62E-01	BI284599	Rn.6673
9	74698785	1367904_at	Resp18	regulated endocrine-specific protein 18	1.10	6.91E-01	-1.55	2.57E-02	1.02	9.88E-01	1.22	5.53E-01	NM_019278	Rn.2225
10	13125542	1393241_at	---	Transcribed locus	-1.02	9.46E-01	2.22	3.20E-02	1.01	9.95E-01	-1.05	8.89E-01	BI295197	---
10	14128644	1387816_at	---	Transcribed locus	1.13	4.17E-01	1.41	1.45E-02	1.03	9.75E-01	-1.01	9.76E-01	NM_053329	Rn.7327
10	20939793	1368993_at	Odz2	odd Oz/ten-m homolog 2 (Drosophila)	1.03	9.27E-01	-1.41	4.35E-02	1.02	9.91E-01	-1.05	8.58E-01	NM_020088	Rn.53894
10	21391324	1375331_at	---	Transcribed locus	1.08	3.83E-01	-1.67	1.45E-02	1.04	9.74E-01	1.00	9.92E-01	BF418058	Rn.63527
10	46305027	1387908_at	Rasd1	RAS, dexamethasone-induced 1	-1.01	9.81E-01	-1.00	9.94E-01	1.09	9.74E-01	2.04	3.23E-02	AF239157	Rn.7500
10	56913694	1370149_at	Asgr1	asialoglycoprotein receptor 1	1.46	9.06E-03	1.01	9.53E-01	1.05	9.55E-01	-1.11	7.22E-01	NM_012503	Rn.44300
10	57077030	1386833_at	Bcl6b	B-cell CLL/lymphoma 6, member B	-1.07	6.99E-01	1.01	9.82E-01	-1.07	9.77E-01	-1.51	2.85E-03	BF554138	Rn.20865
10	57545284	1367605_at	Pfn1	profilin 1	1.09	2.63E-01	1.28	2.60E-02	1.00	1.00E+00	1.02	9.32E-01	NM_022511	Rn.1152
10	59290195	1385171_s_at	---	Transcribed locus	-1.21	4.98E-02	1.07	7.46E-01	1.01	9.97E-01	2.58	4.78E-01	BE115612	---
10	72347376	1382754_at	---	Transcribed locus	1.08	5.86E-01	1.50	2.14E-02	1.02	9.88E-01	-1.07	7.69E-01	H33080	Rn.14621
10	75221353	1390117_at	---	Transcribed locus	-1.49	6.37E-02	-1.39	2.15E-02	-1.22	9.46E-01	-1.10	5.91E-01	BG372455	Rn.16601
10	78760103	1374709_at	---	Transcribed locus	1.32	4.46E-02	1.06	9.01E-01	1.20	8.20E-01	1.04	8.75E-01	AI406795	Rn.19878
10	78762133	1385585_at	---	Transcribed locus	1.39	3.08E-02	1.18	5.64E-01	1.18	9.05E-01	1.04	8.17E-01	AI111674	---
10	83208696	1392952_at	---	Transcribed locus	-1.26	5.92E-03	1.27	4.71E-01	1.03	9.88E-01	-1.17	5.08E-01	BI277131	---
10	83636918	1388116_at	Col1a1	collagen, type 1, alpha 1	1.15	6.87E-01	1.36	5.23E-03	-1.19	9.80E-01	-1.04	9.76E-01	BI285575	Rn.2953
10	87555387	1370816_at	Nr1d1	nuclear receptor subfamily 1, group D, member 1	1.36	2.54E-03	1.28	4.63E-01	1.24	8.02E-01	1.16	6.86E-01	M25804	Rn.29848
10	92844349	1374900_at	Gosr2	golgi SNAP receptor complex member 2	1.23	1.64E-02	1.12	4.68E-01	1.11	9.30E-01	-1.09	5.89E-01	BM388807	Rn.13518
10	92845193	1375438_at	Gosr2	golgi SNAP receptor complex member 2	1.23	5.47E-03	1.13	3.06E-01	-1.02	9.89E-01	1.02	9.29E-01	AA964687	Rn.12617
10	92846566	1370031_at	Gosr2	golgi SNAP receptor complex member 2	1.42	2.46E-03	1.16	4.35E-01	-1.21	8.50E-01	1.11	6.58E-01	NM_031685	Rn.10734

10	96908433	1392983_at	---	Transcribed locus	1.03	8.44E-01	-1.27	3.42E-02	-1.05	9.55E-01	1.02	9.17E-01	BE103250	Rn.15873
11	32442818	1388686_at	Dscr1	Down syndrome critical region homolog 1	1.05	8.34E-01	1.50	2.91E-02	-1.32	8.58E-01	1.01	9.79E-01	AI170193	Rn.12942
11	56393581	1381206_at	---	Transcribed locus	1.18	3.50E-01	-2.08	3.06E-02	-1.01	9.94E-01	1.09	6.60E-01	BE121026	Rn.53717
11	64157425	1370445_at	Pla1a	phospholipase A1 member A	-1.13	4.07E-01	-1.49	2.35E-03	-1.28	5.87E-01	1.01	9.84E-01	D88666	Rn.10696
11	66537744	1368195_at	Hspbap1	Hspb associated protein 1	1.22	2.49E-02	-1.01	9.74E-01	-1.01	9.94E-01	-1.17	3.38E-01	NM_134419	Rn.25280
11	69159862	1381998_at	Znf148	Zinc finger protein 148	1.02	9.32E-01	-1.28	3.64E-02	-1.03	9.88E-01	-1.00	9.98E-01	AW519820	Rn.19989
11	80334440	1387082_at	Fetub	fetuin beta	1.60	5.25E-04	1.06	6.73E-01	1.04	9.79E-01	-1.03	8.81E-01	NM_053348	Rn.2464
11	84962339	1372920_at	---	Proline dehydrogenase	-1.41	3.57E-02	-1.40	2.44E-01	1.06	9.89E-01	-1.50	1.16E-01	AI411345	Rn.41672
11	85084498	1367773_at	Slc25a1	solute carrier family 25, member 1	1.09	3.39E-01	1.30	3.62E-02	-1.08	9.74E-01	1.57	4.17E-01	NM_017307	Rn.8368
12	---	1397788_at	Rimbp2	RIM binding protein 2	1.48	6.57E-03	1.00	9.92E-01	-1.10	9.54E-01	-1.12	6.29E-01	AW521711	Rn.45229
12	17482434	1387234_at	Azgp1	alpha-2-glycoprotein 1, zinc	-1.41	2.14E-02	-1.02	9.22E-01	1.02	9.91E-01	-1.04	8.97E-01	NM_012826	Rn.5007
12	22903157	1395753_at	Eln	elastin	-1.14	2.54E-01	-1.36	4.71E-02	-1.27	8.92E-01	-1.01	9.80E-01	BI274506	Rn.79980
12	286526	1378006_at	---	Transcribed locus	-1.79	1.88E-04	1.02	9.37E-01	-1.31	1.56E-01	-1.85	2.14E-03	AI233832	---
12	28875205	1395991_at	---	Transcribed locus	1.46	3.80E-03	1.01	9.84E-01	-1.08	9.44E-01	-1.14	4.96E-01	BE107556	Rn.50806
12	3695844	1391871_at	---	Transcribed locus	-1.19	3.04E-02	-1.35	1.62E-01	-1.23	5.24E-01	-1.12	3.55E-01	AI763550	---
12	37144481	1369864_a_at	Sds	serine dehydratase	1.29	2.16E-01	1.86	6.35E-03	-1.03	9.83E-01	1.01	9.79E-01	NM_053962	Rn.9918
12	40364270	1390431_at	---	Transcribed locus	-1.03	7.84E-01	-1.28	3.71E-02	-1.07	9.74E-01	-1.18	5.59E-01	AI111559	Rn.22062
12	9546914	1370593_at	Cyp3a11	cytochrome P450, family 3, subfamily a, polypeptide 11	1.03	8.61E-01	-1.52	3.54E-02	1.00	1.00E+00	1.03	9.26E-01	U09742	Rn.37424
13	106349250	1371904_at	Smyd2	SET and MYND domain containing 2	1.09	4.54E-01	1.29	2.62E-02	-1.04	9.89E-01	1.31	4.70E-01	BI294698	Rn.7052
13	33333182	1389377_at	Insig2	insulin induced gene 2	1.04	7.88E-01	-1.69	4.11E-02	1.11	9.37E-01	1.04	8.59E-01	AA851803	Rn.8474
13	33334533	1393690_at	Insig2	insulin induced gene 2	-1.00	9.87E-01	-1.91	1.42E-02	1.12	8.78E-01	-1.02	9.49E-01	AA818627	Rn.16736
13	43799887	1371099_at	---	Polymeric immunoglobulin receptor AATTAA-containing	-1.34	4.43E-02	1.01	9.56E-01	1.07	9.66E-01	-1.13	3.33E-01	U07886	Rn.54456
13	45260373	1374160_at	---	Transcribed locus	1.59	5.52E-02	3.61	2.70E-03	1.10	9.67E-01	2.39	8.40E-02	BE111296	Rn.19339
13	46276780	1368190_at	---	Transcribed locus	-1.43	3.42E-02	1.05	8.27E-01	-1.02	9.89E-01	1.03	9.34E-01	J02941	---
13	48764243	1370057_at	Csrp1	cysteine and glycine-rich protein 1	-1.03	7.72E-01	1.42	3.42E-02	-1.09	9.74E-01	-1.21	4.06E-01	NM_017148	Rn.2401
13	53011980	1383046_at	---	CDNA clone MGC:108856 IMAGE:7370585	-1.58	4.46E-02	-1.05	8.57E-01	-1.02	9.88E-01	1.04	8.02E-01	AA957258	---
13	67474689	1379899_at	---	Transcribed locus	-1.36	1.56E-03	1.02	9.46E-01	1.06	9.80E-01	-1.02	9.65E-01	BI298010	---
13	70072894	1389355_at	---	Transcribed locus	-1.18	4.46E-02	-1.20	5.72E-01	-1.22	9.74E-01	-1.13	4.97E-01	BF285187	Rn.8590
13	78517950	1387053_at	Fmo1	flavin containing monooxygenase 1	1.00	9.95E-01	-1.70	1.32E-03	1.03	9.89E-01	-1.05	9.02E-01	NM_012792	Rn.867
13	80588998	1373947_at	Dpt	dermatopontin	1.05	6.84E-01	1.56	1.40E-02	-1.21	9.67E-01	1.07	6.62E-01	BI278545	Rn.8396
13	85459512	1369957_at	Rgs5	regulator of G-protein signaling 5	1.03	8.62E-01	-1.58	1.06E-02	1.07	9.79E-01	1.56	1.07E-01	NM_019341	Rn.1150
13	87248359	1390912_at	---	CDNA clone IMAGE:7300848	1.07	7.50E-01	-2.06	6.37E-03	-1.03	9.79E-01	1.09	7.12E-01	BE101168	Rn.61111
13	87248897	1390227_at	---	CDNA clone IMAGE:7300848	1.03	7.74E-01	-1.60	2.77E-02	-1.04	9.74E-01	1.09	7.04E-01	AI101017	Rn.43361
13	87293124	1368797_at	Nr1i3	nuclear receptor subfamily 1, group I, member 3	-1.02	9.16E-01	-1.65	1.32E-03	-1.04	9.74E-01	1.10	5.11E-01	NM_022941	Rn.48759
13	87340224	1393730_at	---	Transcribed locus	-1.08	6.33E-01	1.03	8.76E-01	-1.22	9.07E-01	-1.49	4.11E-03	BI277836	---
13	88402731	1369364_at	Atp1a4	ATPase, Na+/K+ transporting, alpha 4 polypeptide	1.22	3.71E-02	-1.02	9.36E-01	1.01	9.89E-01	1.03	8.36E-01	NM_022848	Rn.10013
13	57510680	1370235_at	Dbi	diazepam binding inhibitor	1.03	8.22E-01	1.31	2.56E-02	-1.10	9.54E-01	1.34	4.23E-01	AI175009	Rn.3285
14	22786923	1381852_at	---	Transcribed locus	-1.08	6.39E-01	-1.47	2.90E-02	1.01	9.96E-01	-1.07	6.93E-01	AI059908	Rn.19484
14	2487625	1384394_at	---	Transcribed locus	-1.05	7.51E-01	-1.33	4.49E-02	1.06	9.74E-01	1.25	5.08E-01	BF548081	---
14	3241305	1373092_at	---	Transcribed locus	-1.19	3.13E-02	-1.13	4.71E-01	-1.05	9.74E-01	-1.31	1.18E-01	BE109587	Rn.8191
14	34904624	1381470_at	Kit	V-kit Hardy-Zuckerman 4 feline sarcoma viral oncogene homolog	1.04	6.88E-01	-1.38	3.19E-02	1.01	9.95E-01	-1.28	3.85E-01	AI454052	Rn.16698

14	45525305	1373079_at	---	Transcribed locus	-1.04	7.04E-01	1.41	3.64E-02	-1.10	8.96E-01	-1.05	7.68E-01	BI296427	Rn.8272
14	62661945	1368168_at	Slc34a2	solute carrier family 34 (sodium phosphate), member 2	-1.04	9.14E-01	-2.45	2.14E-02	1.01	9.91E-01	-1.02	9.33E-01	NM_053380	Rn.16933
14	80732529	1372565_at	---	Transcribed locus	-1.08	4.88E-01	1.02	9.58E-01	-1.41	3.10E-02	-1.38	1.97E-01	BI274192	---
14	99712778	1384308_at	Meis1	myeloid ecotropic viral integration site 1 homolog	-1.13	1.68E-01	-1.32	5.00E-02	1.01	9.95E-01	-1.32	2.78E-01	AW532697	Rn.32269
15	108030330	1371963_at	Pcca	propionyl-coenzyme A carboxylase, alpha polypeptide	-1.02	8.66E-01	-1.28	4.83E-02	1.04	9.88E-01	1.14	5.33E-01	BF395042	Rn.6033
15	108046123	1392725_at	RGD1304748	similar to cDNA sequence BC006662	1.07	4.91E-01	1.46	3.42E-03	-1.01	9.98E-01	1.07	7.77E-01	AA924588	Rn.32611
15	34557617	1368501_s_at	Mcpt8	mast cell protease 8	-1.01	9.73E-01	1.27	2.99E-02	-1.11	9.04E-01	-1.23	4.96E-01	NM_019323	Rn.10651
15	38386963	1390433_at	---	Transcribed locus	1.00	9.93E-01	-1.26	3.27E-02	-1.04	9.80E-01	-1.04	8.80E-01	AI102429	Rn.19335
15	4615310	1375637_at	---	Transcribed locus	1.03	7.75E-01	1.32	3.84E-02	-1.11	9.62E-01	-1.03	9.04E-01	AI010441	---
16	47618509	1385783_at	---	Transcribed locus	1.04	7.33E-01	-1.36	2.69E-02	-1.02	9.94E-01	1.06	8.39E-01	BF418697	---
16	49248790	1379888_at	---	Transcribed locus	-1.01	9.43E-01	3.01	2.77E-02	-1.06	9.70E-01	1.02	9.45E-01	AA945268	Rn.7850
16	67694238	1376829_at	---	Transcribed locus	1.08	6.99E-01	1.58	1.63E-02	1.06	9.55E-01	1.03	8.60E-01	AI716500	Rn.39005
16	73711582	1367800_at	Plat	plasminogen activator, tissue	-1.21	1.46E-02	1.13	4.77E-01	-1.16	9.44E-01	-1.18	3.97E-01	NM_013151	Rn.1002
16	74980319	1369660_at	---	Transcribed locus	-1.25	4.84E-03	-1.05	8.01E-01	1.07	9.54E-01	-1.03	9.44E-01	AF068860	Rn.31800
17	22525760	1375183_at	Idb4	Inhibitor of DNA binding 4	-1.03	8.77E-01	-1.57	4.17E-02	-1.11	8.77E-01	1.05	8.58E-01	AW916745	---
17	37145294	1373718_at	---	Transcribed locus	1.01	9.53E-01	-2.11	1.45E-02	-1.09	9.91E-01	-1.01	9.73E-01	BM384071	---
17	64000643	1382183_at	---	Transcribed locus	-1.24	3.34E-02	1.01	9.66E-01	1.05	9.85E-01	-1.13	6.46E-01	AA849479	Rn.21436
17	6546464	1393875_at	Slc35d2	Solute carrier family 35, member D2	1.07	7.27E-01	1.49	3.20E-02	-1.05	9.67E-01	-1.00	9.92E-01	AI500781	Rn.34490
17	72131830	1372869_at	---	Transcribed locus	-1.04	8.03E-01	1.30	3.64E-02	-1.06	9.44E-01	-1.21	3.06E-01	BE109202	---
17	77158160	1391056_at	---	Transcribed locus strongly similar to RAKb	-1.01	9.68E-01	1.94	6.46E-03	-1.01	9.90E-01	1.04	8.54E-01	AA817695	---
17	77553428	1372000_at	---	Transcribed locus	-1.10	3.75E-01	-1.50	2.76E-02	-1.10	9.55E-01	-1.16	6.15E-01	AI180187	Rn.3889
17	78496768	1376259_at	---	Transcribed locus	-1.01	9.39E-01	1.00	9.88E-01	1.41	1.29E-02	1.46	4.83E-01	AI171093	---
17	87556722	1387037_at	Cubn	cubilin (intrinsic factor-cobalamin receptor)	-1.08	5.14E-01	1.05	8.23E-01	-1.07	9.74E-01	1.63	3.55E-02	AF022247	Rn.3236
18	15579502	1391246_at	---	Transcribed locus	-1.04	7.90E-01	-1.02	9.43E-01	-1.34	4.98E-02	1.20	6.02E-01	BF390318	Rn.45419
18	24705577	1395147_at	---	Transcribed locus	1.02	9.35E-01	-1.35	2.20E-02	-1.09	9.54E-01	-1.12	6.02E-01	BE116565	---
18	25867266	1371412_a_at	---	Transcribed locus moderately similar to LOC461994	1.24	5.47E-03	1.58	1.03E-01	1.31	8.80E-01	1.79	1.74E-01	BE107450	Rn.8180
18	26842101	1371481_at	---	Transcribed locus	1.08	4.35E-01	1.35	3.50E-02	1.00	1.00E+00	1.06	7.08E-01	BI274372	Rn.40477
18	37168686	1368447_x_at	Spink1	serine protease inhibitor, Kazal type 1	1.02	8.70E-01	-1.69	9.56E-03	1.17	8.26E-01	1.03	9.30E-01	M27882	Rn.9767
18	37168686	1387193_a_at	Spink1	serine protease inhibitor, Kazal type 1	-1.03	8.43E-01	-1.76	9.22E-03	-1.02	9.89E-01	-1.03	9.33E-01	M27882	Rn.9767
18	75718118	1389562_at	---	Transcribed locus	-1.08	4.35E-01	-1.64	4.99E-02	-1.08	9.79E-01	1.01	9.88E-01	BF396686	Rn.43242
18	81234130	1390569_at	MGC93742	Carnosinase 1	1.33	4.97E-02	-1.48	5.44E-02	-1.47	9.54E-01	-1.05	9.44E-01	AI231438	Rn.15548
19	11266456	1371237_a_at	Mt1a	Metallothionein	1.06	7.17E-01	-1.06	8.43E-01	-1.32	9.87E-01	-1.88	2.82E-02	AF411318	Rn.54397
19	22887531	1382232_at	---	Transcribed locus	1.04	7.75E-01	1.38	3.69E-02	-1.01	9.94E-01	-1.43	1.39E-01	AI454322	Rn.20343
19	24969606	1398750_at	Calr	calreticulin	-1.07	6.06E-01	1.34	3.83E-02	1.05	9.87E-01	-1.01	9.75E-01	NM_022399	Rn.974
19	36447573	1386947_at	Cdh1	cadherin 1	-1.07	5.18E-01	-1.49	2.80E-02	-1.01	9.92E-01	-1.02	9.56E-01	NM_031334	Rn.1303
19	37243573	1398421_at	---	Transcribed locus	-1.21	2.64E-02	-1.19	3.58E-01	-1.09	9.36E-01	-1.06	8.56E-01	BM384203	Rn.22934
19	45862849	1392566_at	Maf	V-maf musculoaponeurotic fibrosarcoma (avian) oncogene homolog	-1.00	9.99E-01	-1.41	1.62E-02	-1.13	9.79E-01	-1.20	4.31E-01	AA957811	Rn.6545
19	45863020	1385243_at	Maf	V-maf musculoaponeurotic fibrosarcoma (avian) oncogene homolog	-1.05	7.91E-01	-1.38	7.49E-03	-1.09	9.87E-01	-1.38	2.65E-01	AI180340	Rn.6545
19	49687022	1370336_at	Okl38	pregnancy-induced growth inhibitor	1.16	2.30E-01	-2.16	4.02E-02	1.22	9.60E-01	1.21	3.79E-01	AY081218	Rn.15599
20	20640868	1397831_at	---	Transcribed locus	-1.42	1.51E-03	1.01	9.88E-01	-1.04	9.83E-01	1.08	8.25E-01	AW527319	Rn.46497
20	2106852	1372845_at	Rpp21	ribonuclease P 21kDa subunit (human)	1.00	9.96E-01	1.37	4.62E-02	-1.04	9.83E-01	1.06	8.35E-01	BG380782	Rn.3720

20	44212599	1386538_at	RGD1310495	similar to KIAA1919 protein	1.21	3.57E-02	1.08	6.96E-01	1.13	8.20E-01	1.03	9.48E-01	AW916827	Rn.24001
20	5132761	1376271_at	---	Transcribed locus	1.06	6.43E-01	-1.82	3.83E-02	-1.09	9.21E-01	-1.27	3.62E-01	AI178914	---
X	26384667	1386990_at	Ebp	phenylalkylamine Ca2+ antagonist binding protein	1.07	5.07E-01	1.32	3.40E-02	-1.00	1.00E+00	1.14	5.34E-01	NM_057137	Rn.19436
X	26865897	1372931_at	---	Similar to DXImx39e protein	-1.00	9.93E-01	1.39	3.65E-02	1.07	9.89E-01	1.10	5.85E-01	AI178741	Rn.6226
X	27940533	1384274_at	---	CDNA clone IMAGE:7300876	-1.02	8.30E-01	2.97	2.60E-02	1.04	9.71E-01	-1.05	8.11E-01	BG377595	Rn.20197
X	40109800	1367744_at	Maged2	melanoma antigen, family D, 2	1.11	3.28E-01	1.03	9.37E-01	1.05	9.89E-01	1.40	1.99E-02	NM_080479	Rn.11984
X	73181394	1393669_at	MGC105830	similar to Ras-related protein Rab-1B	1.17	1.61E-01	1.07	7.76E-01	1.30	5.11E-01	1.92	1.43E-03	BM383757	Rn.81353
X	89447034	1392828_at	---	Similar to OPA-containing protein 1	-1.04	7.83E-01	-1.39	3.06E-02	1.01	9.94E-01	-1.25	2.57E-01	BI283139	Rn.78344
X	91471849	1368736_at	Tsx	testis specific X-linked gene	1.41	4.03E-03	-1.08	7.74E-01	-1.01	9.91E-01	1.04	8.42E-01	NM_019203	Rn.11146
---	---	1368080_at	---	Transcribed locus	1.73	1.56E-02	1.02	9.47E-01	-1.28	9.36E-01	1.04	8.85E-01	NM_054008	Rn.3504
---	---	1371394_x_at	---	Transcribed locus	-1.83	1.75E-06	-1.53	3.63E-03	-1.15	8.19E-01	-1.12	5.28E-01	BG664827	---
---	---	1377719_a_at	---	Transcribed locus	1.03	8.61E-01	1.42	1.42E-02	1.09	9.38E-01	-1.05	8.58E-01	AA892765	---
---	---	1377720_x_at	---	Transcribed locus	1.01	9.46E-01	1.31	4.17E-02	1.06	9.64E-01	-1.02	9.36E-01	AA892765	---
---	---	1378413_at	---	Transcribed locus	-1.43	1.77E-03	-2.19	1.70E-05	-1.35	5.43E-01	-2.01	1.21E-03	BG379394	---
---	---	1384013_at	---	Transcribed locus	-1.01	9.83E-01	1.03	9.19E-01	1.05	9.63E-01	1.77	3.40E-02	BF524215	---
---	---	1384027_a_at	---	Transcribed locus	1.31	8.14E-03	-1.06	7.24E-01	-1.07	9.36E-01	1.08	6.45E-01	AI072042	Rn.20680
---	---	1384028_at	---	Transcribed locus	1.26	1.65E-02	1.05	8.82E-01	1.02	9.87E-01	-1.09	6.29E-01	AI072042	Rn.20680
---	---	1384302_at	---	NTT4 (Ntt4)	-1.19	4.51E-02	1.04	8.75E-01	1.07	8.98E-01	1.01	9.76E-01	AI712791	---
---	---	1385805_at	---	Transcribed locus	1.21	2.56E-02	1.12	4.80E-01	1.03	9.76E-01	1.03	9.35E-01	AI071958	---
---	---	1390513_at	---	Transcribed locus	1.00	9.96E-01	1.44	2.03E-02	1.03	9.74E-01	1.18	5.02E-01	BI291604	---
---	---	1391481_at	---	Transcribed locus	-1.08	8.60E-01	-1.41	6.21E-03	-1.10	9.64E-01	1.01	9.82E-01	BE104424	---
---	---	1392627_x_at	---	Transcribed locus	-1.22	6.29E-02	-1.31	2.15E-02	-1.90	1.09E-03	-1.21	4.22E-01	BI282114	---
---	---	1393394_at	---	Transcribed locus	1.08	6.82E-01	1.06	7.98E-01	-1.03	9.88E-01	-1.52	3.23E-02	AI071827	Rn.20649
---	---	1395877_at	---	Transcribed locus	1.42	1.24E-04	1.17	1.30E-01	-1.00	9.98E-01	1.48	2.28E-01	AW919687	---
---	---	1368059_at	Crym	crystallin, mu	-1.19	4.16E-02	-3.27	3.21E-03	-1.23	9.36E-01	1.34	3.56E-01	NM_053955	Rn.24561
---	---	1375751_at	---	Transcribed locus	1.30	2.10E-02	-1.20	4.56E-01	1.11	9.74E-01	1.28	6.15E-01	BE098727	---
---	---	1388103_at	Pr1	protein distantly related to to the gamma subunit family	1.42	2.14E-02	-1.14	4.31E-01	-1.00	1.00E+00	1.12	7.76E-01	AF361355	Rn.2975
---	---	1375230_at	---	Transcribed locus	-1.58	5.25E-04	-1.71	2.42E-03	-1.38	8.34E-01	1.11	7.12E-01	AA800192	---
---	---	1385270_s_at	MGC105830	similar to Ras-related protein Rab-1B	1.13	2.86E-01	1.10	6.27E-01	1.31	4.87E-01	1.80	2.31E-03	AI236576	Rn.40785
---	---	1382946_a_at	MGC105830	similar to Ras-related protein Rab-1B	1.05	7.50E-01	1.01	9.83E-01	1.27	5.43E-01	1.61	3.24E-03	AI711306	Rn.40785
